# Effects of dietary supplementation with lysozyme on the structure and function of the cecal microbiota in broiler chickens

**DOI:** 10.1371/journal.pone.0216748

**Published:** 2019-06-19

**Authors:** Yun Xia, James Kong, Guobing Zhang, Xuxiang Zhang, Robert Seviour, Yunhong Kong

**Affiliations:** 1 Department of Life Science and Technology, Kunming University, Kunming, China; 2 Computer Science, York University, York, Canada; 3 First Affiliated Hospital of Kunming Medical University, Kunming, China; 4 Microbiology Department, La Trobe University, Bundoora, Victoria, Australia; Institute of Subtropical Agriculture, Chinese Academy of Sciences, CHINA

## Abstract

Lysozyme is known to eliminate intestinal pathogens in poultry and improve their growth performance. However, whether it can replace antibiotic growth promoters without the associated risk of the emergence of antibiotic-resistant bacterial strains is not known, and the effects of lysozyme supplementation on the composition, biodiversity, and function of the chicken gut microbiota remain unclear. Here, we used the 16S rRNA gene and ITS fragment Illumina sequencing combined with transcriptomic analysis to address this issue. A total of 400 1-d-old Di Gao chicks were allocated randomly to five groups, each consisting of four replicates (20 birds/group). The chicks were fed a starter (1–21 d) and a grower (22–42 d) diet supplemented with 0 (control), 40 (LYS40), 100 (LYS100), or 200 ppm (LYS200) lysozyme, or 400 ppm flavomycin as an antibiotic control for 6 weeks. Lysozyme administration did not contribute significantly (*P* > 0.05) to the growth of the broiler chickens. No significant (*P* > 0.05) differences in the diversity and composition of the bacterial and fungal communities in the cecal microbiota of chickens in the different diet groups were found. However, lysozyme supplementation led to a significant (*P* < 0.05) enrichment of genes involved in the synthesis/degradation of bacterial outer membranes and cell walls, cross-cell substrate transport, and carbohydrate metabolic processes, thus possibly promoting the cecal microbiota carbon and energy metabolism. *Bacteroides* contributed 31.9% of glycoside hydrolase genes (17,681–24,590), 26.1% of polysaccharide lyase genes (479–675), 20.7% of carbohydrate esterase genes (3,509–4,101), 8.8% of auxiliary activity genes (705–1,000), 16.2% of glycosyltransferase genes (5,301–6,844), and 13.9% of carbohydrate-binding module genes (8838–15,172) identified in the cecal samples. Thus, they were the main players in the breakdown of non-starch polysaccharides in the cecum, although *Parabacteroides*, *Alistipes*, *Prevotella*, *Clostridium*, *Blastocystis*, *Barnesiella*, *Blautia*, *Faecalibacterium*, *Subdoligranulum*, *Megamonas*, *Eubacterium*, *Ruminococcus*, *Paenibacillus*, *Bifidobacterium*, *Akkermansia*, and other bacteria also participated.

## Introduction

Sub-therapeutic antibiotics (AGPs) have been used in the poultry industry as growth promoters for more than 60 years in attempts to increase meat production [1]. The practice has led to the emergence of antibiotic-resistant pathogens, which pose a potential threat to human health [2], resulting in increased bans on their use in animal feed worldwide [1,3]. The poultry industry is in need of non-antibiotic alternatives to maintain animal health and improve feed conversion. Therefore, research on and development of dietary supplements, including probiotics, prebiotics, herbs, and exogenous enzymes, has attracted increased attention [4].

As a natural antibacterial enzyme, lysozyme exerts bacteriolytic activity directly by hydrolyzing the β-1,4-glycosidic linkage between N-acetylmuramic acid and N-acetyl glucosamine of bacterial peptidoglycans in the cell wall [5] and indirectly by stimulating macrophage phagocytic function [6]. Dietary supplementation with lysozyme has been reported to improve the immune response, help maintain gut barrier function, and improve growth performance in weaned pigs [7,8]. It has also been shown to reduce pathogen counts in the ceca of broiler chickens [9,10], enhance their gut antioxidant status and nonspecific immunity, and improve their growth performance [11]. Therefore, lysozyme appears to have the potential to replace AGPs in efforts to increase production in the poultry industry [10].

The gut microbiota of chickens play important roles in nutrient assimilation, vitamin and amino acid production, and prevention of pathogen colonization [12]. Limited studies have been carried out on the effect of dietary lysozyme on the intestinal microbiota in broiler chickens. Almost all these studies focused on small numbers of functional populations, including *Clostridium perfringens*, *Lactobacillus* spp., and *Bifidobacterium* [9–11], using culture-dependent techniques. Consequently, the overall effects of lysozyme on the composition and diversity of their gut microbiota remain unclear. In particular, little information exists about its effects on enzyme expression and metabolism of the microbiota.

In this study, we used 16S rRNA and ITS (internal transcribed spacer) fragment Illumina sequencing combined with transcriptomic analysis to investigate the impact of dietary supplementation with lysozyme on the composition, diversity, and function of the gut microbiota of broiler chickens. We compared the enrichment profiles of the gene ontology (GO) terms and Kyoto Encyclopedia of Genes and Genomes (KEGG) pathways of their cecal microbiota with and without lysozyme exposure with the aim of identifying which expressed genes and metabolic pathways were affected by lysozyme exposure. We believe the information presented here will improve our understanding of the interactions between lysozyme and the gut microbiota and its effect on animal growth performance and health.

## Materials and methods

### Ethics statement

Animal care and experimental protocols were approved by the Animal Care and Use Committee of Yunnan Agriculture University, China. All efforts were made to minimize animal suffering.

### Animals, experimental design, and diets

A total of 400 1-d-old male Di-Gao chicks were obtained from a local commercial hatchery (Kunming Yunling Guangda Breeder Ltd., Kunming, China) and randomly divided into five dietary groups with 80 birds placed in each group on the basis of similar body weights (46±5 g). Birds in each group were arranged in four identical stainless-steel cages (20 birds in each cage) with plastic mesh floors (1.5 m^2^ floor area/pen) and with the same number of nipple drinkers and feed hoppers. Room temperature was maintained at 34°C for the first 5 d and then gradually reduced to 26°C after the 3^rd^ week. The chicks were exposed to continuous light. Corn-soybean-based basal diets (BD) (S1 Table) were formulated for the starter phase (1–21 d) [23.6% CP (crude protein) and 3094 kcal/kg metabolizable energy (ME) diet] and the grower phase (22–42 d) (22.6% CP and 3110 kcal/kg ME diet). The nutrients in the diets met the requirements for broiler chickens as recommended by National Research Council (NRC) [13]. The ingredients and nutrient composition of the BD were analyzed according to AOAC methods [14]. Birds consumed feed and water *ad libitum*. Body weight and feed intake were recorded weekly during the experiments.

Of the five dietary groups, the control group was fed the BD only. The antibiotic control group was fed the BD plus 400 ppm flavomycin prepared with 10% flavomycin (Lu-Kang Biotechnology Co., Ltd., Shandong, China), while the remaining three groups were supplied continuously with the BD supplemented with 40, 100, or 200 ppm lysozyme. Concentrated (≥90%, ≥40,000 units/mg protein) lysozyme powder from chicken egg white (Sigma) was first mixed with 5 kg corn powder before being mixed with the BD in a diet mixer to reach the designated concentrations by replacing an equivalent mass of corn power from each diet. All animals were vaccinated against the ND (Newcastle disease) virus (Hitchner B1, Intervet, Boxmeer, the Netherlands) at days 10 and 26 using a spraying method, and coccidiostats (lasalocid sodium; 5 g/kg) were included in the diets after days 11, 15, and 21.

### Sample collection

At the end (42 d) of the experiment, birds randomly chosen from each group were intravenously injected with sodium pentobarbital (50 mg/kg) and immediately necropsied to collect cecal samples. For RNA analysis, cecal samples (*n* = 3) from each group were taken aseptically, kept in Eppendorf tubes, and immersed instantly in liquid nitrogen for use in mRNA expression analysis. For microbial diversity analysis, cecal samples (*n* = 4) from each group were taken aseptically, kept in Eppendorf tubes, and frozen at -80°C until used for 16S rRNA and ITS fragment sequencing.

### DNA extraction, PCR amplification, and Illumina sequencing

Total genomic DNA of cecal samples was extracted using the Qiagen QIAamp Fast Stool Mini Kit (Qiagen, Shanghai, China) according to the manufacturer’s protocol. V3-V4 hypervariable regions of the bacterial 16S rRNA gene were amplified with the primer set 338F (5ʹ-ACTCCTACGGGAGGCAGCAG-3ʹ) and barcoded 806R (5ʹ-GGACTACHVGGGTWTCTAAT-3ʹ) [15] under the following PCR cycling conditions: initial denaturation at 94°C for 3 min followed by 5 cycles of denaturing at 94°C for 10 s, annealing at 55°C for 15 s, and extension at 72°C for 30 s before a final extension at 72°C for 5 min. Fungal ITS regions were amplified with the primers ITS3_KYO2 and ITS4_KYO3 [16]. The following PCR cycling conditions were used: an initial denaturation of 15 min at 95°C; followed by 30 cycles of 30 s at 95°C, 30 s at either 51°C or 55°C, and 30 s at 72°C; and final elongation for 5 min at 72°C. Purification, quantification, and sequencing of 16S rRNA and ITS amplicons were carried out following the procedure described by Song et al. [17]. The 16S rRNA and ITS amplicon sequences have been deposited in the NCBI Sequence Read Archive under the Submission ID: PRJNA523884.

### 16S rRNA gene and ITS sequence analyses

The 16S rRNA and ITS sequences were pair-end assembled and checked using Flash software [18] with the following criteria: (1) reads were truncated at any site receiving an average quality score <20 over a 50 bp sliding window, and any read containing a N-base was removed; (2) pair-end assembly was performed by setting a minimum overlap length of 10 bp; (3) the maximum mistake match ratio was set at 0.2, and unqualified reads were discarded; and (4) sequences matched perfectly with the index sequences and with no more than one mismatch error present in the forward primer sequences were used. The trimmed sequences were uploaded into Usearch 7.0 [19] and analyzed with RDP classifier (Release 11.1 http://rdp.cme.msu.edu/) against the database silva128/16s_bacteria and silva128/18s_eukaryota for bacterial and fungal OTU (operational taxonomic unit) clustering, respectively, using a confidence threshold of 0.7. The amplicon sequences were grouped into OTUs at a 97% identity threshold (3% dissimilarity levels). Any OTU represented by ≤3 sequences was removed. Biodiversity indices, including the Cho index, Shannon index, and coverage ratios, were calculated with Mothur [20] following the procedures provided and again after applying a 97% identity threshold.

### RNA extraction, library preparation, and sequencing

Total RNA was isolated using TRIzol reagent (ThermoFisher Scientific, Shanghai, China) after grinding the frozen cecal sample into a fine powder in a liquid nitrogen environment. For each dietary treatment, RNAs were extracted from the cecal samples of three birds. Equimolar portions of RNAs from three birds were combined for transcriptomic analysis. The rationale for pooling RNA samples from individual samples was that it is cost effective and provides genome-wide information about potentially functionally relevant variations [21,22]. The main purpose of the RNA-seq analysis performed in the current study was to comprehensively screen the differentially expressed genes (DEGs), and to investigate changes in the expression of genes involved in metabolic pathways in the cecal microbiota as a result of dietary supplementation with lysozyme. The quality and quantity of extracted RNAs were monitored using 1% agarose gels before rRNAs were removed using Ribo-Zero rRNA Removal Kits (Qiagen, Shanghai, China) following the manufacturer’s instructions. For each sample, a library with about 200 bp insert sizes was prepared with a TruSeq RNA Sample Prep Kit (Qiagen, Shanghai, China), and mRNAs were amplified with a “bridge PCR” using a HiSeq 3000/4000 Cluster Kit (Illumina, Shanghai, China) according to the manufacturer’s instructions. The cDNAs obtained were subjected to 2 × 100 bp paired-end (PE100) sequencing on a HiSeq 2000 instrument (Illumina, Shanghai, China) using HiSeq 3000/4000 SBS Kits (Illumina, Shanghai, China). The raw sequence data are publicly available in the NCBI Short Reads Archive (SRA) under the accession number: PRJNA540969.

### Differential gene expression analysis and taxonomy of genes

To ensure high-quality data, we truncated the cDNA sequences at the 3ʹ and 5ʹ ends using Seqprep (https://github.com/jstjohn/SeqPrep), removed (reads containing adapter contamination or at least 10 Ns from the raw data (FASTQ format) using Sickle (https://github.com/najoshi/sickle), and rRNA reads using SortMeRNA (http://bioinfo.lifl.fr/RNA/sortmerna/) against the Silva SSU and LSU databases. High-quality reads were assembled using Trinity (http://trinityrnaseq.github.io/, version: trinityrnaseq-r2013-02-25) under the default setting. The sequences assembled were annotated using TransGeneScan (http://sourceforge.net/projects/transgenescan/) for ORF prediction. Gene sequences from different cecal samples were compared using CD-HIT (http://www.bioinformatics.org/cd-hit/) to build non-redundant catalogs with 95% identity and 90% coverage. Gene expression levels in the different cecal microbiota were expressed as Fragments Per Kilobase of Exon Model per Million Mapped reads (FPKM) and calculated with RSEM (http://deweylab.biostat.wisc.edu/rsem/) using the following equation:

$$FPKM=\frac{total\;exon\;Fragments}{mapped\;Fragments\;(millions)*exon\;length\;(KB)}$$

Taxonomical analyses of the genes and construction of their expressed abundances at different phylogenetic levels were performed using BLASTP (http://blast.ncbi.nlm.nih.gov/Blast.cgi, BLAST Version 2.2.28+) against the NCBI nr database.

### Functional enrichment and annotation analyses

To gain insight into the biological functions of the DEGs, the GO terms and KEGG pathways were determined using GOATOOLS [23] and KOBAS 2.0 [24], respectively, using the default settings.

### Carbohydrate-active enzymes

Annotations of carbohydrate-active enzymes were conducted using hmmscan (http://www.hmmer.org/) against the CAZy database V5.0 (http://www.cazy.org/) with an e-value cutoff of 1e^-5^.

### Statistical analyses

Principal coordinate analyses (PCoA) of individual bacterial and fungal microbiota, Venn diagrams of the OTU distribution, and volcano diagrams of DEGs were performed or constructed using the R package software (https://www.r-project.org). Statistical differences in diversity indices and phylogenetic compositions between bacterial and fungal communities in the cecal microbiota of birds fed different diets were determined using Student’s *T* test and one-way ANOVA, respectively. *P* < 0.05 was considered statistically significant. The DEGs were declared at a significance level of |log_2_ (fold change)| > 1, FDR < 0.05. In the GO and KEGG enrichment analyses, Fisher’s exact test was used to estimate the significance of enrichment of GO terms and KEGG pathways. Again, *P* < 0.05 was considered statistically significant.

## Results

### Growth performance

Dietary supplementation with 40, 100, and 200 ppm lysozyme and 400 ppm flavomycin had no greater (*P* > 0.05) effects on chicken body weight, feed intake, or feed conversion at the end of experiment (i.e., 42 d) (Table 1).

**Table 1:** Effects of dietary supplementation with 0 (control), 40 ppm (LYS40), 100 ppm (LYS100), and 200 ppm (LYS200) lysozyme and 400 ppm flavomycin (FLA) on the growth performance (1–42 d) of broiler chickens.

	Control	FLA	LYS40	LYS100	LYS200	SEM	*P* value
**BW gain (g)**	1078.74	1205.07	1142.1	1143.36	1156.48	12.1	0.084
**Feed intake (g)**	2275.28	2353.11	2188.25	2333.94	2135.33	34.7	0.21
**Feed conversion** **(feed/gain)**	2.11	1.95	1.92	2.04	1.85	0.03	0.099

Note: Data are the least-square means of five observations for all treatments. SEM, standard error of the mean.

### Composition and biodiversity of the cecal microbiota

**Fig 1:**
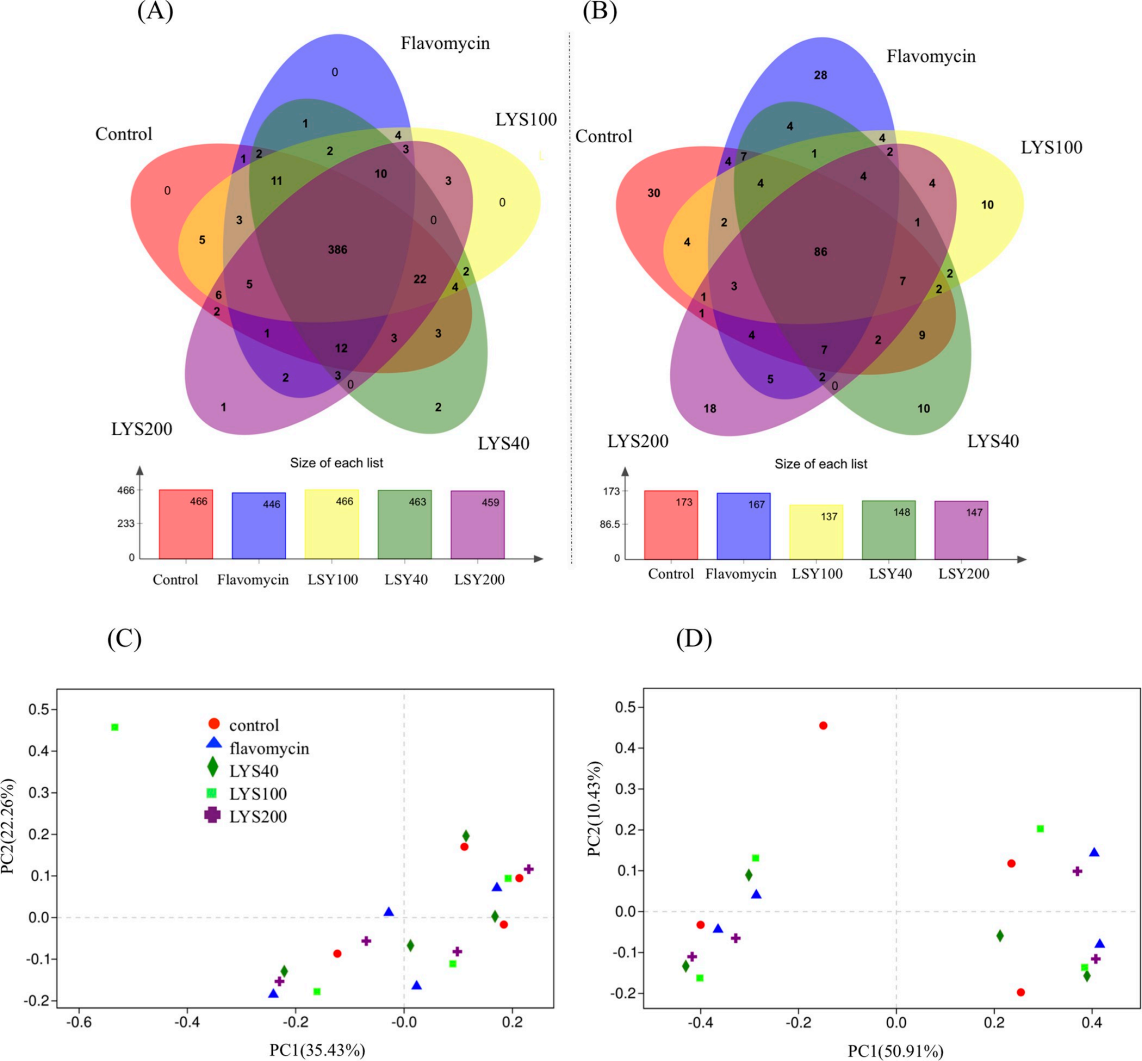
Effects of dietary supplementation with lysozyme and flavomycin on the composition and distribution of bacterial and fungal OTUs in the cecal microbiota of broiler chickens. (A&B) Venn diagrams showing the occurrence of bacterial (A) and fungal (B) OTUs identified in 16S rRNA and ITS fragment sequencing of cecal microbiota of chickens fed a basal diet supplemented with or without 40 (LYS40), 100 (LYS100), or 200 ppm (LYS200) lysozyme or 400 ppm flavomycin. (C&D) Grouping of cecal bacterial (C) and fungal (D) communities based on principle component analyses of Illumina sequencing of 16S rRNA amplicons (V3-V4 region) and ITS fragment sequencing, respectively.

Illumina sequencing analyses of the 16S rRNA gene and ITS fragment amplicons were used to investigate the effects of dietary supplementation with lysozyme on the composition and diversity of the cecal microbiota in broiler chickens. In total, 491,774 bacterial 16S rRNA and 715,291 fungal ITS high-quality reads were obtained from 20 cecal DNA samples taken from birds fed one of the five different dietary treatments (four replicates for each treatment). Phylogenetic analyses identified a total of 499 bacterial OTUs (Fig 1A) belonging to 119 genera and 12 phyla and 268 fungal OTUs (Fig 1B) belonging to 29 genera and 7 phyla. No significant (*P* > 0.05) differences in bacterial and fungi OTU numbers, Shannon indices, or Chao1 values (Table 2) were found among the four replicate samples from the five dietary groups given the different feeds. The coverage values calculated for individual microbiota were all above 99%, indicating that the sequencing depths adequately covered the bacterial and fungi diversity in the microbiota of the broiler chickens. PCoA revealed no clear clustering patterns for both bacteria and fungi among the four replicate microbiota in the same dietary treatment group (Fig 1C and 1D).

**Table 2:** Biodiversity indices of the gut microbiota in broiler chickens fed a basal diet supplemented with 0 (control), 40 (LYS40), 100 (LYS100), or 200 ppm (LYS200) lysozyme or 400 ppm flavomycin.

Index* (Mean±STD)	Control	Flavomycin	LYS40	LYS100	LYS200
**Bacteria**					
**Shannon**	3.76±0.29	3.66±0.26	3.98±0.26	3.75±0.38	3.81±0.22
**Chao1**	329±98	312±93	331±109	311±106	316±108
**Observed species**	293±100	278±87	296±89	284±97	278±103
**Coverage (%)**	99.83	99.84	99.8	99.84	99.79
**Fungi**					
**Shannon**	2.40±0.22	2.64±0.20	2.42±0.14	2.24±0.34	2.38±0.13
**Chao1**	88±27	88±3	78±36	72±33	80±25
**Observed species**	86±28	84±34	75±32	65±26	77±22
**Coverage (%)**	99.99	99.98	99.98	99.98	99.98

* Each biodiversity index value is the mean value of four replicates, and the standard deviation is listed after ±.

**Fig 2:**
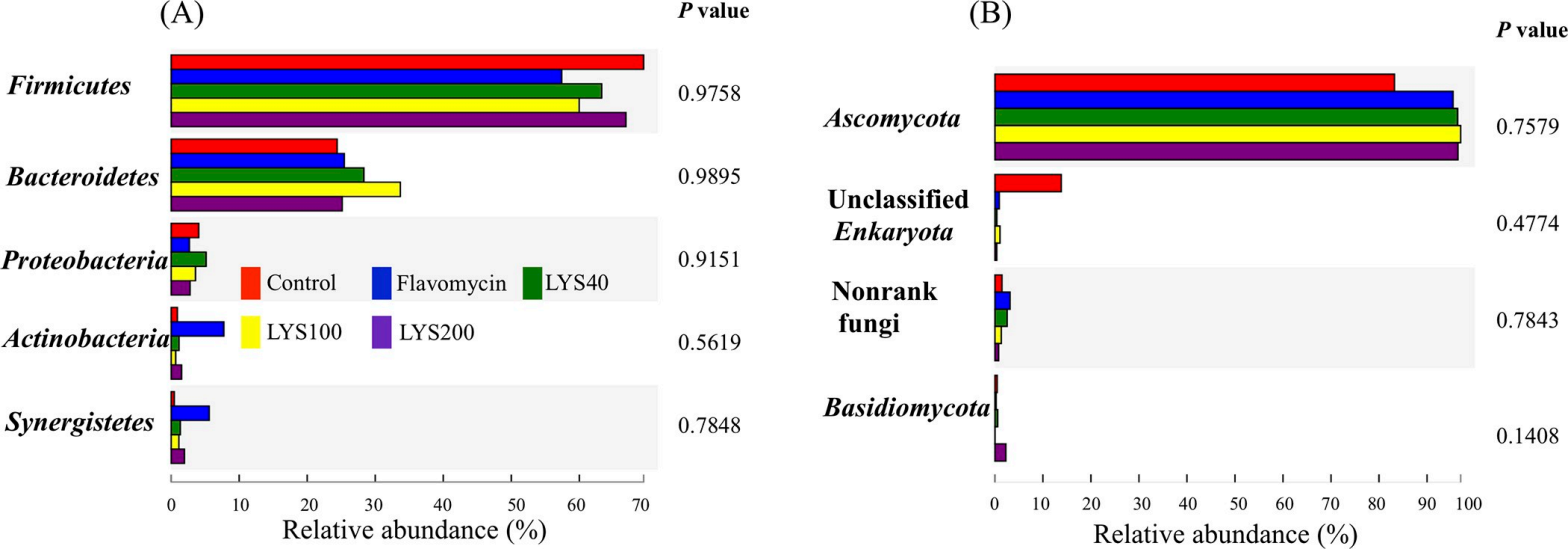
Phylogenetic classification and differences in the cecal microbiota of broiler chickens fed a basal diet supplemented with or without 40 (LYS40), 100 (LYS100), or 200 ppm (LYS200) lysozyme or 400 ppm flavomycin. Phylum compositions of the bacterial (A) and fungal (B) communities in the cecal microbiota characterized based on sequencing of 16S rRNA amplicons (V3-V4 region) and ITS fragment sequencing.

**Fig 3:**
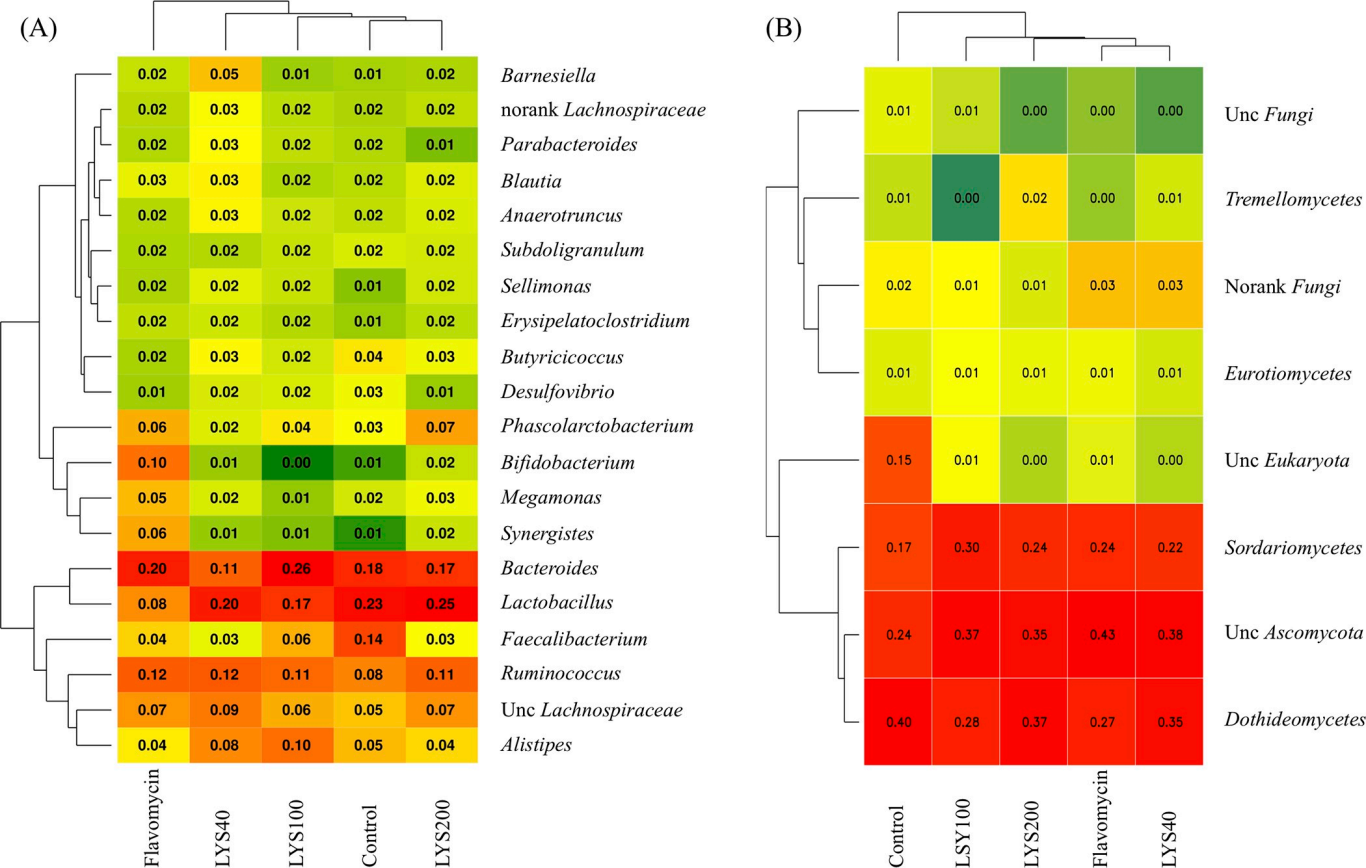
**Clustering analysis of the compositions of the abundant (>1%) bacterial genera (A) and fungal orders (B) in the cecum of chickens fed a basal diet plus 0 (control), 40 (LYS40), 100 (LYS100), or 200 ppm (LYS200) lysozyme or 400 ppm flavomycin.** Values in individual squares represent relative percentage abundances of individual genera or orders. Unc represents unclassified.

Dietary supplementation with lysozyme and flavomycin had no statistically (*P* > 0.05) significant effect on the compositions of the bacterial and fungal communities. In all the cecal microbiota examined, bacteria belonging to *Firmicutes* (58.8–70.9%) and *Bacteroidetes* (21.9–33.5%) constituted the majority of community members, and the remainder were mainly members of *Proteobacteria* (2.5–4.4%) and *Actinobacteria* (0.7–8.2%) (Fig 2A). Fungal members from the phylum *Ascomycota* comprised most (82–97%) of the fungal community, while the rest were *Basidiomycota* (0.1–2.1%) and as yet unclassified fungi (Fig 2B). Bacteria belonging to *Bacteroides* (average 15.27%), *Lactobacillus* (average 14.99%), *Ruminococcus* (average 8.90%), unclassified *Lachnospiraceae* (average 5.70%), *Alistipes* (average 5.17%), and *Faecalibacterium* (average 5.03%) were abundant in the cecal samples from the different dietary treatment groups (Fig 3A). No significant differences (*P* > 0.05) in bacterial relative percentage abundances were found at the phylum (Fig 2A) or genus (Fig 3A) level among the five microbiota from broilers fed the different diets. Most of the fungi could only be classified at the order level, and they belonged mainly to unclassified *Ascomycota* (average 35.13%), *Dothideomycetes* (average 33.60%), and *Sordariomycetes* (average 23.28%) (Fig 3B). Similarly, no significant differences (*P* > 0.05) in fungal relative abundances were found at the phylum (Fig 2B) or order level (Fig 3B).

### Differential gene expression in the cecal microbiota of broilers fed different diets

High-throughput RNA sequencing used to determine the gene expression profiles of the cecal microbiota yielded c.a 294 million raw paired-end reads. After quality control, between 51 and 66 million high-quality reads were obtained for each of the five microbiota. After removal of rRNA reads, each sample had 22 to 41 million reads, which were assembled to yield 62,000 to 113,000 cDNA reads. These were then used for ORF (open reading frame) prediction. Each sample yielded 42,000–80,000 ORFs that were used for gene expression analysis.

The relative expression levels of genes were normalized as FPKM. In total, 226,623 transcripts were identified among the five cecal microbiota. Of these, 191,363 genes were differentially expressed following supplementation with LYS40, with 95,432 genes up- and 95,931 genes down-regulated; 189,553 genes were differentially expressed following supplementation with LYS100, with 94,975 genes up- and 94,578 genes down-regulated; 180,252 genes were differentially expressed following supplementation with LYS200, with 80,538 genes up- and 99,714 genes down-regulated; and 178,365 genes were differentially expressed following supplementation flavomycin, with 78,422 genes up- and 99,943 genes down-regulated (S1 Fig).

### Gene annotations and enrichment analyses

All the DEGs were assigned to GO terms on the basis of GO annotation. GO enrichment analysis (Table 3) was carried out using three categories: molecular function (MF), cellular component (CC), and biological process (BP). Comparison with the control microbiota of birds fed only the BD revealed that no GO functions belonging to any of these three categories were enriched significantly following LYS40 and LYS100 supplementation (Table 3). However, when LYS200 was administered, GO functions belonging to all three categories were enriched significantly. As shown in Table 3, based on the numbers of genes enriched in each subcategory, the representative GO functions significantly (*P* < 0.05) enriched by LYS200 exposure included “oxidoreductase activity” (553 genes) in MF; “external encapsulating structure part” (“EESP”) (124 genes) in CC; and “oxidation-reduction process” (663 genes), “carbohydrate metabolic process” (527 genes), and “transport” (656 genes) in BP. In contrast, supplementation with flavomycin did not significantly (*P* > 0.05) enrich the expression of genes involved in “oxidoreductase activity” or “transport” as supplementation with lysozyme did, but instead genes involved in “EESP” (136 genes), and “oxidation-reduction process” (810 genes), and “carbohydrate metabolic process” were enriched (665 genes).

**Table 3:** Enrichment of Gene Ontology (GO) functions in the cecal microbiota of broiler chickens fed a basal diet supplemented with 40 (LYS40), 100 (LYS100), or 200 ppm (LYS200) lysozyme or 400 ppm flavomycin determined by comparison with the GO functions observed in the cecal microbiota of broiler chickens fed only the basal diet (control).

Gene category	Description	*P* values corrected*			
		Flavomycin	LYS40	LYS100	LYS200
**Molecular** **function**	Oxidoreductase activity	-	-	-	0.0012(553)
	Receptor activity	-	-	-	0.0141(118)
	RNA helicase activity	-	-	-	0.0434(10)
	Glyceraldehyde-3-phosphate dehydrogenase (NAD+) activity	5.46E-04(21)	-	-	-
	Acid phosphatase activity	0.0192(15)	-	-	-
	RNA-directed RNA polymerase activity	0.0494(17)	-	-	-
**Cellular** **component**	External encapsulating structure part	5.97E-04(136)	-	-	9.10E-07(124)
	Outer membrane	0.0072(127)	-	-	3.5E-04(110)
	Cell outer membrane	6.58E-04(116)	-	-	1.86E-06(105)
	Other organism cell membrane	0.0323(10)	-	-	-
	Other organism membrane	0.0323(10)	-	-	-
	Host cell membrane	0.0323(10)	-	-	-
	RNA-directed RNA polymerase complex	0.0494(17)	-	-	-
**Biological** **process**	Oxidation-reduction process	7.49E-06(810)	-	-	2.28E-06(663)
	Carbohydrate metabolic process	2.24E-06(665)	-	-	1.37E-05(527)
	Single-organism carbohydrate metabolic process	2.26E-06(473)	-	-	2.09E-04(367)
	Monocarboxylic acid metabolic process	0.0017(378)	-	-	-
	Generation of precursor metabolites and energy	2.05E-06(353)	-	-	3.72E-04(273)
	Monosaccharide metabolic process	6.03E-05(325)	-	-	0.0078(252)
	Hexose metabolic process	2.22E-04(306)	-	-	0.0040(242)
	Carbohydrate biosynthetic process	7.5E-05(275)	-	-	-
	Pyruvate metabolic process	8.05E-04(266)	-	-	-
	Glucose metabolic process	0.0076(236)	-	-	-
	Cellular carbohydrate metabolic process	0.0074(232)	-	-	0.0367(185)
	Carbohydrate catabolic process	0.0040(224)	-	-	-
	Single-organism carbohydrate catabolic process	0.0036(216)	-	-	-
	Monosaccharide biosynthetic process	0.0234(171)	-	-	-
	Gluconeogenesis	0.0159(170)	-	-	-
	Hexose biosynthetic process	0.0159(170)	-	-	-
	Glycolytic process	8.86E-04(161)	-	-	-
	Glycogen metabolic process	0.0368(69)	-	-	0.044(57)
	Energy reserve metabolic process	0.0368(69)	-	-	0.044(57)
	Glycogen biosynthetic process	0.0252(58)	-	-	-
	Interspecies interaction between organisms	0.0019(27)	-	-	9.3E-04(24)
	Multi-organism cellular process	-	-	-	0.0069(25)
	Transport	-	-	-	0.0445(656)
	Interaction with host	0.0064(11)	-	-	-
	Entry into host cell	0.0084(10)	-	-	-
	Transcription, RNA-templated	0.0494(17)	-	-	-

* values in parentheses represent the numbers of genes enriched in individual GO functions.

KEGG enrichment analysis (Table 4) was used to investigate the effect of dietary supplementation with lysozyme on metabolic and signaling pathways in the cecal microbiota of broiler chickens. Lysozyme supplemented at all three different dosages significantly (*P* < 0.05) enriched the expression of genes for “carbon metabolism” (average 460 genes), including mainly “starch and sucrose metabolism” and “glycolysis.” Only LYS40 significantly (*P* < 0.05) enriched the expression of genes for “methane metabolism” (148 genes), while LYS100 enriched (*P* < 0.05) the expression of genes for “ABC transporters” (143 genes) and LYS200 enriched (*P* < 0.05) the expression of genes for “carbon fixation pathways in prokaryotes” (226 genes). LYS40 and LYS100 both enriched (*P* < 0.05) the expression of genes for “two component system” (average 107 genes), while dietary supplementation with flavomycin significantly *(P* < 0.05) enriched the expression of genes for “glycosaminoglycan degradation” (39 genes) and both “glycosphingolipid biosynthesis-globo (37 genes) and -ganglio (30 genes) series” (Table 4).

**Table 4:** Enrichment of Kyoto Encyclopedia of Genes and Genomes (KEGG) pathways in the cecal microbiota of broiler chickens fed a basal diet supplemented with 40 (LYS40), 100 (LYS100), or 200 ppm (LYS200) lysozyme or 400 ppm flavomycin determined by comparison with the KEGG functions observed in the cecal microbiota of broiler chickens fed only the basal diet (control).

KEGG pathways	*P* values corrected*			
	Flavomycin	LYS40	LYS100	LYS200
**Carbon metabolism**	-	1.51E-04(485)	0.0125(385)	1.53E-05(511)
**Starch and sucrose metabolism**	-	1.51E-04(126)	0.0010(121)	0.0075(134)
**Glycolysis / Gluconeogenesis**	-	1.51E-04(177)	0.0051(154)	0.0485(171)
**Galactose metabolism**	-	1.51E-04(91)	-	-
**Other glycan degradation**	-	-	-	0.0063(71)
**Carbon fixation pathways in prokaryotes**	-	-	-	0.0109(226)
**Amino sugar and nucleotide sugar metabolism**	-	-	0.0495(135)	0.0063(175)
**Methane metabolism**	-	1.51E-04(148)	-	-
**Glycosaminoglycan degradation**	0.0177(39)	-	0.0495(27)	0.0075(36)
**Sphingolipid metabolism**	-	1.51E-04(47)	-	-
**Glycosphingolipid biosynthesis—globo series**	0.0177(37)	1.51E-04(33)	0.0051(32)	0.0054(38)
**Glycosphingolipid biosynthesis—ganglio series**	0.0180(30)	-	-	0.0063(30)
**Glycerolipid metabolism**	-	-	0.0140(31)	-
**Flagellar assembly**	-	9.75E-05(58)	0.0193(39)	0.0063(49)
**Two-component system**	-	1.51E-04(121)	0.0495(92)	-
**ABC transporters**	-	-	4.35E-06(143)	-
**Phosphotransferase system (PTS)**	-	-	0.0125(32)	-

* values in parentheses represent the numbers of genes enriched in individual KEGG pathways.

### Carbohydrate-active enzymes

**Fig 4:**
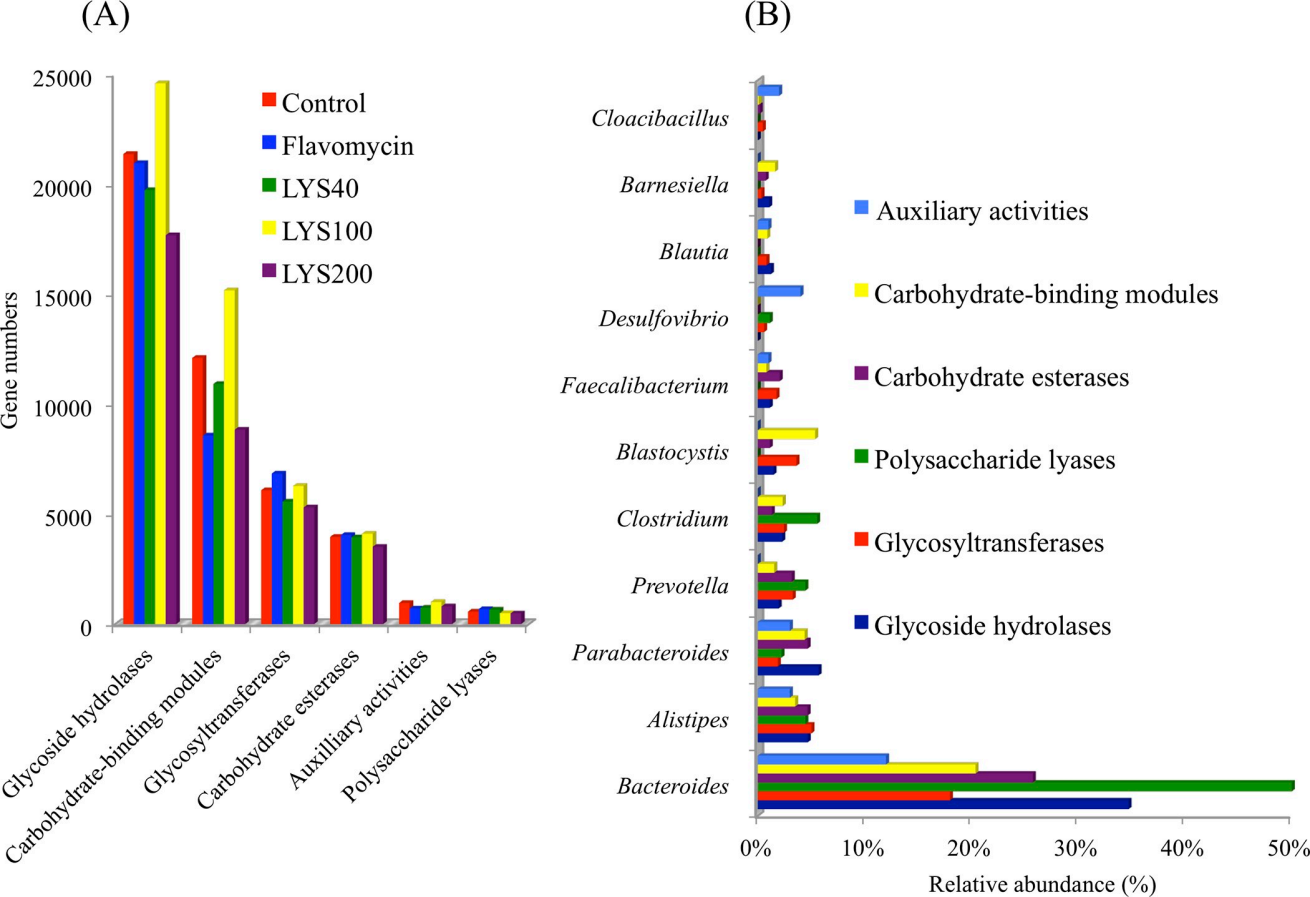
Expression and classification of carbohydrate-active enzymes in the cecal microbiota of broilers fed a corn-based diet supplemented with 0 (control), 40 (LYS40), 100 (LYS100), or 200ppm (LYS200) lysozyme or 400 ppm flavomycin. (A) Expression of carbohydrate-active enzyme genes in the cecal microbiota of broilers with different dietary treatments. (B) Taxonomy and relative abundances of the classifiable carbohydrate-active enzyme genes identified in cecal microbiota of broilers with different dietary treatments.

The carbohydrate-active enzymes (CAEs) analyzed were glycoside hydrolases (GH), carbohydrate-binding modules (CBM), glycosyltransferases (GT), carbohydrate esterases (CE), polysaccharide lyases (PL), and auxiliary activities (AA). The numbers of CAE genes expressed in each of the individual cecal samples and their taxonomy (at the genus level) are shown in Fig 4A. Of the CAEs identified, the GH genes were the most abundant (17,681–24,590), followed by CBM (8838–15,172), GT (5,301–6,844), CE (3,509–4,101), AA (705–1,000), and PL (479–675) genes.

Dietary supplementation with lysozyme or flavomycin affected the gene expression of CAEs to different degrees (Fig 4A). The cecal microbiota in birds supplemented with 40 ppm lysozyme had 16% more PL genes but 10% fewer CBM and 23% fewer AA genes than those in the control birds. The cecal microbiota in birds supplemented with 100 ppm lysozyme contained 15% more GH and 25% more CBM genes but 23% fewer PL genes than those in the control birds. Supplementation with 200 ppm lysozyme decreased (11–27% less) the number of each of the CAEs. The cecal microbiota in birds supplemented with flavomycin contained 12% more GT and 19% more PL genes but 10% fewer CBM and 23% fewer AA genes than those in the control birds.

### Taxonomy of CAEs

A major fraction (70%) of GH genes identified in all cecal samples were assigned to bacteria, and the remaining 30% could not be assigned to any microorganisms (S2 Table). Of the classifiable GH genes (Fig 4B), members of the *Bacteroides* contributed 34.7%, the *Parabacteroides* 5.7%, *Alistipes* 4.7%, *Clostridium* 2.5%, *Prevotella* 1.9%, *Blastocystis* 1.5%, *Blautia* 1.2%, *Faecalibacterium* 1.1%, and *Barnesiella* 1.1%. Other bacterial genera, including *Ruminococcus*, *Akkermansia*, and *Megamonas* each contributed a negligible amount (<0.5% of total). A minor fraction (6.4%) of classifiable GH genes could only be assigned to taxa above genus level (S2 Table). A significant amount of the GT (47.3%) (S3 Table), PL (27.8%) (S4 Table), CE (17.7%) (S5 Table), CBM (41.1%) (S6 Table), and AA (50.0%) (S7 Table) genes could not be classified phylogenetically. Of those that were classifiable, and as found for the GH genes, the *Bacteroides* accounted for 12.0–50.0%, *Alistipes* 3.0–5.0%, *Parabacteroides* 1.9–5.7%, *Prevotella* 0–4.4%, *Clostridium* 0–5.6%, *Blastocystis* 0–5.4%, and *Faecalibacterium* 0–2.1%.

## Discussion

The effect of the cecal microbiota in chickens on their growth performance has attracted considerable interest, especially with the availability of next-generation sequencing technology [12,25]. The anaerobic cecum is where urea is recycled, B vitamins and essential amino acids are synthesized, and water regulation occurs, and it is where complex non-starch polysaccharides (NSPs) present in the grain feed are degraded and the products are fermented to generate short-chain fatty acids (SCFAs) of nutritional value. Consequently, the cecal microbiota is considered to have a marked impact on chicken growth and wellbeing [12,26,27,28]. Based on the data generated in other studies (e.g., [29,30]), it is generally agreed that the chicken cecal bacterial microbiome is always dominated by members of the phyla *Bacteroidetes*, *Firmicutes*, and *Proteobacteria*, with a lower abundance of *Actinobacteria*, although their relative abundances may change. The same general pattern was seen in the control broilers used in this study (Fig 2). At the genus level (Fig 3), members of *Bacteroides*, *Lactobacillus*, *Ruminococcus*, *Alistipes*, and *Faecalibacterium* constituted a major fraction of the total bacteria in different cecal samples. These results are also generally in line with those from other studies [29,30].

Surprisingly, there was a high biodiversity of fungi (268 fungal OTUs belonging to 29 genera and 7 phyla) in the chicken cecal microbiota in our study. What role these fungi might play there is poorly understood, as similar published data are scarce. Byrd et al. [31] using culture-dependent methods, managed to identify a large number of fungi in the cecum of broilers using rep-PCR. However, their culture method was not carried out under the anaerobic conditions found in the cecum, and most identified fungi appeared to be common highly sporulating airborne fungi, whereas some could not be identified. It seems probable that these are not true members of the cecal microbiome; it is more likely that their presence was the result of their spores being ingested by the chicken either accidentally or as contaminants in the grain feed, which then eventually germinated in the culture conditions used for their retrieval. In our study, culture-independent methods based on ITS fragments were used. The most abundant fungal populations recovered are shown in Fig 3B, and not surprisingly, most could not be identified below the level of Order. However, their role in cecal microbiology is not clear. As with the data of Byrd et al. [31], it seems probable that most are unlikely to grow under the anaerobic conditions in the cecum and arrived there after ingestion as dormant spores, which are able to withstand the hostile anaerobic and low pH conditions encountered within the cecum. Consequently, these data raise several interesting questions, including whether any of the fungi found there are strict anaerobes and whether they exist within the cecum as actively growing hyphae.

The cecal microbiota is affected by many factors, including bird behavior, season, diet, and bird age [12,25]. Many attempts have been made to manipulate the bacterial cecal community to improve chicken growth rates and increase body weight. For example, strategies involving changing the diet or adding prebiotic and probiotic substrates and antibiotics to the feed are commonly reported. Their aim is to either remove pathogenic bacteria with antibiotic supplementation or, by using prebiotics and probiotics, to enable the beneficial populations in the cecum to flourish at the expense of those that are possibly harmful [4]. Limited studies have been carried out to investigate the effect of exogenous lysozyme on poultry intestinal microbiota. In this study, we used 16S rRNA and ITS fragment high-throughput sequencing combined with transcriptomic analysis to provide a comprehensive view to provide insights into this issue. Our results indicate that dietary supplementation with increasing levels (40, 100, and 200 ppm) of lysozyme did not significantly (*P* > 0.05) affect the composition and diversity of the cecal bacterial and fungal communities, although there were substantial changes in some phylum (Fig 2), genus (Fig 3), OTU (S8 Table) relative abundances in the lysozyme group. Furthermore, no changes in body weight gain, feed intake, and feed conversion were detectable between control chickens and those given lysozyme-supplemented feeds (Table 1). Based on culture-dependent methodology, it has been reported that 90–100 ppm lysozyme feed supplementation reduced fecal *E*. *coli* counts in broilers [11] and weaned pigs [10] and cecal *E*. *coli* counts in broiler chickens [9], while increasing those of the beneficial *Lactobacillus* sp. However, no explanation was provided to explain why this might have occurred. The one-way ANOVA analyses here failed to show any significant differences in the relative abundances of the cecal members of all genera, including *Escherichia*, *Bifidobacterium*, and *Lactobacillus*, between chickens fed diets with and without lysozyme supplementation.

Supplementation with lysozyme appeared to impact enzyme expression and metabolism of the cecal microbiota in the chickens. Both GO and KEGG enrichment analyses showed that lysozyme supplementation significantly enriched genes for “carbohydrate metabolic process” (GO) and “carbon metabolism” (KEGG) of the cecal microbiota, including “starch and sucrose metabolism,” “monosaccharide biosynthesis process,” “monosaccharide catabolic process,” “glycolysis,” and “pyruvate metabolic process” (Tables 3& 4). These data imply that lysozyme could potentially enhance the capability of the chicken cecal microbiota to use NSPs in the digesta entering the cecum more efficiently than those in chickens not exposed to it. Their anaerobic degradation would generate mainly SCFAs as discussed earlier (e.g., [26,32]). The SCFAs formed are beneficial to the host in at least two ways: (1) SCFAs may enter the blood stream, thus contributing partly (3–5%) to the energy requirements of the chickens [32,33], and (2) SCFAs decrease the pH of the intestinal environment, thus inhibiting potential pathogens, decreasing the solubility of bile acids, increasing indirectly the absorption of minerals, and reducing ammonia absorption by the protonic dissociation of ammonia and other amines (see the review by Wong et al. [34]). Therefore, lysozyme supplementation may play an important role in maintaining intestinal function and health by promoting the populations that produce SCFAs. We analyzed the CAEs that catalyze the breakdown and/or modification of glycoconjugates and oligo- and polysaccharides in the cecal samples based on our metatranscriptomic dataset. GH catalyze the hydrolysis of glycosidic bonds in complex sugars, including cellulose (cellulase), hemicellulose, and starch (amylase); PL cleave certain activated glycosidic linkages present in acidic polysaccharides; CE catalyze the O-de- or N-deacylation of substituted saccharides; AA catalyze lignin degradation; and GT catalyze the transfer of saccharide moieties from the glycosyl donor to a glycosyl acceptor molecule and CBM (carbohydrate-binding modules) (http://www.cazy.org/). In this study, GH catalyzed the hydrolysis of NSPs present in the feed, while CE, PL, and AA catalyzed the breakdown of the cell walls of plant cells. GT and CBM assisted in the degradation of polysaccharides. We found that GH genes were the most abundant in each cecal sample, contributing on average 48.2% of the total genes coded for CAEs, while CE, AA, and PL only accounted for 9.0, 1.9, and 1.3% of them, respectively.

We showed that *Bacteroides* contributed 31.9% of GH (S2 Table), 16.2% of GT (S3 Table), 26.1% of PL (S4 Table), 20.7% of CE (S5 Table), 13.9% of CBM (S6 Table) and 8.8% of AA (S7 Table) genes identified in the cecal samples, thus representing the main players in the breakdown of NSPs, although *Parabacteroides*, *Alistipes*, *Prevotella*, *Clostridium*, *Blastocystis*, *Barnesiella*, *Blautia*, *Faecalibacterium*, *Subdoligranulum*, *Megamonas*, *Eubacterium*, *Ruminococcus*, *Paenibacillus*, *Bifidobacterium*, *Akkermansia*, and other bacteria also participated in the process. Interestingly, most of these bacteria also possess CBM (S6 Table) capable of binding to their substrates, thus ensuring the efficient breakdown of NSPs. Mancabelli et al. [27] carried out a metagenomic analysis of the cecal microbiota of broilers raised in large-scale commercial production and in semi-wild conditions. They found that the cecal microbiota of birds reared in semi-wild conditions contained more and a higher diversity of GH genes than those of birds raised in commercial production conditions. Sergent et al. [28] performed a metagenomic analysis of the cecal microbiota of broilers fed a wheat-based diet with 5% maize and identified 9,033 GH genes. Although no phylogenetic information was provided for these GH genes and it was not determined whether these GH genes were expressed in vivo, they found evidence of over 500 polysaccharide utilization systems [35], which digest and import the products of polysaccharide degradation, in *Bacteroides* from the chicken cecum. Our metatranscriptomic and their metagenomic results complement each other to provide evidence for an important role of *Bacteroides* in the degradation of NSPs in the chicken cecum. We also noticed that a significant fraction of GT (47.3%), PL (27.8%), CE (17.7%), CBM (41.1%), and AA (50.0%) genes could not be classified, further emphasizing the complexity of the composition and diversity of the cecal microbiota of broiler chickens.

The mechanism/s whereby lysozyme affects gene expression remain unknown. Lysozyme has never been shown to play any role in regulating gene expression, and so it is more likely that the changes seen here might involve the products of its activity. As reviewed by Lukasiewicz and Lugowski [36] the action of lysozyme seems depend more on its muramidase-independent activities than its muramidase-dependent bactericidal activities. Lysozyme may act on both Gram-negative and Gram-positive bacteria by impairing DNA and RNA synthesis, activating autolysin production [37–40], and permeabilizing cell walls and membranes, thus resulting in their depolarization, and finally cytosol leakage [37, 38, 41]. This helps to explain the overexpression of genes associated with bacterial outer membranes and cell walls with lysozyme (LYS200) found in our study, as a response to permeabilization of cell wall and membrane. Therefore, a possible explanation is that the overexpression of genes associated with bacterial outer membranes and cell walls, cross-cell substrate transport, and carbohydrate metabolic processes induced by supplementation with lysozyme (LYS200), as revealed by the GO enrichment analysis, could imply that lysozyme permeabilizes their cell walls and thus increases their permeability and allows the more rapid uptake of exogenous substrates, leading to an increased rate of energy production by the cecal microbiota.

Although lysozyme supplementation promoted cecal carbohydrate metabolic processes in the birds, it did not significantly (*P* > 0.05) improve their growth performance in terms of body weight gain, feed intake, and feed conversion. This may reflect that the cecal microbiota contribute only a minor fraction (3–7%) of the total energy requirement of chickens. Any increase in energy contribution from the cecal microbiota following supplementation with lysozyme could be easily diluted by the differences in BD gain resulting from differences in the physiology of the individual birds in each treatment group. Thus, the mean BW gains of the birds given different lysozyme dosages were generally higher but not significantly higher than those of the controls. The metabolic data obtained in this study may also help to explain the conflicting results concerning the effect of lysozyme exposure on growth performance in chickens and pigs [9]. Abdel-Latif et al. [11] did report that dietary addition of 90 ppm lysozyme improved growth performance of Ross 308 chicks fed a corn-based diet over 35 days. Except for differences in the breeds of chickens used, no substantial differences in housing environments and diets used were found between their and our experiments, thus indicating that chicken breed may be an important determinant in how lysozyme affects growth performance.

Flavomycin has been widely used as an antimicrobial growth promoter in the pig and poultry industries to increase production since its discovery in the mid-1950s [42]. It inhibits peptidoglycan synthesis by inhibiting peptidoglycan polymerases of gram-positive and gram-negative bacteria by impairing the transglycosylase activities of penicillin-binding proteins, resulting in the specific blocking of the biosynthesis or insertion into the expanding wall of growing peptidoglycan chains (reviewed by Butaye et al. [43]). In this study, we used flavomycin as an antibiotic control, and as with lysozyme, it did not significantly affect the diversity or composition of bacterial communities in the cecal microbiota. This is generally in line with other studies [44] that reported that treatment of chickens with AGPs did not alter intestinal bacterial communities, although individual taxa may have changed. In this study, GO enrichment analyses revealed that supplementation with lysozyme (LYS200) and with flavomycin enriched genes for “EESP,” “oxidation-reduction process,” and “carbohydrate metabolic process” but that only lysozyme enriched those involved in “transport.” Similarly, KEGG analyses revealed that lysozyme supplementation at the three dosages enriched genes involved in “carbon metabolism,” “starch and sucrose metabolism,” and “glycolysis/gluconeogenesis” but that flavomycin did not. Although it was not the aim of this study, these results indicate that the effects of flavomycin on the metabolism of the cecal microbiota varied from those of lysozyme.

## Supporting information

S1 Table Chemical composition of the broiler diet. (PDF)

S2 Table Taxonomy of glycoside hydrolase (GH) genes identified in the cecal microbiota of broilers fed a corn-based diet supplemented with 0 (R1 in gene query name), 40 (R7 in gene query name), 100 (R8 in gene query name), or 200 ppm (R9 in gene query name) lysozyme or 400 ppm flavomycin (R3 in gene query name) [the gene names in query refer to those in transcriptome dataset deposited as PRJNA523864 in NCBI Sequence Read Archive]. (PDF)

S3 Table Taxonomy of glycosyltransferase (GT) genes identified in the cecal microbiota of broilers fed a corn-based diet supplemented with 0 (R1 in gene query name), 40 (R7 in gene query name), 100 (R8 in gene query name), or 200 ppm (R9 in gene query name) lysozyme or 400 ppm flavomycin (R3 in gene query name) [the gene names in query refer to those in transcriptome dataset deposited as PRJNA523864 in NCBI Sequence Read Archive]. (PDF)

S4 Table Taxonomy of polysaccharide lyases (PL) genes identified in the cecal microbiota of broilers fed a corn-based diet supplemented with 0 (R1 in gene query name), 40 (R7 in gene query name), 100 (R8 in gene query name), or 200 ppm (R9 in gene query name) lysozyme or 400 ppm flavomycin (R3 in gene query name) [the gene names in query refer to those in transcriptome dataset deposited as PRJNA523864 in NCBI Sequence Read Archive]. (PDF)

S5 Table Taxonomy of carbohydrate esterase (CE) genes identified in the cecal microbiota of broilers fed a corn-based diet supplemented with 0 (R1 in gene query name), 40 (R7 in gene query name), 100 (R8 in gene query name), or 200 ppm (R9 in gene query name) lysozyme or 400 ppm flavomycin (R3 in gene query name) [the gene names in query refer to the those in the transcriptome dataset deposited as PRJNA523864 in NCBI Sequence Read Archive]. (PDF)

S6 Table Taxonomy of carbohydrate-binding modules (CBM) genes identified in the cecal microbiota of broilers fed a corn-based diet supplemented with 0 (R1 in gene query name), 40 (R7 in gene query name), 100 (R8 in gene query name), or 200 ppm (R9 in gene query name) lysozyme or 400 ppm flavomycin (R3 in gene query name) [the gene names in query refer to those in the transcriptome dataset deposited as PRJNA523864 in NCBI Sequence Read Archive]. (PDF)

S7 Table Taxonomy of auxiliary activities (AA) genes identified in the cecal microbiota of broilers fed a corn-based diet supplemented with 0 (R1 in gene query), 40 (R7 in gene query name), 100 (R8 in gene query name), or 200 ppm (R9 in gene query name) lysozyme or 400 ppm flavomycin (R3 in gene query name) [the gene names in query refer to those in the transcriptome dataset deposited as PRJNA523864 in NCBI Sequence Read Archive]. (PDF)

S8 Table Composition of dominant bacterial species (with average percentage abundance > 0.5% in all caecal samples) and their distribution in the ceca of broiler chickens fed a corn-based diet supplemented with 40, 100 and 200 ppm lysozyme or 400 ppm flavomycin. (PDF)

S1 Fig Volcano plots showing the genes in the cecal microbiota that were up- and down-regulated by dietary supplementation with 40 (LYS40), 100 (LYS100), or 200 ppm (LYS200) lysozyme or 400 ppm flavomycin (fdr in the figure represents false detection rate). (PDF)
