# Supplementary material for: Effects of dietary supplementation with lysozyme on the structure and function of the cecal microbiota in broiler chickens
Source: PLoS One. 2019 Jun 19;14(6):e0216748. doi: 10.1371/journal.pone.0216748 (PMC6583987; doi:10.1371/journal.pone.0216748)
Supplement: S1 Table — (PDF) [file pone.0216748.s001.pdf]

S1 Table. Chemical composition of broiler diet.

Ingredient	Starter	Grower
Corn (%)	58.00	61.80
Soybean meal (%)	27.00	23.7
Corn gluten meal (%)	5.00	6.90
Fish meal (%)	2.80	0.00
Soybean oil (%)	3.00	3.20
Calcium hydrogen phosphate (%)	1.45	1.68
Fine stone powder (%)	1.15	0.76
Coarse stone powder (%)	0.00	0.40
Salt (%)	0.23	0.33
Methionine (%)	0.17	0.06
Lysine (%)	0.16	0.17
Vitamin premix* (%)	1.00	1.00
Total	100	100
**Analyzed and calculated composition**	**Starter**	**Grower**
Metablizable energy (KC/Kg)	3050	3100
Crude protein (%)	23.6	22.6
Crude fibre (%)	2.70	22.6
Crude fat (%)	4.82	4.49
Dry matter (%)	89.2	89.9
Lysine (%)	1.14	1.17
Methionine (%)	0.33	0.33
Calcium (%)	1.0	1.06
Available P (%)	0.45	0.40
NaCl (%)	0.35	0.35

*2.5 kg of vitamin premix contains: 10.8 g retinal, 1.6 g calcidiol, 72 g tocopheryl acetate, 8 g menadione, 7.2 g thiamine, 26.4 g riboflavin, 40 g niacin, 120 g calcium pantothenate, 12 g pyridoxine, 4 g folic acid, 0.06 g cyanocobalamin, 1000 g choline chloride, 0.4 g biotin.
