# Supplementary material for: Effects of dietary supplementation with lysozyme on the structure and function of the cecal microbiota in broiler chickens
Source: PLoS One. 2019 Jun 19;14(6):e0216748. doi: 10.1371/journal.pone.0216748 (PMC6583987; doi:10.1371/journal.pone.0216748)
Supplement: S2 Table — (PDF) [file pone.0216748.s002.pdf]

S2 Table. Taxonomy of glycoside hydrolase (GH) genes identified in the cecal microbiota of broilers fed a corn-based diet supplemented with 0 (R1 in gene query name), 40 (R7 in gene query name), 100 (R8 in gene query name), or 200 ppm (R9 in gene query name) lysozyme or 400 ppm flavomycin (R3 in gene query name) [the gene names in query refer to those in transcriptome dataset deposited as PRJNA523864 in NCBI Sequence Read Archive].

No.	Gene query	Family	Taxonomy
1	comp27558_c0_seq1.26.2039.minus.R1_1	GH29	*Acidobacteriaceae_bacterium*_KBS_96
2	comp93205_c0_seq1.1.765.minus.R8_1	GH28	*Saccharomonospora_cyanea*
3	comp124929_c0_seq1.1.1122.minus.R1_1	GH33	*Atopobium_fossor*
4	comp155452_c0_seq1.1.737.minus.R7_1	GH20	*Armatimonadetes*_bacterium_CSP1-3
5	comp111141_c0_seq1.17.784.plus.R1_1	GH92	*Bacteroides_acidifaciens*
6	comp46635_c0_seq4.141.1102.minus.R8_1	GH130	*Bacteroides_acidifaciens*
7	comp10119_c0_seq1.22.2123.minus.R3_1	GH29	*Bacteroides_barnesiae*
8	comp125732_c0_seq1.1.906.minus.R3_1	GH31	*Bacteroides_barnesiae*
9	comp165066_c0_seq1.1.841.minus.R1_1	GH97	*Bacteroides_barnesiae*
10	comp187760_c0_seq1.1.811.minus.R1_1	GH31	*Bacteroides_barnesiae*
11	comp19450_c0_seq1.1.1253.minus.R9_1	GH89	*Bacteroides_barnesiae*
12	comp21486_c0_seq1.1.857.minus.R3_1	GH23	*Bacteroides_barnesiae*
13	comp23337_c0_seq1.100.1380.plus.R9_1	GH29	*Bacteroides_barnesiae*
14	comp23514_c0_seq1.75.2777.minus.R9_1	GH77	*Bacteroides_barnesiae*
15	comp25130_c0_seq1.653.1990.plus.R9_1	GH23	*Bacteroides_barnesiae*
16	comp25408_c0_seq1.669.1584.minus.R8_1	GH13	*Bacteroides_barnesiae*
17	comp26697_c0_seq1.7.1974.plus.R3_1	GH20	*Bacteroides_barnesiae*
18	comp28554_c0_seq1.38.1624.plus.R9_1	GH33	*Bacteroides_barnesiae*
19	comp30363_c0_seq2.97.2328.minus.R3_1	GH92	*Bacteroides_barnesiae*
20	comp31285_c0_seq8.135.1718.plus.R3_1	GH109	*Bacteroides_barnesiae*
21	comp36057_c0_seq18.3316.4689.plus.R3_1	GH57	*Bacteroides_barnesiae*
22	comp36057_c0_seq7.67.2010.plus.R3_1	GH133	*Bacteroides_barnesiae*
23	comp36339_c0_seq5.781.2661.minus.R3_1	GH13	*Bacteroides_barnesiae*
24	comp36462_c0_seq2.50.1408.plus.R3_1	GH20	*Bacteroides_barnesiae*
25	comp37496_c0_seq1.48.2396.minus.R9_1	GH20	*Bacteroides_barnesiae*
26	comp38623_c0_seq1.50.814.plus.R9_1	GH23	*Bacteroides_barnesiae*
27	comp39212_c0_seq11.39.1068.minus.R9_1	GH4	*Bacteroides_barnesiae*
28	comp39212_c0_seq6.39.1040.minus.R9_1	GH4	*Bacteroides_barnesiae*
29	comp39834_c0_seq1.28.1552.minus.R9_1	GH20	*Bacteroides_barnesiae*
30	comp43029_c0_seq1.77.1003.plus.R1_1	GH97	*Bacteroides_barnesiae*
31	comp47873_c0_seq1.1.897.minus.R1_1	GH13	*Bacteroides_barnesiae*
32	comp48717_c0_seq2.277.2609.minus.R8_1	GH20	*Bacteroides_barnesiae*
33	comp48717_c0_seq7.277.2622.minus.R8_1	GH20	*Bacteroides_barnesiae*
34	comp49288_c0_seq1.190.2221.minus.R7_1	GH92	*Bacteroides_barnesiae*
35	comp49416_c0_seq1.166.2045.minus.R7_1	GH32	*Bacteroides_barnesiae*
36	comp51887_c0_seq1.157.2447.minus.R3_1	GH92	*Bacteroides_barnesiae*
37	comp51995_c0_seq1.1.969.minus.R7_1	GH97	*Bacteroides_barnesiae*
38	comp54142_c0_seq1.1200.2897.plus.R1_1	GH13	*Bacteroides_barnesiae*
39	comp55598_c0_seq1.542.1543.minus.R7_1	GH4	*Bacteroides_barnesiae*
40	comp55774_c1_seq16.98.2662.plus.R7_1	GH2	*Bacteroides_barnesiae*
41	comp57050_c0_seq1.1126.2510.minus.R3_1	GH29	*Bacteroides_barnesiae*
42	comp60761_c0_seq1.1.1950.minus.R3_1	GH92	*Bacteroides_barnesiae*
43	comp61821_c0_seq2.14.1957.minus.R1_1	GH133	*Bacteroides_barnesiae*

44	comp62853_c0_seq1.17.2482.plus.R9_1	GH95	*Bacteroides_barnesiae*
45	comp67019_c0_seq1.381.1218.minus.R9_1	GH51	*Bacteroides_barnesiae*
46	comp68960_c0_seq1.1.861.minus.R9_1	GH29	*Bacteroides_barnesiae*
47	comp90435_c0_seq1.3.1175.plus.R1_1	GH29	*Bacteroides_barnesiae*
48	comp9798_c0_seq1.94.1935.plus.R3_1	GH20	*Bacteroides_barnesiae*
49	comp60311_c0_seq9.1.3331.minus.R1_1	GH2	*Bacteroides_barnesiae*
50	comp60311_c0_seq4.1130.3561.minus.R1_1	GH2	*Bacteroides_caccae*
51	comp94433_c0_seq1.302.1312.minus.R1_1	GH109	*Bacteroides_caccae*
52	comp58149_c0_seq1.1.1156.minus.R9_1	GH36	*Bacteroides_cellulosilyticus*
53	comp249742_c0_seq1.1.753.minus.R1_1	GH97	*Bacteroides_cellulosilyticus*_CAG
54	comp35288_c0_seq1.1.1056.minus.R8_1	GH31	*Bacteroides_cellulosilyticus*_CAG
55	comp25967_c0_seq2.17.1235.minus.R8_1	GH88	*Bacteroides_clarus*
56	comp15826_c0_seq1.1.748.minus.R8_1	GH97	*Bacteroides_clarus*_CAG
57	comp10236_c0_seq1.25.2742.minus.R3_1	GH77	*Bacteroides_coprocola*
58	comp30363_c0_seq1.97.2328.minus.R3_1	GH92	*Bacteroides_coprocola*
59	comp31956_c0_seq2.5441.6538.plus.R3_1	GH18	*Bacteroides_coprocola*
60	comp33857_c0_seq1.6079.8076.plus.R3_1	GH13	*Bacteroides_coprocola*
61	comp35877_c1_seq3.41.1042.plus.R3_1	GH4	*Bacteroides_coprocola*
62	comp47868_c0_seq9.369.2648.plus.R8_1	GH92	*Bacteroides_coprocola*
63	comp48717_c0_seq10.1.950.minus.R8_1	GH20	*Bacteroides_coprocola*
64	comp51208_c0_seq1.31.2026.minus.R1_1	GH92	*Bacteroides_coprocola*
65	comp53999_c0_seq1.4841.5995.plus.R1_1	GH18	*Bacteroides_coprocola*
66	comp54146_c0_seq7.57.966.minus.R7_1	GH92	*Bacteroides_coprocola*
67	comp57639_c0_seq3.905.3244.minus.R1_1	GH20	*Bacteroides_coprocola*
68	comp60769_c0_seq3.68.2350.minus.R1_1	GH92	*Bacteroides_coprocola*
69	comp60769_c0_seq5.68.920.minus.R1_1	GH92	*Bacteroides_coprocola*
70	comp61014_c0_seq4.448.2784.plus.R1_1	GH20	*Bacteroides_coprocola*
71	comp61014_c0_seq8.79.2346.plus.R1_1	GH20	*Bacteroides_coprocola*
72	comp61019_c0_seq1.1539.2510.plus.R1_1	GH130	*Bacteroides_coprocola*
73	comp61019_c0_seq1.2630.4861.plus.R1_1	GH92	*Bacteroides_coprocola*
74	comp64562_c0_seq1.409.2589.plus.R1_1	GH97	*Bacteroides_coprocola*
75	comp68103_c0_seq1.1.1227.minus.R1_1	GH97	*Bacteroides_coprocola*
76	comp70234_c0_seq1.16.1467.minus.R9_1	GH2	*Bacteroides_coprocola*
77	comp7027_c0_seq1.1.806.minus.R3_1	GH13	*Bacteroides_coprocola*
78	comp99717_c0_seq1.49.1300.minus.R1_1	GH105	*Bacteroides_coprocola*
79	comp127710_c0_seq1.1.1697.minus.R1_1	GH38	*Bacteroides_coprocola*_CAG
80	comp146595_c0_seq1.101.1562.minus.R1_1	GH28	*Bacteroides_coprocola*_CAG
81	comp27736_c0_seq1.40.1263.plus.R8_1	GH36	*Bacteroides_coprocola*_CAG
82	comp31009_c0_seq1.1.1289.minus.R1_1	GH2	*Bacteroides_coprocola*_CAG
83	comp35955_c0_seq1.2146.3753.minus.R3_1	GH28	*Bacteroides_coprocola*_CAG
84	comp39464_c0_seq1.1.1247.minus.R7_1	GH43	*Bacteroides_coprocola*_CAG
85	comp44714_c0_seq1.1.817.minus.R7_1	GH2	*Bacteroides_coprocola*_CAG
86	comp46881_c0_seq2.1.1784.minus.R1_1	GH3	*Bacteroides_coprocola*_CAG
87	comp47868_c0_seq12.16.2358.minus.R8_1	GH92	*Bacteroides_coprocola*_CAG
88	comp48373_c0_seq3.774.1757.plus.R1_1	GH23	*Bacteroides_coprocola*_CAG
89	comp48386_c0_seq3.609.2830.minus.R1_1	GH92	*Bacteroides_coprocola*_CAG
90	comp48692_c0_seq1.127.864.plus.R1_1	GH43	*Bacteroides_coprocola*_CAG
91	comp48717_c0_seq5.1.1028.minus.R8_1	GH20	*Bacteroides_coprocola*_CAG
92	comp49229_c0_seq3.1.942.minus.R1_1	GH3	*Bacteroides_coprocola*_CAG
93	comp49229_c0_seq4.1.939.minus.R1_1	GH3	*Bacteroides_coprocola*_CAG

94	comp53999_c0_seq3.2545.4602.plus.R1_1	GH20	*Bacteroides_coprocola*_CAG
95	comp61821_c0_seq7.43.1416.minus.R1_1	GH57	*Bacteroides_coprocola*_CAG
96	comp63824_c0_seq1.7616.9961.plus.R1_1	GH3	*Bacteroides_coprocola*_CAG
97	comp70504_c0_seq1.15.1160.minus.R7_1	GH10	*Bacteroides_coprocola*_CAG
98	comp73663_c0_seq1.1592.3808.minus.R1_1	GH92	*Bacteroides_coprocola*_CAG
99	comp77739_c0_seq1.1243.3616.minus.R1_1	GH3	*Bacteroides_coprocola*_CAG
100	comp94645_c0_seq1.492.1613.plus.R1_1	GH76	*Bacteroides_coprocola*_CAG
101	comp48373_c0_seq1.731.3841.plus.R1_1	GH2	*Bacteroides_coprocola*_CAG
102	comp108820_c0_seq1.115.1251.plus.R8_1	GH109	*Bacteroides_coprophilus*
103	comp114911_c0_seq1.1.822.minus.R1_1	GH3	*Bacteroides_coprophilus*
104	comp11730_c0_seq1.2330.3667.plus.R7_1	GH29	*Bacteroides_coprophilus*
105	comp129693_c0_seq1.1.941.minus.R7_1	GH2	*Bacteroides_coprophilus*
106	comp131698_c0_seq1.1.857.minus.R1_1	GH16	*Bacteroides_coprophilus*
107	comp13258_c0_seq1.835.1655.minus.R7_1	GH16	*Bacteroides_coprophilus*
108	comp13793_c0_seq1.18.2153.plus.R8_1	GH2	*Bacteroides_coprophilus*
109	comp138418_c0_seq1.13.921.plus.R7_1	GH29	*Bacteroides_coprophilus*
110	comp22036_c0_seq1.14.940.plus.R1_1	GH109	*Bacteroides_coprophilus*
111	comp252207_c0_seq1.1.856.minus.R7_1	GH5	*Bacteroides_coprophilus*
112	comp28438_c0_seq1.30.2264.minus.R7_1	GH92	*Bacteroides_coprophilus*
113	comp31287_c0_seq2.29.2053.plus.R1_1	GH20	*Bacteroides_coprophilus*
114	comp32526_c0_seq1.42.1346.plus.R1_1	GH29	*Bacteroides_coprophilus*
115	comp35942_c0_seq1.491.1822.minus.R9_1	GH23	*Bacteroides_coprophilus*
116	comp43387_c0_seq1.2.1510.plus.R8_1	GH13	*Bacteroides_coprophilus*
117	comp43418_c0_seq1.356.2626.minus.R7_1	GH92	*Bacteroides_coprophilus*
118	comp44895_c0_seq2.536.1504.plus.R1_1	GH23	*Bacteroides_coprophilus*
119	comp47749_c0_seq2.63.1691.minus.R1_1	GH33	*Bacteroides_coprophilus*
120	comp48717_c0_seq3.6353.8920.minus.R8_1	GH2	*Bacteroides_coprophilus*
121	comp48932_c0_seq1.54.2371.minus.R1_1	GH20	*Bacteroides_coprophilus*
122	comp50437_c0_seq2.155.1531.minus.R7_1	GH57	*Bacteroides_coprophilus*
123	comp51619_c0_seq6.710.2722.minus.R7_1	GH13	*Bacteroides_coprophilus*
124	comp51996_c0_seq3.47.2392.minus.R7_1	GH20	*Bacteroides_coprophilus*
125	comp54731_c0_seq8.1079.3415.plus.R7_1	GH20	*Bacteroides_coprophilus*
126	comp55598_c0_seq6.25.1026.minus.R7_1	GH4	*Bacteroides_coprophilus*
127	comp57179_c0_seq3.16.1566.plus.R1_1	GH109	*Bacteroides_coprophilus*
128	comp60626_c0_seq1.846.3084.minus.R8_1	GH84	*Bacteroides_coprophilus*
129	comp61052_c0_seq2.2545.3801.plus.R1_1	GH23	*Bacteroides_coprophilus*
130	comp61821_c0_seq6.14.1989.minus.R1_1	GH133	*Bacteroides_coprophilus*
131	comp62257_c0_seq2.546.1922.plus.R1_1	GH57	*Bacteroides_coprophilus*
132	comp64747_c0_seq1.195.1144.minus.R8_1	GH18	*Bacteroides_coprophilus*
133	comp65926_c0_seq1.208.1101.plus.R1_1	GH25	*Bacteroides_coprophilus*
134	comp66882_c0_seq1.49.1410.plus.R8_1	GH29	*Bacteroides_coprophilus*
135	comp67374_c0_seq1.56.1856.minus.R8_1	GH2	*Bacteroides_coprophilus*
136	comp83850_c0_seq1.133.2292.plus.R1_1	GH29	*Bacteroides_coprophilus*
137	comp87541_c0_seq1.128.1099.plus.R1_1	GH130	*Bacteroides_coprophilus*
138	comp95240_c0_seq1.9.785.plus.R1_1	GH16	*Bacteroides_coprophilus*
139	comp96594_c0_seq1.60.1145.plus.R1_1	GH95	*Bacteroides_coprophilus*
140	comp9913_c0_seq1.1.833.minus.R7_1	GH3	*Bacteroides_coprophilus*
141	comp9950_c0_seq1.65.1980.minus.R8_1	GH29	*Bacteroides_coprophilus*
142	comp118842_c0_seq1.1.1096.minus.R1_1	GH97	*Bacteroides_coprophilus*_CAG
143	comp125702_c0_seq1.1.755.minus.R7_1	GH97	*Bacteroides_coprophilus*_CAG

144	comp16435_c0_seq1.51.890.plus.R7_1	GH73	*Bacteroides_coprophilus*_CAG
145	comp18468_c0_seq1.62.2776.plus.R1_1	GH2	*Bacteroides_coprophilus*_CAG
146	comp27347_c0_seq1.1.821.minus.R8_1	GH125	*Bacteroides_coprophilus*_CAG
147	comp32616_c0_seq1.1.1150.minus.R1_1	GH2	*Bacteroides_coprophilus*_CAG
148	comp33576_c0_seq1.2049.4330.minus.R1_1	GH29	*Bacteroides_coprophilus*_CAG
149	comp44210_c0_seq3.1.1146.minus.R8_1	GH36	*Bacteroides_coprophilus*_CAG
150	comp46781_c0_seq2.99.1535.plus.R7_1	GH29	*Bacteroides_coprophilus*_CAG
151	comp49247_c0_seq1.1728.3107.minus.R7_1	GH23	*Bacteroides_coprophilus*_CAG
152	comp51801_c0_seq1.18.2053.minus.R3_1	GH20	*Bacteroides_coprophilus*_CAG
153	comp51948_c0_seq1.28.2011.minus.R7_1	GH2	*Bacteroides_coprophilus*_CAG
154	comp55144_c0_seq5.24.2537.plus.R7_1	GH2	*Bacteroides_coprophilus*_CAG
155	comp55909_c0_seq1.1.1471.minus.R1_1	GH20	*Bacteroides_coprophilus*_CAG
156	comp57647_c0_seq1.38.2170.minus.R1_1	GH2	*Bacteroides_coprophilus*_CAG
157	comp62771_c0_seq9.5903.7774.plus.R1_1	GH32	*Bacteroides_coprophilus*_CAG
158	comp62915_c0_seq1.1.840.minus.R1_1	GH2	*Bacteroides_coprophilus*_CAG
159	comp62915_c0_seq8.1.1019.minus.R1_1	GH2	*Bacteroides_coprophilus*_CAG
160	comp74036_c0_seq1.428.2617.plus.R7_1	GH77	*Bacteroides_coprophilus*_CAG
161	comp74466_c0_seq1.1.769.minus.R7_1	GH31	*Bacteroides_coprophilus*_CAG
162	comp75953_c0_seq1.1828.3927.minus.R1_1	GH29	*Bacteroides_coprophilus*_CAG
163	comp82959_c0_seq1.1.1311.minus.R7_1	GH95	*Bacteroides_coprophilus*_CAG
164	comp88103_c0_seq1.1.1089.minus.R7_1	GH2	*Bacteroides_coprophilus*_CAG
165	comp89362_c0_seq1.1.1124.minus.R1_1	GH43	*Bacteroides_coprophilus*_CAG
166	comp90408_c0_seq1.26.1102.plus.R1_1	GH43	*Bacteroides_coprophilus*_CAG
167	comp91251_c0_seq1.2.985.minus.R1_1	GH92	*Bacteroides_coprophilus*_CAG
168	comp96092_c0_seq1.1.1682.minus.R7_1	GH2	*Bacteroides_coprophilus*_CAG
169	comp49895_c0_seq8.215.3808.plus.R8_1	GH2	*Bacteroides_coprophilus*_CAG
170	comp49895_c0_seq9.575.4174.plus.R8_1	GH2	*Bacteroides_coprophilus*_CAG
171	comp60311_c0_seq6.1.3319.minus.R1_1	GH2	*Bacteroides_coprophilus*_CAG
172	comp68993_c0_seq1.75.3911.minus.R7_1	GH31	*Bacteroides_coprophilus*_CAG
173	comp20801_c0_seq1.1.772.minus.R3_1	GH127	*Bacteroides_dorei*
174	comp22962_c0_seq1.1284.2396.minus.R9_1	GH99	*Bacteroides_dorei*
175	comp102279_c0_seq1.1.962.minus.R1_1	GH43	*Bacteroides_faecichinchillae*
176	comp10907_c0_seq1.139.1653.plus.R1_1	GH27	*Bacteroides_faecichinchillae*
177	comp113580_c0_seq1.46.1554.plus.R1_1	GH32	*Bacteroides_faecichinchillae*
178	comp115599_c0_seq1.61.1716.plus.R1_1	GH92	*Bacteroides_faecichinchillae*
179	comp118575_c0_seq1.30.1154.minus.R1_1	GH92	*Bacteroides_faecichinchillae*
180	comp120378_c0_seq1.1.801.minus.R1_1	GH109	*Bacteroides_faecichinchillae*
181	comp127568_c0_seq1.1.944.minus.R1_1	GH92	*Bacteroides_faecichinchillae*
182	comp127568_c0_seq1.1.944.minus.R1_1	GH92	*Bacteroides_faecichinchillae*
183	comp142818_c0_seq1.1.703.minus.R1_1	GH43	*Bacteroides_faecichinchillae*
184	comp30174_c0_seq1.1.1168.minus.R1_1	GH92	*Bacteroides_faecichinchillae*
185	comp49071_c0_seq1.1.747.minus.R8_1	GH97	*Bacteroides_faecichinchillae*
186	comp65570_c0_seq1.353.1559.minus.R1_1	GH88	*Bacteroides_faecichinchillae*
187	comp67713_c0_seq1.23.1336.plus.R1_1	GH109	*Bacteroides_faecichinchillae*
188	comp130619_c0_seq1.179.1246.minus.R1_1	GH92	*Bacteroides_faecis*
189	comp54681_c0_seq1.1159.4758.minus.R1_1	GH38	*Bacteroides_faecis*
190	comp80696_c0_seq1.1036.2484.plus.R1_1	GH125	*Bacteroides_faecis*_CAG
191	comp93189_c0_seq1.113.1441.minus.R3_1	GH109	*Bacteroides_faecis*_CAG
192	comp22985_c0_seq1.10.1815.plus.R8_1	GH31	*Bacteroides_finegoldii*
193	comp52367_c0_seq1.13.2508.plus.R1_1	GH31	*Bacteroides_finegoldii*

194	comp23490_c0_seq1.106.1949.minus.R3_1	GH31	*Bacteroides_fluxus*
195	comp42457_c1_seq1.48.2342.minus.R8_1	GH3	*Bacteroides_fluxus*
196	comp44159_c0_seq1.236.2536.minus.R3_1	GH3	*Bacteroides_fluxus*
197	comp49356_c0_seq5.39.1988.plus.R8_1	GH133	*Bacteroides_fluxus*
198	comp111083_c0_seq1.1.836.minus.R1_1	GH13	*Bacteroides_fragilis*
199	comp15209_c0_seq1.1.739.minus.R1_1	GH20	*Bacteroides_fragilis*
200	comp16643_c0_seq1.47.874.plus.R3_1	GH97	*Bacteroides_fragilis*
201	comp21486_c0_seq2.1.755.minus.R3_1	GH23	*Bacteroides_fragilis*
202	comp51948_c0_seq4.142.2628.minus.R7_1	GH2	*Bacteroides_fragilis*
203	comp68010_c0_seq1.50.2551.minus.R1_1	GH2	*Bacteroides_fragilis*_CAG
204	comp24007_c0_seq2.29.1957.plus.R9_1	GH133	*Bacteroides_gallinarum*
205	comp196606_c0_seq1.216.985.minus.R1_1	GH43	*Bacteroides_intestinalis*
206	comp38884_c0_seq1.2897.5101.plus.R9_1	GH10	*Bacteroides_intestinalis*
207	comp41184_c0_seq3.249.1724.plus.R8_1	GH109	*Bacteroides_intestinalis*
208	comp41184_c0_seq4.249.1724.plus.R8_1	GH109	*Bacteroides_intestinalis*
209	comp45492_c0_seq1.99.2501.plus.R3_1	GH95	*Bacteroides_intestinalis*
210	comp46248_c0_seq1.1.869.minus.R8_1	GH109	*Bacteroides_intestinalis*
211	comp86469_c0_seq1.1.1034.minus.R8_1	GH43	*Bacteroides_intestinalis*
212	comp94922_c0_seq1.1.829.minus.R8_1	GH5	*Bacteroides_intestinalis*
213	comp221533_c0_seq1.1.835.minus.R1_1	GH115	*Bacteroides_intestinalis*_CAG
214	comp24646_c0_seq1.1.836.minus.R1_1	GH2	*Bacteroides_intestinalis*_CAG
215	comp103309_c0_seq1.1.902.minus.R7_1	GH2	*Bacteroides_massiliensis*
216	comp93435_c0_seq1.1.901.minus.R1_1	GH20	*Bacteroides_massiliensis*
217	comp31285_c0_seq5.46.1587.plus.R3_1	GH109	*Bacteroides_nordii*
218	comp31285_c0_seq6.46.1623.plus.R3_1	GH109	*Bacteroides_nordii*
219	comp53565_c0_seq1.39.1445.plus.R9_1	GH2	*Bacteroides_nordii*
220	comp89213_c0_seq1.76.1077.plus.R1_1	GH4	*Bacteroides_nordii*
221	comp17781_c0_seq1.35.1089.minus.R1_1	GH2	*Bacteroides_oleiciplenus*
222	comp49836_c0_seq1.445.2301.minus.R1_1	GH2	*Bacteroides_oleiciplenus*
223	comp49836_c0_seq2.445.2301.minus.R1_1	GH2	*Bacteroides_oleiciplenus*
224	comp72668_c0_seq1.35.1042.plus.R8_1	GH18	*Bacteroides_oleiciplenus*
225	comp47107_c0_seq1.456.3832.minus.R9_1	GH2	*Bacteroides_oleiciplenus*
226	comp145589_c0_seq1.193.946.minus.R1_1	GH123	*Bacteroides_ovatus*
227	comp151616_c0_seq1.1.755.minus.R1_1	GH78	*Bacteroides_ovatus*
228	comp44581_c0_seq1.1.1298.minus.R1_1	GH32	*Bacteroides_ovatus*
229	comp45031_c0_seq2.4099.5517.plus.R8_1	GH18	*Bacteroides_ovatus*
230	comp77427_c0_seq1.16.996.plus.R1_1	GH32	*Bacteroides_ovatus*
231	comp80735_c0_seq1.8.3019.minus.R7_1	GH76	*Bacteroides_ovatus*
232	comp72804_c0_seq1.1.892.minus.R8_1	GH36	*Bacteroides_paurosaccharolyticus*
233	comp115475_c0_seq1.201.1295.plus.R1_1	GH43	*Bacteroides_plebeius*
234	comp124610_c0_seq1.1.1362.minus.R1_1	GH2	*Bacteroides_plebeius*
235	comp186634_c0_seq1.1.1258.minus.R1_1	GH95	*Bacteroides_plebeius*
236	comp25771_c0_seq1.7232.9952.minus.R3_1	GH97	*Bacteroides_plebeius*
237	comp27646_c0_seq1.75.872.plus.R3_1	GH97	*Bacteroides_plebeius*
238	comp27646_c0_seq2.76.1284.plus.R3_1	GH97	*Bacteroides_plebeius*
239	comp32027_c0_seq1.11.2928.minus.R1_1	GH2	*Bacteroides_plebeius*
240	comp32027_c0_seq2.140.3030.minus.R1_1	GH2	*Bacteroides_plebeius*
241	comp34075_c0_seq1.6554.7783.plus.R3_1	GH27	*Bacteroides_plebeius*
242	comp40367_c0_seq1.11089.13350.plus.R7_1	GH10	*Bacteroides_plebeius*
243	comp41847_c0_seq1.1.1210.minus.R1_1	GH29	*Bacteroides_plebeius*

244	comp43524_c0_seq1.2158.4308.plus.R1_1	GH105	*Bacteroides_plebeius*
245	comp45907_c0_seq1.463.2364.plus.R1_1	GH51	*Bacteroides_plebeius*
246	comp47749_c0_seq1.43.1668.minus.R1_1	GH33	*Bacteroides_plebeius*
247	comp48963_c0_seq2.86.2496.minus.R1_1	GH127	*Bacteroides_plebeius*
248	comp50843_c0_seq1.1.1838.minus.R3_1	GH2	*Bacteroides_plebeius*
249	comp51619_c0_seq4.1.760.minus.R7_1	GH13	*Bacteroides_plebeius*
250	comp54146_c0_seq10.37.906.minus.R7_1	GH92	*Bacteroides_plebeius*
251	comp60311_c0_seq8.1.1528.minus.R1_1	GH2	*Bacteroides_plebeius*
252	comp62771_c0_seq5.1391.3583.plus.R1_1	GH36	*Bacteroides_plebeius*
253	comp62771_c0_seq5.8.1369.plus.R1_1	GH32	*Bacteroides_plebeius*
254	comp62771_c0_seq9.1452.3257.plus.R1_1	GH32	*Bacteroides_plebeius*
255	comp72000_c0_seq1.1.1317.minus.R3_1	GH43	*Bacteroides_plebeius*
256	comp72785_c0_seq1.1.752.minus.R3_1	GH29	*Bacteroides_plebeius*
257	comp9201_c0_seq1.1948.3726.plus.R3_1	GH110	*Bacteroides_plebeius*
258	comp59899_c0_seq1.86.2980.plus.R1_1	GH2	*Bacteroides_plebeius*
259	comp64212_c0_seq1.6354.9017.plus.R1_1	GH2	*Bacteroides_plebeius*
260	comp101262_c0_seq1.1.1611.minus.R1_1	GH92	*Bacteroides_plebeius*_CAG
261	comp101318_c0_seq1.92.1861.plus.R1_1	GH92	*Bacteroides_plebeius*_CAG
262	comp19210_c0_seq1.1.772.minus.R3_1	GH92	*Bacteroides_plebeius*_CAG
263	comp29416_c0_seq1.1023.1991.plus.R3_1	GH23	*Bacteroides_plebeius*_CAG
264	comp30122_c0_seq2.70.1752.plus.R3_1	GH29	*Bacteroides_plebeius*_CAG
265	comp31765_c0_seq2.1.728.minus.R1_1	GH28	*Bacteroides_plebeius*_CAG
266	comp31856_c0_seq2.1.2145.minus.R1_1	GH127	*Bacteroides_plebeius*_CAG
267	comp32793_c0_seq1.276.2168.plus.R3_1	GH89	*Bacteroides_plebeius*_CAG
268	comp34592_c0_seq1.214.1626.minus.R3_1	GH29	*Bacteroides_plebeius*_CAG
269	comp36092_c0_seq2.6510.7607.plus.R3_1	GH53	*Bacteroides_plebeius*_CAG
270	comp36221_c0_seq1.665.2450.minus.R3_1	GH95	*Bacteroides_plebeius*_CAG
271	comp37642_c0_seq2.1.1099.minus.R1_1	GH97	*Bacteroides_plebeius*_CAG
272	comp38905_c0_seq1.14.763.plus.R3_1	GH53	*Bacteroides_plebeius*_CAG
273	comp38905_c0_seq1.3345.4358.plus.R3_1	GH53	*Bacteroides_plebeius*_CAG
274	comp38905_c0_seq1.846.3338.plus.R3_1	GH2	*Bacteroides_plebeius*_CAG
275	comp43184_c0_seq1.201.949.minus.R3_1	GH25	*Bacteroides_plebeius*_CAG
276	comp44608_c0_seq1.94.2684.minus.R1_1	GH84	*Bacteroides_plebeius*_CAG
277	comp46891_c0_seq1.1.2134.minus.R3_1	GH35	*Bacteroides_plebeius*_CAG
278	comp54901_c0_seq1.11.961.plus.R3_1	GH77	*Bacteroides_plebeius*_CAG
279	comp61052_c0_seq1.394.1659.plus.R1_1	GH23	*Bacteroides_plebeius*_CAG
280	comp62666_c0_seq13.3256.4902.plus.R1_1	GH20	*Bacteroides_plebeius*_CAG
281	comp62666_c0_seq7.65.2062.plus.R1_1	GH20	*Bacteroides_plebeius*_CAG
282	comp63658_c0_seq1.16.903.plus.R3_1	GH109	*Bacteroides_plebeius*_CAG
283	comp68454_c0_seq1.1.1033.minus.R8_1	GH43	*Bacteroides_plebeius*_CAG
284	comp75768_c0_seq1.8.1978.plus.R8_1	GH97	*Bacteroides_plebeius*_CAG
285	comp86286_c0_seq1.2.1108.plus.R1_1	GH105	*Bacteroides_plebeius*_CAG
286	comp62666_c0_seq7.2076.5657.plus.R1_1	GH2	*Bacteroides_plebeius*_CAG
287	comp51502_c0_seq1.12412.13518.plus.R1_1	GH16	*Bacteroides_pyogenes*
288	comp39308_c0_seq1.303.1500.minus.R9_1	GH109	*Bacteroides_reticulotermitis*
289	comp45166_c0_seq1.1.732.minus.R1_1	GH105	*Bacteroides_reticulotermitis*
290	comp100589_c0_seq1.1.885.minus.R9_1	GH51	*Bacteroides_salanitronis*
291	comp111963_c0_seq1.1.1464.minus.R8_1	GH33	*Bacteroides_salanitronis*
292	comp119218_c0_seq1.1.798.minus.R7_1	GH13	*Bacteroides_salanitronis*
293	comp135862_c0_seq1.1.816.minus.R7_1	GH92	*Bacteroides_salanitronis*

294	comp30036_c0_seq1.264.2372.plus.R3_1	GH97	*Bacteroides_salanitronis*
295	comp30640_c0_seq1.44.1114.plus.R8_1	GH97	*Bacteroides_salanitronis*
296	comp31535_c0_seq1.1.1185.minus.R1_1	GH97	*Bacteroides_salanitronis*
297	comp31729_c0_seq1.128.2818.minus.R1_1	GH77	*Bacteroides_salanitronis*
298	comp31765_c0_seq1.523.1659.minus.R1_1	GH28	*Bacteroides_salanitronis*
299	comp32052_c0_seq1.1.702.minus.R7_1	GH51	*Bacteroides_salanitronis*
300	comp32763_c0_seq1.2471.4829.minus.R1_1	GH3	*Bacteroides_salanitronis*
301	comp33616_c0_seq1.137.2451.minus.R3_1	GH95	*Bacteroides_salanitronis*
302	comp33929_c0_seq1.61.2253.plus.R3_1	GH95	*Bacteroides_salanitronis*
303	comp34004_c0_seq1.1.828.minus.R3_1	GH13	*Bacteroides_salanitronis*
304	comp35749_c0_seq1.266.2593.minus.R9_1	GH3	*Bacteroides_salanitronis*
305	comp36339_c0_seq2.1217.3403.minus.R3_1	GH97	*Bacteroides_salanitronis*
306	comp36339_c0_seq4.359.2542.minus.R3_1	GH97	*Bacteroides_salanitronis*
307	comp36339_c0_seq6.1.1020.minus.R3_1	GH97	*Bacteroides_salanitronis*
308	comp40148_c0_seq7.26.2122.plus.R9_1	GH97	*Bacteroides_salanitronis*
309	comp42840_c0_seq1.8694.10073.plus.R8_1	GH26	*Bacteroides_salanitronis*
310	comp46128_c0_seq1.1.1757.minus.R1_1	GH2	*Bacteroides_salanitronis*
311	comp46157_c0_seq1.2455.4621.minus.R8_1	GH3	*Bacteroides_salanitronis*
312	comp47303_c0_seq1.285.2594.minus.R7_1	GH92	*Bacteroides_salanitronis*
313	comp47868_c0_seq13.3.1016.plus.R8_1	GH92	*Bacteroides_salanitronis*
314	comp51511_c0_seq1.1.993.minus.R7_1	GH13	*Bacteroides_salanitronis*
315	comp54052_c0_seq1.1278.3584.plus.R1_1	GH92	*Bacteroides_salanitronis*
316	comp54146_c0_seq4.1.1205.minus.R7_1	GH92	*Bacteroides_salanitronis*
317	comp54146_c0_seq8.1243.3516.minus.R7_1	GH92	*Bacteroides_salanitronis*
318	comp54313_c0_seq1.85.2271.minus.R1_1	GH89	*Bacteroides_salanitronis*
319	comp56842_c0_seq3.1.1709.minus.R1_1	GH2	*Bacteroides_salanitronis*
320	comp58105_c0_seq1.10123.11289.plus.R1_1	GH130	*Bacteroides_salanitronis*
321	comp58105_c0_seq1.12915.14105.plus.R1_1	GH76	*Bacteroides_salanitronis*
322	comp58105_c0_seq1.8886.9989.plus.R1_1	GH26	*Bacteroides_salanitronis*
323	comp61174_c0_seq1.1.1400.minus.R8_1	GH77	*Bacteroides_salanitronis*
324	comp61732_c0_seq19.3143.4825.plus.R1_1	GH43	*Bacteroides_salanitronis*
325	comp62771_c0_seq1.2408.4276.plus.R1_1	GH32	*Bacteroides_salanitronis*
326	comp62847_c0_seq2.2074.3120.plus.R1_1	GH43	*Bacteroides_salanitronis*
327	comp65545_c0_seq1.18.2243.minus.R1_1	GH36	*Bacteroides_salanitronis*
328	comp74659_c0_seq1.1.806.minus.R3_1	GH127	*Bacteroides_salanitronis*
329	comp75050_c0_seq1.1.1586.minus.R3_1	GH105	*Bacteroides_salanitronis*
330	comp80736_c0_seq1.46.2316.plus.R1_1	GH92	*Bacteroides_salanitronis*
331	comp86135_c0_seq1.22.1584.plus.R1_1	GH43	*Bacteroides_salanitronis*
332	comp95779_c0_seq1.1.987.minus.R8_1	GH3	*Bacteroides_salanitronis*
333	comp97522_c0_seq1.1.1045.minus.R8_1	GH92	*Bacteroides_salanitronis*
334	comp56853_c0_seq1.1.3993.minus.R1_1	GH33	*Bacteroides_salyersiae*
335	comp56853_c0_seq2.41.5296.minus.R1_1	GH33	*Bacteroides_salyersiae*
336	comp30808_c0_seq1.26.1981.minus.R1_1	GH97	*Bacteroides*_sp._1_1_6
337	comp40365_c0_seq5.6.2027.plus.R9_1	GH13	*Bacteroides*_sp._2_1_33B
338	comp61019_c0_seq2.73.2247.plus.R1_1	GH20	*Bacteroides*_sp._2_1_33B
339	comp89523_c0_seq1.1.1098.minus.R9_1	GH78	*Bacteroides*_sp._2_1_33B
340	comp34932_c0_seq1.7.3165.plus.R9_1	GH2	*Bacteroides*_sp._2_1_33B
341	comp49833_c0_seq3.7152.9065.plus.R8_1	GH43	*Bacteroides*_sp._2_2_4
342	comp57054_c0_seq1.128.2041.minus.R1_1	GH43	*Bacteroides*_sp._2_2_4
343	comp114508_c0_seq1.1.1261.minus.R1_1	GH33	*Bacteroides*_sp._3_1_13

344	comp100282_c0_seq1.1.823.minus.R9_1	GH29	*Bacteroides*_sp._3_1_19
345	comp50206_c0_seq1.16.2141.minus.R7_1	GH2	*Bacteroides*_sp._3_1_19
346	comp62326_c0_seq1.1664.3481.plus.R1_1	GH123	*Bacteroides*_sp._3_1_19
347	comp34989_c0_seq2.132.2084.plus.R3_1	GH97	*Bacteroides*_sp._3_1_40A
348	comp100007_c0_seq1.1.722.minus.R7_1	GH28	*Bacteroides*_sp._CAG
349	comp100265_c0_seq1.1.736.minus.R8_1	GH13	*Bacteroides*_sp._CAG
350	comp100268_c0_seq1.51.1049.plus.R1_1	GH106	*Bacteroides*_sp._CAG
351	comp102443_c0_seq1.565.2724.minus.R1_1	GH97	*Bacteroides*_sp._CAG
352	comp102935_c0_seq1.1.1015.minus.R1_1	GH4	*Bacteroides*_sp._CAG
353	comp102944_c0_seq1.9.764.minus.R1_1	GH105	*Bacteroides*_sp._CAG
354	comp10520_c0_seq1.243.1847.minus.R9_1	GH43	*Bacteroides*_sp._CAG
355	comp10610_c0_seq1.84.1859.plus.R3_1	GH123	*Bacteroides*_sp._CAG
356	comp106636_c0_seq1.1.996.minus.R1_1	GH36	*Bacteroides*_sp._CAG
357	comp107640_c0_seq1.1.833.minus.R1_1	GH77	*Bacteroides*_sp._CAG
358	comp108955_c0_seq1.1.713.minus.R3_1	GH133	*Bacteroides*_sp._CAG
359	comp109059_c0_seq1.1.706.minus.R1_1	GH3	*Bacteroides*_sp._CAG
360	comp111921_c0_seq1.1.729.minus.R1_1	GH29	*Bacteroides*_sp._CAG
361	comp11397_c0_seq1.47.2971.plus.R3_1	GH2	*Bacteroides*_sp._CAG
362	comp11416_c0_seq1.23.1381.plus.R3_1	GH29	*Bacteroides*_sp._CAG
363	comp11669_c0_seq1.93.2090.minus.R1_1	GH13	*Bacteroides*_sp._CAG
364	comp119728_c0_seq1.1.839.minus.R8_1	GH29	*Bacteroides*_sp._CAG
365	comp121848_c0_seq1.9.1591.minus.R1_1	GH28	*Bacteroides*_sp._CAG
366	comp123543_c0_seq1.38.1375.plus.R1_1	GH2	*Bacteroides*_sp._CAG
367	comp12394_c0_seq1.1.729.minus.R1_1	GH97	*Bacteroides*_sp._CAG
368	comp127101_c0_seq1.1.710.minus.R8_1	GH53	*Bacteroides*_sp._CAG
369	comp127303_c0_seq1.1.1131.minus.R7_1	GH2	*Bacteroides*_sp._CAG
370	comp127592_c0_seq1.1.815.minus.R1_1	GH133	*Bacteroides*_sp._CAG
371	comp12770_c0_seq1.1.853.minus.R7_1	GH2	*Bacteroides*_sp._CAG
372	comp12788_c0_seq2.1.1054.minus.R8_1	GH2	*Bacteroides*_sp._CAG
373	comp12961_c0_seq1.13.1038.plus.R1_1	GH43	*Bacteroides*_sp._CAG
374	comp13121_c0_seq1.44.1396.plus.R7_1	GH28	*Bacteroides*_sp._CAG
375	comp13501_c0_seq1.124.2565.plus.R1_1	GH95	*Bacteroides*_sp._CAG
376	comp13636_c0_seq1.17.938.minus.R7_1	GH51	*Bacteroides*_sp._CAG
377	comp136647_c0_seq1.1.706.minus.R7_1	GH127	*Bacteroides*_sp._CAG
378	comp138545_c0_seq1.1.728.minus.R9_1	GH20	*Bacteroides*_sp._CAG
379	comp150891_c0_seq1.39.785.minus.R3_1	GH77	*Bacteroides*_sp._CAG
380	comp154863_c0_seq1.1.1017.minus.R1_1	GH133	*Bacteroides*_sp._CAG
381	comp155486_c0_seq1.1.708.minus.R1_1	GH97	*Bacteroides*_sp._CAG
382	comp16097_c0_seq1.22.769.minus.R1_1	GH13	*Bacteroides*_sp._CAG
383	comp163754_c0_seq1.1.1082.minus.R8_1	GH133	*Bacteroides*_sp._CAG
384	comp172469_c0_seq1.1.719.minus.R1_1	GH109	*Bacteroides*_sp._CAG
385	comp17257_c0_seq1.70.1255.minus.R3_1	GH84	*Bacteroides*_sp._CAG
386	comp18390_c0_seq1.1.2692.minus.R1_1	GH33	*Bacteroides*_sp._CAG
387	comp18390_c0_seq1.1.2692.minus.R1_1	GH78	*Bacteroides*_sp._CAG
388	comp19734_c0_seq1.1.1517.minus.R3_1	GH2	*Bacteroides*_sp._CAG
389	comp20901_c0_seq1.68.1429.plus.R3_1	GH43	*Bacteroides*_sp._CAG
390	comp211396_c0_seq1.1.775.minus.R1_1	GH109	*Bacteroides*_sp._CAG
391	comp22135_c0_seq1.1.827.minus.R3_1	GH28	*Bacteroides*_sp._CAG
392	comp22382_c0_seq1.14.2014.plus.R3_1	GH13	*Bacteroides*_sp._CAG
393	comp22799_c0_seq1.149.2161.minus.R1_1	GH67	*Bacteroides*_sp._CAG

394	comp22992_c0_seq1.89.1799.minus.R7_1	GH43	*Bacteroides*_sp._CAG
395	comp23510_c0_seq1.106.2010.plus.R9_1	GH92	*Bacteroides*_sp._CAG
396	comp24068_c0_seq1.11.961.plus.R7_1	GH43	*Bacteroides*_sp._CAG
397	comp24122_c0_seq1.678.1880.minus.R7_1	GH88	*Bacteroides*_sp._CAG
398	comp24556_c0_seq1.1346.3805.minus.R1_1	GH31	*Bacteroides*_sp._CAG
399	comp24852_c0_seq1.1344.2723.minus.R1_1	GH23	*Bacteroides*_sp._CAG
400	comp25959_c0_seq1.40.1752.minus.R8_1	GH13	*Bacteroides*_sp._CAG
401	comp25998_c0_seq1.1.730.minus.R3_1	GH20	*Bacteroides*_sp._CAG
402	comp26710_c0_seq2.1.769.minus.R9_1	GH57	*Bacteroides*_sp._CAG
403	comp26733_c0_seq1.11.1862.minus.R3_1	GH51	*Bacteroides*_sp._CAG
404	comp26778_c0_seq1.1.867.minus.R3_1	GH73	*Bacteroides*_sp._CAG
405	comp26948_c0_seq1.1275.3270.minus.R3_1	GH67	*Bacteroides*_sp._CAG
406	comp27892_c0_seq1.200.1404.minus.R1_1	GH105	*Bacteroides*_sp._CAG
407	comp28801_c0_seq1.1609.3996.plus.R1_1	GH127	*Bacteroides*_sp._CAG
408	comp28801_c0_seq1.4012.5958.plus.R1_1	GH97	*Bacteroides*_sp._CAG
409	comp28939_c0_seq1.1.2140.minus.R7_1	GH105	*Bacteroides*_sp._CAG
410	comp29374_c0_seq1.21.962.plus.R3_1	GH20	*Bacteroides*_sp._CAG
411	comp29546_c0_seq1.38.1987.plus.R7_1	GH133	*Bacteroides*_sp._CAG
412	comp29873_c0_seq1.1.963.minus.R3_1	GH20	*Bacteroides*_sp._CAG
413	comp30230_c0_seq3.84.1055.plus.R3_1	GH130	*Bacteroides*_sp._CAG
414	comp30237_c0_seq1.20.1597.plus.R9_1	GH109	*Bacteroides*_sp._CAG
415	comp30280_c0_seq1.49.2367.minus.R3_1	GH105	*Bacteroides*_sp._CAG
416	comp30491_c0_seq1.1.765.minus.R9_1	GH133	*Bacteroides*_sp._CAG
417	comp30575_c0_seq1.1.769.minus.R1_1	GH51	*Bacteroides*_sp._CAG
418	comp31153_c0_seq1.1.1659.minus.R9_1	GH105	*Bacteroides*_sp._CAG
419	comp31241_c0_seq1.19.1304.minus.R3_1	GH89	*Bacteroides*_sp._CAG
420	comp31285_c0_seq10.13.1593.plus.R3_1	GH109	*Bacteroides*_sp._CAG
421	comp31910_c0_seq1.1018.2650.minus.R3_1	GH105	*Bacteroides*_sp._CAG
422	comp32228_c0_seq1.194.1159.minus.R1_1	GH23	*Bacteroides*_sp._CAG
423	comp32593_c0_seq1.39.2300.plus.R1_1	GH92	*Bacteroides*_sp._CAG
424	comp32793_c0_seq2.276.2174.plus.R3_1	GH89	*Bacteroides*_sp._CAG
425	comp33405_c0_seq1.18.1955.plus.R3_1	GH51	*Bacteroides*_sp._CAG
426	comp33436_c0_seq1.43.2136.minus.R1_1	GH43	*Bacteroides*_sp._CAG
427	comp33900_c0_seq1.1.1255.minus.R1_1	GH9	*Bacteroides*_sp._CAG
428	comp34655_c0_seq4.134.940.plus.R3_1	GH110	*Bacteroides*_sp._CAG
429	comp34766_c0_seq1.28.837.plus.R8_1	GH51	*Bacteroides*_sp._CAG
430	comp34981_c0_seq2.1.950.minus.R7_1	GH23	*Bacteroides*_sp._CAG
431	comp34989_c0_seq4.132.2090.plus.R3_1	GH97	*Bacteroides*_sp._CAG
432	comp35236_c0_seq1.259.2681.minus.R3_1	GH3	*Bacteroides*_sp._CAG
433	comp35402_c0_seq2.17.2356.minus.R3_1	GH35	*Bacteroides*_sp._CAG
434	comp35487_c0_seq2.1.1086.minus.R3_1	GH31	*Bacteroides*_sp._CAG
435	comp35492_c0_seq5.181.1113.minus.R3_1	GH25	*Bacteroides*_sp._CAG
436	comp35651_c0_seq8.1129.3561.plus.R3_1	GH2	*Bacteroides*_sp._CAG
437	comp35749_c0_seq1.1.2418.minus.R3_1	GH2	*Bacteroides*_sp._CAG
438	comp35947_c0_seq5.1.986.minus.R3_1	GH51	*Bacteroides*_sp._CAG
439	comp36057_c0_seq1.280.2220.plus.R3_1	GH133	*Bacteroides*_sp._CAG
440	comp36057_c0_seq16.67.2025.plus.R3_1	GH133	*Bacteroides*_sp._CAG
441	comp36057_c0_seq2.3311.4684.plus.R3_1	GH57	*Bacteroides*_sp._CAG
442	comp36057_c0_seq4.61.2028.plus.R3_1	GH133	*Bacteroides*_sp._CAG
443	comp36098_c0_seq1.14.2164.plus.R3_1	GH2	*Bacteroides*_sp._CAG

444	comp36102_c0_seq1.6.1343.plus.R9_1	GH4	*Bacteroides*_sp._CAG
445	comp36312_c0_seq1.38.1219.plus.R3_1	GH97	*Bacteroides*_sp._CAG
446	comp36462_c0_seq3.21.1364.plus.R3_1	GH20	*Bacteroides*_sp._CAG
447	comp36462_c0_seq4.29.1615.plus.R3_1	GH20	*Bacteroides*_sp._CAG
448	comp36616_c0_seq11.110.2305.minus.R3_1	GH36	*Bacteroides*_sp._CAG
449	comp36616_c0_seq3.2399.4594.minus.R3_1	GH36	*Bacteroides*_sp._CAG
450	comp36616_c0_seq4.1.1473.minus.R3_1	GH32	*Bacteroides*_sp._CAG
451	comp38533_c0_seq1.574.1959.plus.R9_1	GH29	*Bacteroides*_sp._CAG
452	comp38707_c0_seq3.74.1336.minus.R9_1	GH92	*Bacteroides*_sp._CAG
453	comp38883_c0_seq1.1760.4101.minus.R9_1	GH31	*Bacteroides*_sp._CAG
454	comp38883_c0_seq5.30.1584.minus.R9_1	GH109	*Bacteroides*_sp._CAG
455	comp39212_c0_seq4.868.1869.minus.R9_1	GH4	*Bacteroides*_sp._CAG
456	comp39218_c0_seq1.203.2411.minus.R3_1	GH36	*Bacteroides*_sp._CAG
457	comp40010_c0_seq1.85.2032.minus.R9_1	GH133	*Bacteroides*_sp._CAG
458	comp40148_c0_seq1.11.2128.plus.R9_1	GH97	*Bacteroides*_sp._CAG
459	comp40255_c0_seq4.88.933.plus.R9_1	GH10	*Bacteroides*_sp._CAG
460	comp40379_c0_seq3.2.889.plus.R9_1	GH97	*Bacteroides*_sp._CAG
461	comp40788_c0_seq2.9.2437.minus.R8_1	GH127	*Bacteroides*_sp._CAG
462	comp41005_c0_seq1.1178.3190.plus.R3_1	GH13	*Bacteroides*_sp._CAG
463	comp41112_c0_seq2.237.2916.minus.R1_1	GH77	*Bacteroides*_sp._CAG
464	comp41228_c0_seq1.935.1923.minus.R3_1	GH109	*Bacteroides*_sp._CAG
465	comp42228_c0_seq1.1.960.minus.R1_1	GH89	*Bacteroides*_sp._CAG
466	comp43440_c0_seq1.35.1036.plus.R8_1	GH4	*Bacteroides*_sp._CAG
467	comp43763_c0_seq1.78.1872.minus.R3_1	GH2	*Bacteroides*_sp._CAG
468	comp43939_c0_seq1.1.967.minus.R1_1	GH95	*Bacteroides*_sp._CAG
469	comp44268_c0_seq1.38.2029.plus.R8_1	GH105	*Bacteroides*_sp._CAG
470	comp44324_c0_seq1.13.1422.minus.R1_1	GH127	*Bacteroides*_sp._CAG
471	comp44476_c0_seq1.8.2161.plus.R7_1	GH97	*Bacteroides*_sp._CAG
472	comp44476_c0_seq3.10.771.plus.R7_1	GH97	*Bacteroides*_sp._CAG
473	comp44887_c0_seq2.1.704.minus.R7_1	GH127	*Bacteroides*_sp._CAG
474	comp44988_c0_seq1.15.1382.plus.R3_1	GH43	*Bacteroides*_sp._CAG
475	comp46186_c0_seq1.15.881.plus.R8_1	GH30	*Bacteroides*_sp._CAG
476	comp46881_c0_seq1.508.2730.minus.R1_1	GH3	*Bacteroides*_sp._CAG
477	comp46934_c0_seq4.63.1307.plus.R8_1	GH77	*Bacteroides*_sp._CAG
478	comp47014_c0_seq1.1.1692.minus.R8_1	GH105	*Bacteroides*_sp._CAG
479	comp48388_c0_seq1.164.2572.plus.R8_1	GH127	*Bacteroides*_sp._CAG
480	comp48666_c0_seq1.21.992.plus.R1_1	GH43	*Bacteroides*_sp._CAG
481	comp48717_c0_seq1.46.2366.minus.R8_1	GH20	*Bacteroides*_sp._CAG
482	comp48717_c0_seq6.46.2379.minus.R8_1	GH20	*Bacteroides*_sp._CAG
483	comp48843_c0_seq11.120.1322.plus.R8_1	GH57	*Bacteroides*_sp._CAG
484	comp48859_c0_seq1.242.1627.minus.R8_1	GH57	*Bacteroides*_sp._CAG
485	comp48859_c0_seq2.1.1020.minus.R8_1	GH57	*Bacteroides*_sp._CAG
486	comp49059_c0_seq3.894.3168.minus.R8_1	GH2	*Bacteroides*_sp._CAG
487	comp49059_c0_seq6.1.1428.minus.R8_1	GH2	*Bacteroides*_sp._CAG
488	comp49356_c0_seq1.35.1567.plus.R8_1	GH133	*Bacteroides*_sp._CAG
489	comp49515_c0_seq6.46.1458.plus.R8_1	GH13	*Bacteroides*_sp._CAG
490	comp49626_c1_seq7.1.924.minus.R8_1	GH4	*Bacteroides*_sp._CAG
491	comp49833_c0_seq2.7152.9050.plus.R8_1	GH43	*Bacteroides*_sp._CAG
492	comp49984_c0_seq2.430.3132.minus.R8_1	GH77	*Bacteroides*_sp._CAG
493	comp50038_c0_seq1.1.1119.minus.R1_1	GH92	*Bacteroides*_sp._CAG

494	comp50057_c0_seq3.2019.3999.minus.R8_1	GH20	*Bacteroides*_sp._CAG
495	comp50120_c0_seq1.92.2590.plus.R1_1	GH51	*Bacteroides*_sp._CAG
496	comp50120_c1_seq1.2502.4814.minus.R1_1	GH105	*Bacteroides*_sp._CAG
497	comp50249_c0_seq1.501.1469.plus.R7_1	GH130	*Bacteroides*_sp._CAG
498	comp51948_c0_seq3.142.2107.minus.R7_1	GH2	*Bacteroides*_sp._CAG
499	comp52166_c0_seq1.1.1951.minus.R3_1	GH43	*Bacteroides*_sp._CAG
500	comp52190_c1_seq1.276.2531.minus.R1_1	GH92	*Bacteroides*_sp._CAG
501	comp52511_c0_seq1.11.1555.plus.R7_1	GH109	*Bacteroides*_sp._CAG
502	comp52511_c0_seq2.82.1629.plus.R7_1	GH109	*Bacteroides*_sp._CAG
503	comp52866_c0_seq2.1.862.minus.R7_1	GH29	*Bacteroides*_sp._CAG
504	comp54007_c0_seq2.122.2530.plus.R1_1	GH127	*Bacteroides*_sp._CAG
505	comp54142_c0_seq3.1181.2878.plus.R1_1	GH13	*Bacteroides*_sp._CAG
506	comp54146_c0_seq3.1145.3418.minus.R7_1	GH92	*Bacteroides*_sp._CAG
507	comp54291_c0_seq1.1749.3293.plus.R7_1	GH51	*Bacteroides*_sp._CAG
508	comp54358_c0_seq2.117.2480.plus.R1_1	GH3	*Bacteroides*_sp._CAG
509	comp54401_c0_seq3.8.2332.plus.R7_1	GH20	*Bacteroides*_sp._CAG
510	comp54401_c0_seq4.46.2322.plus.R7_1	GH20	*Bacteroides*_sp._CAG
511	comp54633_c0_seq1.488.1677.minus.R7_1	GH53	*Bacteroides*_sp._CAG
512	comp54720_c0_seq1.49.2451.plus.R3_1	GH2	*Bacteroides*_sp._CAG
513	comp54730_c0_seq3.10.1959.minus.R7_1	GH20	*Bacteroides*_sp._CAG
514	comp54730_c0_seq5.122.1798.minus.R7_1	GH20	*Bacteroides*_sp._CAG
515	comp54731_c0_seq9.20.1339.plus.R7_1	GH20	*Bacteroides*_sp._CAG
516	comp54966_c0_seq7.27.1970.plus.R7_1	GH133	*Bacteroides*_sp._CAG
517	comp55044_c0_seq2.2555.3919.plus.R7_1	GH57	*Bacteroides*_sp._CAG
518	comp55044_c0_seq8.535.1899.plus.R7_1	GH57	*Bacteroides*_sp._CAG
519	comp55346_c0_seq5.21.1614.minus.R7_1	GH13	*Bacteroides*_sp._CAG
520	comp57054_c0_seq2.128.2026.minus.R1_1	GH43	*Bacteroides*_sp._CAG
521	comp57179_c0_seq1.752.2302.plus.R1_1	GH109	*Bacteroides*_sp._CAG
522	comp57570_c0_seq1.30.2367.minus.R8_1	GH20	*Bacteroides*_sp._CAG
523	comp57624_c0_seq1.32.1182.minus.R8_1	GH20	*Bacteroides*_sp._CAG
524	comp57826_c0_seq1.17.1624.plus.R1_1	GH28	*Bacteroides*_sp._CAG
525	comp57976_c0_seq4.19.2013.plus.R1_1	GH13	*Bacteroides*_sp._CAG
526	comp58421_c0_seq1.1074.3152.plus.R1_1	GH43	*Bacteroides*_sp._CAG
527	comp58421_c0_seq1.1074.3152.plus.R1_1	GH43	*Bacteroides*_sp._CAG
528	comp58686_c0_seq2.18.995.plus.R1_1	GH109	*Bacteroides*_sp._CAG
529	comp59037_c0_seq1.864.2564.plus.R1_1	GH13	*Bacteroides*_sp._CAG
530	comp59468_c0_seq2.70.1527.plus.R1_1	GH97	*Bacteroides*_sp._CAG
531	comp59476_c0_seq2.1201.2190.plus.R1_1	GH23	*Bacteroides*_sp._CAG
532	comp59591_c0_seq1.567.2886.minus.R1_1	GH65	*Bacteroides*_sp._CAG
533	comp59614_c0_seq1.264.2519.minus.R1_1	GH92	*Bacteroides*_sp._CAG
534	comp59614_c0_seq3.579.2894.minus.R1_1	GH92	*Bacteroides*_sp._CAG
535	comp59831_c0_seq2.31.1233.plus.R1_1	GH88	*Bacteroides*_sp._CAG
536	comp60413_c0_seq3.1405.2796.plus.R1_1	GH57	*Bacteroides*_sp._CAG
537	comp60721_c0_seq1.76.1762.minus.R1_1	GH20	*Bacteroides*_sp._CAG
538	comp60916_c0_seq4.973.2925.plus.R1_1	GH13	*Bacteroides*_sp._CAG
539	comp61014_c0_seq2.71.2377.plus.R1_1	GH20	*Bacteroides*_sp._CAG
540	comp61176_c0_seq1.1178.3187.plus.R1_1	GH13	*Bacteroides*_sp._CAG
541	comp61356_c0_seq1.31.2539.minus.R1_1	GH2	*Bacteroides*_sp._CAG
542	comp61356_c0_seq2.1.2179.minus.R1_1	GH2	*Bacteroides*_sp._CAG
543	comp61427_c0_seq1.433.2595.plus.R1_1	GH97	*Bacteroides*_sp._CAG

544	comp61431_c0_seq1.60.2135.plus.R1_1	GH43	*Bacteroides*_sp._CAG
545	comp61597_c0_seq5.34.1578.minus.R1_1	GH51	*Bacteroides*_sp._CAG
546	comp61810_c0_seq5.2939.4948.minus.R1_1	GH13	*Bacteroides*_sp._CAG
547	comp61821_c0_seq11.2234.4588.minus.R1_1	GH35	*Bacteroides*_sp._CAG
548	comp61821_c0_seq1.206.1570.minus.R1_1	GH57	*Bacteroides*_sp._CAG
549	comp61821_c0_seq3.43.1416.minus.R1_1	GH57	*Bacteroides*_sp._CAG
550	comp61821_c0_seq4.2723.4660.minus.R1_1	GH133	*Bacteroides*_sp._CAG
551	comp61821_c0_seq5.1318.3282.minus.R1_1	GH133	*Bacteroides*_sp._CAG
552	comp61887_c0_seq1.870.2177.minus.R1_1	GH29	*Bacteroides*_sp._CAG
553	comp62134_c0_seq7.39.1040.plus.R1_1	GH4	*Bacteroides*_sp._CAG
554	comp62246_c0_seq1.122.2551.minus.R1_1	GH2	*Bacteroides*_sp._CAG
555	comp62326_c0_seq2.231.2012.plus.R1_1	GH123	*Bacteroides*_sp._CAG
556	comp62326_c0_seq4.1664.3481.plus.R1_1	GH123	*Bacteroides*_sp._CAG
557	comp62328_c0_seq10.2460.4577.minus.R1_1	GH13	*Bacteroides*_sp._CAG
558	comp62428_c0_seq2.1181.2617.plus.R1_1	GH109	*Bacteroides*_sp._CAG
559	comp62428_c0_seq2.12.1085.plus.R1_1	GH109	*Bacteroides*_sp._CAG
560	comp62428_c0_seq3.1139.2626.plus.R1_1	GH109	*Bacteroides*_sp._CAG
561	comp62464_c0_seq1.73.1581.plus.R1_1	GH30	*Bacteroides*_sp._CAG
562	comp62511_c0_seq1.926.2932.plus.R1_1	GH13	*Bacteroides*_sp._CAG
563	comp62511_c0_seq3.926.2932.plus.R1_1	GH13	*Bacteroides*_sp._CAG
564	comp62553_c0_seq2.672.3080.plus.R1_1	GH95	*Bacteroides*_sp._CAG
565	comp62578_c0_seq1.1.1035.minus.R3_1	GH43	*Bacteroides*_sp._CAG
566	comp62617_c0_seq1.81.1753.minus.R1_1	GH43	*Bacteroides*_sp._CAG
567	comp62629_c0_seq1.1197.3101.plus.R1_1	GH29	*Bacteroides*_sp._CAG
568	comp62629_c0_seq1.29.1171.plus.R1_1	GH109	*Bacteroides*_sp._CAG
569	comp62771_c0_seq1.4414.6615.plus.R1_1	GH36	*Bacteroides*_sp._CAG
570	comp62771_c0_seq3.39.1910.plus.R1_1	GH32	*Bacteroides*_sp._CAG
571	comp62810_c0_seq1.22.1386.minus.R3_1	GH105	*Bacteroides*_sp._CAG
572	comp62915_c0_seq3.1.1737.minus.R1_1	GH2	*Bacteroides*_sp._CAG
573	comp62915_c0_seq5.259.2138.minus.R1_1	GH29	*Bacteroides*_sp._CAG
574	comp62930_c0_seq3.2719.3999.plus.R1_1	GH23	*Bacteroides*_sp._CAG
575	comp62992_c0_seq1.1994.3469.plus.R1_1	GH125	*Bacteroides*_sp._CAG
576	comp62992_c0_seq1.3504.5777.plus.R1_1	GH92	*Bacteroides*_sp._CAG
577	comp62992_c0_seq2.15.2069.plus.R1_1	GH20	*Bacteroides*_sp._CAG
578	comp62997_c0_seq2.700.2835.plus.R1_1	GH97	*Bacteroides*_sp._CAG
579	comp63112_c0_seq1.5405.7219.plus.R1_1	GH29	*Bacteroides*_sp._CAG
580	comp63453_c0_seq1.7732.9276.plus.R1_1	GH51	*Bacteroides*_sp._CAG
581	comp63453_c0_seq1.9283.11259.plus.R1_1	GH51	*Bacteroides*_sp._CAG
582	comp63562_c0_seq1.44.1785.minus.R3_1	GH2	*Bacteroides*_sp._CAG
583	comp63636_c0_seq1.27.1478.plus.R1_1	GH18	*Bacteroides*_sp._CAG
584	comp63805_c0_seq1.1.1255.minus.R3_1	GH2	*Bacteroides*_sp._CAG
585	comp63827_c0_seq1.796.2677.minus.R1_1	GH97	*Bacteroides*_sp._CAG
586	comp64377_c0_seq1.1.1582.minus.R3_1	GH31	*Bacteroides*_sp._CAG
587	comp65490_c0_seq1.5015.5971.plus.R1_1	GH18	*Bacteroides*_sp._CAG
588	comp65790_c0_seq1.1.1344.minus.R3_1	GH28	*Bacteroides*_sp._CAG
589	comp66755_c0_seq1.20.1528.plus.R8_1	GH2	*Bacteroides*_sp._CAG
590	comp67222_c0_seq1.102.1095.minus.R1_1	GH43	*Bacteroides*_sp._CAG
591	comp67605_c0_seq1.1180.3126.minus.R1_1	GH97	*Bacteroides*_sp._CAG
592	comp68177_c0_seq1.23.1147.plus.R7_1	GH29	*Bacteroides*_sp._CAG
593	comp69107_c0_seq1.21.908.plus.R8_1	GH33	*Bacteroides*_sp._CAG

594	comp69571_c0_seq1.21.1095.minus.R3_1	GH31	*Bacteroides*_sp._CAG
595	comp7061_c0_seq2.131.1718.minus.R9_1	GH97	*Bacteroides*_sp._CAG
596	comp70792_c0_seq1.20.1453.minus.R7_1	GH28	*Bacteroides*_sp._CAG
597	comp71197_c0_seq1.1.1177.minus.R3_1	GH106	*Bacteroides*_sp._CAG
598	comp71286_c0_seq1.35.1447.plus.R1_1	GH109	*Bacteroides*_sp._CAG
599	comp7145_c0_seq1.1.2155.minus.R9_1	GH133	*Bacteroides*_sp._CAG
600	comp72651_c0_seq1.59.1597.plus.R1_1	GH51	*Bacteroides*_sp._CAG
601	comp74044_c0_seq1.1.1277.minus.R3_1	GH97	*Bacteroides*_sp._CAG
602	comp75202_c0_seq1.7.1578.plus.R1_1	GH109	*Bacteroides*_sp._CAG
603	comp77683_c0_seq1.1.1017.minus.R8_1	GH13	*Bacteroides*_sp._CAG
604	comp79362_c0_seq1.17.1616.minus.R3_1	GH3	*Bacteroides*_sp._CAG
605	comp81276_c0_seq1.32.862.plus.R3_1	GH123	*Bacteroides*_sp._CAG
606	comp81535_c0_seq1.101.1141.plus.R3_1	GH105	*Bacteroides*_sp._CAG
607	comp82145_c0_seq1.153.2161.minus.R1_1	GH29	*Bacteroides*_sp._CAG
608	comp82504_c0_seq1.1008.2074.minus.R7_1	GH43	*Bacteroides*_sp._CAG
609	comp84173_c0_seq1.57.773.plus.R3_1	GH105	*Bacteroides*_sp._CAG
610	comp84665_c0_seq1.153.2129.plus.R1_1	GH33	*Bacteroides*_sp._CAG
611	comp84769_c0_seq1.223.2022.plus.R1_1	GH84	*Bacteroides*_sp._CAG
612	comp84868_c0_seq1.1.812.minus.R7_1	GH84	*Bacteroides*_sp._CAG
613	comp85398_c0_seq1.10.1329.plus.R1_1	GH43	*Bacteroides*_sp._CAG
614	comp85954_c0_seq1.3070.4512.plus.R1_1	GH5	*Bacteroides*_sp._CAG
615	comp86115_c0_seq1.1.1204.minus.R1_1	GH32	*Bacteroides*_sp._CAG
616	comp86323_c0_seq1.1.1598.minus.R7_1	GH95	*Bacteroides*_sp._CAG
617	comp86572_c0_seq1.8.1111.plus.R8_1	GH92	*Bacteroides*_sp._CAG
618	comp86958_c0_seq1.1.1181.minus.R8_1	GH29	*Bacteroides*_sp._CAG
619	comp87805_c0_seq1.1.1347.minus.R1_1	GH28	*Bacteroides*_sp._CAG
620	comp88108_c0_seq1.2488.3744.plus.R1_1	GH10	*Bacteroides*_sp._CAG
621	comp8990_c0_seq1.426.2669.plus.R3_1	GH31	*Bacteroides*_sp._CAG
622	comp9279_c0_seq1.1.1561.minus.R3_1	GH51	*Bacteroides*_sp._CAG
623	comp9339_c0_seq1.64.1665.minus.R3_1	GH43	*Bacteroides*_sp._CAG
624	comp95112_c0_seq1.35.1276.plus.R3_1	GH51	*Bacteroides*_sp._CAG
625	comp9543_c0_seq1.1934.2908.plus.R3_1	GH43	*Bacteroides*_sp._CAG
626	comp96907_c0_seq1.101.1144.plus.R1_1	GH20	*Bacteroides*_sp._CAG
627	comp98937_c0_seq1.1.795.minus.R1_1	GH13	*Bacteroides*_sp._CAG
628	comp98988_c0_seq1.1.763.minus.R3_1	GH28	*Bacteroides*_sp._CAG
629	comp25826_c0_seq1.3687.7871.plus.R3_1	GH2	*Bacteroides*_sp._CAG
630	comp46900_c0_seq1.1.3022.minus.R8_1	GH85	*Bacteroides*_sp._CAG
631	comp48373_c0_seq2.703.3792.plus.R1_1	GH2	*Bacteroides*_sp._CAG
632	comp49895_c0_seq1.575.4177.plus.R8_1	GH2	*Bacteroides*_sp._CAG
633	comp54291_c0_seq3.6.3332.plus.R7_1	GH35	*Bacteroides*_sp._CAG
634	comp54291_c0_seq3.6.3332.plus.R7_1	GH43	*Bacteroides*_sp._CAG
635	comp55144_c0_seq2.12.3002.plus.R7_1	GH2	*Bacteroides*_sp._CAG
636	comp55144_c0_seq4.19.2700.plus.R7_1	GH2	*Bacteroides*_sp._CAG
637	comp60505_c0_seq1.25.2853.minus.R1_1	GH3	*Bacteroides*_sp._CAG
638	comp61732_c0_seq2.19.2691.plus.R1_1	GH77	*Bacteroides*_sp._CAG
639	comp61920_c0_seq1.37.3237.minus.R1_1	GH2	*Bacteroides*_sp._CAG
640	comp62344_c1_seq1.961.3726.minus.R1_1	GH77	*Bacteroides*_sp._CAG
641	comp62421_c0_seq1.62.3106.minus.R1_1	GH85	*Bacteroides*_sp._CAG
642	comp62602_c0_seq3.2380.4960.minus.R1_1	GH115	*Bacteroides*_sp._CAG
643	comp62915_c0_seq2.940.4539.minus.R1_1	GH2	*Bacteroides*_sp._CAG

644	comp62915_c0_seq6.940.4542.minus.R1_1	GH2	*Bacteroides*_sp._CAG
645	comp62992_c0_seq1.5978.9583.plus.R1_1	GH2	*Bacteroides*_sp._CAG
646	comp76270_c0_seq1.40.2637.plus.R1_1	GH31	*Bacteroides*_sp._CAG
647	comp85954_c0_seq1.592.3039.plus.R1_1	GH95	*Bacteroides*_sp._CAG
648	comp89444_c0_seq1.38.3710.minus.R1_1	GH2	*Bacteroides*_sp._CAG
649	comp9325_c0_seq1.7.2973.plus.R3_1	GH95	*Bacteroides*_sp._CAG
650	comp78739_c0_seq1.1.1372.minus.R1_1	GH85	*Bacteroides*_sp._HPS0048
651	comp59207_c0_seq2.9.3752.plus.R1_1	GH85	*Bacteroides*_sp._HPS0048
652	comp199269_c0_seq1.1.759.minus.R1_1	GH16	*Bacteroides*_sp._I48
653	comp31699_c0_seq1.1.2684.minus.R1_1	GH78	*Bacteroides*_sp._I48
654	comp61540_c0_seq1.959.2350.minus.R1_1	GH109	*Bacteroides*_sp._I48
655	comp44951_c0_seq1.1.784.minus.R3_1	GH133	*Bacteroides_stercorirosoris*
656	comp29380_c0_seq1.1.732.minus.R9_1	GH3	*Bacteroides_stercoris*
657	comp48717_c0_seq8.25.2370.minus.R8_1	GH20	*Bacteroides_thetaiotaomicron*
658	comp77612_c0_seq1.34.1314.plus.R9_1	GH109	*Bacteroides_thetaiotaomicron*
659	comp60311_c0_seq7.60.3395.minus.R1_1	GH2	*Bacteroides_thetaiotaomicron*
660	comp114364_c0_seq1.1.745.minus.R1_1	GH13	*Bacteroides_uniformis*
661	comp135522_c0_seq1.1.806.minus.R8_1	GH95	*Bacteroides_uniformis*
662	comp145939_c0_seq1.1.1522.minus.R1_1	GH36	*Bacteroides_uniformis*
663	comp18496_c0_seq1.48.1163.plus.R3_1	GH26	*Bacteroides_uniformis*
664	comp189187_c0_seq1.1.1000.minus.R1_1	GH38	*Bacteroides_uniformis*
665	comp28574_c0_seq1.1.1350.minus.R7_1	GH2	*Bacteroides_uniformis*
666	comp39581_c0_seq2.1.1089.minus.R8_1	GH66	*Bacteroides_uniformis*
667	comp41536_c0_seq2.15.1381.minus.R8_1	GH9	*Bacteroides_uniformis*
668	comp49356_c0_seq3.39.1988.plus.R8_1	GH133	*Bacteroides_uniformis*
669	comp50167_c0_seq1.1.778.minus.R7_1	GH36	*Bacteroides_uniformis*
670	comp53889_c0_seq1.266.2215.plus.R1_1	GH133	*Bacteroides_uniformis*
671	comp60556_c0_seq2.1933.4260.plus.R1_1	GH3	*Bacteroides_uniformis*
672	comp60556_c0_seq2.4342.6837.plus.R1_1	GH3	*Bacteroides_uniformis*
673	comp70891_c0_seq1.20.1270.plus.R3_1	GH5	*Bacteroides_uniformis*
674	comp82813_c0_seq1.1.722.minus.R3_1	GH77	*Bacteroides_uniformis*
675	comp39944_c0_seq1.982.3564.minus.R3_1	GH2	*Bacteroides_vulgatus*
676	comp84737_c0_seq1.1817.2890.minus.R1_1	GH99	*Bacteroides_vulgatus*
677	comp99097_c0_seq1.1.2187.minus.R1_1	GH84	*Bacteroides_vulgatus*
678	comp73099_c0_seq1.935.2362.minus.R1_1	GH109	*Bacteroides_xylanisolvens*
679	comp49149_c0_seq2.1.720.minus.R8_1	GH3	Candidatus_Bacteroides_timonensis
680	comp91062_c0_seq1.15.1403.plus.R8_1	GH16	Candidatus_Bacteroides_timonensis
681	comp111759_c0_seq1.1.791.minus.R8_1	GH27	*Bacteriodes*_norank
682	comp11449_c0_seq1.1.996.minus.R1_1	GH57	*Bacteriodes*_norank
683	comp21617_c0_seq1.825.1886.plus.R8_1	GH43	*Bacteriodes*_norank
684	comp28005_c0_seq1.78.2105.plus.R8_1	GH97	*Bacteriodes*_norank
685	comp31046_c0_seq1.68.844.plus.R1_1	GH23	*Bacteriodes*_norank
686	comp39212_c0_seq5.868.1872.minus.R9_1	GH4	*Bacteriodes*_norank
687	comp45088_c0_seq1.146.1621.plus.R1_1	GH109	*Bacteriodes*_norank
688	comp48961_c0_seq1.2033.4000.plus.R3_1	GH2	*Bacteriodes*_norank
689	comp63435_c0_seq1.59.1999.plus.R8_1	GH92	*Bacteriodes*_norank
690	comp64819_c0_seq1.504.2954.minus.R3_1	GH2	*Bacteriodes*_norank
691	comp70755_c0_seq1.1.1200.minus.R1_1	GH92	*Bacteriodes*_norank
692	comp71897_c0_seq1.50.1936.plus.R1_1	GH133	*Bacteriodes*_norank
693	comp86883_c0_seq1.303.1733.minus.R1_1	GH18	*Bacteriodes*_norank

694	comp9091_c0_seq1.35.1627.plus.R3_1	GH27	*Bacteriodes*_norank
695	comp103646_c0_seq1.1.1246.minus.R8_1	GH3	uncultured_*Bacteroides*_sp.
696	comp108402_c0_seq1.1.831.minus.R1_1	GH127	uncultured_*Bacteroides*_sp.
697	comp112687_c0_seq1.1.1224.minus.R7_1	GH20	uncultured_*Bacteroides*_sp.
698	comp11519_c0_seq1.1.1383.minus.R9_1	GH77	uncultured_*Bacteroides*_sp.
699	comp12191_c0_seq1.29.1201.plus.R9_1	GH92	uncultured_*Bacteroides*_sp.
700	comp22002_c0_seq1.24.2255.plus.R9_1	GH92	uncultured_*Bacteroides*_sp.
701	comp29876_c0_seq1.40.2373.plus.R9_1	GH20	uncultured_*Bacteroides*_sp.
702	comp29876_c0_seq2.3.2354.plus.R9_1	GH20	uncultured_*Bacteroides*_sp.
703	comp30435_c0_seq2.1.932.minus.R9_1	GH130	uncultured_*Bacteroides*_sp.
704	comp34711_c0_seq1.17.1696.plus.R3_1	GH33	uncultured_*Bacteroides*_sp.
705	comp37642_c0_seq1.22.1998.minus.R1_1	GH97	uncultured_*Bacteroides*_sp.
706	comp38988_c0_seq1.1.1208.minus.R7_1	GH43	uncultured_*Bacteroides*_sp.
707	comp39212_c0_seq2.1.715.minus.R9_1	GH4	uncultured_*Bacteroides*_sp.
708	comp40379_c0_seq1.127.1011.plus.R9_1	GH97	uncultured_*Bacteroides*_sp.
709	comp48272_c0_seq1.6.1826.plus.R9_1	GH29	uncultured_*Bacteroides*_sp.
710	comp50847_c0_seq1.56.2647.plus.R3_1	GH3	uncultured_*Bacteroides*_sp.
711	comp50946_c0_seq1.10.1242.plus.R7_1	GH88	uncultured_*Bacteroides*_sp.
712	comp58933_c0_seq1.1.839.minus.R9_1	GH20	uncultured_*Bacteroides*_sp.
713	comp63363_c0_seq1.1.808.minus.R3_1	GH36	uncultured_*Bacteroides*_sp.
714	comp64042_c0_seq1.1.1023.minus.R9_1	GH20	uncultured_*Bacteroides*_sp.
715	comp69406_c0_seq1.1.1123.minus.R9_1	GH13	uncultured_*Bacteroides*_sp.
716	comp69444_c0_seq1.1.842.minus.R9_1	GH3	uncultured_*Bacteroides*_sp.
717	comp85162_c0_seq1.1.733.minus.R9_1	GH13	uncultured_*Bacteroides*_sp.
718	comp12224_c0_seq1.33.1838.plus.R1_1	GH29	*Saccharicrinis_fermentans*
719	comp79526_c0_seq1.1.1024.minus.R1_1	GH2	*Bacteroidales_bacterium*_6E
720	comp82523_c0_seq1.65.1225.plus.R8_1	GH9	*Bacteroidales*_bacterium_KA00344
721	comp60629_c0_seq1.1612.2994.plus.R1_1	GH109	*Bacteroidales*_norank
722	comp62257_c0_seq3.52.1986.plus.R1_1	GH133	*Bacteroidales*_norank
723	comp62686_c0_seq1.1550.2773.plus.R1_1	GH109	*Bacteroidales*_norank
724	comp77047_c0_seq1.11.1267.plus.R7_1	GH109	*Bacteroidales*_norank
725	comp80026_c0_seq1.1.933.minus.R1_1	GH57	*Bacteroidales*_norank
726	comp55598_c0_seq3.120.1133.minus.R7_1	GH4	*Phocaeicola_abscessus*
727	comp67779_c0_seq1.44.809.minus.R3_1	GH16	*Phocaeicola_abscessus*
728	comp58653_c0_seq3.17.817.plus.R1_1	GH88	*Odoribacter_laneus*
729	comp141410_c0_seq1.188.1442.minus.R1_1	GH127	*Odoribacter_splanchnicus*
730	comp42049_c0_seq1.814.2784.plus.R3_1	GH20	*Odoribacter_splanchnicus*
731	comp102462_c0_seq1.89.1495.minus.R1_1	GH109	*Odoribacter_splanchnicus*_CAG
732	comp143104_c0_seq1.60.1628.plus.R1_1	GH33	*Barnesiella_intestinihominis*
733	comp27950_c0_seq2.1.1045.minus.R9_1	GH133	*Barnesiella_intestinihominis*
734	comp31938_c0_seq1.155.2101.minus.R7_1	GH133	*Barnesiella_intestinihominis*
735	comp39561_c0_seq3.8.1384.minus.R8_1	GH57	*Barnesiella_intestinihominis*
736	comp50587_c0_seq1.1.887.minus.R7_1	GH109	*Barnesiella_intestinihominis*
737	comp62421_c0_seq3.1.953.minus.R1_1	GH85	*Barnesiella_intestinihominis*
738	comp84132_c0_seq1.1.1057.minus.R1_1	GH57	*Barnesiella_intestinihominis*
739	comp36594_c0_seq2.5296.8343.plus.R3_1	GH85	*Barnesiella_intestinihominis*
740	comp145548_c0_seq1.1.973.minus.R1_1	GH2	*Barnesiella_viscericola*
741	comp15134_c0_seq1.52.1494.plus.R1_1	GH29	*Barnesiella_viscericola*
742	comp16582_c0_seq1.1.2929.minus.R1_1	GH84	*Barnesiella_viscericola*
743	comp21884_c0_seq1.40.2199.minus.R3_1	GH97	*Barnesiella_viscericola*

744	comp42756_c0_seq1.53.1999.minus.R1_1	GH133	*Barnesiella_viscericola*
745	comp50107_c1_seq3.7465.9240.plus.R1_1	GH66	*Barnesiella_viscericola*
746	comp51805_c0_seq1.37.1983.plus.R7_1	GH133	*Barnesiella_viscericola*
747	comp51954_c0_seq3.26.1649.minus.R1_1	GH97	*Barnesiella_viscericola*
748	comp52310_c0_seq2.27.2369.plus.R1_1	GH20	*Barnesiella_viscericola*
749	comp82287_c0_seq1.50.1627.plus.R1_1	GH31	*Barnesiella_viscericola*
750	comp83575_c0_seq1.1.2092.minus.R1_1	GH33	*Barnesiella_viscericola*
751	comp91371_c0_seq1.568.2043.minus.R1_1	GH20	*Barnesiella_viscericola*
752	comp47593_c0_seq1.22.3378.minus.R1_1	GH85	*Barnesiella_viscericola*
753	comp50107_c1_seq3.9334.12402.plus.R1_1	GH31	*Barnesiella_viscericola*
754	comp55909_c0_seq2.1.906.minus.R1_1	GH20	*Coprobacter_fastidiosus*
755	comp55909_c0_seq4.1.2018.minus.R1_1	GH20	*Coprobacter_fastidiosus*
756	comp59402_c0_seq1.2.1435.plus.R3_1	GH109	*Dysgonomonas_gadei*
757	comp67372_c0_seq1.26.1761.minus.R7_1	GH43	*Dysgonomonas_gadei*
758	comp11431_c0_seq1.1.1094.minus.R9_1	GH2	*Dysgonomonas_macrotermitis*
759	comp170203_c0_seq1.1.721.minus.R1_1	GH123	*Dysgonomonas*_sp._HGC4
760	comp85132_c0_seq1.7.1089.plus.R3_1	GH92	*Macellibacteroides*_sp._HH-ZS
761	comp60311_c0_seq1.17.3142.minus.R1_1	GH2	*Macellibacteroides*_sp._HH-ZS
762	comp11608_c0_seq1.1.1286.minus.R9_1	GH77	*Parabacteroides*_norank
763	comp17944_c0_seq1.1.1256.minus.R1_1	GH109	*Parabacteroides*_norank
764	comp30205_c0_seq1.423.1373.minus.R3_1	GH23	*Parabacteroides*_norank
765	comp35048_c0_seq4.741.2343.minus.R9_1	GH36	*Parabacteroides*_norank
766	comp35569_c0_seq1.93.2579.plus.R3_1	GH84	*Parabacteroides*_norank
767	comp35572_c0_seq2.870.2339.minus.R3_1	GH109	*Parabacteroides*_norank
768	comp39608_c0_seq1.1.2569.minus.R9_1	GH2	*Parabacteroides*_norank
769	comp48630_c0_seq1.1.1620.minus.R7_1	GH92	*Parabacteroides*_norank
770	comp54096_c0_seq2.2390.4696.plus.R7_1	GH20	*Parabacteroides*_norank
771	comp55143_c0_seq6.1626.3011.plus.R7_1	GH109	*Parabacteroides*_norank
772	comp55996_c0_seq1.48.1520.minus.R1_1	GH30	*Parabacteroides*_norank
773	comp59104_c0_seq1.61.1533.plus.R1_1	GH125	*Parabacteroides*_norank
774	comp60960_c0_seq1.684.3011.plus.R1_1	GH3	*Parabacteroides*_norank
775	comp61019_c0_seq2.2286.4529.plus.R1_1	GH92	*Parabacteroides*_norank
776	comp62193_c0_seq2.62.2212.minus.R1_1	GH92	*Parabacteroides*_norank
777	comp62748_c1_seq1.75.2426.minus.R1_1	GH20	*Parabacteroides*_norank
778	comp80435_c0_seq1.19.1421.minus.R1_1	GH109	*Parabacteroides*_norank
779	comp84603_c0_seq1.33.2219.minus.R1_1	GH20	*Parabacteroides*_norank
780	comp62050_c0_seq1.793.3405.minus.R1_1	GH3	*Parabacteroides*_norank
781	comp117535_c0_seq1.1.847.minus.R7_1	GH23	*Parabacteroides_distasonis*
782	comp29636_c0_seq1.1.707.minus.R7_1	GH109	*Parabacteroides_distasonis*
783	comp34471_c0_seq1.40.1782.minus.R3_1	GH20	*Parabacteroides_distasonis*
784	comp36801_c0_seq1.1.1147.minus.R9_1	GH13	*Parabacteroides_distasonis*
785	comp41242_c1_seq1.1771.3105.plus.R8_1	GH109	*Parabacteroides_distasonis*
786	comp47868_c0_seq5.18.2360.plus.R8_1	GH92	*Parabacteroides_distasonis*
787	comp49718_c0_seq1.1664.3736.minus.R7_1	GH29	*Parabacteroides_distasonis*
788	comp51215_c0_seq2.29.988.plus.R1_1	GH130	*Parabacteroides_distasonis*
789	comp53306_c0_seq1.1094.2545.minus.R1_1	GH109	*Parabacteroides_distasonis*
790	comp54905_c0_seq1.1.836.minus.R1_1	GH43	*Parabacteroides_distasonis*
791	comp55598_c0_seq4.25.1038.minus.R7_1	GH4	*Parabacteroides_distasonis*
792	comp60044_c0_seq2.9.1175.plus.R1_1	GH109	*Parabacteroides_distasonis*
793	comp61929_c0_seq1.22.2373.minus.R1_1	GH3	*Parabacteroides_distasonis*

794	comp61981_c0_seq1.820.2358.minus.R1_1	GH23	*Parabacteroides_distasonis*
795	comp61981_c0_seq2.1119.2648.minus.R1_1	GH23	*Parabacteroides_distasonis*
796	comp62490_c0_seq5.2751.4565.minus.R1_1	GH2	*Parabacteroides_distasonis*
797	comp62562_c0_seq2.36.1697.minus.R1_1	GH109	*Parabacteroides_distasonis*
798	comp62686_c0_seq1.28.1434.plus.R1_1	GH109	*Parabacteroides_distasonis*
799	comp79028_c0_seq1.1.909.minus.R7_1	GH125	*Parabacteroides_distasonis*
800	comp82982_c0_seq1.1.1014.minus.R3_1	GH92	*Parabacteroides_distasonis*
801	comp85383_c0_seq1.1.1223.minus.R9_1	GH2	*Parabacteroides_distasonis*
802	comp60066_c0_seq1.14.3631.plus.R1_1	GH2	*Parabacteroides_distasonis*
803	comp66053_c0_seq1.11.2698.plus.R8_1	GH77	*Parabacteroides_distasonis*
804	comp79123_c0_seq1.57.1307.plus.R1_1	GH109	*Parabacteroides_goldsteinii*
805	comp83185_c0_seq1.1.1326.minus.R1_1	GH33	*Parabacteroides_goldsteinii*
806	comp33213_c0_seq2.101.1129.minus.R3_1	GH43	*Parabacteroides_gordonii*
807	comp99105_c0_seq1.15.934.minus.R1_1	GH106	*Parabacteroides_gordonii*
808	comp42981_c0_seq1.13.3129.plus.R9_1	GH2	*Parabacteroides_gordonii*
809	comp26633_c0_seq1.47.1510.minus.R3_1	GH109	*Parabacteroides_johnsonii*
810	comp27517_c0_seq1.1.1363.minus.R8_1	GH109	*Parabacteroides_johnsonii*
811	comp31814_c0_seq1.3404.5401.plus.R3_1	GH78	*Parabacteroides_johnsonii*
812	comp50652_c0_seq3.639.2609.minus.R1_1	GH78	*Parabacteroides_johnsonii*
813	comp80670_c0_seq1.1.1106.minus.R1_1	GH99	*Parabacteroides_johnsonii*
814	comp38714_c0_seq1.1983.3047.plus.R8_1	GH43	*Parabacteroides_merdae*
815	comp49059_c0_seq1.1.1873.minus.R8_1	GH2	*Parabacteroides_merdae*
816	comp57639_c0_seq2.20.2344.minus.R1_1	GH20	*Parabacteroides_merdae*
817	comp102194_c0_seq1.1.1992.minus.R1_1	GH92	*Parabacteroides*_sp._CAG
818	comp10291_c0_seq1.21.1415.minus.R1_1	GH109	*Parabacteroides*_sp._CAG
819	comp106893_c0_seq1.41.1823.minus.R1_1	GH51	*Parabacteroides*_sp._CAG
820	comp107243_c0_seq1.1.931.minus.R1_1	GH127	*Parabacteroides*_sp._CAG
821	comp108953_c0_seq1.195.1068.minus.R1_1	GH43	*Parabacteroides*_sp._CAG
822	comp109033_c0_seq1.1.1032.minus.R1_1	GH2	*Parabacteroides*_sp._CAG
823	comp110452_c0_seq1.1.946.minus.R1_1	GH95	*Parabacteroides*_sp._CAG
824	comp118579_c0_seq1.1.913.minus.R1_1	GH3	*Parabacteroides*_sp._CAG
825	comp118880_c0_seq1.313.1389.minus.R1_1	GH109	*Parabacteroides*_sp._CAG
826	comp13359_c0_seq1.1.785.minus.R1_1	GH120	*Parabacteroides*_sp._CAG
827	comp136104_c0_seq1.1.708.minus.R7_1	GH92	*Parabacteroides*_sp._CAG
828	comp15130_c0_seq1.17.1459.plus.R7_1	GH20	*Parabacteroides*_sp._CAG
829	comp181249_c0_seq1.1.770.minus.R1_1	GH97	*Parabacteroides*_sp._CAG
830	comp19519_c0_seq1.251.1588.plus.R1_1	GH109	*Parabacteroides*_sp._CAG
831	comp24851_c0_seq1.55.1467.plus.R3_1	GH109	*Parabacteroides*_sp._CAG
832	comp25169_c0_seq2.2823.4803.minus.R1_1	GH133	*Parabacteroides*_sp._CAG
833	comp25462_c0_seq2.1.1179.minus.R9_1	GH109	*Parabacteroides*_sp._CAG
834	comp30206_c0_seq1.1442.3784.plus.R9_1	GH20	*Parabacteroides*_sp._CAG
835	comp31511_c0_seq1.61.1459.minus.R1_1	GH109	*Parabacteroides*_sp._CAG
836	comp33621_c0_seq1.1731.3116.plus.R1_1	GH109	*Parabacteroides*_sp._CAG
837	comp33962_c0_seq1.1.1095.minus.R1_1	GH20	*Parabacteroides*_sp._CAG
838	comp39718_c0_seq2.63.936.minus.R8_1	GH92	*Parabacteroides*_sp._CAG
839	comp39963_c0_seq2.171.2108.plus.R9_1	GH133	*Parabacteroides*_sp._CAG
840	comp41463_c0_seq1.669.1799.minus.R8_1	GH109	*Parabacteroides*_sp._CAG
841	comp41463_c0_seq2.1002.2160.minus.R8_1	GH109	*Parabacteroides*_sp._CAG
842	comp44236_c0_seq1.14.2347.minus.R7_1	GH20	*Parabacteroides*_sp._CAG
843	comp45292_c0_seq1.555.1856.plus.R9_1	GH57	*Parabacteroides*_sp._CAG

844	comp47868_c0_seq6.84.2393.plus.R8_1	GH92	*Parabacteroides*_sp._CAG
845	comp47868_c0_seq7.84.2393.plus.R8_1	GH92	*Parabacteroides*_sp._CAG
846	comp48235_c0_seq1.1.1322.minus.R1_1	GH13	*Parabacteroides*_sp._CAG
847	comp48386_c0_seq2.1.876.minus.R1_1	GH92	*Parabacteroides*_sp._CAG
848	comp49356_c0_seq10.38.1996.plus.R8_1	GH133	*Parabacteroides*_sp._CAG
849	comp49356_c0_seq2.981.1934.plus.R8_1	GH133	*Parabacteroides*_sp._CAG
850	comp60087_c0_seq2.26.1462.plus.R1_1	GH109	*Parabacteroides*_sp._CAG
851	comp60406_c0_seq5.1.2326.minus.R1_1	GH20	*Parabacteroides*_sp._CAG
852	comp60769_c0_seq1.1.2268.minus.R1_1	GH92	*Parabacteroides*_sp._CAG
853	comp60919_c0_seq1.1.1646.minus.R9_1	GH92	*Parabacteroides*_sp._CAG
854	comp60957_c0_seq1.10.2391.plus.R1_1	GH3	*Parabacteroides*_sp._CAG
855	comp61019_c0_seq3.16.996.plus.R1_1	GH130	*Parabacteroides*_sp._CAG
856	comp62691_c0_seq6.1.997.minus.R1_1	GH20	*Parabacteroides*_sp._CAG
857	comp63916_c0_seq1.1.708.minus.R1_1	GH109	*Parabacteroides*_sp._CAG
858	comp70180_c0_seq1.27.1445.minus.R1_1	GH29	*Parabacteroides*_sp._CAG
859	comp70701_c0_seq1.66.1396.minus.R8_1	GH109	*Parabacteroides*_sp._CAG
860	comp71106_c0_seq1.119.1255.minus.R7_1	GH109	*Parabacteroides*_sp._CAG
861	comp71604_c0_seq1.171.908.plus.R1_1	GH31	*Parabacteroides*_sp._CAG
862	comp75182_c0_seq1.1.1304.minus.R1_1	GH109	*Parabacteroides*_sp._CAG
863	comp79346_c0_seq1.1.1066.minus.R9_1	GH32	*Parabacteroides*_sp._CAG
864	comp82567_c0_seq1.16.1188.plus.R7_1	GH109	*Parabacteroides*_sp._CAG
865	comp84716_c0_seq1.10.1521.plus.R1_1	GH20	*Parabacteroides*_sp._CAG
866	comp87175_c0_seq1.43.1350.plus.R1_1	GH20	*Parabacteroides*_sp._CAG
867	comp89998_c0_seq1.1.1960.minus.R1_1	GH20	*Parabacteroides*_sp._CAG
868	comp91313_c0_seq1.1.837.minus.R7_1	GH92	*Parabacteroides*_sp._CAG
869	comp91581_c0_seq1.156.2192.plus.R7_1	GH36	*Parabacteroides*_sp._CAG
870	comp92451_c0_seq1.1.1067.minus.R1_1	GH29	*Parabacteroides*_sp._CAG
871	comp95085_c0_seq1.46.1350.minus.R7_1	GH57	*Parabacteroides*_sp._CAG
872	comp95114_c0_seq1.1.939.minus.R1_1	GH109	*Parabacteroides*_sp._CAG
873	comp98459_c0_seq1.19.1239.plus.R1_1	GH109	*Parabacteroides*_sp._CAG
874	comp77664_c0_seq1.10.2685.minus.R1_1	GH77	*Parabacteroides*_sp._CAG
875	comp45697_c0_seq1.1901.3768.minus.R9_1	GH13	*Parabacteroides*_sp._D13
876	comp38420_c0_seq3.34.1188.plus.R9_1	GH13	*Parabacteroides*_sp._D26
877	comp8865_c0_seq1.1.1015.minus.R7_1	GH2	*Parabacteroides*_sp._HGS0025
878	comp27961_c0_seq1.1.1278.minus.R7_1	GH13	*Parabacteroides*_sp._YL27
879	comp114028_c0_seq1.1.815.minus.R1_1	GH106	*Proteiniphilum_acetatigenes*
880	comp134413_c0_seq1.29.1393.plus.R1_1	GH92	*Tannerella*_sp._6_1_58FAA_CT1
881	comp28676_c0_seq1.1.978.minus.R7_1	GH13	*Tannerella*_sp._6_1_58FAA_CT1
882	comp29067_c0_seq1.15.1466.minus.R1_1	GH109	*Tannerella*_sp._6_1_58FAA_CT1
883	comp36105_c0_seq2.13.1464.minus.R3_1	GH109	*Tannerella*_sp._6_1_58FAA_CT1
884	comp72396_c0_seq1.1.1857.minus.R1_1	GH133	*Tannerella*_sp._6_1_58FAA_CT1
885	comp44527_c0_seq1.1.916.minus.R1_1	GH43	*Paraprevotella_clara*
886	comp51572_c0_seq1.486.2893.minus.R7_1	GH51	*Prevotella_brevis*
887	comp36570_c0_seq11.1582.3531.plus.R3_1	GH97	*Prevotella*_sp._10(H)
888	comp35673_c0_seq1.107.1543.plus.R3_1	GH74	*Prevotella*_sp._109
889	comp47138_c0_seq1.157.2859.minus.R3_1	GH77	*Prevotella*_sp._109
890	comp10559_c0_seq1.12.1430.plus.R3_1	GH28	*Prevotella*_sp._CAG
891	comp10719_c0_seq1.12.1652.minus.R3_1	GH43	*Prevotella*_sp._CAG
892	comp12040_c0_seq1.1.1001.minus.R8_1	GH13	*Prevotella*_sp._CAG
893	comp144097_c0_seq1.1.779.minus.R8_1	GH13	*Prevotella*_sp._CAG

894	comp18666_c0_seq1.53.1963.minus.R3_1	GH10	*Prevotella*_sp._CAG
895	comp19546_c0_seq1.25.2826.minus.R1_1	GH16	*Prevotella*_sp._CAG
896	comp25405_c0_seq1.272.2107.plus.R3_1	GH35	*Prevotella*_sp._CAG
897	comp28345_c0_seq1.1532.4157.minus.R3_1	GH105	*Prevotella*_sp._CAG
898	comp28345_c0_seq1.1532.4157.minus.R3_1	GH28	*Prevotella*_sp._CAG
899	comp29082_c0_seq5.1879.3453.plus.R3_1	GH28	*Prevotella*_sp._CAG
900	comp32491_c0_seq2.1001.3070.plus.R3_1	GH13	*Prevotella*_sp._CAG
901	comp34069_c0_seq1.1188.2212.minus.R3_1	GH4	*Prevotella*_sp._CAG
902	comp34749_c0_seq4.78.2189.plus.R3_1	GH97	*Prevotella*_sp._CAG
903	comp36057_c0_seq11.69.2012.plus.R3_1	GH133	*Prevotella*_sp._CAG
904	comp36057_c0_seq13.48.1991.plus.R3_1	GH133	*Prevotella*_sp._CAG
905	comp36057_c0_seq1.3546.4958.plus.R3_1	GH57	*Prevotella*_sp._CAG
906	comp36233_c0_seq11.81.2063.plus.R3_1	GH13	*Prevotella*_sp._CAG
907	comp37202_c0_seq1.30.1022.minus.R3_1	GH4	*Prevotella*_sp._CAG
908	comp39581_c0_seq1.1.734.minus.R8_1	GH66	*Prevotella*_sp._CAG
909	comp39851_c0_seq1.1.1296.minus.R8_1	GH97	*Prevotella*_sp._CAG
910	comp39862_c0_seq1.1.1225.minus.R3_1	GH13	*Prevotella*_sp._CAG
911	comp46635_c0_seq1.142.1850.minus.R3_1	GH43	*Prevotella*_sp._CAG
912	comp47715_c0_seq1.32.2272.minus.R8_1	GH3	*Prevotella*_sp._CAG
913	comp49149_c0_seq1.1.1905.minus.R8_1	GH3	*Prevotella*_sp._CAG
914	comp50241_c0_seq1.87.1730.minus.R3_1	GH43	*Prevotella*_sp._CAG
915	comp52439_c0_seq1.10.813.plus.R3_1	GH23	*Prevotella*_sp._CAG
916	comp56630_c0_seq1.64.1579.minus.R3_1	GH5	*Prevotella*_sp._CAG
917	comp63425_c0_seq1.1039.3015.plus.R1_1	GH2	*Prevotella*_sp._CAG
918	comp7527_c0_seq1.38.1094.minus.R3_1	GH53	*Prevotella*_sp._CAG
919	comp75901_c0_seq1.30.914.plus.R3_1	GH73	*Prevotella*_sp._CAG
920	comp8888_c0_seq1.62.2452.plus.R3_1	GH2	*Prevotella*_sp._CAG
921	comp9431_c0_seq1.102.1952.plus.R3_1	GH13	*Prevotella*_sp._CAG
922	comp95757_c0_seq1.1.1243.minus.R1_1	GH3	*Prevotella*_sp._CAG
923	comp9849_c0_seq1.18.2633.plus.R3_1	GH95	*Prevotella*_sp._CAG
924	comp9849_c0_seq2.18.2657.plus.R3_1	GH95	*Prevotella*_sp._CAG
925	comp74954_c0_seq1.1480.4062.plus.R1_1	GH78	*Prevotella*_sp._KHD1
926	comp61837_c0_seq4.7.930.plus.R1_1	GH29	*Prolixibacter_bellariivorans*
927	comp108063_c0_seq1.1.746.minus.R9_1	GH77	*Alistipes_finegoldii*
928	comp126961_c0_seq1.1.1074.minus.R3_1	GH110	*Alistipes_finegoldii*
929	comp16091_c0_seq1.65.1774.plus.R1_1	GH123	*Alistipes_finegoldii*
930	comp34380_c0_seq1.60.2348.plus.R3_1	GH20	*Alistipes_finegoldii*
931	comp49049_c0_seq1.26.1106.minus.R1_1	GH77	*Alistipes_finegoldii*
932	comp64982_c0_seq1.63.1730.plus.R3_1	GH2	*Alistipes_finegoldii*
933	comp21998_c0_seq2.48.1364.minus.R3_1	GH29	*Alistipes_finegoldii*_CAG
934	comp36087_c0_seq5.5563.7764.plus.R3_1	GH20	*Alistipes_finegoldii*_CAG
935	comp62249_c0_seq1.11.1979.minus.R3_1	GH97	*Alistipes_finegoldii*_CAG
936	comp9110_c0_seq1.93.1838.minus.R3_1	GH123	*Alistipes_finegoldii*_CAG
937	comp41334_c0_seq1.1154.3145.minus.R8_1	GH133	*Alistipes_inops*
938	comp128102_c0_seq1.1.844.minus.R1_1	GH57	*Alistipes_obesi*
939	comp16153_c0_seq1.177.1513.minus.R1_1	GH2	*Alistipes_obesi*
940	comp180697_c0_seq1.1.806.minus.R1_1	GH23	*Alistipes_obesi*
941	comp34238_c0_seq1.50.1250.minus.R1_1	GH13	*Alistipes_obesi*
942	comp50653_c0_seq1.1.1142.minus.R1_1	GH2	*Alistipes_obesi*
943	comp51337_c0_seq2.33.962.plus.R7_1	GH130	*Alistipes_obesi*

944	comp52606_c0_seq1.65.2056.plus.R7_1	GH2	*Alistipes_obesi*
945	comp60413_c0_seq9.5448.6668.plus.R1_1	GH57	*Alistipes_obesi*
946	comp61152_c0_seq1.4303.5928.plus.R1_1	GH18	*Alistipes_onderdonkii*
947	comp118321_c0_seq1.113.982.plus.R1_1	GH73	*Alistipes_putredinis*
948	comp24675_c0_seq2.1.946.minus.R3_1	GH97	*Alistipes_senegalensis*
949	comp55051_c0_seq2.116.1495.plus.R7_1	GH2	*Alistipes_senegalensis*
950	comp85687_c0_seq1.136.1947.plus.R7_1	GH2	*Alistipes_senegalensis*
951	comp36154_c0_seq6.25.1239.minus.R3_1	GH57	*Alistipes_shahii*
952	comp40064_c0_seq12.76.903.plus.R9_1	GH57	*Alistipes_shahii*
953	comp40064_c0_seq2.76.903.plus.R9_1	GH57	*Alistipes_shahii*
954	comp48034_c0_seq1.727.1962.minus.R8_1	GH88	*Alistipes_shahii*
955	comp54146_c0_seq11.75.1706.minus.R7_1	GH92	*Alistipes_shahii*
956	comp58653_c0_seq1.6.1220.plus.R1_1	GH88	*Alistipes_shahii*
957	comp68826_c0_seq1.729.2660.plus.R7_1	GH97	*Alistipes_shahii*
958	comp27452_c0_seq1.417.1833.minus.R3_1	GH2	*Alistipes*_sp._AL-1
959	comp29021_c0_seq1.54.3140.plus.R1_1	GH2	*Alistipes*_sp._AL-1
960	comp36087_c0_seq11.6143.7807.plus.R3_1	GH18	*Alistipes*_sp._AL-1
961	comp60673_c0_seq1.1.2060.minus.R3_1	GH92	*Alistipes*_sp._AL-1
962	comp61152_c0_seq1.5991.7067.plus.R1_1	GH18	*Alistipes*_sp._AL-1
963	comp72324_c0_seq1.1.984.minus.R3_1	GH18	*Alistipes*_sp._AL-1
964	comp46372_c0_seq1.39.3125.plus.R3_1	GH2	*Alistipes*_sp._AL-1
965	comp104517_c0_seq1.1.3074.minus.R1_1	GH31	*Alistipes*_sp._CAG
966	comp160321_c0_seq1.1.1236.minus.R1_1	GH2	*Alistipes*_sp._CAG
967	comp206841_c0_seq1.1.1020.minus.R1_1	GH2	*Alistipes*_sp._CAG
968	comp20690_c0_seq1.593.1489.plus.R3_1	GH13	*Alistipes*_sp._CAG
969	comp21150_c0_seq1.1.778.minus.R3_1	GH13	*Alistipes*_sp._CAG
970	comp21154_c0_seq2.224.1378.plus.R3_1	GH33	*Alistipes*_sp._CAG
971	comp25873_c0_seq1.4.1422.minus.R1_1	GH109	*Alistipes*_sp._CAG
972	comp25903_c0_seq1.1.1101.minus.R3_1	GH20	*Alistipes*_sp._CAG
973	comp27438_c0_seq1.76.1020.plus.R3_1	GH20	*Alistipes*_sp._CAG
974	comp30148_c0_seq1.1.1714.minus.R3_1	GH31	*Alistipes*_sp._CAG
975	comp32740_c0_seq1.1.2533.minus.R3_1	GH77	*Alistipes*_sp._CAG
976	comp32981_c0_seq1.33.2039.plus.R3_1	GH13	*Alistipes*_sp._CAG
977	comp36425_c0_seq5.71.1069.minus.R3_1	GH4	*Alistipes*_sp._CAG
978	comp37163_c0_seq1.1.1254.minus.R9_1	GH109	*Alistipes*_sp._CAG
979	comp37797_c0_seq2.53.1051.plus.R9_1	GH4	*Alistipes*_sp._CAG
980	comp40064_c0_seq3.16.2106.plus.R9_1	GH133	*Alistipes*_sp._CAG
981	comp40064_c0_seq7.2.1210.plus.R9_1	GH57	*Alistipes*_sp._CAG
982	comp40064_c0_seq8.16.2157.plus.R9_1	GH133	*Alistipes*_sp._CAG
983	comp45369_c0_seq1.30.2393.minus.R3_1	GH92	*Alistipes*_sp._CAG
984	comp47380_c0_seq3.764.2236.minus.R1_1	GH109	*Alistipes*_sp._CAG
985	comp48843_c0_seq4.189.1346.plus.R8_1	GH57	*Alistipes*_sp._CAG
986	comp54730_c0_seq4.10.2011.minus.R7_1	GH20	*Alistipes*_sp._CAG
987	comp54731_c0_seq1.17.1294.plus.R7_1	GH20	*Alistipes*_sp._CAG
988	comp54767_c0_seq2.1.842.minus.R7_1	GH109	*Alistipes*_sp._CAG
989	comp55346_c0_seq2.1.1247.minus.R7_1	GH13	*Alistipes*_sp._CAG
990	comp55346_c0_seq6.1.1637.minus.R7_1	GH13	*Alistipes*_sp._CAG
991	comp55346_c0_seq7.1.1250.minus.R7_1	GH13	*Alistipes*_sp._CAG
992	comp55736_c0_seq2.2939.5037.minus.R7_1	GH133	*Alistipes*_sp._CAG
993	comp55736_c0_seq5.222.952.minus.R7_1	GH57	*Alistipes*_sp._CAG

994	comp56413_c0_seq1.56.1345.plus.R3_1	GH5	*Alistipes*_sp._CAG
995	comp60413_c0_seq9.1983.4091.plus.R1_1	GH133	*Alistipes*_sp._CAG
996	comp61152_c0_seq5.4785.5903.plus.R1_1	GH18	*Alistipes*_sp._CAG
997	comp61626_c0_seq11.793.1770.minus.R1_1	GH133	*Alistipes*_sp._CAG
998	comp61626_c0_seq1.793.2721.minus.R1_1	GH133	*Alistipes*_sp._CAG
999	comp80800_c0_seq1.1.1161.minus.R3_1	GH20	*Alistipes*_sp._CAG
1000	comp87990_c0_seq1.1.1229.minus.R3_1	GH30	*Alistipes*_sp._CAG
1001	comp94810_c0_seq1.169.1329.plus.R1_1	GH2	*Alistipes*_sp._CAG
1002	comp34749_c0_seq2.7.2145.plus.R3_1	GH97	*Alistipes*_sp._CHKCI003
1003	comp27879_c0_seq1.101.1270.plus.R3_1	GH2	*Alistipes*_sp._HGB5
1004	comp38725_c0_seq1.1.827.minus.R7_1	GH125	*Alistipes*_sp._HGB5
1005	comp45854_c0_seq1.34.1660.minus.R3_1	GH29	*Alistipes*_sp._HGB5
1006	comp54232_c0_seq1.5650.7924.minus.R7_1	GH13	*Alistipes*_sp._HGB5
1007	comp58776_c0_seq1.1.1864.minus.R3_1	GH3	*Alistipes*_sp._HGB5
1008	comp68826_c0_seq1.2665.4797.plus.R7_1	GH65	*Alistipes*_sp._HGB5
1009	comp9200_c0_seq1.9.2030.plus.R3_1	GH2	*Alistipes*_sp._HGB5
1010	comp108938_c0_seq1.690.2099.minus.R1_1	GH43	*Alistipes*_sp._Marseille-P2431
1011	comp24675_c0_seq4.1.910.minus.R3_1	GH97	*Alistipes*_sp._Marseille-P2431
1012	comp7208_c0_seq1.1.2127.minus.R3_1	GH2	*Alistipes*_sp._Marseille-P2431
1013	comp76677_c0_seq1.88.1209.plus.R7_1	GH105	*Alistipes*_sp._Marseille-P2431
1014	comp82069_c0_seq1.122.914.minus.R3_1	GH5	*Alistipes*_sp._Marseille-P2431
1015	comp31814_c0_seq1.238.2094.plus.R3_1	GH2	*Alistipes_timonensis*
1016	comp32575_c0_seq2.20.2545.plus.R3_1	GH89	*Alistipes_timonensis*
1017	comp65811_c0_seq1.9.1243.minus.R3_1	GH115	*Alistipes_timonensis*
1018	comp8085_c0_seq1.18.1061.plus.R8_1	GH133	*Alistipes_timonensis*
1019	comp62428_c0_seq1.451.1929.plus.R1_1	GH109	Candidatus_Alistipes_marseilloanorexicus
1020	comp28916_c0_seq1.2695.4302.plus.R3_1	GH18	*Alistipes*_norank
1021	comp36154_c0_seq1.81.1298.minus.R3_1	GH57	*Alistipes*_norank
1022	comp50454_c0_seq1.34.1338.minus.R3_1	GH2	*Alistipes*_norank
1023	comp35456_c0_seq3.1547.4099.plus.R3_1	GH84	*Rikenella_microfusus*
1024	comp142671_c0_seq1.1.795.minus.R1_1	GH29	*Runella_limosa*
1025	comp55492_c0_seq1.1.1943.minus.R3_1	GH95	*Hymenobacter_norwichensis*
1026	comp56842_c0_seq1.1048.3348.minus.R1_1	GH2	*Hymenobacter*_sp._PAMC_26628
1027	comp56842_c0_seq4.17.2347.minus.R1_1	GH2	*Hymenobacter*_sp._PAMC_26628
1028	comp54887_c0_seq1.4.1083.plus.R1_1	GH77	*Persicobacter*_sp._CCB-QB2
1029	comp110402_c0_seq1.306.2174.plus.R1_1	GH20	*Arenibacter_certesii*
1030	comp24521_c0_seq1.1.724.minus.R1_1	GH31	*Flavobacterium*_sp._VMW
1031	comp104165_c0_seq1.157.1278.minus.R1_1	GH99	*Polaribacter_dokdonensis*
1032	comp39291_c0_seq1.1917.3275.plus.R3_1	GH109	*Bacteroidetes*_bacterium_oral_taxon_272
1033	comp29755_c0_seq1.184.1281.plus.R7_1	GH105	*Pedobacter_kyungheensis*
1034	comp38359_c0_seq2.1.1106.minus.R7_1	GH3	*Chloroflexi*_bacterium_OLB15
1035	comp37977_c0_seq1.1.1282.minus.R7_1	GH57	*Mucispirillum_schaedleri*
1036	comp75944_c0_seq1.1.703.minus.R7_1	GH57	*Mucispirillum_schaedleri*
1037	comp54630_c0_seq1.1.1483.minus.R7_1	GH13	*Bacillus_massiliogorillae*
1038	comp34507_c0_seq1.169.4413.plus.R9_1	GH3	*Bacillus_thermoamylovorans*
1039	comp179520_c0_seq1.1.830.minus.R7_1	GH109	*Bacillus*_norank
1040	comp64051_c0_seq1.446.2443.plus.R8_1	GH3	*Bacillus*_norank
1041	comp108018_c0_seq1.1.862.minus.R8_1	GH36	*Paenibacillus_amylolyticus*
1042	comp221085_c0_seq1.1.798.minus.R1_1	GH3	*Paenibacillus_amylolyticus*
1043	comp31749_c0_seq1.1.927.minus.R9_1	GH3	*Paenibacillus_macerans*

1044	comp35420_c0_seq2.1.1186.minus.R8_1	GH43	*Paenibacillus_odorifer*
1045	comp53633_c0_seq1.1.971.minus.R9_1	GH129	*Paenibacillus_oryzae*
1046	comp52921_c0_seq1.1.1120.minus.R9_1	GH32	*Paenibacillus_riograndensis*
1047	comp53719_c0_seq2.35.1558.plus.R7_1	GH43	*Paenibacillus*_sp._DMB5
1048	comp61031_c0_seq1.19.5947.minus.R1_1	GH115	*Paenibacillus*_sp._FSL_R5-0345
1049	comp61031_c0_seq2.19.5926.minus.R1_1	GH115	*Paenibacillus*_sp._FSL_R5-0345
1050	comp46542_c0_seq2.3282.5681.plus.R8_1	GH32	*Paenibacillus*_sp._Soil766
1051	comp49959_c0_seq1.13.846.plus.R7_1	GH109	*Paenibacillus*_sp._UNC451MF
1052	comp31892_c0_seq2.125.3298.minus.R1_1	GH20	*Paenibacillus_terrigena*
1053	comp11609_c0_seq1.1.962.minus.R8_1	GH65	*Enterococcus_faecalis*
1054	comp61607_c0_seq1.14.1303.plus.R1_1	GH65	*Lactobacillus_aviarius*
1055	comp53566_c0_seq1.1.739.minus.R7_1	GH13	*Streptococcus_anginosus*
1056	comp113688_c0_seq1.1.900.minus.R8_1	GH20	*Streptococcus_entericus*
1057	comp49890_c0_seq11.1229.2890.minus.R8_1	GH13	*Streptococcus_suis*
1058	comp49890_c0_seq4.78.1594.minus.R8_1	GH13	*Streptococcus_suis*
1059	comp8055_c0_seq1.23.745.plus.R3_1	GH25	*Caldicoprobacter_oshimai*
1060	comp26391_c0_seq1.2593.4692.minus.R8_1	GH13	*Butyricicoccus_pullicaecorum*
1061	comp33299_c0_seq1.1222.3192.plus.R3_1	GH73	*Butyricicoccus_pullicaecorum*
1062	comp8068_c0_seq1.2.1235.minus.R8_1	GH4	*Butyricicoccus_pullicaecorum*
1063	comp125893_c0_seq1.489.1211.minus.R8_1	GH77	*Clostridium_botulinum*
1064	comp55024_c0_seq1.41.745.plus.R9_1	GH73	*Clostridium_botulinum*
1065	comp49843_c0_seq1.22.3681.minus.R8_1	GH84	*Clostridium_celatum*
1066	comp49904_c0_seq1.83.5758.plus.R7_1	GH2	*Clostridium_celatum*
1067	comp49904_c0_seq1.83.5758.plus.R7_1	GH2	*Clostridium_celatum*
1068	comp35420_c0_seq1.1.823.minus.R8_1	GH43	*Clostridium_cellulovorans*
1069	comp54884_c0_seq1.51.1131.minus.R7_1	GH43	*Clostridium_cellulovorans*
1070	comp11897_c0_seq2.10.3729.plus.R8_1	GH101	*Clostridium_chauvoei*
1071	comp26676_c0_seq1.3.2867.minus.R8_1	GH85	*Clostridium_chauvoei*
1072	comp122847_c0_seq1.487.2430.plus.R1_1	GH120	*Clostridium_hathewayi*_CAG
1073	comp53891_c0_seq1.23.2626.minus.R7_1	GH51	*Clostridium_saccharobutylicum*
1074	comp16019_c0_seq1.4.2232.minus.R1_1	GH36	*Clostridium*_sp._ASF502
1075	comp45223_c0_seq1.114.1181.plus.R7_1	GH77	*Clostridium*_sp._AT5
1076	comp57942_c0_seq1.56.1381.plus.R1_1	GH4	*Clostridium*_sp._AT5
1077	comp49378_c0_seq1.1.1719.minus.R1_1	GH127	*Clostridium*_sp._C105KSO13
1078	comp49644_c0_seq1.58.4206.minus.R8_1	GH127	*Clostridium*_sp._C105KSO13
1079	comp11624_c0_seq1.1.738.minus.R9_1	GH13	*Clostridium*_sp._CAG
1080	comp116579_c0_seq1.1.848.minus.R9_1	GH77	*Clostridium*_sp._CAG
1081	comp31047_c0_seq1.1.762.minus.R9_1	GH57	*Clostridium*_sp._CAG
1082	comp32357_c0_seq1.82.1149.plus.R9_1	GH5	*Clostridium*_sp._CAG
1083	comp39435_c0_seq2.7.1747.minus.R9_1	GH57	*Clostridium*_sp._CAG
1084	comp40798_c0_seq1.640.1692.minus.R1_1	GH109	*Clostridium*_sp._CAG
1085	comp43066_c0_seq1.1.1420.minus.R8_1	GH13	*Clostridium*_sp._CAG
1086	comp54561_c0_seq6.1.1337.minus.R7_1	GH57	*Clostridium*_sp._CAG
1087	comp59595_c0_seq3.37.1155.plus.R1_1	GH109	*Clostridium*_sp._CAG
1088	comp59595_c0_seq4.37.1152.plus.R1_1	GH109	*Clostridium*_sp._CAG
1089	comp60652_c0_seq1.1.1026.minus.R1_1	GH2	*Clostridium*_sp._CAG
1090	comp62249_c0_seq2.35.1699.plus.R1_1	GH26	*Clostridium*_sp._CAG
1091	comp95305_c0_seq1.327.1184.plus.R8_1	GH24	*Clostridium*_sp._CAG
1092	comp97535_c0_seq1.42.1694.plus.R7_1	GH18	*Clostridium*_sp._CAG
1093	comp35317_c0_seq1.89.5629.plus.R3_1	GH33	*Clostridium*_sp._CAG

1094	comp45958_c0_seq1.68.5098.plus.R8_1	GH3	*Clostridium*_sp._CAG
1095	comp46295_c0_seq2.2388.5264.plus.R8_1	GH3	*Clostridium*_sp._CAG
1096	comp54553_c0_seq1.91.3105.plus.R7_1	GH3	*Clostridium*_sp._CAG
1097	comp54314_c0_seq3.1.1434.minus.R7_1	GH2	*Clostridium*_sp._CL-6
1098	comp59121_c0_seq1.8.2236.minus.R1_1	GH2	*Clostridium*_sp._CL-6
1099	comp85763_c0_seq1.88.1644.plus.R1_1	GH13	*Clostridium*_sp._DMHC_10
1100	comp104987_c0_seq1.1.956.minus.R1_1	GH127	*Clostridium*_sp._KLE_1755
1101	comp61964_c0_seq1.701.1540.minus.R1_1	GH109	*Clostridium*_sp._KLE_1755
1102	comp53719_c0_seq1.18.1493.plus.R7_1	GH43	*Clostridium*_sp._Maddingley_MBC34-26
1103	comp12182_c0_seq1.1.710.minus.R1_1	GH36	*Clostridium*_sp._SN17
1104	comp32357_c0_seq1.5182.6906.plus.R9_1	GH127	*Clostridium*_sp._SN17
1105	comp38211_c0_seq2.51.1169.plus.R9_1	GH109	*Clostridium*_sp._SN17
1106	comp38211_c0_seq3.24.1145.plus.R9_1	GH109	*Clostridium*_sp._SN17
1107	comp59516_c0_seq1.1665.2999.plus.R1_1	GH4	*Clostridium*.sp 1
1108	comp166657_c0_seq1.1.729.minus.R1_1	GH43	uncultured_*Clostridium*_sp.
1109	comp44467_c0_seq1.39.1321.minus.R8_1	GH43	uncultured_*Clostridium*_sp.
1110	comp103036_c0_seq1.90.1517.minus.R1_1	GH29	*Hungatella_hathewayi*
1111	comp17286_c0_seq1.19.1381.minus.R1_1	GH31	*Hungatella_hathewayi*
1112	comp83766_c0_seq1.1.858.minus.R8_1	GH3	*Hungatella_hathewayi*
1113	comp46898_c0_seq2.162.4406.plus.R8_1	GH3	*Youngiibacter_fragilis*
1114	comp48553_c0_seq5.1.1119.minus.R8_1	GH13	[*Eubacterium*]_*cellulosolvens*
1115	comp13050_c0_seq1.160.1173.plus.R1_1	GH109	*Eubacterium_desmolans*
1116	comp62576_c0_seq3.14.1039.plus.R1_1	GH109	*Eubacterium_desmolans*
1117	comp123020_c0_seq1.1.1246.minus.R1_1	GH43	*Eubacterium_ramulus*
1118	comp35562_c0_seq1.1.1056.minus.R8_1	GH13	*Eubacterium*_sp._ER2
1119	comp46087_c0_seq1.8.946.plus.R8_1	GH36	*Eubacterium*_sp._ER2
1120	comp46087_c0_seq2.66.1040.plus.R8_1	GH36	*Eubacterium*_sp._ER2
1121	comp60780_c0_seq2.261.1730.plus.R1_1	GH73	uncultured_*Eubacterium*_sp.
1122	comp156792_c0_seq1.1.903.minus.R8_1	GH32	*Eubacteriaceae*_bacterium_CHKCI004
1123	comp43847_c0_seq3.128.2087.minus.R8_1	GH13	*Eubacteriaceae*_bacterium_CHKCI004
1124	comp43847_c0_seq4.128.1370.minus.R8_1	GH13	*Eubacteriaceae*_bacterium_CHKCI004
1125	comp46545_c0_seq1.514.1767.plus.R1_1	GH3	*Eubacteriaceae*_bacterium_CHKCI004
1126	comp48553_c0_seq1.1.1158.minus.R8_1	GH13	*Eubacteriaceae*_bacterium_CHKCI004
1127	comp54236_c0_seq1.483.2663.minus.R1_1	GH36	*Eubacteriaceae*_bacterium_CHKCI004
1128	comp56898_c0_seq1.106.2181.plus.R1_1	GH42	*Eubacteriaceae*_bacterium_CHKCI004
1129	comp58116_c0_seq1.40.1698.minus.R1_1	GH13	*Eubacteriaceae*_bacterium_CHKCI004
1130	comp58303_c0_seq1.60.1667.plus.R1_1	GH77	*Eubacteriaceae*_bacterium_CHKCI004
1131	comp7953_c0_seq1.1.1214.minus.R8_1	GH120	*Eubacteriaceae*_bacterium_CHKCI004
1132	comp126234_c0_seq1.1.701.minus.R1_1	GH20	*Anaerobium_acetethylicum*
1133	comp18379_c0_seq1.671.4975.minus.R8_1	GH20	*Anaerobium_acetethylicum*
1134	comp49019_c1_seq4.14.1024.plus.R8_1	GH109	*Anaerostipes_hadrus*
1135	comp53474_c0_seq1.13.1026.plus.R7_1	GH109	*Anaerostipes*_sp._3_2_56FAA
1136	comp35694_c0_seq1.59.1261.plus.R8_1	GH13	*Blautia_hansenii*
1137	comp58116_c0_seq7.1.1377.minus.R1_1	GH13	*Blautia_hansenii*
1138	comp41569_c0_seq1.113.1798.plus.R8_1	GH112	*Blautia_hydrogenotrophica*
1139	comp49174_c0_seq3.5.1642.plus.R8_1	GH13	*Blautia_hydrogenotrophica*
1140	comp90186_c0_seq1.29.1531.plus.R1_1	GH13	*Blautia_obeum*
1141	comp31162_c0_seq1.1.739.minus.R3_1	GH3	*Blautia_producta*
1142	comp37005_c0_seq1.1.1056.minus.R1_1	GH109	*Blautia_producta*
1143	comp37005_c0_seq2.71.1183.minus.R1_1	GH109	*Blautia_producta*

1144	comp100034_c0_seq1.1.1191.minus.R1_1	GH13	*Blautia_schinkii*
1145	comp124700_c0_seq1.10.1037.minus.R1_1	GH13	*Blautia_schinkii*
1146	comp146070_c0_seq1.1.954.minus.R1_1	GH13	*Blautia_schinkii*
1147	comp45561_c0_seq1.1.1748.minus.R8_1	GH43	*Blautia_schinkii*
1148	comp47940_c0_seq1.21.1874.minus.R8_1	GH13	*Blautia_schinkii*
1149	comp99918_c0_seq1.30.2202.minus.R1_1	GH13	*Blautia_schinkii*
1150	comp39492_c0_seq2.1.743.minus.R9_1	GH36	*Blautia*_sp._CAG
1151	comp39492_c0_seq3.1.1213.minus.R9_1	GH36	*Blautia*_sp._CAG
1152	comp79907_c0_seq1.4.1095.plus.R8_1	GH36	*Blautia*_sp._CAG
1153	comp84290_c0_seq1.1.1754.minus.R1_1	GH13	*Blautia*_sp._CAG
1154	comp135046_c0_seq1.14.826.minus.R1_1	GH32	*Blautia*_sp._Marseille-P2398
1155	comp50857_c0_seq1.1.1969.minus.R1_1	GH43	*Blautia*_sp._YL58
1156	comp128824_c0_seq1.49.1077.plus.R1_1	GH31	*Blautia_wexlerae*
1157	comp155527_c0_seq1.1.836.minus.R1_1	GH77	*Blautia_wexlerae*
1158	comp41895_c0_seq1.1.740.minus.R8_1	GH31	*Blautia_wexlerae*
1159	comp49742_c0_seq1.147.900.minus.R1_1	GH51	*Blautia_wexlerae*
1160	comp37084_c0_seq1.399.1415.plus.R8_1	GH109	uncultured_*Blautia*_sp.
1161	comp72830_c0_seq1.187.3090.plus.R7_1	GH3	*Butyrivibrio_proteoclasticus*
1162	comp55885_c0_seq4.30.2777.plus.R7_1	GH13	*Butyrivibrio*_sp._CAG
1163	comp26639_c0_seq1.302.1493.minus.R9_1	GH36	*Butyrivibrio*_sp._NC3005
1164	comp46388_c0_seq1.716.2113.plus.R8_1	GH4	*Cellulosilyticum_lentocellum*
1165	comp55123_c0_seq2.262.1659.minus.R7_1	GH4	*Cellulosilyticum_lentocellum*
1166	comp49868_c1_seq1.110.5989.plus.R8_1	GH2	*Coprococcus_comes*
1167	comp63178_c0_seq1.404.3529.plus.R1_1	GH105	*Coprococcus_comes*
1168	comp53990_c0_seq1.3796.4908.plus.R1_1	GH53	*Coprococcus_eutactus*
1169	comp59121_c0_seq3.32.2278.minus.R1_1	GH2	*Coprococcus*_sp._HPP0048
1170	comp125434_c0_seq1.1.754.minus.R1_1	GH13	*Eisenbergiella*_tayi
1171	comp13014_c0_seq1.76.1086.plus.R8_1	GH3	*Fusicatenibacter_saccharivorans*
1172	comp47142_c0_seq1.29.1474.minus.R8_1	GH36	[*Clostridium*]_*clostridioforme*
1173	comp136152_c0_seq1.1.1666.minus.R1_1	GH42	[*Clostridium*]_*glycyrrhizinilyticum*
1174	comp49890_c0_seq6.182.888.minus.R8_1	GH13	*Lachnoclostridium_phytofermentans*
1175	comp77391_c0_seq1.1.773.minus.R1_1	GH109	*Marvinbryantia_formatexigens*
1176	comp13507_c0_seq1.86.2320.plus.R8_1	GH31	[*Eubacterium*]_*rectale*
1177	comp26135_c0_seq1.3803.5974.plus.R7_1	GH112	*Lachnospiraceae*_bacterium_2_1_46FAA
1178	comp30006_c0_seq1.70.5400.plus.R9_1	GH101	*Lachnospiraceae*_bacterium_2_1_46FAA
1179	comp60574_c0_seq1.60.5452.minus.R7_1	GH101	*Lachnospiraceae*_bacterium_2_1_46FAA
1180	comp45392_c0_seq1.1.1381.minus.R7_1	GH31	*Lachnospiraceae*_bacterium_2_1_46FAA
1181	comp48674_c0_seq1.20.1189.plus.R1_1	GH27	*Lachnospiraceae*_bacterium_3-2
1182	comp148016_c0_seq1.1.814.minus.R1_1	GH36	*Lachnospiraceae*_bacterium_5_1_63FAA
1183	comp62882_c0_seq2.165.7331.plus.R1_1	GH31	*Lachnospiraceae*_bacterium_6_1_37FAA
1184	comp8825_c0_seq1.27.1550.plus.R7_1	GH33	*Lachnospiraceae*_bacterium_8_1_57FAA
1185	comp26808_c0_seq1.38.4099.plus.R8_1	GH33	*Lachnospiraceae*_bacterium_8_1_57FAA
1186	comp92979_c0_seq1.1433.2455.plus.R7_1	GH109	*Lachnospiraceae*_bacterium_FE2018
1187	comp35440_c0_seq1.1.1054.minus.R8_1	GH36	*Lachnospiraceae*_bacterium_M18-1
1188	comp48452_c0_seq1.25.2052.plus.R8_1	GH42	*Lachnospiraceae*_bacterium_M18-1
1189	comp42405_c0_seq1.1.851.minus.R1_1	GH29	*Lachnospiraceae*_bacterium_mt14
1190	comp26841_c0_seq1.11.2941.plus.R1_1	GH20	*Lachnospiraceae*_norank
1191	comp36472_c0_seq3.49.2790.minus.R3_1	GH20	*Lachnospiraceae*_norank
1192	comp87168_c0_seq1.1.1349.minus.R7_1	GH84	*Lachnospiraceae*_norank
1193	comp49843_c0_seq6.22.4950.minus.R8_1	GH20	*Lachnospiraceae*_norank

1194	comp49880_c0_seq2.347.2036.minus.R8_1	GH13	*Oribacterium*_sp._FC2011
1195	comp59503_c0_seq2.22.1692.plus.R1_1	GH13	*Oribacterium*_sp._P6A1
1196	comp32357_c0_seq1.1200.2894.plus.R9_1	GH2	*Robinsoniella_peoriensis*
1197	comp48884_c0_seq1.3211.5601.plus.R8_1	GH31	*Robinsoniella_peoriensis*
1198	comp62186_c0_seq1.49.2433.minus.R1_1	GH31	*Robinsoniella_peoriensis*
1199	comp15096_c0_seq1.24.755.plus.R7_1	GH13	*Roseburia*_sp._CAG
1200	comp46998_c0_seq1.1.2021.minus.R8_1	GH43	*Roseburia*_sp._CAG
1201	comp46998_c0_seq1.1.2021.minus.R8_1	GH32	*Roseburia*_sp._CAG
1202	comp32691_c0_seq1.1.1168.minus.R3_1	GH33	*Tyzzerella_nexilis*
1203	comp35722_c0_seq1.65.2623.plus.R3_1	GH33	*Tyzzerella_nexilis*
1204	comp18379_c0_seq2.59.3942.minus.R8_1	GH20	*Tyzzerella_nexilis*
1205	comp24341_c0_seq1.171.5702.plus.R7_1	GH43	*Tyzzerella_nexilis*
1206	comp24341_c0_seq1.171.5702.plus.R7_1	GH43	*Tyzzerella_nexilis*
1207	comp48071_c0_seq1.1.5130.minus.R8_1	GH84	*Tyzzerella_nexilis*
1208	comp34483_c0_seq1.1.735.minus.R1_1	GH77	uncultured_*Flavonifractor*_sp.
1209	comp100167_c0_seq1.1.1100.minus.R7_1	GH13	*Clostridiales*_bacterium_CHKCI001
1210	comp169366_c0_seq1.1.818.minus.R7_1	GH3	*Clostridiales*_bacterium_CHKCI001
1211	comp30983_c0_seq2.15.1529.minus.R9_1	GH13	*Clostridiales*_bacterium_CHKCI001
1212	comp34562_c0_seq2.1.1990.minus.R9_1	GH36	*Clostridiales*_bacterium_CHKCI001
1213	comp41685_c0_seq1.3941.6139.plus.R7_1	GH36	*Clostridiales*_bacterium_CHKCI001
1214	comp9040_c0_seq1.1.1420.minus.R7_1	GH32	*Clostridiales*_bacterium_CHKCI001
1215	comp25881_c0_seq1.27.1493.minus.R1_1	GH1	*Clostridiales*_bacterium_CHKCI006
1216	comp42860_c0_seq1.1.1407.minus.R1_1	GH1	*Clostridiales*_bacterium_CHKCI006
1217	comp47296_c0_seq1.1.1403.minus.R1_1	GH1	*Clostridiales*_bacterium_CHKCI006
1218	comp55966_c0_seq2.22.1416.minus.R1_1	GH4	*Clostridiales*_bacterium_CHKCI006
1219	comp79724_c0_seq1.127.2070.minus.R1_1	GH13	*Clostridiales*_bacterium_CHKCI006
1220	comp45467_c0_seq4.188.3991.plus.R8_1	GH3	*Clostridiales*_bacterium_VE202-01
1221	comp53213_c0_seq1.1.1209.minus.R7_1	GH93	*Clostridiales*_bacterium_VE202-27
1222	comp47046_c0_seq1.1.731.minus.R1_1	GH78	*Clostridiales*_norank
1223	comp86857_c0_seq1.43.1380.plus.R8_1	GH31	*Clostridiales*_norank
1224	comp60652_c0_seq3.1.1099.minus.R1_1	GH2	*Clostridiales*_norank
1225	comp29768_c0_seq1.440.2357.minus.R7_1	GH3	*Pseudoflavonifractor_capillosus*
1226	comp42206_c0_seq1.801.1805.minus.R8_1	GH112	*Pseudoflavonifractor_capillosus*
1227	comp52495_c0_seq1.1.904.minus.R7_1	GH112	*Pseudoflavonifractor_capillosus*
1228	comp62991_c0_seq2.126.3158.plus.R1_1	GH3	*Pseudoflavonifractor_capillosus*
1229	comp62991_c0_seq5.162.3170.plus.R1_1	GH3	*Pseudoflavonifractor_capillosus*
1230	comp52198_c0_seq1.1.1243.minus.R7_1	GH13	*Anaerotruncus*_sp._MT15
1231	comp13185_c0_seq1.7.1537.minus.R8_1	GH3	*Soleaferrea_massiliensis*
1232	comp69635_c0_seq1.1.900.minus.R3_1	GH3	*Soleaferrea_massiliensis*
1233	comp48679_c0_seq1.1.2859.minus.R8_1	GH3	*Soleaferrea_massiliensis*
1234	comp11076_c0_seq1.1.771.minus.R1_1	GH1	*Faecalibacterium_prausnitzii*
1235	comp11507_c0_seq1.20.2056.plus.R7_1	GH133	*Faecalibacterium_prausnitzii*
1236	comp118643_c0_seq1.1.792.minus.R1_1	GH32	*Faecalibacterium_prausnitzii*
1237	comp35122_c0_seq2.1.1390.minus.R3_1	GH13	*Faecalibacterium_prausnitzii*
1238	comp35122_c0_seq4.1.1361.minus.R3_1	GH13	*Faecalibacterium_prausnitzii*
1239	comp35713_c0_seq1.1.1339.minus.R1_1	GH2	*Faecalibacterium_prausnitzii*
1240	comp37809_c0_seq1.1002.2480.plus.R3_1	GH109	*Faecalibacterium_prausnitzii*
1241	comp49072_c0_seq1.1.893.minus.R8_1	GH112	*Faecalibacterium_prausnitzii*
1242	comp49072_c0_seq5.1.980.minus.R8_1	GH112	*Faecalibacterium_prausnitzii*
1243	comp49781_c0_seq1.12.857.minus.R8_1	GH109	*Faecalibacterium_prausnitzii*

1244	comp53093_c0_seq1.635.1897.minus.R1_1	GH42	*Faecalibacterium_prausnitzii*
1245	comp55976_c0_seq1.20.1378.plus.R1_1	GH109	*Faecalibacterium_prausnitzii*
1246	comp57600_c0_seq1.26.2437.plus.R1_1	GH2	*Faecalibacterium_prausnitzii*
1247	comp59716_c0_seq1.4.1551.plus.R1_1	GH13	*Faecalibacterium_prausnitzii*
1248	comp60242_c0_seq1.999.2382.minus.R1_1	GH77	*Faecalibacterium_prausnitzii*
1249	comp61195_c0_seq3.7.888.plus.R1_1	GH88	*Faecalibacterium_prausnitzii*
1250	comp61854_c0_seq1.100.1952.minus.R1_1	GH3	*Faecalibacterium_prausnitzii*
1251	comp62282_c0_seq1.203.1585.plus.R1_1	GH25	*Faecalibacterium_prausnitzii*
1252	comp62804_c0_seq1.622.2787.minus.R1_1	GH112	*Faecalibacterium_prausnitzii*
1253	comp62941_c1_seq1.3297.5498.plus.R1_1	GH36	*Faecalibacterium_prausnitzii*
1254	comp62975_c0_seq1.3898.6237.minus.R1_1	GH13	*Faecalibacterium_prausnitzii*
1255	comp58881_c0_seq1.1.1190.minus.R8_1	GH13	*Faecalibacterium*_sp._CAG
1256	comp60731_c0_seq1.1007.1954.plus.R1_1	GH109	*Faecalibacterium*_sp._CAG
1257	comp29369_c0_seq1.26.1222.plus.R8_1	GH109	*Ruminococcaceae*_norank
1258	comp150960_c0_seq1.1.1094.minus.R7_1	GH133	*Ruminococcaceae*_bacterium_AM2
1259	comp47366_c0_seq1.1.863.minus.R7_1	GH13	*Ruminococcaceae*_bacterium_AM2
1260	comp47518_c0_seq1.305.2218.minus.R8_1	GH13	*Ruminococcaceae*_bacterium_AM2
1261	comp49174_c0_seq2.1896.3356.plus.R8_1	GH77	*Ruminococcaceae*_bacterium_AM2
1262	comp49174_c0_seq2.3369.5003.plus.R8_1	GH13	*Ruminococcaceae*_bacterium_AM2
1263	comp59397_c0_seq2.57.1196.plus.R1_1	GH133	*Ruminococcaceae*_bacterium_AM2
1264	comp61104_c0_seq1.2.1630.minus.R1_1	GH13	*Ruminococcaceae*_bacterium_AM2
1265	comp70087_c0_seq1.33.1637.plus.R8_1	GH133	*Ruminococcaceae*_bacterium_AM2
1266	comp81745_c0_seq1.168.1688.plus.R8_1	GH25	*Ruminococcaceae*_bacterium_AM2
1267	comp79339_c0_seq1.15.761.plus.R1_1	GH36	*Ruminococcaceae*_bacterium_D16
1268	comp176819_c0_seq1.1.1003.minus.R1_1	GH84	*Ruminococcaceae*_bacterium_mt9
1269	comp62878_c0_seq8.286.6126.plus.R1_1	GH31	*Ruminococcaceae*_bacterium_mt9
1270	comp47987_c0_seq2.298.2043.plus.R8_1	GH13	[*Clostridium*]_*cellulosi*
1271	comp11663_c0_seq1.36.1028.minus.R9_1	GH2	[*Clostridium*]_*termitidis*
1272	comp58300_c0_seq1.13.1029.plus.R1_1	GH109	*Ruminococcus_gauvreauii*
1273	comp95447_c0_seq1.20.964.plus.R1_1	GH4	*Ruminococcus_gauvreauii*
1274	comp32582_c0_seq1.1.1312.minus.R9_1	GH101	*Ruminococcus_lactaris*
1275	comp53474_c0_seq8.13.1032.plus.R7_1	GH109	*Ruminococcus_lactaris*
1276	comp61284_c0_seq1.1.3655.minus.R8_1	GH84	*Ruminococcus_lactaris*
1277	comp131614_c0_seq1.25.979.minus.R8_1	GH18	*Ruminococcus*_sp._AT10
1278	comp12290_c0_seq1.5.1030.plus.R8_1	GH25	*Ruminococcus*_sp._AT12
1279	comp38581_c0_seq1.14.1111.plus.R7_1	GH13	*Ruminococcus*_sp._CAG
1280	comp12418_c0_seq1.1.964.minus.R8_1	GH77	uncultured_*Ruminococcus*_sp.
1281	comp52229_c0_seq1.48.2051.plus.R8_1	GH13	uncultured_*Ruminococcus*_sp.
1282	comp54247_c0_seq1.21.4337.plus.R8_1	GH84	uncultured_*Ruminococcus*_sp.
1283	comp85607_c0_seq1.7.3960.minus.R1_1	GH29	uncultured_*Ruminococcus*_sp.
1284	comp49072_c0_seq4.1.1840.minus.R8_1	GH112	*Subdoligranulum*_sp._4_3_54A2FAA
1285	comp93961_c0_seq1.20.958.plus.R8_1	GH109	*Subdoligranulum*_sp._4_3_54A2FAA
1286	comp28370_c0_seq1.376.1870.minus.R1_1	GH13	*Subdoligranulum_variabile*
1287	comp34547_c0_seq1.1.750.minus.R1_1	GH42	*Subdoligranulum_variabile*
1288	comp55453_c0_seq1.27.1648.minus.R1_1	GH18	*Clostridia*_bacterium_UC5.1-1D10
1289	comp26524_c0_seq1.1.1086.minus.R3_1	GH84	*Clostridia*_bacterium_UC5.1-1E11
1290	comp58526_c0_seq1.1.1413.minus.R1_1	GH78	*Clostridia*_bacterium_UC5.1-1E11
1291	comp35873_c0_seq1.1351.2661.plus.R3_1	GH23	*Clostridia*_bacterium_UC5.1-2F7
1292	comp55123_c0_seq7.1.957.minus.R7_1	GH4	*Thermoanaerobacterium_aotearoense*
1293	comp46542_c0_seq2.5711.7711.plus.R8_1	GH32	*Thermoanaerobacter_italicus*

1294	comp16744_c0_seq1.8.1489.minus.R1_1	GH1	*Allobaculum_stercoricanis*
1295	comp44466_c0_seq1.82.1020.plus.R8_1	GH25	Candidatus_Stoquefichus_sp._KLE1796
1296	comp44466_c0_seq2.7.1029.plus.R8_1	GH25	Candidatus_Stoquefichus_sp._KLE1796
1297	comp45156_c0_seq1.1.923.minus.R1_1	GH1	Candidatus_Stoquefichus_sp._SB1
1298	comp53095_c0_seq1.30.1847.minus.R9_1	GH2	*Catenibacterium_mitsuokai*
1299	comp49890_c0_seq9.78.781.minus.R8_1	GH13	*Coprobacillus*_sp._CAG
1300	comp57396_c0_seq1.47.2677.minus.R1_1	GH3	*Coprobacillus*_norank
1301	comp71625_c0_seq1.1.1802.minus.R8_1	GH20	[*Clostridium*]_*saccharogumia*
1302	comp49868_c1_seq2.266.5041.plus.R8_1	GH29	[*Clostridium*]_*saccharogumia*
1303	comp128348_c0_seq1.1.791.minus.R1_1	GH20	[*Clostridium*]_*spiroforme*
1304	comp38314_c0_seq1.1.1066.minus.R8_1	GH84	[*Clostridium*]_*spiroforme*
1305	comp60028_c0_seq1.79.2343.plus.R1_1	GH3	[*Clostridium*]_*spiroforme*
1306	comp61967_c0_seq6.168.3662.plus.R1_1	GH3	[*Clostridium*]_*spiroforme*
1307	comp33323_c0_seq1.1.1773.minus.R1_1	GH31	[*Eubacterium*]_*dolichum*
1308	comp15202_c0_seq1.36.1130.plus.R1_1	GH13	*Erysipelotrichaceae*_bacterium_3_1_53
1309	comp55072_c0_seq2.2408.3817.minus.R1_1	GH39	*Erysipelotrichaceae*_bacterium_NK3D112
1310	comp50383_c0_seq1.3.1295.plus.R1_1	GH13	*Erysipelotrichaceae*_norank
1311	comp55966_c0_seq1.1.945.minus.R1_1	GH4	*Erysipelotrichaceae*_norank
1312	comp59824_c0_seq1.1.1425.minus.R1_1	GH1	*Erysipelotrichaceae*_norank
1313	comp59824_c0_seq3.1.815.minus.R1_1	GH1	*Erysipelotrichaceae*_norank
1314	comp44042_c0_seq1.1.1103.minus.R1_1	GH13	*Acidaminococcus*_sp._CAG
1315	comp83124_c0_seq1.43.1566.plus.R7_1	GH20	*Acidaminococcus*_sp._CAG
1316	comp104917_c0_seq1.18.1130.plus.R1_1	GH23	*Megamonas_funiformis*
1317	comp62904_c0_seq1.2620.4632.plus.R1_1	GH13	*Megamonas_funiformis*
1318	comp209417_c0_seq1.1.733.minus.R1_1	GH77	*Megamonas_rupellensis*
1319	comp51167_c0_seq1.1.1124.minus.R1_1	GH13	*Megamonas_rupellensis*
1320	comp61381_c0_seq1.130.1182.plus.R1_1	GH77	*Megamonas_rupellensis*
1321	comp60336_c0_seq1.47.1378.plus.R1_1	GH4	*Megamonas*_norank
1322	comp8736_c0_seq1.1.876.minus.R9_1	GH78	Propionispora.sp 2-37
1323	comp110400_c0_seq1.1.1061.minus.R1_1	GH85	*Firmicutes*_bacterium_CAG
1324	comp15602_c0_seq1.1.1103.minus.R7_1	GH2	*Firmicutes*_bacterium_CAG
1325	comp17017_c0_seq2.10.1076.minus.R1_1	GH4	*Firmicutes*_bacterium_CAG
1326	comp222343_c0_seq1.378.1164.minus.R1_1	GH13	*Firmicutes*_bacterium_CAG
1327	comp30743_c0_seq2.3055.5472.plus.R9_1	GH43	*Firmicutes*_bacterium_CAG
1328	comp32357_c0_seq1.2903.5086.plus.R9_1	GH112	*Firmicutes*_bacterium_CAG
1329	comp34725_c0_seq1.1.950.minus.R8_1	GH42	*Firmicutes*_bacterium_CAG
1330	comp40472_c0_seq1.7293.9809.plus.R9_1	GH31	*Firmicutes*_bacterium_CAG
1331	comp42916_c0_seq1.4.2586.plus.R8_1	GH13	*Firmicutes*_bacterium_CAG
1332	comp48370_c0_seq1.90.2290.minus.R8_1	GH38	*Firmicutes*_bacterium_CAG
1333	comp49816_c0_seq2.4.885.plus.R8_1	GH109	*Firmicutes*_bacterium_CAG
1334	comp50816_c0_seq1.18.1004.minus.R1_1	GH109	*Firmicutes*_bacterium_CAG
1335	comp51101_c0_seq2.29.1196.minus.R1_1	GH109	*Firmicutes*_bacterium_CAG
1336	comp55384_c0_seq2.1.826.minus.R7_1	GH109	*Firmicutes*_bacterium_CAG
1337	comp55384_c0_seq3.383.1501.minus.R7_1	GH109	*Firmicutes*_bacterium_CAG
1338	comp58116_c0_seq3.24.1672.minus.R1_1	GH13	*Firmicutes*_bacterium_CAG
1339	comp58148_c0_seq1.47.2479.plus.R9_1	GH43	*Firmicutes*_bacterium_CAG
1340	comp60567_c0_seq1.1.1623.minus.R1_1	GH13	*Firmicutes*_bacterium_CAG
1341	comp62613_c0_seq1.447.2390.plus.R8_1	GH3	*Firmicutes*_bacterium_CAG
1342	comp70555_c0_seq1.1.946.minus.R9_1	GH42	*Firmicutes*_bacterium_CAG
1343	comp76267_c0_seq1.5.1840.plus.R1_1	GH20	*Helcococcus_kunzii*

1344	comp54561_c0_seq3.31.1792.minus.R7_1	GH57	*Fusobacterium*_sp._CAG
1345	comp54561_c0_seq4.31.1267.minus.R7_1	GH57	*Fusobacterium*_sp._CAG
1346	comp83774_c0_seq1.41.1843.plus.R1_1	GH20	*Fusobacterium* bacterium_SCN_57-13
1347	comp45906_c0_seq1.1.1460.minus.R8_1	GH13	uncultured_bacterium
1348	comp40606_c0_seq1.1.734.minus.R7_1	GH109	uncultured_bacterium_1i11
1349	comp8587_c0_seq1.1.1326.minus.R3_1	GH5	*Planctomyces*_sp._SH-PL62
1350	comp49741_c0_seq1.14.3739.minus.R1_1	GH2	*Rhodopirellula_sallentina*
1351	comp115236_c0_seq1.1.1343.minus.R1_1	GH24	*Hoeflea*_sp._BRH_c9
1352	comp107949_c0_seq1.24.779.minus.R1_1	GH23	*Azospirillum*_sp._CAG
1353	comp52637_c0_seq1.1.1080.minus.R7_1	GH24	*Azospirillum*_sp._CAG
1354	comp57440_c0_seq1.124.1164.plus.R1_1	GH24	*Azospirillum*_sp._CAG
1355	comp91135_c0_seq1.11.1060.minus.R7_1	GH24	*Azospirillum*_sp._CAG
1356	comp52074_c0_seq2.1.1753.minus.R1_1	GH31	*Massilia*_sp._NR_4-1
1357	comp52074_c0_seq1.26.3301.minus.R1_1	GH31	*Massilia*_sp._NR_4-1
1358	comp80711_c0_seq1.18.1070.plus.R1_1	GH109	*Desulfoplanes_formicivorans*
1359	comp44647_c0_seq1.1.1716.minus.R3_1	GH133	*Desulfovibrio_piger*
1360	comp73899_c0_seq1.174.1163.plus.R3_1	GH5	*Cystobacter_fuscus*
1361	comp46029_c0_seq1.2225.4318.plus.R1_1	GH13	*Deferrisoma_camini*
1362	comp144400_c0_seq1.36.913.minus.R1_1	GH20	*Aeromonas_molluscorum*
1363	comp130527_c0_seq1.1.1791.minus.R1_1	GH2	*Anaerobiospirillum_succiniciproducens*
1364	comp42245_c0_seq1.163.3075.plus.R8_1	GH2	*Aliivibrio*_sp._1S128
1365	comp26260_c0_seq1.54.4295.plus.R8_1	GH111	*Treponema_lecithinolyticum*
1366	comp37808_c0_seq1.9.1025.minus.R9_1	GH109	*Cloacibacillus_porcorum*
1367	comp39965_c0_seq1.18.1932.minus.R9_1	GH13	*Cloacibacillus_porcorum*
1368	comp48507_c0_seq1.107.1053.minus.R8_1	GH23	*Synergistes*_sp._3_1_syn1
1369	comp50023_c0_seq1.1076.4362.minus.R8_1	GH57	*Synergistes*_sp._3_1_syn1
1370	comp27730_c0_seq1.1.2541.minus.R8_1	GH3	*Acholeplasma_axanthum*
1371	comp33832_c0_seq1.35.1522.plus.R9_1	GH3	*Acholeplasma_axanthum*
1372	comp40021_c0_seq1.41.1810.plus.R9_1	GH78	*Acholeplasma_axanthum*
1373	comp54126_c0_seq1.197.1606.plus.R1_1	GH32	*Acholeplasma_axanthum*
1374	comp40472_c0_seq2.2086.4065.plus.R9_1	GH43	*Acholeplasma_granularum*
1375	comp40472_c0_seq2.2086.4065.plus.R9_1	GH43	*Acholeplasma_granularum*
1376	comp70895_c0_seq1.11.1468.plus.R1_1	GH33	*Verrucomicrobia*_bacterium_SCGC_AAA168-F10
1377	comp41990_c0_seq1.198.1628.plus.R3_1	GH71	*Diplosphaera_colitermitum*
1378	comp28204_c0_seq1.160.2370.plus.R1_1	GH13	*Opitutaceae*_bacterium_BACL24_MAG-120322-bin51
1379	comp151989_c0_seq1.12.815.minus.R1_1	GH43	*Akkermansia_glycaniphila*
1380	comp10336_c0_seq1.28.2457.plus.R1_1	GH84	*Akkermansia_muciniphila*
1381	comp23576_c0_seq1.47.1573.plus.R1_1	GH89	*Akkermansia_muciniphila*
1382	comp77573_c0_seq1.36.2453.plus.R1_1	GH36	*Akkermansia_muciniphila*
1383	comp98317_c0_seq1.156.887.minus.R1_1	GH16	*Akkermansia_muciniphila*
1384	comp82146_c0_seq1.28.3816.minus.R1_1	GH2	*Akkermansia_muciniphila*
1385	comp79609_c0_seq1.42.1964.plus.R1_1	GH35	*Akkermansia_muciniphila*_CAG
1386	comp56842_c0_seq5.17.2429.minus.R1_1	GH2	*Akkermansia*_sp._CAG
1387	comp56842_c0_seq6.1048.3430.minus.R1_1	GH2	*Akkermansia*_sp._CAG
1388	comp85262_c0_seq1.19.2454.plus.R1_1	GH84	*Akkermansia*_sp._KLE1797
1389	comp98717_c0_seq1.1.1166.minus.R1_1	GH33	*Akkermansia*_sp._KLE1797
1390	comp81711_c0_seq1.1030.2244.minus.R1_1	GH109	*Verrucomicrobium*_sp._BvORR106
1391	comp12669_c0_seq1.1.831.minus.R9_1	GH27	*Capsaspora_owczarzaki*
1392	comp34366_c0_seq1.30.1407.minus.R1_1	GH31	*Capsaspora_owczarzaki*

1393	comp39099_c0_seq1.69.830.minus.R1_1	GH13	*Acytostelium_subglobosum*
1394	comp52287_c0_seq1.43.1228.minus.R7_1	GH13	*Acytostelium_subglobosum*
1395	comp13947_c0_seq2.53.1148.minus.R9_1	GH65	*Dictyostelium_lacteum*
1396	comp40186_c0_seq1.3.782.plus.R7_1	GH5	*Dictyostelium_lacteum*
1397	comp54356_c0_seq1.1.736.minus.R1_1	GH5	*Acanthamoeba_castellanii*
1398	comp13196_c0_seq1.1.1321.minus.R8_1	GH125	*Blastocystis_hominis*
1399	comp20468_c0_seq1.56.1842.minus.R3_1	GH35	*Blastocystis_hominis*
1400	comp26990_c0_seq1.1.773.minus.R9_1	GH31	*Blastocystis_hominis*
1401	comp36203_c0_seq1.1.2882.minus.R3_1	GH38	*Blastocystis_hominis*
1402	comp46399_c0_seq1.1.720.minus.R8_1	GH29	*Blastocystis_hominis*
1403	comp46814_c0_seq1.4.1535.minus.R8_1	GH20	*Blastocystis_hominis*
1404	comp47623_c0_seq2.1.1377.minus.R8_1	GH35	*Blastocystis_hominis*
1405	comp49077_c0_seq1.9.1823.minus.R8_1	GH35	*Blastocystis_hominis*
1406	comp49304_c0_seq1.32.1363.plus.R8_1	GH18	*Blastocystis_hominis*
1407	comp53530_c0_seq1.14.2168.minus.R1_1	GH13	*Blastocystis_hominis*
1408	comp54505_c0_seq1.3.1184.minus.R7_1	GH27	*Blastocystis_hominis*
1409	comp54505_c0_seq2.9.1201.minus.R7_1	GH27	*Blastocystis_hominis*
1410	comp54557_c0_seq1.7.1185.minus.R7_1	GH27	*Blastocystis_hominis*
1411	comp54557_c0_seq1.7.1185.minus.R7_1	GH27	*Blastocystis_hominis*
1412	comp54557_c0_seq2.7.1174.minus.R7_1	GH27	*Blastocystis_hominis*
1413	comp54628_c0_seq2.1.1347.minus.R7_1	GH35	*Blastocystis_hominis*
1414	comp57795_c0_seq1.1.1337.minus.R1_1	GH29	*Blastocystis_hominis*
1415	comp58393_c0_seq1.1.1360.minus.R1_1	GH125	*Blastocystis_hominis*
1416	comp58831_c0_seq1.14.1537.plus.R1_1	GH20	*Blastocystis_hominis*
1417	comp59194_c0_seq1.17.1204.plus.R1_1	GH27	*Blastocystis_hominis*
1418	comp59194_c0_seq1.17.1204.plus.R1_1	GH27	*Blastocystis_hominis*
1419	comp60297_c0_seq1.51.1849.minus.R1_1	GH35	*Blastocystis_hominis*
1420	comp60702_c0_seq1.32.2194.minus.R1_1	GH35	*Blastocystis_hominis*
1421	comp61986_c0_seq1.50.784.plus.R1_1	GH3	*Blastocystis_hominis*
1422	comp61986_c0_seq3.50.2347.plus.R1_1	GH3	*Blastocystis_hominis*
1423	comp74162_c0_seq1.1.889.minus.R8_1	GH3	*Blastocystis_hominis*
1424	comp77872_c0_seq1.1.729.minus.R8_1	GH31	*Blastocystis_hominis*
1425	comp62210_c0_seq1.26.2905.plus.R1_1	GH38	*Blastocystis_hominis*
1426	comp56396_c0_seq1.1.1760.minus.R1_1	GH20	*Blastocystis*_sp._subtype_4
1427	comp58342_c0_seq1.1.2024.minus.R1_1	GH31	*Blastocystis*_sp._subtype_4
1428	comp45475_c0_seq1.114.1820.plus.R7_1	GH20	*Entamoeba_histolytica*
1429	comp46532_c0_seq1.56.1378.plus.R7_1	GH14	*Entamoeba_invadens*
1430	comp118232_c0_seq1.1.1018.minus.R1_1	GH18	*Entamoeba_nuttalli*
1431	comp43770_c0_seq1.1.1709.minus.R1_1	GH38	uncultured_microorganism
1432	comp47147_c0_seq1.1.1625.minus.R1_1	GH47	uncultured_microorganism
1433	comp16180_c0_seq1.1.742.minus.R3_1	GH3	uncultured_microorganism
1434	comp62839_c0_seq1.26.2205.minus.R8_1	GH94	uncultured_microorganism
1435	comp22955_c0_seq1.1.1349.minus.R3_1	GH30	uncultured_microorganism
1436	comp100325_c0_seq1.1.636.minus.R1_1	GH103	unclassified
1437	comp100451_c0_seq1.1.447.minus.R9_1	GH130	unclassified
1438	comp10084_c0_seq1.3089.3772.plus.R9_1	GH25	unclassified
1439	comp100862_c0_seq1.1.697.minus.R7_1	GH109	unclassified
1440	comp100969_c0_seq1.1.284.minus.R3_1	GH109	unclassified
1441	comp101746_c0_seq1.1.392.minus.R8_1	GH109	unclassified
1442	comp102280_c0_seq1.1.427.minus.R8_1	GH13	unclassified

1443	comp102739_c0_seq1.2.418.plus.R1_1	GH130	unclassified
1444	comp102763_c0_seq1.24.689.plus.R7_1	GH25	unclassified
1445	comp102850_c0_seq1.21.460.minus.R8_1	GH47	unclassified
1446	comp103515_c0_seq1.1.606.minus.R3_1	GH43	unclassified
1447	comp104417_c0_seq1.102.668.plus.R8_1	GH43	unclassified
1448	comp104544_c0_seq1.1.408.minus.R3_1	GH28	unclassified
1449	comp104772_c0_seq1.1.355.minus.R1_1	GH33	unclassified
1450	comp104880_c0_seq1.1.502.minus.R1_1	GH25	unclassified
1451	comp106171_c0_seq1.61.693.plus.R7_1	GH51	unclassified
1452	comp10649_c0_seq2.1.486.minus.R8_1	GH73	unclassified
1453	comp106670_c0_seq1.1.471.minus.R8_1	GH109	unclassified
1454	comp106733_c0_seq1.1.367.minus.R7_1	GH29	unclassified
1455	comp106812_c0_seq1.1.488.minus.R1_1	GH43	unclassified
1456	comp107236_c0_seq1.1.475.minus.R8_1	GH43	unclassified
1457	comp107354_c0_seq1.1.627.minus.R8_1	GH43	unclassified
1458	comp107446_c0_seq1.1.245.minus.R1_1	GH109	unclassified
1459	comp107957_c0_seq1.1.662.minus.R9_1	GH78	unclassified
1460	comp108066_c0_seq1.52.640.minus.R9_1	GH123	unclassified
1461	comp108133_c0_seq1.1.601.minus.R3_1	GH110	unclassified
1462	comp108822_c0_seq1.1.335.minus.R8_1	GH109	unclassified
1463	comp108866_c0_seq1.1.322.minus.R3_1	GH80	unclassified
1464	comp109078_c0_seq1.1.563.minus.R1_1	GH76	unclassified
1465	comp109331_c0_seq1.1.498.minus.R1_1	GH84	unclassified
1466	comp109548_c0_seq1.1.509.minus.R7_1	GH105	unclassified
1467	comp109764_c0_seq1.37.420.plus.R3_1	GH43	unclassified
1468	comp110177_c0_seq1.1.471.minus.R7_1	GH43	unclassified
1469	comp110550_c0_seq1.1.383.minus.R1_1	GH43	unclassified
1470	comp111092_c0_seq1.1.572.minus.R1_1	GH125	unclassified
1471	comp111245_c0_seq1.1.424.minus.R1_1	GH20	unclassified
1472	comp111586_c0_seq1.1.433.minus.R3_1	GH57	unclassified
1473	comp11236_c0_seq1.1.412.minus.R8_1	GH109	unclassified
1474	comp113014_c0_seq1.18.269.plus.R9_1	GH25	unclassified
1475	comp113107_c0_seq1.1.437.minus.R8_1	GH29	unclassified
1476	comp113429_c0_seq1.1.393.minus.R8_1	GH57	unclassified
1477	comp11391_c0_seq1.1.132.minus.R9_1	GH109	unclassified
1478	comp114000_c0_seq1.1.587.minus.R1_1	GH3	unclassified
1479	comp114091_c0_seq1.1.541.minus.R8_1	GH25	unclassified
1480	comp114203_c0_seq1.23.427.plus.R9_1	GH16	unclassified
1481	comp1144_c0_seq1.1.513.minus.R9_1	GH43	unclassified
1482	comp114561_c0_seq1.1.569.minus.R3_1	GH28	unclassified
1483	comp115093_c0_seq1.1.351.minus.R3_1	GH29	unclassified
1484	comp115400_c0_seq1.3.678.minus.R7_1	GH97	unclassified
1485	comp115477_c0_seq1.1.466.minus.R1_1	GH47	unclassified
1486	comp115762_c0_seq1.1.352.minus.R3_1	GH23	unclassified
1487	comp116033_c0_seq1.1.577.minus.R7_1	GH133	unclassified
1488	comp116182_c0_seq1.1.419.minus.R3_1	GH105	unclassified
1489	comp116183_c0_seq1.1.309.minus.R3_1	GH43	unclassified
1490	comp116365_c0_seq1.1.344.minus.R1_1	GH13	unclassified
1491	comp117274_c0_seq1.1.393.minus.R7_1	GH3	unclassified
1492	comp117301_c0_seq1.1.694.minus.R8_1	GH89	unclassified

1493	comp117758_c0_seq1.1.446.minus.R1_1	GH3	unclassified
1494	comp117778_c0_seq1.1.400.minus.R8_1	GH73	unclassified
1495	comp117805_c0_seq1.3.554.plus.R8_1	GH78	unclassified
1496	comp118584_c0_seq1.1.462.minus.R3_1	GH105	unclassified
1497	comp118798_c0_seq1.1.356.minus.R3_1	GH33	unclassified
1498	comp119286_c0_seq1.1.386.minus.R1_1	GH109	unclassified
1499	comp119700_c0_seq1.1.676.minus.R8_1	GH20	unclassified
1500	comp119960_c0_seq1.1.568.minus.R7_1	GH13	unclassified
1501	comp120108_c0_seq1.1.645.minus.R7_1	GH13	unclassified
1502	comp120734_c0_seq1.1.333.minus.R1_1	GH109	unclassified
1503	comp121226_c0_seq1.1.480.minus.R3_1	GH43	unclassified
1504	comp121305_c0_seq1.1.322.minus.R3_1	GH4	unclassified
1505	comp12131_c0_seq1.1.656.minus.R8_1	GH36	unclassified
1506	comp121324_c0_seq1.1.673.minus.R1_1	GH85	unclassified
1507	comp122074_c0_seq1.1.531.minus.R1_1	GH29	unclassified
1508	comp122820_c0_seq1.1.500.minus.R9_1	GH13	unclassified
1509	comp123320_c0_seq1.27.322.minus.R9_1	GH43	unclassified
1510	comp123333_c0_seq1.1.668.minus.R1_1	GH13	unclassified
1511	comp123341_c0_seq1.1.388.minus.R1_1	GH109	unclassified
1512	comp123413_c0_seq1.1.470.minus.R1_1	GH25	unclassified
1513	comp123939_c0_seq1.1.402.minus.R3_1	GH13	unclassified
1514	comp124605_c0_seq1.1.469.minus.R1_1	GH125	unclassified
1515	comp126572_c0_seq1.60.312.minus.R1_1	GH25	unclassified
1516	comp127368_c0_seq1.375.935.minus.R1_1	GH92	unclassified
1517	comp127591_c0_seq1.1.627.minus.R9_1	GH92	unclassified
1518	comp127948_c0_seq1.1.527.minus.R7_1	GH43	unclassified
1519	comp127963_c0_seq1.1.353.minus.R9_1	GH16	unclassified
1520	comp129089_c0_seq1.1.650.minus.R3_1	GH36	unclassified
1521	comp129132_c0_seq1.1.531.minus.R9_1	GH127	unclassified
1522	comp129317_c0_seq1.57.611.plus.R1_1	GH43	unclassified
1523	comp129319_c0_seq1.1.363.minus.R3_1	GH130	unclassified
1524	comp129347_c0_seq1.1.669.minus.R1_1	GH16	unclassified
1525	comp129644_c0_seq1.1.614.minus.R8_1	GH20	unclassified
1526	comp129761_c0_seq1.1.360.minus.R9_1	GH3	unclassified
1527	comp130719_c0_seq1.1.506.minus.R1_1	GH31	unclassified
1528	comp131032_c0_seq1.1.262.minus.R9_1	GH109	unclassified
1529	comp131336_c0_seq1.1.568.minus.R1_1	GH77	unclassified
1530	comp131356_c0_seq1.1.466.minus.R7_1	GH117	unclassified
1531	comp131553_c0_seq1.1.374.minus.R9_1	GH23	unclassified
1532	comp131872_c0_seq1.1.390.minus.R7_1	GH13	unclassified
1533	comp132467_c0_seq1.1.571.minus.R3_1	GH20	unclassified
1534	comp132482_c0_seq1.80.508.plus.R1_1	GH43	unclassified
1535	comp132753_c0_seq1.1.491.minus.R8_1	GH13	unclassified
1536	comp132769_c0_seq1.1.379.minus.R7_1	GH109	unclassified
1537	comp132770_c0_seq1.1.556.minus.R8_1	GH16	unclassified
1538	comp133033_c0_seq1.1.472.minus.R1_1	GH32	unclassified
1539	comp133579_c0_seq1.113.362.minus.R1_1	GH24	unclassified
1540	comp133882_c0_seq1.1.470.minus.R1_1	GH13	unclassified
1541	comp134360_c0_seq1.1.333.minus.R7_1	GH84	unclassified
1542	comp134606_c0_seq1.1.574.minus.R1_1	GH109	unclassified

1543	comp13480_c0_seq1.23.787.plus.R1_1	GH23	unclassified
1544	comp13545_c0_seq1.1.446.minus.R3_1	GH13	unclassified
1545	comp135954_c0_seq1.1.463.minus.R8_1	GH13	unclassified
1546	comp136272_c0_seq1.21.581.plus.R1_1	GH88	unclassified
1547	comp136299_c0_seq1.1.370.minus.R1_1	GH32	unclassified
1548	comp136388_c0_seq1.1.397.minus.R8_1	GH13	unclassified
1549	comp137139_c0_seq1.1.500.minus.R8_1	GH16	unclassified
1550	comp137202_c0_seq1.1.535.minus.R7_1	GH3	unclassified
1551	comp13722_c0_seq1.1.426.minus.R7_1	GH88	unclassified
1552	comp13722_c0_seq2.46.552.minus.R7_1	GH88	unclassified
1553	comp137332_c0_seq1.1.314.minus.R9_1	GH105	unclassified
1554	comp137570_c0_seq1.1.497.minus.R1_1	GH28	unclassified
1555	comp137820_c0_seq1.34.408.minus.R8_1	GH16	unclassified
1556	comp137882_c0_seq1.1.323.minus.R1_1	GH13	unclassified
1557	comp137895_c0_seq1.1.420.minus.R9_1	GH43	unclassified
1558	comp138009_c0_seq1.1.311.minus.R1_1	GH109	unclassified
1559	comp138140_c0_seq1.1.521.minus.R3_1	GH109	unclassified
1560	comp138522_c0_seq1.103.772.minus.R8_1	GH78	unclassified
1561	comp138907_c0_seq1.106.570.plus.R8_1	GH10	unclassified
1562	comp139137_c0_seq1.1.560.minus.R8_1	GH92	unclassified
1563	comp139500_c0_seq1.41.490.plus.R1_1	GH43	unclassified
1564	comp139544_c0_seq1.1.540.minus.R8_1	GH3	unclassified
1565	comp139942_c0_seq1.1.453.minus.R7_1	GH3	unclassified
1566	comp14014_c0_seq1.1.362.minus.R1_1	GH4	unclassified
1567	comp14014_c0_seq2.1.362.minus.R1_1	GH4	unclassified
1568	comp14031_c0_seq1.1.565.minus.R1_1	GH4	unclassified
1569	comp140416_c0_seq1.1.407.minus.R1_1	GH84	unclassified
1570	comp14093_c0_seq1.1.487.minus.R3_1	GH3	unclassified
1571	comp141699_c0_seq1.1.455.minus.R7_1	GH25	unclassified
1572	comp142834_c0_seq1.1.333.minus.R9_1	GH3	unclassified
1573	comp142868_c0_seq1.6.540.minus.R7_1	GH30	unclassified
1574	comp142983_c0_seq1.1.529.minus.R7_1	GH88	unclassified
1575	comp143118_c0_seq1.1.566.minus.R7_1	GH30	unclassified
1576	comp143256_c0_seq1.1.316.minus.R8_1	GH109	unclassified
1577	comp143311_c0_seq1.1.339.minus.R1_1	GH16	unclassified
1578	comp143923_c0_seq1.56.494.minus.R8_1	GH133	unclassified
1579	comp14394_c0_seq1.1.417.minus.R9_1	GH3	unclassified
1580	comp144279_c0_seq1.1.446.minus.R8_1	GH125	unclassified
1581	comp144518_c0_seq1.1.450.minus.R3_1	GH43	unclassified
1582	comp14453_c0_seq1.1.389.minus.R9_1	GH85	unclassified
1583	comp144554_c0_seq1.1.437.minus.R1_1	GH33	unclassified
1584	comp145831_c0_seq1.1.471.minus.R1_1	GH85	unclassified
1585	comp145981_c0_seq1.1.533.minus.R8_1	GH92	unclassified
1586	comp146031_c0_seq1.1.337.minus.R8_1	GH109	unclassified
1587	comp146120_c0_seq1.1.698.minus.R3_1	GH43	unclassified
1588	comp146242_c0_seq1.1.480.minus.R3_1	GH3	unclassified
1589	comp146872_c0_seq1.1.673.minus.R7_1	GH89	unclassified
1590	comp147050_c0_seq1.1.369.minus.R3_1	GH102	unclassified
1591	comp147609_c0_seq1.1.464.minus.R1_1	GH109	unclassified
1592	comp150753_c0_seq1.1.451.minus.R7_1	GH43	unclassified

1593	comp151053_c0_seq1.1.393.minus.R8_1	GH13	unclassified
1594	comp151176_c0_seq1.1.532.minus.R8_1	GH32	unclassified
1595	comp151311_c0_seq1.1.674.minus.R1_1	GH112	unclassified
1596	comp151615_c0_seq1.8.400.plus.R1_1	GH43	unclassified
1597	comp151762_c0_seq1.1.677.minus.R1_1	GH78	unclassified
1598	comp151808_c0_seq1.1.410.minus.R3_1	GH35	unclassified
1599	comp151958_c0_seq1.1.465.minus.R1_1	GH13	unclassified
1600	comp152045_c0_seq1.1.395.minus.R1_1	GH3	unclassified
1601	comp15209_c0_seq2.268.807.minus.R1_1	GH20	unclassified
1602	comp152285_c0_seq1.1.324.minus.R1_1	GH3	unclassified
1603	comp152485_c0_seq1.1.506.minus.R8_1	GH85	unclassified
1604	comp152949_c0_seq1.1.588.minus.R1_1	GH92	unclassified
1605	comp153249_c0_seq1.1.466.minus.R3_1	GH43	unclassified
1606	comp153407_c0_seq1.11.631.plus.R1_1	GH125	unclassified
1607	comp154164_c0_seq1.1.471.minus.R8_1	GH43	unclassified
1608	comp154188_c0_seq1.1.323.minus.R8_1	GH13	unclassified
1609	comp154317_c0_seq1.1.417.minus.R8_1	GH20	unclassified
1610	comp154658_c0_seq1.1.557.minus.R7_1	GH43	unclassified
1611	comp155448_c0_seq1.1.360.minus.R1_1	GH84	unclassified
1612	comp155673_c0_seq1.1.501.minus.R7_1	GH31	unclassified
1613	comp155817_c0_seq1.1.418.minus.R9_1	GH3	unclassified
1614	comp15585_c0_seq1.60.590.plus.R1_1	GH33	unclassified
1615	comp156513_c0_seq1.1.342.minus.R1_1	GH105	unclassified
1616	comp156888_c0_seq1.1.454.minus.R7_1	GH29	unclassified
1617	comp157504_c0_seq1.1.354.minus.R8_1	GH18	unclassified
1618	comp157758_c0_seq1.1.486.minus.R7_1	GH28	unclassified
1619	comp157990_c0_seq1.1.630.minus.R1_1	GH31	unclassified
1620	comp158223_c0_seq1.1.480.minus.R1_1	GH10	unclassified
1621	comp160255_c0_seq1.1.418.minus.R1_1	GH13	unclassified
1622	comp160258_c0_seq1.1.341.minus.R1_1	GH13	unclassified
1623	comp160706_c0_seq1.24.573.minus.R1_1	GH5	unclassified
1624	comp160719_c0_seq1.1.372.minus.R8_1	GH29	unclassified
1625	comp160890_c0_seq1.1.441.minus.R1_1	GH23	unclassified
1626	comp162493_c0_seq1.1.393.minus.R1_1	GH109	unclassified
1627	comp162634_c0_seq1.1.311.minus.R8_1	GH3	unclassified
1628	comp162714_c0_seq1.1.555.minus.R1_1	GH3	unclassified
1629	comp162877_c0_seq1.1.692.minus.R1_1	GH13	unclassified
1630	comp163449_c0_seq1.1.545.minus.R1_1	GH13	unclassified
1631	comp163466_c0_seq1.16.395.minus.R9_1	GH25	unclassified
1632	comp163929_c0_seq1.1.405.minus.R8_1	GH31	unclassified
1633	comp164464_c0_seq1.1.591.minus.R7_1	GH77	unclassified
1634	comp164912_c0_seq1.1.656.minus.R1_1	GH31	unclassified
1635	comp165731_c0_seq1.1.558.minus.R8_1	GH3	unclassified
1636	comp166260_c0_seq1.7.338.minus.R8_1	GH25	unclassified
1637	comp166336_c0_seq1.1.528.minus.R1_1	GH43	unclassified
1638	comp166896_c0_seq1.1.425.minus.R1_1	GH29	unclassified
1639	comp167467_c0_seq1.1.380.minus.R8_1	GH16	unclassified
1640	comp168419_c0_seq1.1.473.minus.R9_1	GH84	unclassified
1641	comp168567_c0_seq1.1.316.minus.R7_1	GH20	unclassified
1642	comp168866_c0_seq1.1.448.minus.R8_1	GH105	unclassified

1643	comp169021_c0_seq1.1.375.minus.R1_1	GH109	unclassified
1644	comp169732_c0_seq1.17.610.minus.R7_1	GH20	unclassified
1645	comp170155_c0_seq1.1.559.minus.R1_1	GH109	unclassified
1646	comp170583_c0_seq1.56.614.minus.R1_1	GH92	unclassified
1647	comp17114_c0_seq1.1.604.minus.R1_1	GH43	unclassified
1648	comp172158_c0_seq1.1.591.minus.R1_1	GH78	unclassified
1649	comp172216_c0_seq1.97.645.minus.R1_1	GH16	unclassified
1650	comp172222_c0_seq1.20.295.plus.R7_1	GH25	unclassified
1651	comp17227_c0_seq1.20.403.plus.R9_1	GH3	unclassified
1652	comp173198_c0_seq1.1.383.minus.R8_1	GH4	unclassified
1653	comp174139_c0_seq1.1.457.minus.R8_1	GH32	unclassified
1654	comp174231_c0_seq1.1.566.minus.R1_1	GH31	unclassified
1655	comp174851_c0_seq1.1.434.minus.R1_1	GH13	unclassified
1656	comp17508_c0_seq1.1.360.minus.R3_1	GH13	unclassified
1657	comp17508_c0_seq2.1.350.minus.R3_1	GH13	unclassified
1658	comp17552_c0_seq1.1.462.minus.R7_1	GH82	unclassified
1659	comp176252_c0_seq1.42.536.plus.R9_1	GH43	unclassified
1660	comp176594_c0_seq1.1.434.minus.R1_1	GH31	unclassified
1661	comp176676_c0_seq1.1.320.minus.R1_1	GH18	unclassified
1662	comp177293_c0_seq1.1.497.minus.R7_1	GH33	unclassified
1663	comp177700_c0_seq1.1.388.minus.R1_1	GH65	unclassified
1664	comp177763_c0_seq1.1.636.minus.R1_1	GH57	unclassified
1665	comp177920_c0_seq1.1.423.minus.R7_1	GH13	unclassified
1666	comp178513_c0_seq1.1.390.minus.R8_1	GH25	unclassified
1667	comp178553_c0_seq1.1.365.minus.R7_1	GH133	unclassified
1668	comp17857_c0_seq1.1.543.minus.R7_1	GH5	unclassified
1669	comp17862_c0_seq1.1.433.minus.R7_1	GH43	unclassified
1670	comp178717_c0_seq1.1.538.minus.R8_1	GH10	unclassified
1671	comp178841_c0_seq1.1.522.minus.R8_1	GH31	unclassified
1672	comp179467_c0_seq1.1.398.minus.R7_1	GH3	unclassified
1673	comp18009_c0_seq1.7.621.minus.R1_1	GH5	unclassified
1674	comp180413_c0_seq1.1.302.minus.R3_1	GH32	unclassified
1675	comp181173_c0_seq1.1.478.minus.R8_1	GH43	unclassified
1676	comp181268_c0_seq1.1.371.minus.R7_1	GH38	unclassified
1677	comp18137_c0_seq1.1.531.minus.R8_1	GH13	unclassified
1678	comp18137_c0_seq2.1.466.minus.R8_1	GH13	unclassified
1679	comp181771_c0_seq1.1.401.minus.R7_1	GH109	unclassified
1680	comp181774_c0_seq1.1.331.minus.R8_1	GH43	unclassified
1681	comp181910_c0_seq1.1.538.minus.R7_1	GH3	unclassified
1682	comp183365_c0_seq1.1.375.minus.R8_1	GH109	unclassified
1683	comp18352_c0_seq2.1.616.minus.R3_1	GH73	unclassified
1684	comp18492_c0_seq1.1.468.minus.R7_1	GH29	unclassified
1685	comp184979_c0_seq1.1.443.minus.R1_1	GH10	unclassified
1686	comp185371_c0_seq1.1.385.minus.R1_1	GH20	unclassified
1687	comp185506_c0_seq1.1.470.minus.R8_1	GH31	unclassified
1688	comp185598_c0_seq1.1.337.minus.R1_1	GH3	unclassified
1689	comp185840_c0_seq1.1.535.minus.R1_1	GH20	unclassified
1690	comp186134_c0_seq1.1.397.minus.R1_1	GH3	unclassified
1691	comp186543_c0_seq1.1.551.minus.R3_1	GH133	unclassified
1692	comp18660_c0_seq1.1.437.minus.R3_1	GH130	unclassified

1693	comp186810_c0_seq1.1.293.minus.R7_1	GH109	unclassified
1694	comp187304_c0_seq1.1.408.minus.R1_1	GH23	unclassified
1695	comp187991_c0_seq1.1.512.minus.R1_1	GH73	unclassified
1696	comp188216_c0_seq1.1.497.minus.R7_1	GH31	unclassified
1697	comp188805_c0_seq1.1.449.minus.R1_1	GH20	unclassified
1698	comp189202_c0_seq1.1.361.minus.R1_1	GH3	unclassified
1699	comp189651_c0_seq1.1.380.minus.R7_1	GH73	unclassified
1700	comp189985_c0_seq1.1.509.minus.R1_1	GH85	unclassified
1701	comp190141_c0_seq1.1.337.minus.R1_1	GH43	unclassified
1702	comp190409_c0_seq1.1.448.minus.R1_1	GH84	unclassified
1703	comp190440_c0_seq1.1.502.minus.R1_1	GH31	unclassified
1704	comp190835_c0_seq1.1.281.minus.R8_1	GH23	unclassified
1705	comp193159_c0_seq1.17.353.minus.R1_1	GH43	unclassified
1706	comp193704_c0_seq1.1.523.minus.R7_1	GH92	unclassified
1707	comp195207_c0_seq1.1.491.minus.R8_1	GH109	unclassified
1708	comp195478_c0_seq1.1.361.minus.R7_1	GH43	unclassified
1709	comp196459_c0_seq1.1.357.minus.R7_1	GH43	unclassified
1710	comp196519_c0_seq1.1.385.minus.R7_1	GH3	unclassified
1711	comp197254_c0_seq1.1.374.minus.R8_1	GH43	unclassified
1712	comp19835_c0_seq1.1.536.minus.R8_1	GH13	unclassified
1713	comp198515_c0_seq1.1.379.minus.R7_1	GH38	unclassified
1714	comp19875_c0_seq1.1660.2067.minus.R3_1	GH73	unclassified
1715	comp198942_c0_seq1.1.485.minus.R1_1	GH53	unclassified
1716	comp199399_c0_seq1.1.338.minus.R1_1	GH109	unclassified
1717	comp199834_c0_seq1.1.610.minus.R1_1	GH28	unclassified
1718	comp200550_c0_seq1.1.334.minus.R3_1	GH29	unclassified
1719	comp201490_c0_seq1.1.523.minus.R1_1	GH116	unclassified
1720	comp202113_c0_seq1.1.402.minus.R7_1	GH13	unclassified
1721	comp20215_c0_seq1.1.562.minus.R3_1	GH125	unclassified
1722	comp202399_c0_seq1.1.684.minus.R1_1	GH33	unclassified
1723	comp204688_c0_seq1.1.434.minus.R3_1	GH18	unclassified
1724	comp205633_c0_seq1.1.374.minus.R1_1	GH33	unclassified
1725	comp20622_c0_seq1.1.519.minus.R8_1	GH32	unclassified
1726	comp207038_c0_seq1.1.562.minus.R1_1	GH3	unclassified
1727	comp207094_c0_seq1.1.607.minus.R1_1	GH109	unclassified
1728	comp207107_c0_seq1.1.378.minus.R1_1	GH20	unclassified
1729	comp209041_c0_seq1.3.461.minus.R7_1	GH23	unclassified
1730	comp20908_c0_seq1.1.455.minus.R8_1	GH28	unclassified
1731	comp209634_c0_seq1.1.555.minus.R1_1	GH88	unclassified
1732	comp21021_c0_seq1.1.624.minus.R7_1	GH16	unclassified
1733	comp210605_c0_seq1.1.617.minus.R1_1	GH32	unclassified
1734	comp21135_c0_seq1.1.663.minus.R3_1	GH102	unclassified
1735	comp211535_c0_seq1.1.388.minus.R1_1	GH109	unclassified
1736	comp214675_c0_seq1.1.419.minus.R8_1	GH25	unclassified
1737	comp215270_c0_seq1.1.416.minus.R7_1	GH3	unclassified
1738	comp215414_c0_seq1.1.332.minus.R9_1	GH38	unclassified
1739	comp21555_c0_seq1.1.416.minus.R3_1	GH3	unclassified
1740	comp215730_c0_seq1.1.404.minus.R1_1	GH33	unclassified
1741	comp21665_c0_seq1.1.395.minus.R3_1	GH28	unclassified
1742	comp217115_c0_seq1.1.375.minus.R7_1	GH35	unclassified

1743	comp21806_c0_seq1.95.730.minus.R1_1	GH25	unclassified
1744	comp218447_c0_seq1.1.359.minus.R7_1	GH20	unclassified
1745	comp218669_c0_seq1.1.538.minus.R1_1	GH109	unclassified
1746	comp218870_c0_seq1.1.359.minus.R1_1	GH43	unclassified
1747	comp219400_c0_seq1.12.343.minus.R9_1	GH105	unclassified
1748	comp219851_c0_seq1.1.372.minus.R7_1	GH38	unclassified
1749	comp221762_c0_seq1.1.536.minus.R1_1	GH77	unclassified
1750	comp22208_c0_seq2.1.470.minus.R9_1	GH29	unclassified
1751	comp222679_c0_seq1.1.385.minus.R1_1	GH20	unclassified
1752	comp223918_c0_seq1.1.205.minus.R8_1	GH25	unclassified
1753	comp223949_c0_seq1.1.421.minus.R7_1	GH20	unclassified
1754	comp22501_c0_seq1.1.434.minus.R7_1	GH65	unclassified
1755	comp225216_c0_seq1.1.333.minus.R1_1	GH109	unclassified
1756	comp225626_c0_seq1.67.431.minus.R1_1	GH73	unclassified
1757	comp22752_c0_seq1.1.435.minus.R9_1	GH13	unclassified
1758	comp228252_c0_seq1.1.327.minus.R8_1	GH23	unclassified
1759	comp229331_c0_seq1.1.678.minus.R1_1	GH127	unclassified
1760	comp230552_c0_seq1.1.404.minus.R7_1	GH13	unclassified
1761	comp231351_c0_seq1.52.324.minus.R3_1	GH25	unclassified
1762	comp23145_c0_seq1.1.564.minus.R3_1	GH28	unclassified
1763	comp231539_c0_seq1.1.302.minus.R3_1	GH109	unclassified
1764	comp232133_c0_seq1.1.369.minus.R1_1	GH20	unclassified
1765	comp232160_c0_seq1.30.517.minus.R8_1	GH16	unclassified
1766	comp232342_c0_seq1.1.348.minus.R3_1	GH13	unclassified
1767	comp23257_c0_seq1.1.385.minus.R9_1	GH23	unclassified
1768	comp236158_c0_seq1.1.380.minus.R8_1	GH25	unclassified
1769	comp239244_c0_seq1.1.400.minus.R1_1	GH43	unclassified
1770	comp239787_c0_seq1.1.492.minus.R1_1	GH77	unclassified
1771	comp243445_c0_seq1.1.316.minus.R1_1	GH43	unclassified
1772	comp245725_c0_seq1.1.436.minus.R1_1	GH23	unclassified
1773	comp24626_c0_seq1.1.371.minus.R3_1	GH13	unclassified
1774	comp24675_c0_seq1.1.605.minus.R3_1	GH97	unclassified
1775	comp247409_c0_seq1.1.372.minus.R1_1	GH25	unclassified
1776	comp247842_c0_seq1.1.421.minus.R1_1	GH29	unclassified
1777	comp248136_c0_seq1.1.548.minus.R7_1	GH65	unclassified
1778	comp24909_c0_seq1.1.429.minus.R3_1	GH20	unclassified
1779	comp250555_c0_seq1.1.334.minus.R8_1	GH20	unclassified
1780	comp25061_c0_seq1.1.562.minus.R9_1	GH43	unclassified
1781	comp25069_c0_seq2.1.549.minus.R1_1	GH4	unclassified
1782	comp250953_c0_seq1.1.335.minus.R9_1	GH3	unclassified
1783	comp25172_c0_seq1.1.394.minus.R3_1	GH3	unclassified
1784	comp251908_c0_seq1.1.319.minus.R7_1	GH10	unclassified
1785	comp25284_c0_seq1.1.444.minus.R1_1	GH130	unclassified
1786	comp25591_c0_seq1.77.619.plus.R8_1	GH25	unclassified
1787	comp256723_c0_seq1.1.325.minus.R1_1	GH43	unclassified
1788	comp257492_c0_seq1.1.353.minus.R7_1	GH43	unclassified
1789	comp25841_c0_seq1.96.605.plus.R3_1	GH77	unclassified
1790	comp259641_c0_seq1.1.442.minus.R1_1	GH77	unclassified
1791	comp26008_c0_seq1.1.553.minus.R9_1	GH84	unclassified
1792	comp260291_c0_seq1.1.325.minus.R1_1	GH23	unclassified

1793	comp261071_c0_seq1.1.304.minus.R8_1	GH3	unclassified
1794	comp261103_c0_seq1.1.323.minus.R1_1	GH3	unclassified
1795	comp262328_c0_seq1.1.360.minus.R8_1	GH3	unclassified
1796	comp26340_c0_seq1.1.345.minus.R9_1	GH13	unclassified
1797	comp26379_c0_seq1.74.562.plus.R8_1	GH3	unclassified
1798	comp26480_c0_seq1.1.673.minus.R9_1	GH97	unclassified
1799	comp264810_c0_seq1.1.446.minus.R1_1	GH13	unclassified
1800	comp268403_c0_seq1.1.328.minus.R1_1	GH4	unclassified
1801	comp27049_c0_seq1.127.544.minus.R8_1	GH73	unclassified
1802	comp270619_c0_seq1.1.467.minus.R1_1	GH13	unclassified
1803	comp272360_c0_seq1.1.324.minus.R1_1	GH29	unclassified
1804	comp273845_c0_seq1.1.418.minus.R7_1	GH47	unclassified
1805	comp275743_c0_seq1.1.322.minus.R1_1	GH43	unclassified
1806	comp27689_c0_seq1.1.420.minus.R9_1	GH29	unclassified
1807	comp27723_c0_seq1.1.404.minus.R1_1	GH13	unclassified
1808	comp27723_c0_seq2.1.562.minus.R1_1	GH13	unclassified
1809	comp277371_c0_seq1.1.369.minus.R1_1	GH18	unclassified
1810	comp278130_c0_seq1.1.672.minus.R1_1	GH78	unclassified
1811	comp280351_c0_seq1.1.465.minus.R7_1	GH30	unclassified
1812	comp28108_c0_seq1.14.561.minus.R3_1	GH77	unclassified
1813	comp281403_c0_seq1.1.333.minus.R8_1	GH32	unclassified
1814	comp282449_c0_seq1.1.387.minus.R8_1	GH109	unclassified
1815	comp284854_c0_seq1.1.392.minus.R1_1	GH73	unclassified
1816	comp287263_c0_seq1.1.434.minus.R1_1	GH13	unclassified
1817	comp287870_c0_seq1.1.494.minus.R8_1	GH25	unclassified
1818	comp2902_c0_seq1.1.529.minus.R3_1	GH23	unclassified
1819	comp292295_c0_seq1.1.328.minus.R7_1	GH3	unclassified
1820	comp29344_c0_seq1.1.606.minus.R8_1	GH31	unclassified
1821	comp29380_c0_seq2.1.521.minus.R9_1	GH3	unclassified
1822	comp294858_c0_seq1.1.379.minus.R1_1	GH3	unclassified
1823	comp294970_c0_seq1.1.495.minus.R1_1	GH23	unclassified
1824	comp29636_c0_seq2.1.564.minus.R7_1	GH109	unclassified
1825	comp29679_c0_seq1.1.360.minus.R8_1	GH3	unclassified
1826	comp29733_c0_seq1.1.378.minus.R3_1	GH33	unclassified
1827	comp300737_c0_seq1.1.411.minus.R7_1	GH16	unclassified
1828	comp30266_c0_seq1.1.371.minus.R8_1	GH43	unclassified
1829	comp30322_c0_seq1.1.478.minus.R1_1	GH77	unclassified
1830	comp303849_c0_seq1.1.467.minus.R1_1	GH20	unclassified
1831	comp30395_c0_seq1.1.603.minus.R3_1	GH57	unclassified
1832	comp30570_c0_seq1.1.505.minus.R1_1	GH13	unclassified
1833	comp30641_c0_seq1.1.450.minus.R8_1	GH77	unclassified
1834	comp30874_c0_seq1.27.500.plus.R9_1	GH43	unclassified
1835	comp309496_c0_seq1.1.369.minus.R1_1	GH43	unclassified
1836	comp31063_c0_seq1.1.466.minus.R8_1	GH23	unclassified
1837	comp311000_c0_seq1.1.314.minus.R8_1	GH3	unclassified
1838	comp312488_c0_seq1.1.308.minus.R1_1	GH32	unclassified
1839	comp315717_c0_seq1.1.306.minus.R1_1	GH43	unclassified
1840	comp316000_c0_seq1.1.321.minus.R1_1	GH130	unclassified
1841	comp316767_c0_seq1.1.349.minus.R8_1	GH43	unclassified
1842	comp317226_c0_seq1.1.333.minus.R8_1	GH73	unclassified

1843	comp31787_c0_seq1.1.574.minus.R7_1	GH13	unclassified
1844	comp31793_c0_seq1.17.715.minus.R1_1	GH23	unclassified
1845	comp31888_c0_seq1.11.352.plus.R1_1	GH73	unclassified
1846	comp31888_c0_seq2.16.351.plus.R1_1	GH73	unclassified
1847	comp319677_c0_seq1.1.303.minus.R8_1	GH43	unclassified
1848	comp32343_c0_seq3.1.360.minus.R9_1	GH35	unclassified
1849	comp325151_c0_seq1.1.319.minus.R1_1	GH109	unclassified
1850	comp327156_c0_seq1.1.345.minus.R1_1	GH73	unclassified
1851	comp330134_c0_seq1.1.326.minus.R7_1	GH3	unclassified
1852	comp33194_c0_seq1.1.386.minus.R7_1	GH13	unclassified
1853	comp33731_c0_seq1.1.483.minus.R8_1	GH109	unclassified
1854	comp33832_c0_seq2.37.702.plus.R9_1	GH3	unclassified
1855	comp338602_c0_seq1.1.311.minus.R7_1	GH43	unclassified
1856	comp34233_c0_seq1.1.571.minus.R8_1	GH97	unclassified
1857	comp342853_c0_seq1.1.444.minus.R1_1	GH3	unclassified
1858	comp34388_c0_seq1.32.733.minus.R3_1	GH23	unclassified
1859	comp34388_c0_seq2.1.440.minus.R3_1	GH23	unclassified
1860	comp34492_c0_seq1.1.469.minus.R8_1	GH79	unclassified
1861	comp345149_c0_seq1.1.367.minus.R8_1	GH109	unclassified
1862	comp34669_c0_seq1.375.953.minus.R3_1	GH125	unclassified
1863	comp349869_c0_seq1.1.521.minus.R1_1	GH29	unclassified
1864	comp35143_c0_seq1.1.350.minus.R9_1	GH13	unclassified
1865	comp354599_c0_seq1.1.465.minus.R1_1	GH29	unclassified
1866	comp35580_c0_seq1.1.452.minus.R1_1	GH92	unclassified
1867	comp35753_c0_seq1.1.613.minus.R8_1	GH97	unclassified
1868	comp35957_c0_seq2.1.346.minus.R7_1	GH3	unclassified
1869	comp36137_c0_seq1.1.642.minus.R1_1	GH92	unclassified
1870	comp36302_c0_seq4.1.663.minus.R3_1	GH43	unclassified
1871	comp36502_c0_seq1.564.1283.plus.R3_1	GH23	unclassified
1872	comp36612_c0_seq2.1.314.minus.R3_1	GH109	unclassified
1873	comp36755_c0_seq1.69.712.minus.R8_1	GH43	unclassified
1874	comp36991_c0_seq1.1.495.minus.R8_1	GH13	unclassified
1875	comp37214_c0_seq1.1.396.minus.R1_1	GH3	unclassified
1876	comp38426_c0_seq1.24.423.minus.R7_1	GH53	unclassified
1877	comp38883_c0_seq2.1.430.minus.R9_1	GH31	unclassified
1878	comp39014_c0_seq1.1.679.minus.R8_1	GH20	unclassified
1879	comp39792_c0_seq4.1.551.minus.R9_1	GH109	unclassified
1880	comp40479_c0_seq1.1.579.minus.R7_1	GH77	unclassified
1881	comp406282_c0_seq1.1.357.minus.R7_1	GH109	unclassified
1882	comp40682_c0_seq1.1.347.minus.R1_1	GH109	unclassified
1883	comp407111_c0_seq1.1.291.minus.R1_1	GH73	unclassified
1884	comp40894_c0_seq1.1.316.minus.R8_1	GH120	unclassified
1885	comp41276_c0_seq1.1.542.minus.R8_1	GH29	unclassified
1886	comp414225_c0_seq1.1.394.minus.R1_1	GH24	unclassified
1887	comp41668_c0_seq1.1.620.minus.R8_1	GH57	unclassified
1888	comp42115_c0_seq1.1.378.minus.R7_1	GH20	unclassified
1889	comp42159_c0_seq1.3.458.minus.R1_1	GH8	unclassified
1890	comp42357_c0_seq1.1.669.minus.R1_1	GH13	unclassified
1891	comp42577_c0_seq1.1.416.minus.R8_1	GH43	unclassified
1892	comp42896_c0_seq1.1.457.minus.R9_1	GH57	unclassified

1893	comp430946_c0_seq1.1.303.minus.R8_1	GH43	unclassified
1894	comp43252_c0_seq1.1.654.minus.R7_1	GH88	unclassified
1895	comp43418_c0_seq2.1.619.minus.R7_1	GH92	unclassified
1896	comp43455_c0_seq1.1.350.minus.R8_1	GH23	unclassified
1897	comp434_c0_seq1.1.362.minus.R3_1	GH13	unclassified
1898	comp43570_c0_seq1.1.498.minus.R7_1	GH25	unclassified
1899	comp43818_c0_seq1.1.455.minus.R7_1	GH23	unclassified
1900	comp44183_c0_seq1.38.700.plus.R8_1	GH3	unclassified
1901	comp44210_c0_seq2.1.679.minus.R8_1	GH36	unclassified
1902	comp45072_c0_seq1.1.436.minus.R1_1	GH29	unclassified
1903	comp45156_c0_seq2.1.534.minus.R1_1	GH1	unclassified
1904	comp45362_c0_seq1.61.456.plus.R1_1	GH23	unclassified
1905	comp45534_c0_seq2.1.425.minus.R7_1	GH29	unclassified
1906	comp45548_c0_seq3.1.357.minus.R1_1	GH35	unclassified
1907	comp45558_c0_seq1.146.583.plus.R3_1	GH23	unclassified
1908	comp45830_c0_seq1.1.632.minus.R7_1	GH31	unclassified
1909	comp46652_c0_seq1.427.1119.plus.R3_1	GH23	unclassified
1910	comp47282_c0_seq2.39.722.plus.R8_1	GH3	unclassified
1911	comp47315_c0_seq3.3.677.plus.R8_1	GH77	unclassified
1912	comp47840_c0_seq1.136.501.plus.R9_1	GH43	unclassified
1913	comp48034_c0_seq2.1.671.minus.R8_1	GH88	unclassified
1914	comp4831_c0_seq1.15.331.minus.R1_1	GH73	unclassified
1915	comp48509_c0_seq1.1.561.minus.R1_1	GH20	unclassified
1916	comp48553_c0_seq7.1.661.minus.R8_1	GH13	unclassified
1917	comp48553_c0_seq8.1.382.minus.R8_1	GH13	unclassified
1918	comp48669_c0_seq1.1.604.minus.R1_1	GH109	unclassified
1919	comp48931_c0_seq2.1.502.minus.R7_1	GH13	unclassified
1920	comp48953_c0_seq2.80.736.plus.R7_1	GH23	unclassified
1921	comp49190_c0_seq1.1.635.minus.R7_1	GH13	unclassified
1922	comp49257_c0_seq1.1.635.minus.R8_1	GH3	unclassified
1923	comp49268_c0_seq1.1.672.minus.R8_1	GH31	unclassified
1924	comp49626_c1_seq4.1.303.minus.R8_1	GH4	unclassified
1925	comp49626_c1_seq6.1.360.minus.R8_1	GH4	unclassified
1926	comp49791_c0_seq6.15.713.plus.R8_1	GH33	unclassified
1927	comp49816_c0_seq7.1.255.minus.R8_1	GH109	unclassified
1928	comp49890_c0_seq12.182.779.minus.R8_1	GH13	unclassified
1929	comp49892_c0_seq3.399.881.minus.R8_1	GH73	unclassified
1930	comp49948_c0_seq1.300.966.minus.R1_1	GH27	unclassified
1931	comp50036_c0_seq8.61.729.plus.R8_1	GH3	unclassified
1932	comp50683_c0_seq1.839.1258.plus.R7_1	GH108	unclassified
1933	comp50821_c0_seq1.2043.2663.plus.R7_1	GH25	unclassified
1934	comp50821_c0_seq2.922.1524.plus.R7_1	GH25	unclassified
1935	comp5084_c0_seq1.1.348.minus.R1_1	GH84	unclassified
1936	comp51118_c0_seq1.1.386.minus.R1_1	GH13	unclassified
1937	comp51118_c1_seq1.1.355.minus.R1_1	GH13	unclassified
1938	comp51212_c0_seq1.1.583.minus.R1_1	GH109	unclassified
1939	comp51215_c0_seq1.50.571.plus.R1_1	GH130	unclassified
1940	comp51677_c0_seq1.72.905.plus.R7_1	GH24	unclassified
1941	comp518179_c0_seq1.1.490.minus.R1_1	GH133	unclassified
1942	comp52040_c0_seq1.1834.2346.plus.R7_1	GH73	unclassified

1943	comp52095_c0_seq1.297.986.plus.R1_1	GH105	unclassified
1944	comp52190_c0_seq1.1.408.minus.R1_1	GH130	unclassified
1945	comp52190_c0_seq2.1.414.minus.R1_1	GH130	unclassified
1946	comp52190_c0_seq3.1.363.minus.R1_1	GH130	unclassified
1947	comp534863_c0_seq1.1.363.minus.R7_1	GH33	unclassified
1948	comp54510_c0_seq3.44.511.plus.R1_1	GH73	unclassified
1949	comp54529_c0_seq2.1.612.minus.R7_1	GH16	unclassified
1950	comp54598_c0_seq1.1.382.minus.R1_1	GH130	unclassified
1951	comp54598_c0_seq2.1.686.minus.R1_1	GH130	unclassified
1952	comp5464_c0_seq1.1.444.minus.R3_1	GH20	unclassified
1953	comp54894_c0_seq1.194.815.minus.R1_1	GH23	unclassified
1954	comp54894_c0_seq2.1.597.minus.R1_1	GH23	unclassified
1955	comp54975_c0_seq1.34.2229.plus.R7_1	GH43	unclassified
1956	comp55307_c0_seq18.1.696.minus.R7_1	GH109	unclassified
1957	comp55581_c0_seq3.1.384.minus.R7_1	GH35	unclassified
1958	comp55581_c0_seq4.1.518.minus.R7_1	GH35	unclassified
1959	comp56120_c0_seq1.51.350.plus.R1_1	GH130	unclassified
1960	comp56161_c0_seq1.1.626.minus.R9_1	GH29	unclassified
1961	comp56307_c0_seq1.1.411.minus.R3_1	GH35	unclassified
1962	comp57103_c0_seq1.1.487.minus.R1_1	GH77	unclassified
1963	comp58634_c0_seq1.1.387.minus.R1_1	GH35	unclassified
1964	comp59230_c0_seq2.407.888.minus.R1_1	GH3	unclassified
1965	comp59824_c0_seq2.1.420.minus.R1_1	GH1	unclassified
1966	comp59824_c0_seq6.1.501.minus.R1_1	GH1	unclassified
1967	comp60205_c0_seq6.1.406.minus.R1_1	GH109	unclassified
1968	comp60205_c0_seq7.1.423.minus.R1_1	GH109	unclassified
1969	comp60205_c0_seq8.1.411.minus.R1_1	GH109	unclassified
1970	comp60274_c0_seq3.1.492.minus.R1_1	GH57	unclassified
1971	comp60400_c0_seq1.1.350.minus.R1_1	GH109	unclassified
1972	comp61519_c0_seq1.1.438.minus.R9_1	GH25	unclassified
1973	comp61906_c0_seq1.22.456.plus.R1_1	GH3	unclassified
1974	comp62667_c0_seq2.30.569.minus.R1_1	GH1	unclassified
1975	comp62667_c0_seq4.30.536.minus.R1_1	GH1	unclassified
1976	comp62833_c0_seq1.6.563.minus.R1_1	GH23	unclassified
1977	comp63831_c0_seq1.1171.1788.plus.R1_1	GH108	unclassified
1978	comp64468_c0_seq1.1.564.minus.R7_1	GH4	unclassified
1979	comp64591_c0_seq1.602.1924.plus.R1_1	GH76	unclassified
1980	comp64748_c0_seq1.53.601.plus.R3_1	GH23	unclassified
1981	comp64883_c0_seq1.1.619.minus.R7_1	GH133	unclassified
1982	comp65261_c0_seq1.7.702.plus.R9_1	GH78	unclassified
1983	comp65340_c0_seq1.1.462.minus.R9_1	GH35	unclassified
1984	comp66281_c0_seq1.420.863.minus.R1_1	GH10	unclassified
1985	comp6808_c0_seq1.16.375.plus.R3_1	GH105	unclassified
1986	comp6871_c0_seq1.1.409.minus.R1_1	GH31	unclassified
1987	comp68826_c0_seq1.70.609.plus.R7_1	GH20	unclassified
1988	comp700_c0_seq1.1.401.minus.R1_1	GH42	unclassified
1989	comp70162_c0_seq1.1.488.minus.R3_1	GH23	unclassified
1990	comp72264_c0_seq1.1.494.minus.R9_1	GH13	unclassified
1991	comp72545_c0_seq1.30.383.plus.R3_1	GH28	unclassified
1992	comp73179_c0_seq1.1.318.minus.R3_1	GH4	unclassified

1993	comp73621_c0_seq1.1.628.minus.R1_1	GH109	unclassified
1994	comp75579_c0_seq1.1.370.minus.R9_1	GH5	unclassified
1995	comp76023_c0_seq1.1215.1747.minus.R1_1	GH73	unclassified
1996	comp76227_c0_seq1.1.657.minus.R1_1	GH109	unclassified
1997	comp76282_c0_seq1.1.399.minus.R1_1	GH29	unclassified
1998	comp76338_c0_seq1.1.370.minus.R1_1	GH85	unclassified
1999	comp76349_c0_seq1.1.548.minus.R3_1	GH103	unclassified
2000	comp76677_c0_seq1.1.560.minus.R3_1	GH3	unclassified
2001	comp78921_c0_seq1.1.619.minus.R9_1	GH30	unclassified
2002	comp80291_c0_seq1.1.666.minus.R8_1	GH13	unclassified
2003	comp80707_c0_seq1.1.378.minus.R7_1	GH109	unclassified
2004	comp80759_c0_seq1.1.468.minus.R9_1	GH57	unclassified
2005	comp80776_c0_seq1.1.543.minus.R9_1	GH13	unclassified
2006	comp82093_c0_seq1.3.398.plus.R9_1	GH130	unclassified
2007	comp82854_c0_seq1.1.295.minus.R1_1	GH109	unclassified
2008	comp82908_c0_seq1.1.688.minus.R9_1	GH13	unclassified
2009	comp83535_c0_seq1.1.310.minus.R9_1	GH4	unclassified
2010	comp84191_c0_seq1.1.673.minus.R3_1	GH66	unclassified
2011	comp84463_c0_seq1.1.686.minus.R8_1	GH105	unclassified
2012	comp84564_c0_seq1.1.677.minus.R8_1	GH13	unclassified
2013	comp8511_c0_seq1.9.585.minus.R8_1	GH24	unclassified
2014	comp85786_c0_seq1.1.401.minus.R3_1	GH43	unclassified
2015	comp86243_c0_seq1.84.704.plus.R7_1	GH77	unclassified
2016	comp86255_c0_seq1.1.553.minus.R3_1	GH28	unclassified
2017	comp86322_c0_seq1.5.589.plus.R3_1	GH25	unclassified
2018	comp86430_c0_seq1.1.587.minus.R7_1	GH35	unclassified
2019	comp86725_c0_seq1.1.566.minus.R1_1	GH13	unclassified
2020	comp88170_c0_seq1.1.315.minus.R3_1	GH43	unclassified
2021	comp88348_c0_seq1.1.370.minus.R8_1	GH4	unclassified
2022	comp88627_c0_seq1.1.641.minus.R8_1	GH18	unclassified
2023	comp88839_c0_seq1.1.422.minus.R9_1	GH43	unclassified
2024	comp88919_c0_seq1.1.537.minus.R3_1	GH13	unclassified
2025	comp89249_c0_seq1.1.533.minus.R8_1	GH25	unclassified
2026	comp89726_c0_seq1.1.525.minus.R3_1	GH31	unclassified
2027	comp90406_c0_seq1.1.468.minus.R9_1	GH3	unclassified
2028	comp90501_c0_seq1.1.465.minus.R1_1	GH29	unclassified
2029	comp90938_c0_seq1.1.628.minus.R1_1	GH133	unclassified
2030	comp91005_c0_seq1.54.692.minus.R8_1	GH25	unclassified
2031	comp91020_c0_seq1.1.406.minus.R8_1	GH109	unclassified
2032	comp91949_c0_seq1.28.570.plus.R1_1	GH23	unclassified
2033	comp92212_c0_seq1.1.604.minus.R1_1	GH1	unclassified
2034	comp92453_c0_seq1.1.323.minus.R9_1	GH32	unclassified
2035	comp92847_c0_seq1.1.653.minus.R9_1	GH13	unclassified
2036	comp92961_c0_seq1.363.943.minus.R3_1	GH109	unclassified
2037	comp93272_c0_seq1.1.402.minus.R1_1	GH109	unclassified
2038	comp93405_c0_seq1.1.537.minus.R3_1	GH92	unclassified
2039	comp93792_c0_seq1.1.355.minus.R7_1	GH130	unclassified
2040	comp9391_c0_seq1.1.669.minus.R9_1	GH20	unclassified
2041	comp93938_c0_seq1.1.352.minus.R7_1	GH109	unclassified
2042	comp94766_c0_seq1.1.427.minus.R1_1	GH84	unclassified

2043	comp95190_c0_seq1.1.628.minus.R8_1	GH13	unclassified
2044	comp95666_c0_seq1.1.383.minus.R3_1	GH29	unclassified
2045	comp96331_c0_seq1.1.555.minus.R8_1	GH13	unclassified
2046	comp96511_c0_seq1.1.430.minus.R3_1	GH92	unclassified
2047	comp96873_c0_seq1.1.689.minus.R7_1	GH20	unclassified
2048	comp97275_c0_seq1.1.558.minus.R3_1	GH130	unclassified
2049	comp9731_c0_seq1.1.319.minus.R8_1	GH23	unclassified
2050	comp98901_c0_seq1.93.750.minus.R1_1	GH18	unclassified
2051	comp9891_c0_seq1.1.626.minus.R1_1	GH13	unclassified
2052	comp99009_c0_seq1.1.479.minus.R3_1	GH109	unclassified
2053	comp99065_c0_seq1.1.524.minus.R1_1	GH92	unclassified
2054	comp99193_c0_seq1.1.359.minus.R9_1	GH10	unclassified
