# Supplementary material for: Effects of dietary supplementation with lysozyme on the structure and function of the cecal microbiota in broiler chickens
Source: PLoS One. 2019 Jun 19;14(6):e0216748. doi: 10.1371/journal.pone.0216748 (PMC6583987; doi:10.1371/journal.pone.0216748)
Supplement: S3 Table — (PDF) [file pone.0216748.s003.pdf]

S3 Table. Taxonomy of glycosyltransferase (GT) genes identified in the cecal microbiota of broilers fed a corn-based diet supplemented with 0 (R1 in gene query name), 40 (R7 in gene query name), 100 (R8 in gene query name), or 200 ppm (R9 in gene query name) lysozyme or 400 ppm flavomycin (R3 in gene query name) [the gene names in query refer to those in transcriptome dataset deposited as PRJNA523864 in NCBI Sequence Read Archive].

No	Gene query	Family	Taxonomy
1	comp126116_c0_seq1.66.1004.plus.R1_1	GT2	*Methanocorpusculum_labreanum*
2	comp63797_c0_seq1.28.1698.minus.R1_1	GT83	*Methanocorpusculum_labreanum*
3	comp77543_c0_seq1.63.2658.minus.R8_1	GT66	*Methanocorpusculum_labreanum*
4	comp13451_c0_seq1.838.6486.minus.R1_1	GT66	methanogenic_archaeon_mixed_culture_ISO4-G1
5	comp32830_c0_seq1.995.2679.minus.R3_1	GT3	*Bacteroides_barnesiae*
6	comp48623_c0_seq1.7.1017.plus.R3_1	GT90	*Bacteroides_barnesiae*
7	comp55044_c0_seq2.1264.2535.plus.R7_1	GT4	*Bacteroides_barnesiae*
8	comp55044_c0_seq2.1264.2535.plus.R7_1	GT5	*Bacteroides_barnesiae*
9	comp66796_c0_seq1.371.1281.minus.R8_1	GT8	*Bacteroides_barnesiae*
10	comp7193_c0_seq1.1.808.minus.R9_1	GT8	*Bacteroides_barnesiae*
11	comp98588_c0_seq1.307.1056.plus.R1_1	GT32	*Bacteroides_cellulosilyticus*
12	comp48652_c0_seq10.24.1382.plus.R8_1	GT35	*Bacteroides_clarus*
13	comp16081_c0_seq1.213.1190.plus.R1_1	GT2	*Bacteroides_coprocola*
14	comp55044_c0_seq5.307.1578.plus.R7_1	GT4	*Bacteroides_coprocola*
15	comp55044_c0_seq5.307.1578.plus.R7_1	GT5	*Bacteroides_coprocola*
16	comp57624_c0_seq1.4.1680.plus.R1_1	GT3	*Bacteroides_coprocola*
17	comp57624_c0_seq2.4.1689.plus.R1_1	GT3	*Bacteroides_coprocola*
18	comp31589_c0_seq1.1.816.minus.R3_1	GT35	*Bacteroides_coprocola*_CAG
19	comp39856_c0_seq4.2.1138.plus.R9_1	GT35	*Bacteroides_coprocola*_CAG
20	comp10553_c0_seq1.30.914.plus.R3_1	GT2	*Bacteroides_coprophilus*
21	comp28712_c0_seq1.283.1233.plus.R7_1	GT2	*Bacteroides_coprophilus*
22	comp36301_c0_seq1.271.1392.plus.R3_1	GT4	*Bacteroides_coprophilus*
23	comp47909_c0_seq1.421.1551.plus.R1_1	GT28	*Bacteroides_coprophilus*
24	comp49356_c0_seq1.1576.2832.plus.R8_1	GT4	*Bacteroides_coprophilus*
25	comp55105_c0_seq3.7.1674.plus.R7_1	GT3	*Bacteroides_coprophilus*
26	comp73351_c0_seq1.393.1138.minus.R8_1	GT2	*Bacteroides_coprophilus*
27	comp79214_c0_seq1.30.1151.plus.R1_1	GT4	*Bacteroides_coprophilus*
28	comp88684_c0_seq1.536.1486.plus.R1_1	GT2	*Bacteroides_coprophilus*
29	comp93094_c0_seq1.1.1082.minus.R7_1	GT4	*Bacteroides_coprophilus*
30	comp80822_c0_seq1.12.2352.minus.R7_1	GT51	*Bacteroides_coprophilus*
31	comp114455_c0_seq1.1.1143.minus.R8_1	GT2	*Bacteroides_coprophilus*_CAG
32	comp32447_c0_seq1.18.743.plus.R7_1	GT90	*Bacteroides_coprophilus*_CAG
33	comp46216_c0_seq2.13.840.plus.R7_1	GT5	*Bacteroides_coprophilus*_CAG
34	comp55105_c0_seq2.38.1225.plus.R7_1	GT35	*Bacteroides_coprophilus*_CAG
35	comp88655_c0_seq1.22.972.plus.R7_1	GT2	*Bacteroides_coprophilus*_CAG
36	comp55105_c0_seq3.1715.4285.plus.R7_1	GT35	*Bacteroides_coprophilus*_CAG
37	comp55105_c0_seq9.307.2877.plus.R7_1	GT35	*Bacteroides_coprophilus*_CAG
38	comp75597_c0_seq1.11.913.plus.R7_1	GT4	*Bacteroides_dorei*

39	comp35492_c0_seq2.1802.2746.plus.R3_1	GT2	*Bacteroides_eggerthii*_CAG
40	comp42246_c0_seq1.103.1326.plus.R1_1	GT30	*Bacteroides_faecichinchillae*
41	comp28198_c0_seq1.2891.3895.minus.R3_1	GT4	*Bacteroides_faecis*
42	comp81066_c0_seq1.17.721.plus.R1_1	GT5	*Bacteroides_faecis*
43	comp83283_c0_seq1.460.1524.minus.R1_1	GT9	*Bacteroides_finegoldii*
44	comp101824_c0_seq1.15.806.plus.R1_1	GT2	*Bacteroides_fluxus*
45	comp85517_c0_seq1.1.763.minus.R1_1	GT35	*Bacteroides_fluxus*
46	comp20064_c0_seq1.7182.8405.plus.R9_1	GT4	*Bacteroides_fragilis*
47	comp62272_c0_seq2.2.745.plus.R1_1	GT4	*Bacteroides_fragilis*
48	comp64548_c0_seq1.11042.11815.plus.R1_1	GT2	*Bacteroides_fragilis*
49	comp33095_c0_seq2.1.800.minus.R3_1	GT2	*Bacteroides_gallinarum*
50	comp53889_c0_seq2.2229.3494.plus.R1_1	GT4	*Bacteroides_helcogenes*
51	comp53889_c0_seq2.2229.3494.plus.R1_1	GT5	*Bacteroides_helcogenes*
52	comp26232_c0_seq1.23.766.minus.R3_1	GT92	*Bacteroides_massiliensis*
53	comp84476_c0_seq1.459.1238.minus.R8_1	GT4	*Bacteroides_massiliensis*
54	comp114303_c0_seq1.1.704.minus.R8_1	GT28	*Bacteroides_oleiciplenus*
55	comp81932_c0_seq1.1.783.minus.R1_1	GT4	*Bacteroides_ovatus*
56	comp22884_c0_seq2.1.885.minus.R3_1	GT8	*Bacteroides_plebeius*
57	comp25036_c0_seq1.735.1640.minus.R3_1	GT2	*Bacteroides_plebeius*
58	comp56703_c0_seq1.166.942.plus.R3_1	GT2	*Bacteroides_plebeius*
59	comp61077_c0_seq1.11.1063.plus.R3_1	GT2	*Bacteroides_plebeius*
60	comp94635_c0_seq1.1.706.minus.R1_1	GT2	*Bacteroides_plebeius*
61	comp36057_c0_seq6.2.952.plus.R3_1	GT4	*Bacteroides_plebeius*_CAG
62	comp92163_c0_seq1.264.1314.minus.R1_1	GT28	*Bacteroides_plebeius*_CAG
63	comp32830_c0_seq5.1011.2732.minus.R3_1	GT3	*Bacteroides_salanitronis*
64	comp45985_c0_seq1.825.1607.plus.R3_1	GT31	*Bacteroides_salanitronis*
65	comp58686_c0_seq1.34.942.plus.R1_1	GT13	*Bacteroides_salanitronis*
66	comp74147_c0_seq1.2034.2930.plus.R1_1	GT2	*Bacteroides_salanitronis*
67	comp83639_c0_seq1.39.953.plus.R7_1	GT2	*Bacteroides_salanitronis*
68	comp92248_c0_seq1.20.1156.plus.R7_1	GT4	*Bacteroides_salanitronis*
69	comp62951_c0_seq1.1720.4287.plus.R1_1	GT35	*Bacteroides_salanitronis*
70	comp96451_c0_seq1.1.736.minus.R1_1	GT14	*Bacteroides*_sp._1_1_30
71	comp71744_c0_seq1.1.713.minus.R3_1	GT2	*Bacteroides*_sp._2_1_22
72	comp62890_c0_seq5.2810.3514.plus.R1_1	GT32	*Bacteroides*_sp._3_1_19
73	comp109401_c0_seq1.3.857.minus.R8_1	GT2	*Bacteroides*_sp._CAG
74	comp109595_c0_seq1.1.717.minus.R3_1	GT3	*Bacteroides*_sp._CAG
75	comp128020_c0_seq1.1.713.minus.R1_1	GT2	*Bacteroides*_sp._CAG
76	comp13595_c0_seq1.31.860.minus.R7_1	GT5	*Bacteroides*_sp._CAG
77	comp13931_c0_seq1.1.825.minus.R8_1	GT30	*Bacteroides*_sp._CAG
78	comp15176_c0_seq1.5.1099.plus.R1_1	GT35	*Bacteroides*_sp._CAG
79	comp170854_c0_seq1.1.769.minus.R1_1	GT4	*Bacteroides*_sp._CAG
80	comp17282_c0_seq1.235.1218.minus.R1_1	GT8	*Bacteroides*_sp._CAG
81	comp21525_c0_seq1.545.1300.plus.R1_1	GT4	*Bacteroides*_sp._CAG
82	comp21656_c0_seq1.1543.2766.plus.R1_1	GT30	*Bacteroides*_sp._CAG

83	comp26571_c0_seq3.66.1208.plus.R3_1	GT28	*Bacteroides*_sp._CAG
84	comp30262_c0_seq1.1001.1915.minus.R1_1	GT92	*Bacteroides*_sp._CAG
85	comp31589_c0_seq4.1.820.minus.R3_1	GT35	*Bacteroides*_sp._CAG
86	comp33825_c0_seq1.1.1027.minus.R9_1	GT90	*Bacteroides*_sp._CAG
87	comp35129_c0_seq4.453.1196.minus.R3_1	GT2	*Bacteroides*_sp._CAG
88	comp36057_c0_seq12.2003.3274.plus.R3_1	GT4	*Bacteroides*_sp._CAG
89	comp36057_c0_seq12.2003.3274.plus.R3_1	GT5	*Bacteroides*_sp._CAG
90	comp36057_c0_seq2.2021.3292.plus.R3_1	GT5	*Bacteroides*_sp._CAG
91	comp36057_c0_seq2.2021.3292.plus.R3_1	GT4	*Bacteroides*_sp._CAG
92	comp36057_c0_seq4.2034.3305.plus.R3_1	GT4	*Bacteroides*_sp._CAG
93	comp36057_c0_seq4.2034.3305.plus.R3_1	GT5	*Bacteroides*_sp._CAG
94	comp36331_c0_seq2.15.839.plus.R3_1	GT5	*Bacteroides*_sp._CAG
95	comp36456_c0_seq1.2.778.plus.R9_1	GT4	*Bacteroides*_sp._CAG
96	comp36463_c1_seq3.79.1128.plus.R3_1	GT28	*Bacteroides*_sp._CAG
97	comp46216_c0_seq1.13.834.plus.R7_1	GT5	*Bacteroides*_sp._CAG
98	comp48311_c0_seq1.17.907.plus.R3_1	GT14	*Bacteroides*_sp._CAG
99	comp48703_c0_seq1.122.1078.minus.R1_1	GT2	*Bacteroides*_sp._CAG
100	comp54470_c0_seq1.1744.2883.plus.R7_1	GT4	*Bacteroides*_sp._CAG
101	comp55044_c0_seq7.1254.2522.plus.R7_1	GT5	*Bacteroides*_sp._CAG
102	comp55044_c0_seq7.1254.2522.plus.R7_1	GT4	*Bacteroides*_sp._CAG
103	comp56876_c0_seq3.1856.3577.minus.R1_1	GT83	*Bacteroides*_sp._CAG
104	comp57255_c0_seq1.235.1107.minus.R1_1	GT92	*Bacteroides*_sp._CAG
105	comp58182_c0_seq1.113.1330.plus.R3_1	GT30	*Bacteroides*_sp._CAG
106	comp58882_c0_seq1.1.1062.minus.R3_1	GT30	*Bacteroides*_sp._CAG
107	comp60413_c0_seq3.121.1383.plus.R1_1	GT4	*Bacteroides*_sp._CAG
108	comp60413_c0_seq3.121.1383.plus.R1_1	GT5	*Bacteroides*_sp._CAG
109	comp60861_c0_seq1.806.1882.minus.R1_1	GT51	*Bacteroides*_sp._CAG
110	comp60966_c0_seq3.10.1152.minus.R1_1	GT19	*Bacteroides*_sp._CAG
111	comp61821_c0_seq19.53.1322.minus.R1_1	GT5	*Bacteroides*_sp._CAG
112	comp61821_c0_seq19.53.1322.minus.R1_1	GT4	*Bacteroides*_sp._CAG
113	comp62027_c0_seq2.3.824.plus.R1_1	GT5	*Bacteroides*_sp._CAG
114	comp62248_c0_seq5.124.991.minus.R1_1	GT2	*Bacteroides*_sp._CAG
115	comp62477_c0_seq4.45.989.plus.R1_1	GT2	*Bacteroides*_sp._CAG
116	comp62913_c0_seq5.3823.4578.plus.R1_1	GT26	*Bacteroides*_sp._CAG
117	comp62918_c0_seq7.267.1454.plus.R1_1	GT2	*Bacteroides*_sp._CAG
118	comp62970_c1_seq1.7801.9468.plus.R1_1	GT3	*Bacteroides*_sp._CAG
119	comp66557_c0_seq1.682.1575.plus.R1_1	GT2	*Bacteroides*_sp._CAG
120	comp6753_c0_seq1.7.1137.plus.R3_1	GT28	*Bacteroides*_sp._CAG
121	comp67645_c0_seq1.1827.3749.minus.R1_1	GT51	*Bacteroides*_sp._CAG
122	comp71144_c0_seq1.314.1219.minus.R3_1	GT4	*Bacteroides*_sp._CAG
123	comp72483_c0_seq1.576.1526.plus.R1_1	GT2	*Bacteroides*_sp._CAG
124	comp73494_c0_seq1.18.1682.plus.R1_1	GT3	*Bacteroides*_sp._CAG
125	comp80863_c0_seq1.565.1536.plus.R7_1	GT2	*Bacteroides*_sp._CAG
126	comp86100_c0_seq1.8.1270.plus.R1_1	GT4	*Bacteroides*_sp._CAG

127	comp99958_c0_seq1.138.1295.plus.R1_1	GT2	*Bacteroides*_sp._CAG
128	comp20042_c0_seq2.2050.4620.minus.R3_1	GT35	*Bacteroides*_sp._CAG
129	comp38883_c0_seq1.1760.4101.minus.R9_1	GT44	*Bacteroides*_sp._CAG
130	comp39856_c0_seq7.10.2070.plus.R9_1	GT35	*Bacteroides*_sp._CAG
131	comp62963_c0_seq2.13265.15661.plus.R1_1	GT2	*Bacteroides*_sp._CAG
132	comp78732_c0_seq1.47.2005.minus.R1_1	GT51	*Bacteroides*_sp._CAG
133	comp85964_c0_seq1.1.748.minus.R1_1	GT2	*Bacteroides*_sp._D22
134	comp35546_c0_seq1.129.1268.plus.R3_1	GT19	*Bacteroides_stercoris*_CAG
135	comp76458_c0_seq1.64.1209.minus.R3_1	GT19	*Bacteroides_stercoris*_CAG
136	comp66557_c0_seq1.3599.4660.plus.R1_1	GT2	*Bacteroides_thetaiotaomicron*
137	comp62831_c0_seq8.1846.2883.minus.R1_1	GT2	*Bacteroides_thetaiotaomicron*_CAG
138	comp57617_c0_seq1.1.876.minus.R3_1	GT2	*Bacteroides_vulgatus*
139	comp94403_c0_seq1.815.1946.minus.R1_1	GT4	*Bacteroides_vulgatus*
140	comp103200_c0_seq1.320.1195.minus.R1_1	GT4	*Bacteroides_xylanisolvens*
141	comp89013_c0_seq1.129.1100.plus.R1_1	GT2	*Bacteroides_xylanisolvens*
142	comp10181_c0_seq1.2293.3195.plus.R9_1	GT13	Candidatus_Bacteroides_timonensis
143	comp11874_c0_seq2.480.1212.minus.R1_1	GT2	Candidatus_Bacteroides_timonensis
144	comp133490_c0_seq1.185.1018.plus.R1_1	GT2	*Bacteroides*_norank
145	comp26829_c0_seq1.1.735.minus.R7_1	GT4	*Bacteroides*_norank
146	comp46987_c0_seq1.7.825.plus.R3_1	GT5	*Bacteroides*_norank
147	comp54120_c0_seq1.1242.2429.plus.R9_1	GT4	*Bacteroides*_norank
148	comp83324_c0_seq1.6141.7175.plus.R1_1	GT4	*Bacteroides*_norank
149	comp83324_c0_seq1.7239.8204.plus.R1_1	GT2	*Bacteroides*_norank
150	comp92318_c0_seq1.82.1050.minus.R1_1	GT2	*Bacteroides*_norank
151	comp100726_c0_seq1.10.936.plus.R1_1	GT8	uncultured_*Bacteroides*_sp.
152	comp36456_c0_seq4.2.1270.plus.R9_1	GT5	uncultured_*Bacteroides*_sp.
153	comp36456_c0_seq4.2.1270.plus.R9_1	GT4	uncultured_*Bacteroides*_sp.
154	comp37228_c0_seq4.2.1258.plus.R9_1	GT5	uncultured_*Bacteroides*_sp.
155	comp37228_c0_seq4.2.1258.plus.R9_1	GT4	uncultured_*Bacteroides*_sp.
156	comp55895_c0_seq1.1.957.minus.R9_1	GT3	uncultured_*Bacteroides*_sp.
157	comp80058_c0_seq1.1.902.minus.R7_1	GT2	uncultured_*Bacteroides*_sp.
158	comp77585_c0_seq1.50.2276.minus.R1_1	GT35	uncultured_*Bacteroides*_sp.
159	comp62477_c0_seq3.365.1930.plus.R1_1	GT83	*Bacteroidales*_bacterium_Barb4
160	comp23994_c0_seq1.498.1679.minus.R3_1	GT4	*Bacteroidales*_norank
161	comp35485_c0_seq1.41.793.plus.R3_1	GT2	*Bacteroidales*_norank
162	comp61806_c0_seq1.7.1662.plus.R1_1	GT3	*Bacteroidales*_norank
163	comp78008_c0_seq1.22.2367.plus.R7_1	GT51	*Bacteroidales*_norank
164	comp128637_c0_seq1.7.822.plus.R7_1	GT5	*Barnesiella_intestinihominis*
165	comp60413_c0_seq5.34.1251.plus.R1_1	GT4	*Barnesiella_viscericola*
166	comp60413_c0_seq5.34.1251.plus.R1_1	GT5	*Barnesiella_viscericola*
167	comp78593_c0_seq1.891.2546.minus.R1_1	GT3	*Coprobacter_secundus*
168	comp82607_c0_seq1.409.1170.minus.R7_1	GT4	*Dysgonomonas_gadei*
169	comp39963_c0_seq1.24.851.plus.R9_1	GT4	*Parabacteroides*_norank
170	comp62257_c0_seq3.2001.3269.plus.R1_1	GT5	*Parabacteroides*_norank

171	comp62257_c0_seq3.2001.3269.plus.R1_1	GT4	*Parabacteroides*_norank
172	comp62890_c0_seq5.1158.2804.plus.R1_1	GT32	*Parabacteroides_distasonis*
173	comp81092_c0_seq1.614.1621.minus.R8_1	GT2	*Parabacteroides_distasonis*
174	comp61806_c0_seq1.1778.4348.plus.R1_1	GT35	*Parabacteroides_distasonis*
175	comp36523_c0_seq4.1311.2393.plus.R3_1	GT4	*Parabacteroides_goldsteinii*
176	comp61683_c0_seq1.124.1149.minus.R1_1	GT9	*Parabacteroides_goldsteinii*
177	comp64548_c0_seq1.7779.8741.plus.R1_1	GT2	*Parabacteroides_goldsteinii*
178	comp32654_c0_seq1.65.835.plus.R7_1	GT2	*Parabacteroides_johnsonii*
179	comp61183_c0_seq2.641.1594.plus.R1_1	GT2	*Parabacteroides_johnsonii*
180	comp61806_c0_seq2.8.1678.plus.R1_1	GT3	*Parabacteroides*_sp._20_3
181	comp10484_c0_seq1.1556.2803.plus.R1_1	GT30	*Parabacteroides*_sp._CAG
182	comp57624_c0_seq3.111.1661.plus.R1_1	GT3	*Parabacteroides*_sp._CAG
183	comp72290_c0_seq1.197.962.minus.R8_1	GT2	*Parabacteroides*_sp._CAG
184	comp93413_c0_seq1.23.739.plus.R1_1	GT28	*Parabacteroides*_sp._CAG
185	comp118208_c0_seq1.1153.2331.plus.R1_1	GT4	*Proteiniphilum_acetatigenes*
186	comp74468_c0_seq1.44.1469.minus.R1_1	GT35	*Tannerella*_sp._6_1_58FAA_CT1
187	comp77308_c0_seq1.35.958.plus.R1_1	GT5	*Tannerella*_sp._CAG
188	comp83639_c0_seq1.995.1732.plus.R7_1	GT2	*Tannerella*_sp._CAG
189	comp96921_c0_seq1.152.1099.plus.R1_1	GT2	*Tannerella*_sp._CAG
190	comp35781_c1_seq17.3185.4216.plus.R3_1	GT94	*Paraprevotella_clara*_CAG
191	comp45968_c0_seq2.1.737.minus.R7_1	GT45	*Prevotella_copri*
192	comp65780_c0_seq1.995.1924.plus.R3_1	GT8	*Prevotella_copri*_CAG
193	comp30320_c0_seq3.53.763.plus.R3_1	GT2	*Prevotella_corporis*
194	comp21525_c0_seq1.1359.2453.plus.R1_1	GT4	*Prevotella_maculosa*
195	comp90295_c0_seq1.80.982.plus.R7_1	GT11	*Prevotella*_sp._109
196	comp12750_c0_seq1.843.1832.plus.R3_1	GT2	*Prevotella*_sp._CAG
197	comp13663_c0_seq1.4392.5444.plus.R1_1	GT2	*Prevotella*_sp._CAG
198	comp17385_c0_seq1.28.771.plus.R3_1	GT2	*Prevotella*_sp._CAG
199	comp27359_c0_seq1.517.1485.minus.R3_1	GT2	*Prevotella*_sp._CAG
200	comp35781_c1_seq17.5118.6251.plus.R3_1	GT4	*Prevotella*_sp._CAG
201	comp35985_c0_seq8.151.1311.plus.R3_1	GT4	*Prevotella*_sp._CAG
202	comp36057_c0_seq1.2233.3507.plus.R3_1	GT5	*Prevotella*_sp._CAG
203	comp36057_c0_seq1.2233.3507.plus.R3_1	GT4	*Prevotella*_sp._CAG
204	comp40578_c0_seq1.81.1664.plus.R3_1	GT3	*Prevotella*_sp._CAG
205	comp40584_c0_seq1.177.1217.plus.R3_1	GT9	*Prevotella*_sp._CAG
206	comp51180_c0_seq1.2720.3859.plus.R3_1	GT4	*Prevotella*_sp._CAG
207	comp56600_c0_seq1.303.1580.minus.R3_1	GT4	*Prevotella*_sp._CAG
208	comp58570_c0_seq1.1.929.minus.R3_1	GT2	*Prevotella*_sp._CAG
209	comp61615_c0_seq9.335.1126.plus.R1_1	GT2	*Prevotella*_sp._CAG
210	comp64849_c0_seq1.316.1476.minus.R3_1	GT19	*Prevotella*_sp._CAG
211	comp67435_c0_seq1.38.781.plus.R3_1	GT51	*Prevotella*_sp._CAG
212	comp6952_c0_seq1.401.1390.plus.R3_1	GT2	*Prevotella*_sp._CAG
213	comp75692_c0_seq1.34.948.plus.R8_1	GT2	*Prevotella*_sp._CAG
214	comp34653_c0_seq1.4212.6746.plus.R3_1	GT51	*Prevotella*_sp._CAG

215	comp40578_c0_seq1.1742.4297.plus.R3_1	GT35	*Prevotella*_sp._CAG
216	comp32293_c0_seq1.186.1100.plus.R1_1	GT8	*Prevotella*_sp._KHD1
217	comp97675_c0_seq1.198.1059.minus.R3_1	GT2	*Prevotella_timonensis*
218	comp35781_c1_seq20.2153.3097.plus.R3_1	GT2	*Draconibacterium_sediminis*
219	comp14967_c0_seq1.384.1530.minus.R1_1	GT21	*Alistipes_finegoldii*
220	comp35985_c0_seq8.2536.3621.plus.R3_1	GT4	*Alistipes_finegoldii*_CAG
221	comp14865_c0_seq1.40.1125.minus.R1_1	GT9	*Alistipes_obesi*
222	comp35048_c0_seq1.713.1840.plus.R3_1	GT9	*Alistipes_obesi*
223	comp34449_c0_seq4.817.3244.minus.R3_1	GT51	*Alistipes_onderdonkii*
224	comp187883_c0_seq1.1.777.minus.R1_1	GT51	*Alistipes_putredinis*
225	comp114514_c0_seq1.1.869.minus.R1_1	GT2	*Alistipes_putredinis*_CAG
226	comp143279_c0_seq1.1.739.minus.R1_1	GT21	*Alistipes_putredinis*_CAG
227	comp27422_c0_seq2.1.1104.minus.R3_1	GT4	*Alistipes_senegalensis*
228	comp27422_c0_seq3.73.1218.minus.R3_1	GT4	*Alistipes_senegalensis*
229	comp44896_c0_seq1.21.749.plus.R7_1	GT2	*Alistipes_senegalensis*
230	comp44896_c0_seq2.20.748.plus.R7_1	GT2	*Alistipes_senegalensis*
231	comp88489_c0_seq1.1.1082.minus.R7_1	GT4	*Alistipes_senegalensis*
232	comp24996_c0_seq1.684.1827.minus.R3_1	GT2	*Alistipes_shahii*
233	comp37900_c0_seq3.21.1322.minus.R9_1	GT4	*Alistipes_shahii*
234	comp37900_c0_seq3.21.1322.minus.R9_1	GT5	*Alistipes_shahii*
235	comp40064_c0_seq3.2295.3590.plus.R9_1	GT4	*Alistipes_shahii*
236	comp40064_c0_seq3.2295.3590.plus.R9_1	GT5	*Alistipes_shahii*
237	comp60413_c0_seq9.4188.5429.plus.R1_1	GT4	*Alistipes_shahii*
238	comp60413_c0_seq9.4188.5429.plus.R1_1	GT5	*Alistipes_shahii*
239	comp42036_c0_seq1.4984.5925.plus.R3_1	GT27	*Alistipes*_sp._AL-1
240	comp44453_c0_seq1.1.945.minus.R7_1	GT19	*Alistipes*_sp._AL-1
241	comp11507_c0_seq1.2184.3473.plus.R7_1	GT5	*Alistipes*_sp._CAG
242	comp11507_c0_seq1.2184.3473.plus.R7_1	GT4	*Alistipes*_sp._CAG
243	comp155714_c0_seq1.7.734.minus.R1_1	GT9	*Alistipes*_sp._CAG
244	comp26879_c0_seq1.533.1714.minus.R3_1	GT28	*Alistipes*_sp._CAG
245	comp34625_c0_seq2.1.763.minus.R9_1	GT3	*Alistipes*_sp._CAG
246	comp40438_c0_seq15.1111.2016.plus.R9_1	GT26	*Alistipes*_sp._CAG
247	comp48843_c0_seq9.298.1599.plus.R8_1	GT4	*Alistipes*_sp._CAG
248	comp48843_c0_seq9.298.1599.plus.R8_1	GT5	*Alistipes*_sp._CAG
249	comp53440_c0_seq1.143.1072.minus.R3_1	GT2	*Alistipes*_sp._CAG
250	comp61626_c0_seq8.1.915.minus.R1_1	GT5	*Alistipes*_sp._CAG
251	comp70127_c0_seq1.14.976.plus.R7_1	GT9	*Alistipes*_sp._CAG
252	comp9387_c0_seq1.1.915.minus.R8_1	GT5	*Alistipes*_sp._CAG
253	comp35098_c0_seq22.2167.3315.minus.R3_1	GT19	*Alistipes*_sp._CHKCI003
254	comp55081_c0_seq1.27.872.plus.R3_1	GT2	*Alistipes*_sp._CHKCI003
255	comp18360_c0_seq1.184.1404.minus.R3_1	GT30	*Alistipes*_sp._HGB5
256	comp60413_c0_seq9.88.1857.plus.R1_1	GT35	*Alistipes*_sp._HGB5
257	comp29041_c0_seq1.2388.3344.plus.R3_1	GT2	*Alistipes*_sp._Marseille-P2431
258	comp52591_c0_seq2.1.1487.minus.R7_1	GT3	*Alistipes*_timonensis

259	comp52591_c0_seq3.1.1736.minus.R7_1	GT3	*Alistipes*_norank
260	comp36154_c0_seq4.4873.9123.minus.R3_1	GT35	*Alistipes*_norank
261	comp36154_c0_seq4.4873.9123.minus.R3_1	GT3	*Alistipes*_norank
262	comp32293_c0_seq1.1142.2368.plus.R1_1	GT4	*Adhaeribacter_aquaticus*
263	comp13117_c0_seq1.334.2005.minus.R8_1	GT2	*Flavobacterium_johnsoniae*
264	comp66736_c0_seq1.217.1023.minus.R3_1	GT2	*Flavobacterium_johnsoniae*
265	comp13663_c0_seq1.5452.6384.plus.R1_1	GT8	*Flavobacterium*_sp._MEB061
266	comp88962_c0_seq1.251.1087.minus.R1_1	GT2	*Bacteroidetes*_bacterium_38_7
267	comp134211_c0_seq1.1.871.minus.R1_1	GT4	*Bacteroidetes*_bacterium_oral_taxon_272
268	comp42455_c0_seq1.104.997.plus.R7_1	GT5	*Mucispirillum_schaedleri*
269	comp116133_c0_seq1.51.854.plus.R1_1	GT4	*Bacillus_coagulans*
270	comp7753_c0_seq1.1.778.minus.R9_1	GT80	*Paenibacillus*_sp._E194
271	comp91160_c0_seq1.1.1040.minus.R8_1	GT4	*Lactobacillus_salivarius*
272	comp76167_c0_seq1.1.940.minus.R8_1	GT5	*Butyricicoccus_pullicaecorum*
273	comp41999_c0_seq1.417.1478.minus.R8_1	GT2	*Clostridium_dakarense*
274	comp114731_c0_seq1.55.1266.plus.R1_1	GT2	*Clostridium*_sp._CAG
275	comp15074_c0_seq1.1.892.minus.R8_1	GT35	*Clostridium*_sp._CAG
276	comp39418_c0_seq4.1.974.minus.R9_1	GT35	*Clostridium*_sp._CAG
277	comp40772_c0_seq1.58.795.plus.R1_1	GT5	*Clostridium*_sp._CAG
278	comp46789_c0_seq1.28.1071.minus.R8_1	GT5	*Clostridium*_sp._CAG
279	comp48560_c0_seq5.22.1431.plus.R8_1	GT5	*Clostridium*_sp._CAG
280	comp49908_c0_seq1.5.1579.minus.R8_1	GT5	*Clostridium*_sp._CAG
281	comp49908_c0_seq4.5.1175.minus.R8_1	GT5	*Clostridium*_sp._CAG
282	comp59380_c0_seq1.23.1249.plus.R1_1	GT35	*Clostridium*_sp._CAG
283	comp60711_c0_seq4.1.936.minus.R1_1	GT35	*Clostridium*_sp._CAG
284	comp63927_c0_seq1.953.2374.plus.R3_1	GT5	*Clostridium*_sp._CAG
285	comp77718_c0_seq1.173.1147.minus.R8_1	GT2	*Clostridium*_sp._CAG
286	comp9041_c0_seq1.1.822.minus.R8_1	GT8	*Clostridium*_sp._CAG
287	comp98000_c0_seq1.245.1042.minus.R7_1	GT28	*Clostridium*_sp._CAG
288	comp26911_c0_seq2.21.1097.plus.R8_1	GT2	*Clostridium*_sp._GD3
289	comp135923_c0_seq1.1.737.minus.R1_1	GT2	uncultured_*Clostridium*_sp.
290	comp45789_c0_seq1.1.1818.minus.R7_1	GT35	uncultured_*Clostridium*_sp.
291	comp65997_c0_seq1.531.1466.plus.R8_1	GT81	uncultured_*Clostridium*_sp.
292	comp31289_c0_seq1.37.1098.plus.R1_1	GT2	*Anaerofustis_stercorihominis*
293	comp26066_c0_seq1.1.775.minus.R3_1	GT5	*Eubacterium_desmolans*
294	comp49621_c0_seq4.1.1722.minus.R8_1	GT35	*Eubacterium_desmolans*
295	comp62742_c0_seq3.40.2473.minus.R1_1	GT35	*Eubacterium_desmolans*
296	comp40387_c0_seq1.3377.4804.plus.R7_1	GT5	*Eubacterium*_sp._CAG
297	comp33152_c0_seq1.62.1513.plus.R1_1	GT5	*Eubacteriaceae*_bacterium_CHKCI004
298	comp47178_c0_seq1.1989.3011.plus.R1_1	GT2	*Eubacteriaceae*_bacterium_CHKCI004
299	comp49174_c0_seq2.11.1885.plus.R8_1	GT35	*Eubacteriaceae*_bacterium_CHKCI004
300	comp59168_c0_seq2.1.1657.minus.R1_1	GT35	*Eubacteriaceae*_bacterium_CHKCI004
301	comp47315_c0_seq3.788.3061.plus.R8_1	GT35	*Eubacteriaceae*_bacterium_CHKCI004
302	comp59168_c0_seq1.97.2532.minus.R1_1	GT35	*Eubacteriaceae*_bacterium_CHKCI004

303	comp105266_c0_seq1.170.1159.plus.R1_1	GT2	[*Ruminococcus*]_*torques*
304	comp62802_c0_seq1.5.1090.plus.R8_1	GT2	*Blautia_producta*
305	comp12070_c0_seq1.771.1508.plus.R1_1	GT5	*Blautia_schinkii*
306	comp12087_c0_seq1.1.807.minus.R1_1	GT28	*Blautia_schinkii*
307	comp46680_c0_seq2.20.1468.plus.R8_1	GT5	*Blautia_schinkii*
308	comp46680_c0_seq3.170.1615.plus.R8_1	GT5	*Blautia_schinkii*
309	comp110691_c0_seq1.35.1420.minus.R7_1	GT5	*Blautia*_sp._CAG
310	comp62111_c0_seq3.257.1240.plus.R1_1	GT2	*Butyrivibrio*_sp._AE2032
311	comp71349_c0_seq1.252.1853.plus.R1_1	GT28	[*Clostridium*]_*aminophilum*
312	comp14534_c0_seq1.134.1162.plus.R8_1	GT2	[*Clostridium*]_*citroniae*
313	comp120508_c0_seq1.59.1356.minus.R1_1	GT4	*Lachnospiraceae*_bacterium_6_1_63FAA
314	comp43640_c0_seq1.17.1309.plus.R8_1	GT4	*Lachnospiraceae*_bacterium_6_1_63FAA
315	comp18265_c0_seq1.15.956.plus.R1_1	GT81	*Tyzzerella_nexilis*
316	comp9307_c0_seq1.3718.4833.plus.R3_1	GT19	*Tyzzerella_nexilis*
317	comp60711_c0_seq2.1.1253.minus.R1_1	GT35	uncultured_*Flavonifractor*_sp.
318	comp53444_c0_seq2.23.1060.plus.R7_1	GT35	*Clostridiales*_bacterium_VE202-09
319	comp59991_c0_seq1.87.2537.minus.R1_1	GT35	*Clostridiales*_bacterium_VE202-09
320	comp19851_c0_seq1.339.1432.minus.R8_1	GT2	*Clostridiales*_bacterium_VE202-13
321	comp62516_c0_seq1.1885.2955.plus.R1_1	GT4	*Oscillospiraceae*_bacterium_VE202-24
322	comp132477_c0_seq1.176.987.minus.R1_1	GT8	*Desulfosporosinus*_sp._BG
323	comp11817_c0_seq1.1.702.minus.R3_1	GT4	*Faecalibacterium_prausnitzii*
324	comp33368_c0_seq2.1.1754.minus.R3_1	GT35	*Faecalibacterium_prausnitzii*
325	comp49908_c0_seq10.117.1799.minus.R8_1	GT5	*Faecalibacterium_prausnitzii*
326	comp58008_c0_seq1.239.1366.plus.R1_1	GT28	*Faecalibacterium_prausnitzii*
327	comp60295_c0_seq1.748.1728.plus.R1_1	GT2	*Faecalibacterium_prausnitzii*
328	comp61936_c0_seq1.618.2097.minus.R1_1	GT27	*Faecalibacterium_prausnitzii*
329	comp61250_c0_seq1.120.2675.plus.R1_1	GT51	*Faecalibacterium_prausnitzii*
330	comp61936_c0_seq2.315.2808.minus.R1_1	GT2	*Faecalibacterium_prausnitzii*
331	comp61936_c0_seq2.315.2808.minus.R1_1	GT2	*Faecalibacterium_prausnitzii*
332	comp62530_c0_seq2.133.2529.plus.R1_1	GT35	*Faecalibacterium_prausnitzii*
333	comp42732_c0_seq1.3253.4695.plus.R8_1	GT5	*Faecalibacterium*_sp._CAG
334	comp60432_c0_seq1.39.1658.minus.R1_1	GT4	*Faecalibacterium*_sp._CAG
335	comp61494_c0_seq1.124.1155.plus.R1_1	GT2	*Faecalibacterium*_sp._CAG
336	comp31423_c0_seq2.1.893.minus.R3_1	GT4	uncultured_*Faecalibacterium*_sp.
337	comp62516_c0_seq1.117.1862.plus.R1_1	GT4	uncultured_*Faecalibacterium*_sp.
338	comp80286_c0_seq1.1.1253.minus.R1_1	GT2	*Ruminococcaceae*_norank
339	comp91921_c0_seq1.12.803.minus.R1_1	GT26	*Ruminococcaceae*_bacterium_AM2
340	comp49174_c0_seq7.12.2162.plus.R8_1	GT35	*Ruminococcaceae*_bacterium_AM2
341	comp28609_c0_seq1.1.728.minus.R3_1	GT35	*Ruminococcus*_sp._CAG
342	comp52210_c0_seq1.95.832.plus.R1_1	GT5	*Ruminococcus*_sp._CAG
343	comp124838_c0_seq1.1.714.minus.R1_1	GT35	*Subdoligranulum*_sp._4_3_54A2FAA
344	comp61054_c0_seq1.8.838.plus.R1_1	GT5	*Subdoligranulum_variabile*
345	comp60757_c0_seq1.22.1057.minus.R1_1	GT5	*Clostridia*_bacterium_UC5.1-1E11
346	comp35649_c0_seq1.13.1077.plus.R8_1	GT2	*Clostridia*_bacterium_UC5.1-2F7

347	comp48181_c0_seq2.1489.2862.plus.R8_1	GT5	Candidatus_Stoquefichus_sp._KLE1796
348	comp61395_c0_seq4.2017.3306.plus.R1_1	GT5	Candidatus_Stoquefichus_sp._KLE1796
349	comp30729_c0_seq1.1.1027.minus.R9_1	GT2	*Coprobacillus*_sp._CAG
350	comp75322_c0_seq1.2180.3565.plus.R1_1	GT5	[*Eubacterium*]_*dolichum*
351	comp33345_c0_seq1.3.1433.minus.R3_1	GT5	*Erysipelotrichaceae*_norank
352	comp55131_c0_seq2.1113.2537.plus.R7_1	GT5	*Erysipelotrichaceae*_norank
353	comp61101_c0_seq1.62.856.plus.R1_1	GT28	*Megamonas_funiformis*
354	comp62904_c0_seq1.135.2594.plus.R1_1	GT35	*Megamonas_funiformis*
355	comp62409_c0_seq1.62.832.plus.R1_1	GT26	*Megamonas_rupellensis*
356	comp62904_c0_seq1.4704.6143.plus.R1_1	GT5	*Megamonas_rupellensis*
357	comp62921_c0_seq2.4140.5300.plus.R1_1	GT19	*Megamonas_rupellensis*
358	comp75008_c0_seq1.473.1633.plus.R1_1	GT19	*Megasphaera*_sp._MJR8396C
359	comp72472_c0_seq1.137.2404.plus.R8_1	GT35	*Firmicutes*_bacterium_ASF500
360	comp117304_c0_seq1.1.871.minus.R8_1	GT2	*Firmicutes*_bacterium_CAG
361	comp22703_c0_seq1.14.745.plus.R8_1	GT19	*Firmicutes*_bacterium_CAG
362	comp32192_c0_seq1.56.955.plus.R1_1	GT5	*Firmicutes*_bacterium_CAG
363	comp36301_c0_seq2.132.1658.plus.R8_1	GT35	*Firmicutes*_bacterium_CAG
364	comp43640_c0_seq1.1314.2423.plus.R8_1	GT4	*Firmicutes*_bacterium_CAG
365	comp46462_c0_seq1.324.1541.plus.R1_1	GT5	*Firmicutes*_bacterium_CAG
366	comp47021_c0_seq1.132.1220.plus.R8_1	GT5	*Firmicutes*_bacterium_CAG
367	comp52020_c0_seq1.66.1442.plus.R1_1	GT35	*Firmicutes*_bacterium_CAG
368	comp55974_c0_seq4.1.734.minus.R1_1	GT5	*Firmicutes*_bacterium_CAG
369	comp57167_c0_seq2.105.1625.plus.R1_1	GT5	*Firmicutes*_bacterium_CAG
370	comp57679_c0_seq1.3.1445.plus.R1_1	GT5	*Firmicutes*_bacterium_CAG
371	comp62516_c0_seq1.2985.3803.plus.R1_1	GT8	*Firmicutes*_bacterium_CAG
372	comp94089_c0_seq1.136.1272.plus.R1_1	GT4	*Firmicutes*_bacterium_CAG
373	comp61436_c0_seq2.54.2516.plus.R1_1	GT35	*Firmicutes*_bacterium_CAG
374	comp61582_c0_seq1.3.2702.plus.R1_1	GT51	*Firmicutes*_bacterium_CAG
375	comp56452_c0_seq1.1621.3000.plus.R8_1	GT5	*Fusobacterium_mortiferum*
376	comp64195_c0_seq1.1.909.minus.R8_1	GT9	*Fusobacterium_mortiferum*
377	comp26416_c0_seq1.112.2493.plus.R8_1	GT35	*Fusobacterium_mortiferum*
378	comp45151_c0_seq1.321.1514.plus.R1_1	GT35	*Fusobacterium*_sp._CAG
379	comp54007_c0_seq2.23.1755.minus.R7_1	GT35	*Fusobacterium*_sp._CAG
380	comp27905_c0_seq1.1907.2935.plus.R8_1	GT2	*Haloplasma_contractile*
381	comp6510_c0_seq1.35.869.minus.R9_1	GT2	*Haloplasma_contractile*
382	comp86555_c0_seq1.1.755.minus.R3_1	GT35	*Haloplasma*_bacterium_OL-1
383	comp64548_c0_seq1.9779.11035.plus.R1_1	GT4	*Haloplasma*_bacterium_SIT8
384	comp117213_c0_seq1.10.945.plus.R1_1	GT35	*Acetobacter*_sp._CAG
385	comp10436_c0_seq1.122.961.plus.R7_1	GT39	*Acidiphilium*_sp._CAG
386	comp16102_c0_seq1.56.895.plus.R7_1	GT35	*Acidiphilium*_sp._CAG
387	comp25216_c0_seq2.1.821.minus.R9_1	GT35	*Acidiphilium*_sp._CAG
388	comp30481_c0_seq2.1.741.minus.R9_1	GT35	*Acidiphilium*_sp._CAG
389	comp54630_c0_seq2.26.1030.plus.R7_1	GT5	*Acidiphilium*_sp._CAG
390	comp77866_c0_seq1.1.1104.minus.R8_1	GT35	*Acidiphilium*_sp._CAG

391	comp86599_c0_seq1.1.726.minus.R8_1	GT35	*Acidiphilium*_sp._CAG
392	comp158888_c0_seq1.36.746.plus.R1_1	GT2	*Sutterella*_sp._CAG
393	comp14742_c0_seq1.48.1172.plus.R1_1	GT4	*Thiobacillus*_sp._SCN_63-1177
394	comp100310_c0_seq1.25.1044.plus.R1_1	GT4	*Desulfovibrio_piger*
395	comp108983_c0_seq1.1.810.minus.R7_1	GT4	*Desulfovibrio_piger*
396	comp46049_c0_seq3.8.772.minus.R1_1	GT51	*Desulfovibrio_piger*
397	comp96242_c0_seq1.1.1199.minus.R3_1	GT4	*Desulfovibrio_piger*
398	comp87456_c0_seq1.1.2779.minus.R1_1	GT3	*Desulfovibrio_piger*
399	comp87148_c0_seq1.22.1095.minus.R1_1	GT28	*Geobacter_pickeringii*
400	comp16588_c0_seq1.3.1841.plus.R1_1	GT9	*Helicobacter_pullorum*
401	comp74753_c0_seq1.2236.3564.plus.R1_1	GT8	*Helicobacter_pullorum*
402	comp74753_c0_seq1.10.2232.plus.R1_1	GT99	*Helicobacter_pullorum*
403	comp84102_c0_seq1.1.747.minus.R8_1	GT82	*Pasteurella_multocida*
404	comp47151_c0_seq1.16.1710.minus.R8_1	GT35	*Acinetobacter*_sp._CAG
405	comp61668_c0_seq1.3012.5504.plus.R1_1	GT35	*Spirochaeta_lutea*
406	comp38794_c0_seq3.4.1032.plus.R9_1	GT19	*Cloacibacillus_evryensis*
407	comp116671_c0_seq1.1.978.minus.R7_1	GT51	*Cloacibacillus_porcorum*
408	comp34638_c0_seq1.1.730.minus.R3_1	GT30	*Cloacibacillus_porcorum*
409	comp52925_c0_seq1.1.1284.minus.R7_1	GT26	*Cloacibacillus_porcorum*
410	comp55666_c0_seq2.1120.2184.plus.R7_1	GT19	*Synergistes*_sp._3_1_syn1
411	comp50023_c0_seq1.1076.4362.minus.R8_1	GT35	*Synergistes*_sp._3_1_syn1
412	comp121816_c0_seq1.61.978.plus.R7_1	GT4	*Thermovirga_lienii*
413	comp123299_c0_seq1.52.927.plus.R1_1	GT30	*Verrucomicrobia*_bacterium_L21-Fru-AB
414	comp46029_c0_seq1.652.2223.plus.R1_1	GT5	*Verrucomicrobia*_bacterium_L21-Fru-AB
415	comp91791_c0_seq1.1.1237.minus.R1_1	GT35	*Opitutaceae*_bacterium_TSB47
416	comp88144_c0_seq1.1.1448.minus.R1_1	GT5	*Coraliomargarita*_sp._CAG
417	comp111808_c0_seq1.1.934.minus.R8_1	GT35	*Blastocystis_hominis*
418	comp128347_c0_seq1.1.718.minus.R8_1	GT8	*Blastocystis_hominis*
419	comp132745_c0_seq1.1.835.minus.R1_1	GT92	*Blastocystis_hominis*
420	comp143649_c0_seq1.1.909.minus.R1_1	GT49	*Blastocystis_hominis*
421	comp16297_c0_seq2.1.1387.minus.R1_1	GT35	*Blastocystis_hominis*
422	comp16513_c0_seq1.1.1102.minus.R1_1	GT31	*Blastocystis_hominis*
423	comp16533_c0_seq1.1.1450.minus.R1_1	GT23	*Blastocystis_hominis*
424	comp18387_c0_seq1.1.1368.minus.R1_1	GT5	*Blastocystis_hominis*
425	comp29315_c0_seq2.1.1288.minus.R3_1	GT75	*Blastocystis_hominis*
426	comp30008_c0_seq1.1.1115.minus.R3_1	GT4	*Blastocystis_hominis*
427	comp33356_c0_seq1.1.1464.minus.R1_1	GT92	*Blastocystis_hominis*
428	comp36692_c0_seq2.9.950.plus.R1_1	GT49	*Blastocystis_hominis*
429	comp45901_c0_seq1.275.1261.plus.R1_1	GT23	*Blastocystis_hominis*
430	comp46750_c0_seq1.1.1500.minus.R1_1	GT4	*Blastocystis_hominis*
431	comp47160_c0_seq1.1.1575.minus.R1_1	GT22	*Blastocystis_hominis*
432	comp53627_c0_seq1.1.818.minus.R1_1	GT49	*Blastocystis_hominis*
433	comp57327_c0_seq1.6.945.minus.R1_1	GT31	*Blastocystis_hominis*
434	comp57327_c0_seq2.6.945.minus.R1_1	GT31	*Blastocystis_hominis*

435	comp61060_c0_seq5.1.1884.minus.R1_1	GT49	*Blastocystis_hominis*
436	comp76149_c0_seq1.1.1619.minus.R8_1	GT35	*Blastocystis_hominis*
437	comp81086_c0_seq1.1.1134.minus.R1_1	GT23	*Blastocystis_hominis*
438	comp84477_c0_seq1.1.854.minus.R8_1	GT49	*Blastocystis_hominis*
439	comp96106_c0_seq1.1.833.minus.R8_1	GT49	*Blastocystis_hominis*
440	comp38554_c0_seq1.1.2095.minus.R8_1	GT66	*Blastocystis_hominis*
441	comp39838_c0_seq4.82.2526.plus.R9_1	GT35	*Blastocystis_hominis*
442	comp59330_c0_seq1.1.2902.minus.R1_1	GT20	*Blastocystis_hominis*
443	comp59664_c0_seq1.296.3271.plus.R1_1	GT20	*Blastocystis_hominis*
444	comp59664_c0_seq1.296.3271.plus.R1_1	GT20	*Blastocystis_hominis*
445	comp12048_c0_seq1.1.1238.minus.R1_1	GT23	*Blastocystis*_sp._subtype_4
446	comp34767_c0_seq1.1.799.minus.R1_1	GT49	*Blastocystis*_sp._subtype_4
447	comp41601_c0_seq2.1.1122.minus.R1_1	GT49	*Blastocystis*_sp._subtype_4
448	comp62000_c0_seq2.106.2483.minus.R1_1	GT35	*Entamoeba_dispar*
449	comp55622_c0_seq4.1.1588.minus.R7_1	GT35	*Volvox_carteri*
450	comp106295_c0_seq1.1.1101.minus.R1_1	GT49	unclassified
451	comp11328_c0_seq2.1.782.minus.R1_1	GT49	unclassified
452	comp118375_c0_seq1.199.1368.plus.R1_1	GT49	unclassified
453	comp35752_c0_seq1.369.1400.minus.R8_1	GT4	unclassified
454	comp36341_c0_seq1.1.1148.minus.R1_1	GT49	unclassified
455	comp55662_c0_seq3.17.1462.plus.R1_1	GT49	unclassified
456	comp57939_c0_seq1.1.1453.minus.R1_1	GT49	unclassified
457	comp84610_c0_seq1.32.1060.plus.R1_1	GT49	unclassified
458	comp61390_c0_seq1.49.2658.plus.R1_1	GT66	unclassified
459	comp100456_c0_seq1.1.365.minus.R3_1	GT51	unclassified
460	comp101623_c0_seq1.1.402.minus.R8_1	GT2	unclassified
461	comp101952_c0_seq1.1.321.minus.R8_1	GT2	unclassified
462	comp102811_c0_seq1.1.489.minus.R9_1	GT51	unclassified
463	comp103183_c0_seq1.107.730.plus.R1_1	GT4	unclassified
464	comp103225_c0_seq1.1.390.minus.R8_1	GT2	unclassified
465	comp103727_c0_seq1.1.351.minus.R3_1	GT2	unclassified
466	comp103864_c0_seq1.291.925.minus.R8_1	GT6	unclassified
467	comp104449_c0_seq1.1.323.minus.R7_1	GT11	unclassified
468	comp104496_c0_seq1.76.771.plus.R1_1	GT4	unclassified
469	comp10451_c0_seq2.1.465.minus.R3_1	GT14	unclassified
470	comp104598_c0_seq1.1.568.minus.R3_1	GT2	unclassified
471	comp105501_c0_seq1.32.298.plus.R8_1	GT28	unclassified
472	comp105665_c0_seq1.1.501.minus.R3_1	GT19	unclassified
473	comp105799_c0_seq1.163.579.plus.R1_1	GT11	unclassified
474	comp105881_c0_seq1.1.620.minus.R8_1	GT21	unclassified
475	comp106397_c0_seq1.56.304.plus.R8_1	GT28	unclassified
476	comp10748_c0_seq1.1.523.minus.R1_1	GT27	unclassified
477	comp109056_c0_seq1.1.640.minus.R1_1	GT4	unclassified
478	comp109179_c0_seq1.1.424.minus.R9_1	GT8	unclassified

479	comp109403_c0_seq1.28.433.minus.R1_1	GT4	unclassified
480	comp110914_c0_seq1.1.346.minus.R3_1	GT32	unclassified
481	comp111439_c0_seq1.341.782.minus.R1_1	GT51	unclassified
482	comp111572_c0_seq1.1.393.minus.R1_1	GT51	unclassified
483	comp111748_c0_seq1.1.339.minus.R1_1	GT4	unclassified
484	comp113098_c0_seq1.1.554.minus.R1_1	GT83	unclassified
485	comp114033_c0_seq1.1.457.minus.R7_1	GT2	unclassified
486	comp11433_c0_seq1.21.479.plus.R1_1	GT4	unclassified
487	comp114664_c0_seq1.1.450.minus.R1_1	GT2	unclassified
488	comp115641_c0_seq1.95.736.plus.R1_1	GT4	unclassified
489	comp116342_c0_seq1.1.386.minus.R9_1	GT51	unclassified
490	comp116731_c0_seq1.1.425.minus.R9_1	GT2	unclassified
491	comp116952_c0_seq1.1.427.minus.R3_1	GT2	unclassified
492	comp117275_c0_seq1.1.636.minus.R8_1	GT92	unclassified
493	comp117532_c0_seq1.1.489.minus.R7_1	GT2	unclassified
494	comp118127_c0_seq1.1.529.minus.R1_1	GT2	unclassified
495	comp118365_c0_seq1.1.486.minus.R1_1	GT23	unclassified
496	comp11841_c0_seq1.1.294.minus.R3_1	GT2	unclassified
497	comp118744_c0_seq1.94.492.plus.R7_1	GT4	unclassified
498	comp11874_c0_seq1.1.562.minus.R1_1	GT2	unclassified
499	comp118889_c0_seq1.1.615.minus.R8_1	GT49	unclassified
500	comp119162_c0_seq1.1.415.minus.R9_1	GT49	unclassified
501	comp119944_c0_seq1.46.600.plus.R8_1	GT4	unclassified
502	comp120208_c0_seq1.8.452.minus.R8_1	GT90	unclassified
503	comp12070_c0_seq1.64.597.plus.R1_1	GT5	unclassified
504	comp121877_c0_seq1.1.554.minus.R1_1	GT49	unclassified
505	comp122010_c0_seq1.295.677.minus.R1_1	GT8	unclassified
506	comp122664_c0_seq1.1.347.minus.R1_1	GT2	unclassified
507	comp123584_c0_seq1.1.582.minus.R1_1	GT32	unclassified
508	comp124299_c0_seq1.1.303.minus.R9_1	GT2	unclassified
509	comp124478_c0_seq1.1.369.minus.R1_1	GT2	unclassified
510	comp124790_c0_seq1.1.332.minus.R9_1	GT2	unclassified
511	comp125238_c0_seq1.1.527.minus.R3_1	GT2	unclassified
512	comp126106_c0_seq1.1.308.minus.R9_1	GT4	unclassified
513	comp126210_c0_seq1.1.362.minus.R1_1	GT28	unclassified
514	comp126648_c0_seq1.1.661.minus.R3_1	GT32	unclassified
515	comp127165_c0_seq1.23.298.plus.R1_1	GT90	unclassified
516	comp127296_c0_seq1.1.359.minus.R9_1	GT19	unclassified
517	comp127990_c0_seq1.1.690.minus.R8_1	GT20	unclassified
518	comp128426_c0_seq1.1.401.minus.R8_1	GT4	unclassified
519	comp129866_c0_seq1.1.352.minus.R3_1	GT2	unclassified
520	comp131116_c0_seq1.1.328.minus.R9_1	GT2	unclassified
521	comp13211_c0_seq1.1.422.minus.R1_1	GT19	unclassified
522	comp132124_c0_seq1.1.380.minus.R3_1	GT19	unclassified

523	comp132496_c0_seq1.1.526.minus.R3_1	GT4	unclassified
524	comp132518_c0_seq1.1.514.minus.R8_1	GT51	unclassified
525	comp133323_c0_seq1.32.325.plus.R8_1	GT2	unclassified
526	comp134951_c0_seq1.1.303.minus.R8_1	GT2	unclassified
527	comp135385_c0_seq1.1.484.minus.R7_1	GT2	unclassified
528	comp136345_c0_seq1.1.587.minus.R9_1	GT30	unclassified
529	comp136712_c0_seq1.1.537.minus.R8_1	GT2	unclassified
530	comp136930_c0_seq1.7.603.minus.R8_1	GT28	unclassified
531	comp13739_c0_seq1.331.948.minus.R1_1	GT2	unclassified
532	comp137623_c0_seq1.1.525.minus.R1_1	GT30	unclassified
533	comp137669_c0_seq1.11.685.plus.R1_1	GT19	unclassified
534	comp137705_c0_seq1.1.592.minus.R1_1	GT2	unclassified
535	comp138189_c0_seq1.1.397.minus.R7_1	GT2	unclassified
536	comp138367_c0_seq1.1.324.minus.R7_1	GT9	unclassified
537	comp139487_c0_seq1.1.456.minus.R1_1	GT5	unclassified
538	comp139640_c0_seq1.74.697.plus.R1_1	GT4	unclassified
539	comp140039_c0_seq1.144.524.plus.R1_1	GT4	unclassified
540	comp140095_c0_seq1.1.550.minus.R1_1	GT5	unclassified
541	comp142459_c0_seq1.1.418.minus.R9_1	GT19	unclassified
542	comp142757_c0_seq1.6.607.minus.R1_1	GT19	unclassified
543	comp14324_c0_seq1.1.435.minus.R1_1	GT2	unclassified
544	comp144196_c0_seq1.1.329.minus.R7_1	GT2	unclassified
545	comp144910_c0_seq1.1.429.minus.R8_1	GT13	unclassified
546	comp145617_c0_seq1.1.654.minus.R1_1	GT2	unclassified
547	comp145927_c0_seq1.1.466.minus.R1_1	GT2	unclassified
548	comp14625_c0_seq1.1.336.minus.R8_1	GT2	unclassified
549	comp146797_c0_seq1.1.385.minus.R3_1	GT2	unclassified
550	comp147570_c0_seq1.1.310.minus.R1_1	GT30	unclassified
551	comp14793_c0_seq1.1.357.minus.R9_1	GT9	unclassified
552	comp148852_c0_seq1.1.675.minus.R8_1	GT31	unclassified
553	comp149373_c0_seq1.1.508.minus.R7_1	GT19	unclassified
554	comp150069_c0_seq1.1.523.minus.R7_1	GT28	unclassified
555	comp150873_c0_seq1.1.481.minus.R7_1	GT51	unclassified
556	comp152966_c0_seq1.1.623.minus.R1_1	GT2	unclassified
557	comp154528_c0_seq1.1.505.minus.R1_1	GT2	unclassified
558	comp154540_c0_seq1.1.316.minus.R1_1	GT4	unclassified
559	comp154587_c0_seq1.1.366.minus.R7_1	GT19	unclassified
560	comp154774_c0_seq1.1.393.minus.R3_1	GT4	unclassified
561	comp15522_c0_seq1.60.567.minus.R1_1	GT49	unclassified
562	comp155409_c0_seq1.1.439.minus.R3_1	GT51	unclassified
563	comp156676_c0_seq1.1.352.minus.R1_1	GT2	unclassified
564	comp15682_c0_seq1.14.312.minus.R9_1	GT4	unclassified
565	comp157239_c0_seq1.1.527.minus.R7_1	GT2	unclassified
566	comp157270_c0_seq1.1.438.minus.R3_1	GT5	unclassified

567	comp157381_c0_seq1.1.349.minus.R1_1	GT2	unclassified
568	comp157951_c0_seq1.8.316.plus.R3_1	GT28	unclassified
569	comp158041_c0_seq1.276.518.minus.R8_1	GT28	unclassified
570	comp158202_c0_seq1.31.459.plus.R1_1	GT4	unclassified
571	comp158682_c0_seq1.1.484.minus.R8_1	GT5	unclassified
572	comp160032_c0_seq1.33.608.plus.R1_1	GT11	unclassified
573	comp160349_c0_seq1.44.638.minus.R1_1	GT4	unclassified
574	comp160391_c0_seq1.1.354.minus.R1_1	GT8	unclassified
575	comp160642_c0_seq1.60.359.plus.R7_1	GT4	unclassified
576	comp162127_c0_seq1.1.581.minus.R9_1	GT51	unclassified
577	comp164515_c0_seq1.1.339.minus.R1_1	GT4	unclassified
578	comp165484_c0_seq1.1.572.minus.R7_1	GT2	unclassified
579	comp166141_c0_seq1.1.342.minus.R1_1	GT2	unclassified
580	comp166693_c0_seq1.1.303.minus.R3_1	GT2	unclassified
581	comp16670_c0_seq1.1.301.minus.R9_1	GT77	unclassified
582	comp166772_c0_seq1.1.312.minus.R8_1	GT51	unclassified
583	comp168065_c0_seq1.1.575.minus.R3_1	GT4	unclassified
584	comp168285_c0_seq1.1.302.minus.R8_1	GT30	unclassified
585	comp17063_c0_seq1.1.486.minus.R8_1	GT51	unclassified
586	comp171001_c0_seq1.1.333.minus.R1_1	GT2	unclassified
587	comp171173_c0_seq1.1.328.minus.R1_1	GT2	unclassified
588	comp17159_c0_seq2.1.167.minus.R1_1	GT2	unclassified
589	comp172804_c0_seq1.1.452.minus.R7_1	GT49	unclassified
590	comp172826_c0_seq1.1.312.minus.R3_1	GT2	unclassified
591	comp173137_c0_seq1.1.579.minus.R1_1	GT2	unclassified
592	comp173209_c0_seq1.44.413.minus.R7_1	GT26	unclassified
593	comp174395_c0_seq1.1.650.minus.R1_1	GT4	unclassified
594	comp175001_c0_seq1.1.363.minus.R1_1	GT4	unclassified
595	comp175094_c0_seq1.1.338.minus.R1_1	GT4	unclassified
596	comp175293_c0_seq1.1.366.minus.R3_1	GT9	unclassified
597	comp17563_c0_seq1.12.488.plus.R1_1	GT4	unclassified
598	comp175690_c0_seq1.1.377.minus.R1_1	GT2	unclassified
599	comp176102_c0_seq1.1.309.minus.R3_1	GT30	unclassified
600	comp176364_c0_seq1.1.329.minus.R1_1	GT4	unclassified
601	comp176406_c0_seq1.1.306.minus.R9_1	GT51	unclassified
602	comp177119_c0_seq1.1.329.minus.R1_1	GT9	unclassified
603	comp17754_c0_seq1.27.647.plus.R1_1	GT49	unclassified
604	comp17780_c0_seq1.1.447.minus.R9_1	GT5	unclassified
605	comp177949_c0_seq1.1.306.minus.R7_1	GT4	unclassified
606	comp178132_c0_seq1.1.567.minus.R9_1	GT5	unclassified
607	comp178207_c0_seq1.135.593.plus.R1_1	GT9	unclassified
608	comp178370_c0_seq1.1.225.minus.R3_1	GT2	unclassified
609	comp178506_c0_seq1.1.308.minus.R9_1	GT25	unclassified
610	comp178571_c0_seq1.1.216.minus.R3_1	GT2	unclassified

611	comp179704_c0_seq1.1.351.minus.R3_1	GT2	unclassified
612	comp181529_c0_seq1.1.379.minus.R9_1	GT2	unclassified
613	comp181873_c0_seq1.1.475.minus.R8_1	GT28	unclassified
614	comp182899_c0_seq1.290.696.minus.R1_1	GT4	unclassified
615	comp183177_c0_seq1.1.441.minus.R1_1	GT2	unclassified
616	comp18372_c0_seq1.1.548.minus.R1_1	GT4	unclassified
617	comp184140_c0_seq1.1.302.minus.R8_1	GT28	unclassified
618	comp184469_c0_seq1.1.365.minus.R9_1	GT30	unclassified
619	comp184472_c0_seq1.1.429.minus.R8_1	GT2	unclassified
620	comp184877_c0_seq1.137.470.minus.R8_1	GT28	unclassified
621	comp185829_c0_seq1.1.557.minus.R8_1	GT1	unclassified
622	comp186240_c0_seq1.1.599.minus.R1_1	GT2	unclassified
623	comp186555_c0_seq1.1.329.minus.R1_1	GT30	unclassified
624	comp187805_c0_seq1.1.528.minus.R8_1	GT5	unclassified
625	comp187893_c0_seq1.82.758.minus.R1_1	GT8	unclassified
626	comp18830_c0_seq1.1.493.minus.R7_1	GT26	unclassified
627	comp188355_c0_seq1.1.354.minus.R1_1	GT2	unclassified
628	comp18836_c0_seq1.11.946.plus.R3_1	GT27	unclassified
629	comp189872_c0_seq1.1.335.minus.R7_1	GT2	unclassified
630	comp190311_c0_seq1.1.345.minus.R8_1	GT31	unclassified
631	comp190892_c0_seq1.1.319.minus.R9_1	GT2	unclassified
632	comp19108_c0_seq1.1.368.minus.R1_1	GT28	unclassified
633	comp191130_c0_seq1.1.378.minus.R1_1	GT2	unclassified
634	comp191379_c0_seq1.1.333.minus.R1_1	GT51	unclassified
635	comp194505_c0_seq1.1.478.minus.R1_1	GT23	unclassified
636	comp194657_c0_seq1.1.379.minus.R7_1	GT19	unclassified
637	comp196212_c0_seq1.1.315.minus.R7_1	GT2	unclassified
638	comp196237_c0_seq1.1.398.minus.R7_1	GT4	unclassified
639	comp199660_c0_seq1.22.546.plus.R1_1	GT4	unclassified
640	comp199786_c0_seq1.1.346.minus.R3_1	GT2	unclassified
641	comp200487_c0_seq1.1.551.minus.R1_1	GT2	unclassified
642	comp201615_c0_seq1.1.490.minus.R1_1	GT2	unclassified
643	comp203591_c0_seq1.1.376.minus.R9_1	GT4	unclassified
644	comp203654_c0_seq1.60.461.plus.R8_1	GT4	unclassified
645	comp204085_c0_seq1.2.532.plus.R1_1	GT4	unclassified
646	comp204177_c0_seq1.330.782.minus.R1_1	GT28	unclassified
647	comp204809_c0_seq1.62.412.minus.R1_1	GT28	unclassified
648	comp204814_c0_seq1.1.344.minus.R8_1	GT4	unclassified
649	comp205582_c0_seq1.1.346.minus.R1_1	GT2	unclassified
650	comp206461_c0_seq1.1.387.minus.R1_1	GT2	unclassified
651	comp206515_c0_seq1.1.304.minus.R9_1	GT51	unclassified
652	comp207668_c0_seq1.1.450.minus.R1_1	GT30	unclassified
653	comp208710_c0_seq1.1.344.minus.R1_1	GT2	unclassified
654	comp210325_c0_seq1.2.385.plus.R7_1	GT26	unclassified

655	comp210370_c0_seq1.1.359.minus.R7_1	GT2	unclassified
656	comp210487_c0_seq1.1.361.minus.R7_1	GT2	unclassified
657	comp211247_c0_seq1.1.338.minus.R1_1	GT2	unclassified
658	comp212172_c0_seq1.1.534.minus.R1_1	GT4	unclassified
659	comp213404_c0_seq1.1.243.minus.R1_1	GT2	unclassified
660	comp214833_c0_seq1.1.312.minus.R1_1	GT51	unclassified
661	comp21561_c0_seq2.29.373.plus.R3_1	GT4	unclassified
662	comp218553_c0_seq1.1.311.minus.R3_1	GT4	unclassified
663	comp219058_c0_seq1.1.329.minus.R1_1	GT2	unclassified
664	comp219075_c0_seq1.2.301.plus.R1_1	GT4	unclassified
665	comp219606_c0_seq1.1.425.minus.R1_1	GT81	unclassified
666	comp220894_c0_seq1.61.388.minus.R1_1	GT4	unclassified
667	comp223515_c0_seq1.1.350.minus.R1_1	GT2	unclassified
668	comp228354_c0_seq1.1.477.minus.R1_1	GT5	unclassified
669	comp228694_c0_seq1.159.490.minus.R1_1	GT4	unclassified
670	comp230027_c0_seq1.1.471.minus.R1_1	GT33	unclassified
671	comp233481_c0_seq1.1.411.minus.R1_1	GT77	unclassified
672	comp23504_c0_seq1.1.319.minus.R8_1	GT28	unclassified
673	comp235932_c0_seq1.1.358.minus.R1_1	GT4	unclassified
674	comp236530_c0_seq1.1.303.minus.R1_1	GT4	unclassified
675	comp237052_c0_seq1.1.334.minus.R7_1	GT2	unclassified
676	comp23877_c0_seq1.1.515.minus.R3_1	GT11	unclassified
677	comp24114_c0_seq1.1.477.minus.R1_1	GT45	unclassified
678	comp24216_c0_seq1.1.304.minus.R9_1	GT51	unclassified
679	comp242544_c0_seq1.1.529.minus.R7_1	GT2	unclassified
680	comp244259_c0_seq1.1.412.minus.R7_1	GT2	unclassified
681	comp245322_c0_seq1.1.337.minus.R1_1	GT51	unclassified
682	comp246518_c0_seq1.1.590.minus.R1_1	GT2	unclassified
683	comp247046_c0_seq1.1.434.minus.R7_1	GT11	unclassified
684	comp24960_c0_seq1.1.698.minus.R8_1	GT28	unclassified
685	comp24960_c0_seq2.1.494.minus.R8_1	GT28	unclassified
686	comp249785_c0_seq1.1.442.minus.R1_1	GT28	unclassified
687	comp251861_c0_seq1.1.348.minus.R7_1	GT2	unclassified
688	comp25568_c0_seq2.182.739.plus.R3_1	GT2	unclassified
689	comp25648_c0_seq1.1.532.minus.R8_1	GT5	unclassified
690	comp25817_c0_seq1.1.522.minus.R1_1	GT49	unclassified
691	comp25976_c0_seq1.32.667.plus.R8_1	GT5	unclassified
692	comp260215_c0_seq1.1.531.minus.R1_1	GT2	unclassified
693	comp263318_c0_seq1.1.401.minus.R8_1	GT28	unclassified
694	comp26879_c0_seq2.116.462.minus.R3_1	GT28	unclassified
695	comp269079_c0_seq1.1.418.minus.R1_1	GT4	unclassified
696	comp26914_c0_seq1.1.459.minus.R1_1	GT49	unclassified
697	comp270424_c0_seq1.1.358.minus.R8_1	GT19	unclassified
698	comp27359_c0_seq2.1.633.minus.R3_1	GT2	unclassified

699	comp274692_c0_seq1.1.406.minus.R1_1	GT78	unclassified
700	comp274959_c0_seq1.1.487.minus.R1_1	GT2	unclassified
701	comp275273_c0_seq1.1.333.minus.R1_1	GT26	unclassified
702	comp28508_c0_seq1.110.799.plus.R3_1	GT5	unclassified
703	comp28529_c0_seq1.698.1075.plus.R1_1	GT2	unclassified
704	comp285788_c0_seq1.1.360.minus.R1_1	GT24	unclassified
705	comp286044_c0_seq1.1.335.minus.R1_1	GT2	unclassified
706	comp286454_c0_seq1.18.436.minus.R1_1	GT4	unclassified
707	comp287414_c0_seq1.1.423.minus.R8_1	GT4	unclassified
708	comp290582_c0_seq1.1.424.minus.R1_1	GT2	unclassified
709	comp29101_c0_seq1.1.354.minus.R9_1	GT2	unclassified
710	comp295765_c0_seq1.1.328.minus.R1_1	GT2	unclassified
711	comp296261_c0_seq1.1.363.minus.R3_1	GT31	unclassified
712	comp29665_c0_seq1.1.673.minus.R8_1	GT2	unclassified
713	comp29833_c0_seq1.1.440.minus.R8_1	GT19	unclassified
714	comp298411_c0_seq1.1.378.minus.R1_1	GT51	unclassified
715	comp300210_c0_seq1.1.304.minus.R8_1	GT2	unclassified
716	comp301963_c0_seq1.1.318.minus.R1_1	GT12	unclassified
717	comp302148_c0_seq1.1.365.minus.R1_1	GT30	unclassified
718	comp306754_c0_seq1.1.332.minus.R1_1	GT4	unclassified
719	comp309229_c0_seq1.1.396.minus.R8_1	GT19	unclassified
720	comp315275_c0_seq1.1.347.minus.R1_1	GT28	unclassified
721	comp31696_c0_seq1.1.328.minus.R3_1	GT26	unclassified
722	comp32170_c0_seq1.1.579.minus.R9_1	GT5	unclassified
723	comp32481_c0_seq2.67.688.minus.R7_1	GT2	unclassified
724	comp325947_c0_seq1.1.395.minus.R1_1	GT2	unclassified
725	comp32740_c0_seq1.1.620.minus.R8_1	GT2	unclassified
726	comp33067_c0_seq1.1.468.minus.R9_1	GT49	unclassified
727	comp33095_c0_seq1.1.304.minus.R3_1	GT2	unclassified
728	comp33198_c0_seq3.1.697.minus.R3_1	GT4	unclassified
729	comp336337_c0_seq1.1.359.minus.R8_1	GT92	unclassified
730	comp337601_c0_seq1.1.357.minus.R7_1	GT28	unclassified
731	comp34010_c0_seq1.1.314.minus.R7_1	GT44	unclassified
732	comp341078_c0_seq1.29.340.plus.R1_1	GT28	unclassified
733	comp3418_c0_seq1.1.423.minus.R7_1	GT4	unclassified
734	comp34334_c0_seq1.1.594.minus.R8_1	GT5	unclassified
735	comp34597_c0_seq1.1.358.minus.R1_1	GT4	unclassified
736	comp347357_c0_seq1.1.465.minus.R1_1	GT31	unclassified
737	comp350240_c0_seq1.1.351.minus.R7_1	GT4	unclassified
738	comp353266_c0_seq1.1.403.minus.R1_1	GT2	unclassified
739	comp35467_c0_seq1.1.550.minus.R3_1	GT2	unclassified
740	comp35538_c0_seq1.17.543.minus.R1_1	GT5	unclassified
741	comp35564_c0_seq1.1.338.minus.R1_1	GT2	unclassified
742	comp35583_c0_seq2.3338.4105.plus.R3_1	GT2	unclassified

743	comp35583_c0_seq2.5649.6407.plus.R3_1	GT32	unclassified
744	comp35699_c0_seq1.203.742.plus.R8_1	GT4	unclassified
745	comp35781_c1_seq17.4386.4568.plus.R3_1	GT32	unclassified
746	comp36022_c0_seq2.125.436.minus.R3_1	GT4	unclassified
747	comp36154_c0_seq5.1.367.minus.R3_1	GT4	unclassified
748	comp36581_c0_seq1.1.319.minus.R7_1	GT51	unclassified
749	comp36857_c0_seq1.1.491.minus.R7_1	GT2	unclassified
750	comp371917_c0_seq1.1.306.minus.R8_1	GT2	unclassified
751	comp37228_c0_seq1.32.502.plus.R9_1	GT4	unclassified
752	comp37239_c0_seq1.67.639.plus.R8_1	GT5	unclassified
753	comp37636_c0_seq1.25.402.plus.R1_1	GT4	unclassified
754	comp379812_c0_seq1.1.351.minus.R1_1	GT4	unclassified
755	comp38173_c0_seq1.1.549.minus.R8_1	GT78	unclassified
756	comp389715_c0_seq1.1.362.minus.R1_1	GT2	unclassified
757	comp39705_c0_seq1.23.622.plus.R1_1	GT8	unclassified
758	comp401764_c0_seq1.1.415.minus.R1_1	GT51	unclassified
759	comp40528_c0_seq1.1.354.minus.R1_1	GT2	unclassified
760	comp40843_c0_seq2.1.643.minus.R7_1	GT3	unclassified
761	comp41470_c0_seq1.1.470.minus.R7_1	GT21	unclassified
762	comp41470_c0_seq2.1.589.minus.R7_1	GT21	unclassified
763	comp42569_c0_seq1.1.494.minus.R1_1	GT5	unclassified
764	comp42649_c0_seq2.111.758.plus.R1_1	GT4	unclassified
765	comp42780_c0_seq1.1.762.minus.R7_1	GT39	unclassified
766	comp43604_c0_seq1.27.776.minus.R8_1	GT6	unclassified
767	comp441069_c0_seq1.1.336.minus.R1_1	GT4	unclassified
768	comp44408_c0_seq1.1.673.minus.R8_1	GT5	unclassified
769	comp44408_c0_seq3.1.575.minus.R8_1	GT5	unclassified
770	comp44488_c0_seq1.1.645.minus.R8_1	GT2	unclassified
771	comp44647_c0_seq1.1.619.minus.R7_1	GT2	unclassified
772	comp45843_c0_seq1.1.471.minus.R8_1	GT23	unclassified
773	comp45848_c0_seq1.1.379.minus.R7_1	GT51	unclassified
774	comp45985_c0_seq2.8.706.plus.R8_1	GT4	unclassified
775	comp46049_c0_seq2.1.662.minus.R1_1	GT51	unclassified
776	comp46327_c0_seq1.1.421.minus.R1_1	GT2	unclassified
777	comp47580_c0_seq1.1.366.minus.R1_1	GT26	unclassified
778	comp48467_c0_seq1.1905.2728.minus.R8_1	GT32	unclassified
779	comp48560_c0_seq2.35.565.plus.R8_1	GT5	unclassified
780	comp48560_c0_seq6.35.565.plus.R8_1	GT5	unclassified
781	comp49236_c0_seq1.1.693.minus.R1_1	GT2	unclassified
782	comp49908_c0_seq6.1.567.minus.R8_1	GT5	unclassified
783	comp50432_c0_seq1.1.501.minus.R1_1	GT2	unclassified
784	comp50432_c0_seq2.190.864.minus.R1_1	GT2	unclassified
785	comp50650_c0_seq1.153.623.plus.R3_1	GT4	unclassified
786	comp52591_c0_seq1.1.643.minus.R7_1	GT3	unclassified

787	comp53971_c0_seq1.958.1761.minus.R3_1	GT12	unclassified
788	comp54244_c0_seq1.76.366.plus.R1_1	GT26	unclassified
789	comp54471_c0_seq1.1.700.minus.R3_1	GT9	unclassified
790	comp54688_c0_seq1.1.621.minus.R1_1	GT5	unclassified
791	comp54688_c0_seq2.1.502.minus.R1_1	GT5	unclassified
792	comp55044_c0_seq4.8.517.plus.R7_1	GT4	unclassified
793	comp55332_c0_seq1.87.551.plus.R1_1	GT4	unclassified
794	comp57167_c0_seq1.69.728.plus.R1_1	GT5	unclassified
795	comp57250_c0_seq1.3.260.plus.R1_1	GT2	unclassified
796	comp58772_c0_seq1.201.587.plus.R1_1	GT4	unclassified
797	comp59114_c0_seq3.1.683.minus.R1_1	GT35	unclassified
798	comp60503_c0_seq1.1.357.minus.R3_1	GT2	unclassified
799	comp60711_c0_seq5.1.656.minus.R1_1	GT35	unclassified
800	comp60966_c0_seq2.1.349.minus.R1_1	GT19	unclassified
801	comp61060_c0_seq3.1.531.minus.R1_1	GT49	unclassified
802	comp61060_c0_seq6.1.418.minus.R1_1	GT49	unclassified
803	comp61424_c0_seq1.1.231.minus.R1_1	GT2	unclassified
804	comp61424_c0_seq3.1.343.minus.R1_1	GT2	unclassified
805	comp62257_c0_seq2.19.528.plus.R1_1	GT4	unclassified
806	comp6317_c0_seq1.1.318.minus.R7_1	GT39	unclassified
807	comp63569_c0_seq1.25.297.plus.R3_1	GT39	unclassified
808	comp64498_c0_seq1.1.659.minus.R3_1	GT2	unclassified
809	comp65152_c0_seq1.1.615.minus.R3_1	GT27	unclassified
810	comp68506_c0_seq1.1.379.minus.R3_1	GT32	unclassified
811	comp68989_c0_seq1.1.501.minus.R7_1	GT28	unclassified
812	comp69734_c0_seq1.1.485.minus.R9_1	GT5	unclassified
813	comp705_c0_seq1.1.697.minus.R7_1	GT2	unclassified
814	comp70893_c0_seq1.1.498.minus.R3_1	GT13	unclassified
815	comp71100_c0_seq1.1.270.minus.R3_1	GT2	unclassified
816	comp71538_c0_seq1.4319.5170.plus.R1_1	GT32	unclassified
817	comp74266_c0_seq1.1.490.minus.R3_1	GT2	unclassified
818	comp74348_c0_seq1.30.485.plus.R7_1	GT4	unclassified
819	comp74931_c0_seq1.41.670.plus.R8_1	GT4	unclassified
820	comp7597_c0_seq1.1.301.minus.R9_1	GT30	unclassified
821	comp76326_c0_seq1.1.379.minus.R3_1	GT51	unclassified
822	comp76646_c0_seq1.1.532.minus.R9_1	GT12	unclassified
823	comp77185_c0_seq1.1.454.minus.R3_1	GT2	unclassified
824	comp78166_c0_seq1.1.315.minus.R7_1	GT26	unclassified
825	comp78779_c0_seq1.1.569.minus.R9_1	GT2	unclassified
826	comp78803_c0_seq1.53.328.plus.R3_1	GT4	unclassified
827	comp79674_c0_seq1.58.580.minus.R1_1	GT4	unclassified
828	comp834_c0_seq1.20.519.minus.R1_1	GT49	unclassified
829	comp84077_c0_seq1.1.353.minus.R3_1	GT51	unclassified
830	comp84296_c0_seq1.1.494.minus.R8_1	GT5	unclassified

831	comp85007_c0_seq1.74.575.minus.R3_1	GT4	unclassified
832	comp85408_c0_seq1.1.487.minus.R7_1	GT5	unclassified
833	comp85862_c0_seq1.1.616.minus.R9_1	GT2	unclassified
834	comp86817_c0_seq1.3.392.plus.R1_1	GT4	unclassified
835	comp86950_c0_seq1.1.679.minus.R8_1	GT35	unclassified
836	comp8772_c0_seq1.8.418.plus.R9_1	GT4	unclassified
837	comp88260_c0_seq1.1.470.minus.R9_1	GT2	unclassified
838	comp88542_c0_seq1.1.493.minus.R3_1	GT4	unclassified
839	comp89673_c0_seq1.1.429.minus.R9_1	GT2	unclassified
840	comp90101_c0_seq1.1.670.minus.R9_1	GT5	unclassified
841	comp90734_c0_seq1.1.369.minus.R3_1	GT2	unclassified
842	comp91365_c0_seq1.1.474.minus.R9_1	GT45	unclassified
843	comp91909_c0_seq1.49.688.minus.R7_1	GT92	unclassified
844	comp93023_c0_seq1.1.489.minus.R3_1	GT2	unclassified
845	comp93226_c0_seq1.58.474.plus.R7_1	GT28	unclassified
846	comp93475_c0_seq1.1.464.minus.R3_1	GT5	unclassified
847	comp93963_c0_seq1.1.412.minus.R9_1	GT4	unclassified
848	comp95190_c0_seq1.1.218.minus.R9_1	GT2	unclassified
849	comp95787_c0_seq1.1.337.minus.R9_1	GT2	unclassified
850	comp95790_c0_seq1.1.397.minus.R7_1	GT2	unclassified
851	comp96232_c0_seq1.1.670.minus.R7_1	GT49	unclassified
852	comp98191_c0_seq1.1.452.minus.R1_1	GT4	unclassified
853	comp9827_c0_seq1.3052.3735.plus.R3_1	GT2	unclassified
854	comp98846_c0_seq1.1.344.minus.R9_1	GT30	unclassified
855	comp99230_c0_seq1.1.355.minus.R9_1	GT4	unclassified
856	comp99726_c0_seq1.98.653.minus.R9_1	GT2	unclassified
