# Supplementary material for: Effects of dietary supplementation with lysozyme on the structure and function of the cecal microbiota in broiler chickens
Source: PLoS One. 2019 Jun 19;14(6):e0216748. doi: 10.1371/journal.pone.0216748 (PMC6583987; doi:10.1371/journal.pone.0216748)
Supplement: S4 Table — (PDF) [file pone.0216748.s004.pdf]

S4 Table. Taxonomy of polysaccharide lyases (PL) genes identified in the cecal microbiota of broilers fed a corn-based diet supplemented with 0 (R1 in gene query name), 40 (R7 in gene query name), 100 (R8 in gene query name), or 200 ppm (R9 in gene query name) lysozyme or 400 ppm flavomycin (R3 in gene query name) [the gene names in query refer to those in transcriptome dataset deposited as PRJNA523864 in NCBI Sequence Read Archive].

| No | Query gene | Family | Taxonomy |
|---|---|---|---|
| 1 | comp81961_c0_seq1.1.766.minus.R3_1 | PL22 | *Bacteroides_barnesiae* |
| 2 | comp39102_c0_seq2.69.2171.plus.R9_1 | PL22 | *Bacteroides_barnesiae* |
| 3 | comp39102_c0_seq2.69.2171.plus.R9_1 | PL22 | *Bacteroides_barnesiae* |
| 4 | comp61733_c0_seq7.1.862.minus.R1_1 | PL22 | *Bacteroides_coprocola* |
| 5 | comp35519_c0_seq2.28.3297.minus.R3_1 | PL22 | *Bacteroides_coprocola* |
| 6 | comp35519_c0_seq2.28.3297.minus.R3_1 | PL22 | *Bacteroides_coprocola* |
| 7 | comp61733_c0_seq2.1.1270.minus.R1_1 | PL22 | *Bacteroides_coprocola*_CAG |
| 8 | comp61733_c0_seq2.1.1270.minus.R1_1 | PL22 | *Bacteroides_coprocola*_CAG |
| 9 | comp88577_c0_seq1.1.1218.minus.R7_1 | PL22 | *Bacteroides_coprophilus* |
| 10 | comp88577_c0_seq1.1.1218.minus.R7_1 | PL22 | *Bacteroides_coprophilus* |
| 11 | comp47182_c0_seq3.457.2574.plus.R8_1 | PL22 | *Bacteroides_coprophilus* |
| 12 | comp47182_c0_seq3.457.2574.plus.R8_1 | PL22 | *Bacteroides_coprophilus* |
| 13 | comp61733_c0_seq1.1.1385.minus.R1_1 | PL22 | *Bacteroides_plebeius* |
| 14 | comp43524_c0_seq1.190.2127.plus.R1_1 | PL11 | *Bacteroides_plebeius* |
| 15 | comp61733_c0_seq11.2.1978.minus.R1_1 | PL22 | *Bacteroides_plebeius* |
| 16 | comp87439_c0_seq1.16.1950.plus.R1_1 | PL8 | *Bacteroides_plebeius*_CAG |
| 17 | comp107768_c0_seq1.116.1402.plus.R7_1 | PL11 | *Bacteroides*_sp._CAG |
| 18 | comp125519_c0_seq1.1.912.minus.R8_1 | PL8 | *Bacteroides*_sp._CAG |
| 19 | comp16864_c0_seq1.1.1369.minus.R3_1 | PL12 | *Bacteroides*_sp._CAG |
| 20 | comp31491_c0_seq1.586.2345.minus.R1_1 | PL1 | *Bacteroides*_sp._CAG |
| 21 | comp34148_c0_seq1.1.1096.minus.R3_1 | PL22 | *Bacteroides*_sp._CAG |
| 22 | comp38653_c0_seq2.1108.2082.minus.R8_1 | PL22 | *Bacteroides*_sp._CAG |
| 23 | comp40451_c0_seq11.1438.2511.minus.R9_1 | PL22 | *Bacteroides*_sp._CAG |
| 24 | comp41875_c0_seq1.1.773.minus.R7_1 | PL1 | *Bacteroides*_sp._CAG |
| 25 | comp47149_c0_seq1.1.1218.minus.R8_1 | PL1 | *Bacteroides*_sp._CAG |
| 26 | comp48046_c0_seq1.17.1318.plus.R8_1 | PL24 | *Bacteroides*_sp._CAG |
| 27 | comp50604_c0_seq1.41.1065.minus.R9_1 | PL22 | *Bacteroides*_sp._CAG |
| 28 | comp61733_c0_seq4.1.1207.minus.R1_1 | PL22 | *Bacteroides*_sp._CAG |
| 29 | comp61733_c0_seq4.1.1207.minus.R1_1 | PL22 | *Bacteroides*_sp._CAG |
| 30 | comp61733_c0_seq8.13.1429.minus.R1_1 | PL22 | *Bacteroides*_sp._CAG |
| 31 | comp71987_c0_seq1.1.903.minus.R3_1 | PL22 | *Bacteroides*_sp._CAG |
| 32 | comp12131_c0_seq1.1.2002.minus.R7_1 | PL1 | *Bacteroides*_sp._CAG |
| 33 | comp20679_c0_seq1.1324.3432.minus.R1_1 | PL22 | *Bacteroides*_sp._CAG |
| 34 | comp20679_c0_seq1.1324.3432.minus.R1_1 | PL22 | *Bacteroides*_sp._CAG |
| 35 | comp26530_c0_seq1.40.2530.minus.R3_1 | PL1 | *Bacteroides*_sp._CAG |
| 36 | comp35110_c0_seq1.30.2144.plus.R3_1 | PL8 | *Bacteroides*_sp._CAG |
| 37 | comp39102_c0_seq4.12.2126.plus.R9_1 | PL22 | *Bacteroides*_sp._CAG |

| | | | |
|---|---|---|---|
| 38 | comp39102_c0_seq4.12.2126.plus.R9_1 | PL22 | *Bacteroides*_sp._CAG |
| 39 | comp52153_c0_seq1.1.2303.minus.R7_1 | PL22 | *Bacteroides*_sp._CAG |
| 40 | comp52153_c0_seq1.1.2303.minus.R7_1 | PL22 | *Bacteroides*_sp._CAG |
| 41 | comp55313_c0_seq1.1834.4020.minus.R1_1 | PL10 | *Bacteroides*_sp._CAG |
| 42 | comp57043_c0_seq1.37.2148.plus.R7_1 | PL1 | *Bacteroides*_sp._CAG |
| 43 | comp69879_c0_seq1.9.2910.minus.R7_1 | PL1 | *Bacteroides*_sp._CAG |
| 44 | comp85359_c0_seq1.29.2425.minus.R7_1 | PL1 | *Bacteroides*_sp._CAG |
| 45 | comp30002_c0_seq1.34.2448.minus.R9_1 | PL8 | uncultured_*Bacteroides*_sp. |
| 46 | comp56422_c0_seq3.1.1997.minus.R1_1 | PL1 | *Butyricimonas*_sp._Marseille-P2440 |
| 47 | comp61157_c0_seq3.23.3202.plus.R1_1 | PL22 | *Parabacteroides_gordonii* |
| 48 | comp82888_c0_seq1.1.1160.minus.R7_1 | PL22 | *Parabacteroides*_sp._20_3 |
| 49 | comp26530_c0_seq2.1.754.minus.R3_1 | PL1 | *Prevotella*_sp._CAG |
| 50 | comp45450_c0_seq1.69.1187.minus.R3_1 | PL10 | *Prevotella*_sp._CAG |
| 51 | comp35255_c0_seq5.1.2169.minus.R3_1 | PL22 | *Prevotella*_sp._CAG |
| 52 | comp36128_c0_seq9.3617.5782.plus.R3_1 | PL22 | *Prevotella*_sp._CAG |
| 53 | comp52392_c0_seq3.186.2993.minus.R7_1 | PL8 | *Alistipes_shahii* |
| 54 | comp28890_c0_seq1.5.2958.minus.R7_1 | PL8 | *Alistipes*_sp._CAG |
| 55 | comp53792_c0_seq1.1.1364.minus.R7_1 | PL22 | *Alistipes*_sp._HGB5 |
| 56 | comp111210_c0_seq1.1.1357.minus.R1_1 | PL22 | *Alistipes_timonensis* |
| 57 | comp34040_c0_seq1.1.979.minus.R9_1 | PL9 | *Clostridium*_sp._CAG |
| 58 | comp34040_c0_seq2.1.1095.minus.R9_1 | PL9 | *Clostridium*_sp._CAG |
| 59 | comp96388_c0_seq1.1.930.minus.R8_1 | PL9 | *Clostridium*_sp._CAG |
| 60 | comp30492_c0_seq1.1.842.minus.R1_1 | PL11 | uncultured_*Clostridium*_sp. |
| 61 | comp8307_c0_seq1.1.2646.minus.R8_1 | PL11 | uncultured_*Clostridium*_sp. |
| 62 | comp57480_c0_seq3.509.1822.plus.R1_1 | PL22 | *Desulfovibrio_piger* |
| 63 | comp13319_c0_seq1.1735.2931.minus.R1_1 | PL22 | *Verrucomicrobia*_bacterium_L21-Fru-AB |
| 64 | comp100868_c0_seq1.174.565.minus.R3_1 | PL12 | unclassified |
| 65 | comp103195_c0_seq1.1.454.minus.R3_1 | PL10 | unclassified |
| 66 | comp112106_c0_seq1.1.459.minus.R3_1 | PL8 | unclassified |
| 67 | comp113745_c0_seq1.26.415.plus.R7_1 | PL20 | unclassified |
| 68 | comp114128_c0_seq1.1.649.minus.R7_1 | PL1 | unclassified |
| 69 | comp134929_c0_seq1.1.337.minus.R3_1 | PL22 | unclassified |
| 70 | comp143315_c0_seq1.1.403.minus.R7_1 | PL10 | unclassified |
| 71 | comp148097_c0_seq1.1.368.minus.R7_1 | PL1 | unclassified |
| 72 | comp151996_c0_seq1.1.440.minus.R3_1 | PL10 | unclassified |
| 73 | comp153733_c0_seq1.1.354.minus.R7_1 | PL8 | unclassified |
| 74 | comp15397_c0_seq1.1.663.minus.R7_1 | PL9 | unclassified |
| 75 | comp15408_c0_seq1.1.257.minus.R9_1 | PL18 | unclassified |
| 76 | comp162842_c0_seq1.1.320.minus.R7_1 | PL1 | unclassified |
| 77 | comp16493_c0_seq1.400.1199.minus.R7_1 | PL6 | unclassified |
| 78 | comp171632_c0_seq1.1.516.minus.R7_1 | PL3 | unclassified |
| 79 | comp174271_c0_seq1.1.398.minus.R3_1 | PL8 | unclassified |
| 80 | comp177469_c0_seq1.1.373.minus.R1_1 | PL8 | unclassified |
| 81 | comp181382_c0_seq1.1.312.minus.R7_1 | PL12 | unclassified |

| | | | |
|---|---|---|---|
| 82 | comp207618_c0_seq1.1.416.minus.R3_1 | PL22 | unclassified |
| 83 | comp328151_c0_seq1.1.334.minus.R8_1 | PL10 | unclassified |
| 84 | comp35801_c0_seq1.1.453.minus.R3_1 | PL8 | unclassified |
| 85 | comp39199_c0_seq1.1.400.minus.R8_1 | PL8 | unclassified |
| 86 | comp426005_c0_seq1.1.318.minus.R8_1 | PL10 | unclassified |
| 87 | comp56972_c0_seq2.1.657.minus.R1_1 | PL22 | unclassified |
| 88 | comp82017_c0_seq1.1.328.minus.R9_1 | PL12 | unclassified |
| 89 | comp82677_c0_seq1.1.626.minus.R7_1 | PL1 | unclassified |
| 90 | comp97211_c0_seq1.5.449.minus.R7_1 | PL14 | unclassified |
