# Supplementary material for: Effects of dietary supplementation with lysozyme on the structure and function of the cecal microbiota in broiler chickens
Source: PLoS One. 2019 Jun 19;14(6):e0216748. doi: 10.1371/journal.pone.0216748 (PMC6583987; doi:10.1371/journal.pone.0216748)
Supplement: S5 Table — (PDF) [file pone.0216748.s005.pdf]

S5 Table. Taxonomy of carbohydrate esterase (CE) genes identified in the cecal microbiota of broilers fed a corn-based diet supplemented with 0 (R1 in gene query name), 40 (R7 in gene query name), 100 (R8 in gene query name), or 200 ppm (R9 in gene query name) lysozyme or 400 ppm flavomycin (R3 in gene query name) [the gene names in query refer to the those in the transcriptome dataset deposited as PRJNA523864 in NCBI Sequence Read Archive].

No	Gene query	Family	Taxonomy
1	comp50727_c0_seq2.1132.2946.minus.R1_1	CE1	*Methanococcoides_methylutens*
2	comp15164_c0_seq1.1.955.minus.R1_1	CE8	*Bifidobacterium_longum*
3	comp70490_c0_seq1.1320.2486.plus.R8_1	CE9	uncultured_*Collinsella*_sp.
4	comp11827_c0_seq1.570.1448.minus.R1_1	CE1	*Bacteroides_barnesiae*
5	comp127914_c0_seq1.16.720.plus.R1_1	CE1	*Bacteroides_barnesiae*
6	comp31612_c0_seq2.40.1206.minus.R3_1	CE9	*Bacteroides_barnesiae*
7	comp35446_c1_seq15.3792.4670.plus.R3_1	CE1	*Bacteroides_barnesiae*
8	comp54358_c0_seq2.3921.5153.plus.R1_1	CE1	*Bacteroides_barnesiae*
9	comp55836_c0_seq1.138.1367.plus.R8_1	CE1	*Bacteroides_barnesiae*
10	comp10222_c0_seq1.2.2437.plus.R3_1	CE1	*Bacteroides_barnesiae*
11	comp34366_c0_seq1.863.2848.plus.R3_1	CE14	*Bacteroides_barnesiae*
12	comp36094_c0_seq1.6.2435.plus.R9_1	CE1	*Bacteroides_barnesiae*
13	comp39102_c0_seq2.69.2171.plus.R9_1	CE10	*Bacteroides_barnesiae*
14	comp61839_c0_seq3.1862.3247.minus.R1_1	CE11	*Bacteroides_caccae*
15	comp69554_c0_seq1.3.851.plus.R7_1	CE1	*Bacteroides_cellulosilyticus*
16	comp54677_c0_seq1.27.782.plus.R3_1	CE2	*Bacteroides_cellulosilyticus*_CAG
17	comp104274_c0_seq1.87.872.minus.R1_1	CE1	*Bacteroides_coprocola*
18	comp49978_c0_seq1.1.734.minus.R7_1	CE7	*Bacteroides_coprocola*
19	comp74211_c0_seq1.16.858.plus.R3_1	CE10	*Bacteroides_coprocola*
20	comp29214_c0_seq1.11.790.plus.R7_1	CE1	*Bacteroides_coprocola*_CAG
21	comp61839_c0_seq4.970.2266.minus.R1_1	CE11	*Bacteroides_coprocola*_CAG
22	comp63824_c0_seq1.6408.7604.plus.R1_1	CE1	*Bacteroides_coprocola*_CAG
23	comp75802_c0_seq1.70.1239.minus.R1_1	CE9	*Bacteroides_coprocola*_CAG
24	comp33125_c0_seq1.1.2245.minus.R1_1	CE10	*Bacteroides_coprocola*_CAG
25	comp54193_c0_seq3.468.1319.plus.R7_1	CE10	*Bacteroides_coprophilus*
26	comp55185_c0_seq7.1114.2499.plus.R7_1	CE11	*Bacteroides_coprophilus*
27	comp47182_c0_seq3.457.2574.plus.R8_1	CE10	*Bacteroides_coprophilus*
28	comp49022_c1_seq3.6491.8527.plus.R8_1	CE14	*Bacteroides_coprophilus*
29	comp32702_c0_seq1.1467.3278.plus.R1_1	CE6	*Bacteroides_coprophilus*_CAG
30	comp32702_c0_seq1.1467.3278.plus.R1_1	CE3	*Bacteroides_coprophilus*_CAG
31	comp33424_c0_seq2.47.1579.plus.R1_1	CE10	*Bacteroides_coprophilus*_CAG
32	comp66659_c0_seq1.1241.2410.plus.R1_1	CE9	*Bacteroides_coprophilus*_CAG
33	comp73695_c0_seq1.74.2275.plus.R1_1	CE1	*Bacteroides_coprophilus*_CAG
34	comp34787_c0_seq13.1160.2545.plus.R3_1	CE11	*Bacteroides_eggerthii*
35	comp34787_c0_seq14.1160.2545.plus.R3_1	CE11	*Bacteroides_eggerthii*

36	comp34787_c0_seq18.1160.2545.plus.R3_1	CE11	*Bacteroides_eggerthii*
37	comp39647_c0_seq2.63.1448.plus.R9_1	CE11	*Bacteroides_eggerthii*
38	comp48962_c0_seq7.1057.2442.plus.R8_1	CE11	*Bacteroides_eggerthii*
39	comp55185_c0_seq4.1075.2460.plus.R7_1	CE11	*Bacteroides_eggerthii*
40	comp113044_c0_seq1.61.876.minus.R1_1	CE1	*Bacteroides_faecichinchillae*
41	comp163651_c0_seq1.1.747.minus.R1_1	CE10	*Bacteroides_faecichinchillae*
42	comp48962_c0_seq10.1057.2442.plus.R8_1	CE11	*Bacteroides_fluxus*
43	comp67154_c0_seq1.339.1106.plus.R3_1	CE3	*Bacteroides_fragilis*
44	comp54649_c0_seq3.1.772.minus.R1_1	CE11	*Bacteroides_helcogenes*
45	comp11687_c0_seq1.7.894.plus.R3_1	CE10	*Bacteroides_plebeius*
46	comp207556_c0_seq1.30.782.plus.R1_1	CE12	*Bacteroides_plebeius*
47	comp31913_c0_seq2.129.977.minus.R1_1	CE10	*Bacteroides_plebeius*
48	comp32756_c0_seq2.803.1972.plus.R1_1	CE9	*Bacteroides_plebeius*
49	comp51127_c0_seq1.2.1168.plus.R3_1	CE9	*Bacteroides_plebeius*
50	comp53071_c0_seq2.48.917.plus.R1_1	CE1	*Bacteroides_plebeius*
51	comp43524_c0_seq1.2158.4308.plus.R1_1	CE12	*Bacteroides_plebeius*
52	comp58590_c0_seq3.855.2840.plus.R1_1	CE14	*Bacteroides_plebeius*
53	comp61733_c0_seq11.2.1978.minus.R1_1	CE10	*Bacteroides_plebeius*
54	comp55016_c0_seq5.122.1810.plus.R7_1	CE14	*Bacteroides_plebeius*_CAG
55	comp61839_c0_seq9.2391.3776.minus.R1_1	CE11	*Bacteroides_plebeius*_CAG
56	comp87133_c0_seq1.36.894.minus.R3_1	CE12	*Bacteroides_plebeius*_CAG
57	comp28630_c0_seq1.986.1833.minus.R7_1	CE10	*Bacteroides_salanitronis*
58	comp68970_c0_seq1.21.1208.plus.R1_1	CE1	*Bacteroides_salanitronis*
59	comp75050_c0_seq1.1.1586.minus.R3_1	CE8	*Bacteroides_salanitronis*
60	comp6947_c0_seq1.1.1228.minus.R3_1	CE1	*Bacteroides*_sp._4_1_36
61	comp35446_c1_seq15.1185.3623.plus.R3_1	CE1	*Bacteroides*_sp._4_1_36
62	comp10449_c0_seq1.51.875.plus.R1_1	CE1	*Bacteroides*_sp._CAG
63	comp105159_c0_seq1.141.1065.minus.R1_1	CE8	*Bacteroides*_sp._CAG
64	comp107788_c0_seq1.1.891.minus.R3_1	CE7	*Bacteroides*_sp._CAG
65	comp126347_c0_seq1.1.790.minus.R7_1	CE6	*Bacteroides*_sp._CAG
66	comp14103_c0_seq1.186.1061.minus.R1_1	CE1	*Bacteroides*_sp._CAG
67	comp143273_c0_seq1.1.753.minus.R7_1	CE12	*Bacteroides*_sp._CAG
68	comp150259_c0_seq1.1.739.minus.R7_1	CE6	*Bacteroides*_sp._CAG
69	comp16175_c0_seq1.627.1550.minus.R1_1	CE1	*Bacteroides*_sp._CAG
70	comp279567_c0_seq1.1.790.minus.R1_1	CE9	*Bacteroides*_sp._CAG
71	comp28321_c0_seq1.5.1645.plus.R3_1	CE11	*Bacteroides*_sp._CAG
72	comp28550_c0_seq1.48.1244.minus.R3_1	CE1	*Bacteroides*_sp._CAG
73	comp29604_c0_seq1.1.889.minus.R9_1	CE14	*Bacteroides*_sp._CAG
74	comp30126_c0_seq1.1.1405.minus.R3_1	CE12	*Bacteroides*_sp._CAG
75	comp30126_c0_seq1.1.1405.minus.R3_1	CE8	*Bacteroides*_sp._CAG
76	comp31301_c0_seq1.134.970.minus.R1_1	CE10	*Bacteroides*_sp._CAG
77	comp31612_c0_seq4.1204.2370.minus.R3_1	CE9	*Bacteroides*_sp._CAG
78	comp31612_c0_seq5.36.744.minus.R3_1	CE9	*Bacteroides*_sp._CAG

79	comp32871_c0_seq1.257.1302.minus.R3_1	CE1	*Bacteroides*_sp._CAG
80	comp33676_c0_seq1.29.1135.minus.R9_1	CE1	*Bacteroides*_sp._CAG
81	comp35042_c0_seq1.42.1238.minus.R9_1	CE1	*Bacteroides*_sp._CAG
82	comp35205_c0_seq2.57.1382.plus.R3_1	CE10	*Bacteroides*_sp._CAG
83	comp38744_c0_seq2.1.1838.minus.R9_1	CE10	*Bacteroides*_sp._CAG
84	comp39950_c0_seq3.297.1643.plus.R9_1	CE7	*Bacteroides*_sp._CAG
85	comp41337_c0_seq1.35.757.plus.R8_1	CE3	*Bacteroides*_sp._CAG
86	comp43263_c0_seq1.51.950.plus.R1_1	CE10	*Bacteroides*_sp._CAG
87	comp48666_c0_seq1.4.969.plus.R3_1	CE5	*Bacteroides*_sp._CAG
88	comp49038_c0_seq1.4.1638.plus.R8_1	CE6	*Bacteroides*_sp._CAG
89	comp49038_c0_seq1.4.1638.plus.R8_1	CE6	*Bacteroides*_sp._CAG
90	comp50931_c0_seq1.815.2005.minus.R3_1	CE1	*Bacteroides*_sp._CAG
91	comp53550_c0_seq1.1.885.minus.R7_1	CE9	*Bacteroides*_sp._CAG
92	comp54358_c0_seq2.2531.3877.plus.R1_1	CE10	*Bacteroides*_sp._CAG
93	comp57792_c0_seq1.443.1553.minus.R7_1	CE10	*Bacteroides*_sp._CAG
94	comp58645_c0_seq1.36.1109.plus.R1_1	CE7	*Bacteroides*_sp._CAG
95	comp59353_c0_seq8.1133.2536.plus.R1_1	CE6	*Bacteroides*_sp._CAG
96	comp59353_c0_seq8.18.1109.plus.R1_1	CE2	*Bacteroides*_sp._CAG
97	comp60551_c0_seq1.61.1047.plus.R3_1	CE1	*Bacteroides*_sp._CAG
98	comp61074_c0_seq1.35.1198.plus.R1_1	CE9	*Bacteroides*_sp._CAG
99	comp61216_c0_seq2.1180.2526.plus.R1_1	CE1	*Bacteroides*_sp._CAG
100	comp61216_c0_seq4.13.957.plus.R1_1	CE7	*Bacteroides*_sp._CAG
101	comp61733_c0_seq5.23.1559.minus.R1_1	CE10	*Bacteroides*_sp._CAG
102	comp61733_c0_seq6.23.740.minus.R1_1	CE10	*Bacteroides*_sp._CAG
103	comp61733_c0_seq8.13.1429.minus.R1_1	CE1	*Bacteroides*_sp._CAG
104	comp61839_c0_seq6.2391.3776.minus.R1_1	CE11	*Bacteroides*_sp._CAG
105	comp62289_c0_seq12.65.1375.plus.R1_1	CE7	*Bacteroides*_sp._CAG
106	comp62646_c0_seq3.2055.2918.plus.R1_1	CE10	*Bacteroides*_sp._CAG
107	comp63530_c0_seq1.1.1461.minus.R3_1	CE1	*Bacteroides*_sp._CAG
108	comp68004_c0_seq1.532.1398.minus.R1_1	CE2	*Bacteroides*_sp._CAG
109	comp68656_c0_seq1.17.950.minus.R1_1	CE6	*Bacteroides*_sp._CAG
110	comp69426_c0_seq1.64.912.minus.R3_1	CE1	*Bacteroides*_sp._CAG
111	comp71987_c0_seq1.1.903.minus.R3_1	CE1	*Bacteroides*_sp._CAG
112	comp90748_c0_seq1.1.944.minus.R7_1	CE12	*Bacteroides*_sp._CAG
113	comp91563_c0_seq1.120.1274.plus.R1_1	CE9	*Bacteroides*_sp._CAG
114	comp96081_c0_seq1.1.1181.minus.R1_1	CE1	*Bacteroides*_sp._CAG
115	comp20679_c0_seq1.1324.3432.minus.R1_1	CE10	*Bacteroides*_sp._CAG
116	comp39102_c0_seq4.12.2126.plus.R9_1	CE10	*Bacteroides*_sp._CAG
117	comp44268_c0_seq1.38.2029.plus.R8_1	CE8	*Bacteroides*_sp._CAG
118	comp54007_c0_seq2.2694.4943.plus.R1_1	CE1	*Bacteroides*_sp._CAG
119	comp55313_c0_seq1.1834.4020.minus.R1_1	CE7	*Bacteroides*_sp._CAG
120	comp59353_c0_seq11.1537.3738.plus.R1_1	CE1	*Bacteroides*_sp._CAG
121	comp61912_c0_seq10.2971.5217.minus.R1_1	CE10	*Bacteroides*_sp._CAG

122	comp61912_c0_seq12.2464.4731.minus.R1_1	CE10	*Bacteroides*_sp._CAG
123	comp85359_c0_seq1.29.2425.minus.R7_1	CE8	*Bacteroides*_sp._CAG
124	comp9325_c0_seq1.7.2973.plus.R3_1	CE3	*Bacteroides*_sp._CAG
125	comp45921_c0_seq1.67.884.minus.R8_1	CE1	*Bacteroides_stercoris*
126	comp80727_c0_seq1.121.831.plus.R1_1	CE3	*Bacteroides_thetaiotaomicron*
127	comp31612_c0_seq3.36.1199.minus.R3_1	CE9	*Bacteroides_uniformis*
128	comp56304_c0_seq1.12.1274.plus.R3_1	CE7	*Bacteroides_uniformis*
129	comp58895_c0_seq1.188.1612.plus.R3_1	CE6	*Bacteroides_uniformis*
130	comp93424_c0_seq1.1.1642.minus.R8_1	CE10	*Bacteroides_uniformis*
131	comp94352_c0_seq1.413.1603.plus.R1_1	CE1	*Bacteroides_uniformis*
132	comp34787_c0_seq11.1160.2548.plus.R3_1	CE11	*Bacteroides*_sp.
133	comp34787_c0_seq2.1160.2545.plus.R3_1	CE11	*Bacteroides*_sp.
134	comp39647_c0_seq8.63.1448.plus.R9_1	CE11	*Bacteroides*_sp.
135	comp53566_c0_seq1.707.2729.minus.R8_1	CE14	*Bacteroides*_sp.
136	comp102833_c0_seq1.12.830.plus.R7_1	CE10	uncultured_*Bacteroides*_sp.
137	comp29092_c0_seq1.441.1221.minus.R7_1	CE9	uncultured_*Bacteroides*_sp.
138	comp34794_c0_seq3.1176.2591.plus.R3_1	CE6	uncultured_*Bacteroides*_sp.
139	comp36314_c0_seq4.670.1842.minus.R9_1	CE9	uncultured_*Bacteroides*_sp.
140	comp39647_c0_seq9.1154.2539.plus.R9_1	CE11	uncultured_*Bacteroides*_sp.
141	comp41180_c0_seq1.1.1881.minus.R7_1	CE14	uncultured_*Bacteroides*_sp.
142	comp88941_c0_seq1.101.1090.plus.R8_1	CE6	*Bacteroidaceae*_bacterium_MS4
143	comp9656_c0_seq2.1.1109.minus.R8_1	CE6	*Bacteroidales*_bacterium_Barb6
144	comp49059_c0_seq2.9.719.plus.R7_1	CE1	*Bacteroidales*_bacterium
145	comp62438_c0_seq2.2483.4468.plus.R1_1	CE14	*Bacteroidales*_bacterium
146	comp25800_c0_seq1.1.1633.minus.R8_1	CE14	*Barnesiella_intestinihominis*
147	comp95885_c0_seq1.40.1374.plus.R1_1	CE11	*Barnesiella_intestinihominis*
148	comp55016_c0_seq19.7.1989.plus.R7_1	CE14	*Barnesiella_intestinihominis*
149	comp32884_c0_seq2.1.1626.minus.R3_1	CE14	*Barnesiella_viscericola*
150	comp103053_c0_seq1.267.1094.plus.R1_1	CE10	*Dysgonomonas_capnocytophagoides*
151	comp60556_c0_seq3.79.1206.plus.R1_1	CE1	*Dysgonomonas_mossii*
152	comp116415_c0_seq1.1.733.minus.R7_1	CE9	*Parabacteroides*_sp.
153	comp11706_c0_seq1.1.837.minus.R9_1	CE10	*Parabacteroides*_sp.
154	comp39242_c0_seq2.746.2134.minus.R9_1	CE11	*Parabacteroides*_sp.
155	comp62595_c0_seq18.828.2213.minus.R1_1	CE11	*Parabacteroides*_sp.
156	comp68681_c0_seq1.1.1115.minus.R9_1	CE11	*Parabacteroides*_sp.
157	comp30845_c0_seq1.60.2525.plus.R3_1	CE1	*Parabacteroides*_sp.
158	comp34829_c0_seq1.88.1281.plus.R3_1	CE1	*Parabacteroides_distasonis*
159	comp58334_c0_seq1.1.992.minus.R1_1	CE1	*Parabacteroides_distasonis*
160	comp64366_c0_seq1.141.1838.minus.R8_1	CE10	*Parabacteroides_distasonis*
161	comp75336_c0_seq1.92.1745.minus.R7_1	CE10	*Parabacteroides_distasonis*
162	comp61871_c0_seq1.7.2712.minus.R1_1	CE10	*Parabacteroides_distasonis*
163	comp62232_c0_seq1.195.2681.plus.R1_1	CE10	*Parabacteroides_distasonis*
164	comp62438_c0_seq2.1214.2374.plus.R1_1	CE9	*Parabacteroides_goldsteinii*

165	comp62381_c0_seq1.1168.2307.plus.R1_1	CE3	*Parabacteroides_gordonii*
166	comp62850_c0_seq1.178.999.plus.R9_1	CE1	*Parabacteroides_merdae*
167	comp100947_c0_seq1.926.1744.plus.R1_1	CE1	*Parabacteroides*_sp._CAG
168	comp12452_c0_seq1.625.1632.plus.R1_1	CE10	*Parabacteroides*_sp._CAG
169	comp15505_c0_seq1.1.874.minus.R1_1	CE1	*Parabacteroides*_sp._CAG
170	comp15686_c0_seq1.27.788.plus.R1_1	CE1	*Parabacteroides*_sp._CAG
171	comp29539_c0_seq3.10.1425.plus.R3_1	CE1	*Parabacteroides*_sp._CAG
172	comp36314_c0_seq2.1.894.minus.R9_1	CE9	*Parabacteroides*_sp._CAG
173	comp61839_c0_seq10.796.2181.minus.R1_1	CE11	*Parabacteroides*_sp._CAG
174	comp76522_c0_seq1.262.1422.plus.R1_1	CE9	*Parabacteroides*_sp._CAG
175	comp84081_c0_seq1.28.786.plus.R1_1	CE1	*Parabacteroides*_sp._CAG
176	comp62438_c0_seq2.32.1210.plus.R1_1	CE9	*Parabacteroides*_sp._D25
177	comp43846_c0_seq1.77.1291.minus.R1_1	CE15	*Proteiniphilum_acetatigenes*
178	comp225944_c0_seq1.1.738.minus.R1_1	CE8	*Tannerella*_sp._6_1_58FAA_CT1
179	comp108169_c0_seq1.1.1171.minus.R7_1	CE14	*Tannerella*_sp._CAG
180	comp125037_c0_seq1.47.2053.plus.R1_1	CE6	*Paraprevotella_clara*_CAG
181	comp10192_c0_seq1.2176.3000.plus.R3_1	CE1	*Prevotella*_sp._CAG
182	comp10313_c0_seq1.1277.2107.plus.R3_1	CE7	*Prevotella*_sp._CAG
183	comp10858_c0_seq1.18.1454.plus.R3_1	CE6	*Prevotella*_sp._CAG
184	comp11219_c0_seq1.46.1608.plus.R3_1	CE10	*Prevotella*_sp._CAG
185	comp112576_c0_seq1.1.717.minus.R8_1	CE14	*Prevotella*_sp._CAG
186	comp34653_c0_seq1.1070.2455.plus.R3_1	CE11	*Prevotella*_sp._CAG
187	comp44674_c0_seq1.60.884.plus.R3_1	CE10	*Prevotella*_sp._CAG
188	comp53544_c0_seq1.38.1074.minus.R3_1	CE7	*Prevotella*_sp._CAG
189	comp54996_c0_seq1.14.1381.plus.R3_1	CE1	*Prevotella*_sp._CAG
190	comp57343_c0_seq1.138.1601.minus.R3_1	CE8	*Prevotella*_sp._CAG
191	comp66282_c0_seq1.11.1153.minus.R7_1	CE3	*Prevotella*_sp._CAG
192	comp23691_c0_seq1.50.3892.plus.R7_1	CE8	*Prevotella*_sp._CAG
193	comp25252_c0_seq1.36.3356.minus.R3_1	CE8	*Prevotella*_sp._CAG
194	comp33484_c0_seq3.20.2209.plus.R3_1	CE10	*Prevotella*_sp._CAG
195	comp35255_c0_seq5.1.2169.minus.R3_1	CE1	*Prevotella*_sp._CAG
196	comp36128_c0_seq9.3617.5782.plus.R3_1	CE1	*Prevotella*_sp._CAG
197	comp38309_c0_seq1.30.2216.plus.R3_1	CE1	*Prevotella*_sp._CAG
198	comp26727_c0_seq2.1999.4128.minus.R3_1	CE10	*Alistipes_finegoldii*
199	comp33237_c0_seq3.56.1990.plus.R3_1	CE1	*Alistipes_finegoldii*
200	comp39606_c0_seq12.315.2264.minus.R9_1	CE14	*Alistipes_onderdonkii*
201	comp11523_c0_seq1.792.2189.minus.R1_1	CE11	*Alistipes_putredinis*
202	comp75969_c0_seq1.103.957.plus.R1_1	CE10	*Alistipes_putredinis*
203	comp92968_c0_seq1.73.945.plus.R3_1	CE6	*Alistipes_senegalensis*
204	comp53217_c0_seq3.1.1512.minus.R7_1	CE11	*Alistipes_shahii*
205	comp33237_c0_seq1.735.2867.plus.R3_1	CE1	*Alistipes_shahii*
206	comp64930_c0_seq1.13.726.minus.R3_1	CE3	*Alistipes*_sp._AL-1
207	comp31593_c0_seq2.1.725.minus.R9_1	CE11	*Alistipes*_sp._CAG

208	comp34787_c0_seq4.9.1400.plus.R3_1	CE11	*Alistipes*_sp._CAG
209	comp45565_c0_seq1.22.882.minus.R3_1	CE1	*Alistipes*_sp._CAG
210	comp49329_c0_seq1.1.1135.minus.R8_1	CE14	*Alistipes*_sp._CAG
211	comp50480_c0_seq2.39.866.plus.R1_1	CE10	*Alistipes*_sp._CAG
212	comp53217_c0_seq2.88.1270.minus.R7_1	CE11	*Alistipes*_sp._CAG
213	comp56480_c0_seq1.40.869.minus.R3_1	CE1	*Alistipes*_sp._CAG
214	comp61522_c0_seq1.37.903.plus.R3_1	CE10	*Alistipes*_sp._CAG
215	comp62093_c0_seq10.1.1632.minus.R1_1	CE14	*Alistipes*_sp._CAG
216	comp62093_c0_seq13.1.1614.minus.R1_1	CE14	*Alistipes*_sp._CAG
217	comp91856_c0_seq1.15.866.plus.R7_1	CE1	*Alistipes*_sp._CAG
218	comp39102_c0_seq3.24.1304.plus.R9_1	CE10	*Alistipes*_sp._CHKCI003
219	comp72051_c0_seq1.56.1381.plus.R7_1	CE10	*Alistipes*_sp._CHKCI003
220	comp161355_c0_seq1.1.763.minus.R1_1	CE6	*Alistipes_timonensis*
221	comp49329_c0_seq4.1.1099.minus.R8_1	CE14	*Alistipes_timonensis*
222	comp114957_c0_seq1.3.707.minus.R7_1	CE3	*Alistipes*_sp.
223	comp61031_c0_seq1.19.5947.minus.R1_1	CE3	*Paenibacillus*_sp._FSL_R5-0345
224	comp61031_c0_seq2.19.5926.minus.R1_1	CE3	*Paenibacillus*_sp._FSL_R5-0345
225	comp52387_c0_seq1.47.766.plus.R8_1	CE4	*Staphylococcus*_sp._CAG
226	comp58436_c0_seq3.652.1476.minus.R1_1	CE10	*Clostridium*_sp._CAG
227	comp62393_c0_seq1.3482.4390.plus.R1_1	CE4	*Clostridium*_sp._CAG
228	comp30073_c0_seq1.1.700.minus.R1_1	CE9	*Clostridium*_sp._KLE_1755
229	comp48495_c0_seq1.107.1114.plus.R1_1	CE10	*Clostridium*_sp._Marseille-P299
230	comp18974_c0_seq1.62.1687.plus.R1_1	CE12	uncultured_*Clostridium*_sp.
231	comp42893_c0_seq1.951.1809.minus.R8_1	CE4	uncultured_*Clostridium*_sp.
232	comp54141_c0_seq1.87.1655.plus.R1_1	CE1	uncultured_*Clostridium*_sp.
233	comp13498_c0_seq1.1.758.minus.R8_1	CE9	*Eubacterium_desmolans*
234	comp26157_c0_seq2.84.1340.minus.R9_1	CE9	*Eubacteriaceae*_bacterium_CHKCI004
235	comp47949_c0_seq1.1.1131.minus.R1_1	CE1	*Eubacteriaceae*_bacterium_CHKCI004
236	comp57181_c0_seq2.1139.2392.plus.R1_1	CE4	*Eubacteriaceae*_bacterium_CHKCI004
237	comp63015_c0_seq1.14.847.minus.R1_1	CE4	*Blautia_schinkii*
238	comp129537_c0_seq1.112.1038.plus.R1_1	CE7	*Blautia*_sp._YL58
239	comp15478_c0_seq1.26.1165.plus.R1_1	CE9	Roseburia_inulinivorans
240	comp41766_c0_seq1.1.979.minus.R8_1	CE10	*Roseburia*_sp._CAG
241	comp58034_c0_seq1.779.1570.plus.R1_1	CE4	*[Clostridium]_neopropionicum*
242	comp59118_c0_seq1.13.1125.minus.R9_1	CE9	*Clostridiales*_bacterium_CHKCI001
243	comp89264_c0_seq1.143.2854.plus.R1_1	CE8	*Clostridiales*_bacterium_CHKCI001
244	comp89264_c0_seq1.143.2854.plus.R1_1	CE8	*Clostridiales*_bacterium_CHKCI001
245	comp171245_c0_seq1.7.798.plus.R1_1	CE1	*Clostridiales*_bacterium_CHKCI006
246	comp59900_c0_seq2.26.1498.plus.R1_1	CE1	*Clostridiales*_bacterium_CHKCI006
247	comp109354_c0_seq1.58.1065.minus.R1_1	CE1	*Clostridiales*_bacterium_VE202-01
248	comp49712_c0_seq2.862.1977.plus.R8_1	CE9	*Clostridiales*_bacterium_VE202-21
249	comp13517_c0_seq1.1.713.minus.R3_1	CE8	*Faecalibacterium_prausnitzii*
250	comp49092_c0_seq2.669.1799.plus.R8_1	CE9	*Faecalibacterium_prausnitzii*

251	comp59136_c0_seq1.50.1206.minus.R1_1	CE2	*Faecalibacterium_prausnitzii*
252	comp61739_c0_seq1.1462.2250.plus.R1_1	CE1	*Faecalibacterium_prausnitzii*
253	comp61600_c0_seq1.291.2255.minus.R1_1	CE3	*Faecalibacterium_prausnitzii*
254	comp146591_c0_seq1.2.875.minus.R8_1	CE7	*Faecalibacterium*_sp._CAG
255	comp45383_c0_seq1.846.1720.minus.R8_1	CE4	*Faecalibacterium*_sp._CAG
256	comp53384_c0_seq1.514.1329.plus.R7_1	CE1	*Faecalibacterium*_sp._CAG
257	comp57992_c0_seq1.15.1022.minus.R1_1	CE3	*Faecalibacterium*_sp._CAG
258	comp61877_c0_seq2.554.1348.plus.R1_1	CE1	*Faecalibacterium*_sp._CAG
259	comp62252_c0_seq3.2407.3378.plus.R1_1	CE8	*Faecalibacterium*_sp._CAG
260	comp55021_c0_seq2.891.2071.minus.R7_1	CE9	*Ruminococcaceae*_norank
261	comp44006_c0_seq1.1.710.minus.R8_1	CE9	*Ruminococcaceae*_bacterium_AM2
262	comp29173_c0_seq1.1.1228.minus.R8_1	CE1	*Ruminococcaceae*_bacterium_mt9
263	comp124767_c0_seq1.1.710.minus.R9_1	CE4	*Ruminococcus_albus*
264	comp49431_c0_seq1.89.2386.plus.R8_1	CE1	uncultured_*Ruminococcus*_sp.
265	comp74143_c0_seq1.1.1124.minus.R9_1	CE9	*Subdoligranulum*_sp._4_3_54A2FAA
266	comp38399_c0_seq1.88.1208.minus.R8_1	CE9	*Clostridia*_bacterium_UC5.1-1D1
267	comp55533_c0_seq1.1.2504.minus.R1_1	CE10	*Clostridia*_bacterium_UC5.1-1E11
268	comp55533_c0_seq1.1.2504.minus.R1_1	CE1	*Clostridia*_bacterium_UC5.1-1E11
269	comp75295_c0_seq1.15.2012.minus.R8_1	CE1	[*Clostridium*]_*spiroforme*
270	comp20390_c0_seq1.1.950.minus.R3_1	CE1	*Erysipelatoclostridium_ramosum*
271	comp47923_c0_seq3.61.2106.plus.R8_1	CE1	*Erysipelatoclostridium_ramosum*
272	comp47923_c0_seq3.61.2106.plus.R8_1	CE1	*Erysipelatoclostridium_ramosum*
273	comp78148_c0_seq1.10.1149.plus.R1_1	CE9	*Erysipelotrichaceae*_bacterium_3_1_53
274	comp107475_c0_seq1.243.1073.minus.R8_1	CE4	*Phascolarctobacterium*_sp._CAG
275	comp62204_c0_seq2.6.833.plus.R1_1	CE11	*Megamonas_rupellensis*
276	comp43239_c0_seq1.912.2045.plus.R8_1	CE9	*Firmicutes*_bacterium_CAG
277	comp49209_c0_seq1.1.764.minus.R7_1	CE9	*Firmicutes*_bacterium_CAG
278	comp57482_c0_seq1.7.834.plus.R9_1	CE1	*Firmicutes*_bacterium_CAG
279	comp58436_c0_seq4.66.914.minus.R1_1	CE10	*Firmicutes*_bacterium_CAG
280	comp58995_c0_seq1.721.1476.minus.R1_1	CE10	*Firmicutes*_bacterium_CAG
281	comp8328_c0_seq1.1.906.minus.R8_1	CE4	*Firmicutes*_bacterium_CAG
282	comp83672_c0_seq1.131.1891.plus.R7_1	CE12	*Firmicutes*_bacterium_CAG
283	comp16054_c0_seq1.370.1263.plus.R1_1	CE1	*Firmicutes*_bacterium_SIT8
284	comp47969_c0_seq1.42.812.plus.R7_1	CE4	*Acidiphilium*_sp._CAG
285	comp85898_c0_seq1.8.958.plus.R1_1	CE11	*Thalassobaculum_salexigens*
286	comp30854_c0_seq1.145.1191.plus.R3_1	CE10	*Sutterella*_sp._CAG
287	comp25306_c0_seq2.6.2128.minus.R3_1	CE7	*Corallococcus*_sp._CAG
288	comp46763_c0_seq1.15.2015.minus.R8_1	CE1	*Corallococcus*_sp._CAG
289	comp47186_c0_seq1.11.2067.minus.R8_1	CE1	*Corallococcus*_sp._CAG
290	comp47186_c0_seq3.11.2067.minus.R8_1	CE1	*Corallococcus*_sp._CAG
291	comp61777_c0_seq1.50.2089.plus.R1_1	CE1	*Corallococcus*_sp._CAG
292	comp16227_c0_seq1.46.882.plus.R7_1	CE11	*Campylobacter_jejuni*
293	comp9125_c0_seq1.285.1166.minus.R3_1	CE4	*Campylobacter*__norank

294	comp49783_c0_seq1.134.1015.plus.R1_1	CE4	*Helicobacter_pullorum*
295	comp49783_c0_seq2.88.969.plus.R1_1	CE4	*Helicobacter_pullorum*
296	comp55863_c0_seq5.32.856.plus.R7_1	CE11	*Cloacibacillus_porcorum*
297	comp43432_c0_seq1.1432.2760.minus.R1_1	CE11	*Coraliomargarita*_sp._CAG
298	comp67024_c0_seq1.27.1190.plus.R1_1	CE9	*Akkermansia_glycaniphila*
299	comp199597_c0_seq1.1.929.minus.R1_1	CE4	*Blastocystis_hominis*
300	comp34160_c0_seq1.1.745.minus.R8_1	CE1	*Blastocystis_hominis*
301	comp34400_c0_seq1.22.1352.minus.R3_1	CE9	*Blastocystis_hominis*
302	comp53570_c0_seq1.1.800.minus.R1_1	CE1	*Blastocystis_hominis*
303	comp53774_c0_seq1.1.923.minus.R1_1	CE10	*Blastocystis_hominis*
304	comp59288_c0_seq1.25.1356.minus.R1_1	CE9	*Blastocystis_hominis*
305	comp50845_c0_seq2.158.1084.plus.R7_1	CE10	*Entamoeba_dispar*
306	comp100591_c0_seq1.1.557.minus.R8_1	CE5	unclassified
307	comp100621_c0_seq1.1.358.minus.R3_1	CE11	unclassified
308	comp101597_c0_seq1.4.600.plus.R3_1	CE10	unclassified
309	comp102361_c0_seq1.25.526.minus.R3_1	CE1	unclassified
310	comp104352_c0_seq1.1.649.minus.R8_1	CE9	unclassified
311	comp10560_c0_seq1.1.477.minus.R7_1	CE11	unclassified
312	comp106492_c0_seq1.1.427.minus.R7_1	CE10	unclassified
313	comp106826_c0_seq1.204.659.plus.R8_1	CE4	unclassified
314	comp107036_c0_seq1.38.450.minus.R9_1	CE1	unclassified
315	comp107839_c0_seq1.1.550.minus.R9_1	CE1	unclassified
316	comp108587_c0_seq1.1.663.minus.R3_1	CE7	unclassified
317	comp108590_c0_seq1.1.390.minus.R3_1	CE11	unclassified
318	comp108641_c0_seq1.1.640.minus.R8_1	CE1	unclassified
319	comp108874_c0_seq1.31.723.plus.R7_1	CE3	unclassified
320	comp108931_c0_seq1.402.1071.minus.R8_1	CE1	unclassified
321	comp109104_c0_seq1.1.326.minus.R7_1	CE14	unclassified
322	comp109166_c0_seq1.1.449.minus.R1_1	CE9	unclassified
323	comp109292_c0_seq1.34.507.minus.R1_1	CE9	unclassified
324	comp112114_c0_seq1.13.427.minus.R3_1	CE1	unclassified
325	comp113665_c0_seq1.53.420.minus.R1_1	CE9	unclassified
326	comp113969_c0_seq1.23.553.plus.R1_1	CE10	unclassified
327	comp114797_c0_seq1.1.511.minus.R3_1	CE5	unclassified
328	comp115320_c0_seq1.1.580.minus.R1_1	CE1	unclassified
329	comp11559_c0_seq1.1.391.minus.R9_1	CE3	unclassified
330	comp115948_c0_seq1.1.376.minus.R8_1	CE1	unclassified
331	comp116094_c0_seq1.1.657.minus.R9_1	CE4	unclassified
332	comp116353_c0_seq1.24.560.plus.R7_1	CE3	unclassified
333	comp116644_c0_seq1.23.709.plus.R1_1	CE2	unclassified
334	comp117838_c0_seq1.53.498.minus.R1_1	CE1	unclassified
335	comp118186_c0_seq1.1.524.minus.R7_1	CE10	unclassified
336	comp11892_c0_seq2.1.489.minus.R9_1	CE3	unclassified

337	comp120278_c0_seq1.1.622.minus.R8_1	CE4	unclassified
338	comp121706_c0_seq1.54.701.plus.R8_1	CE9	unclassified
339	comp122291_c0_seq1.1.549.minus.R3_1	CE1	unclassified
340	comp122705_c0_seq1.116.505.plus.R3_1	CE1	unclassified
341	comp124339_c0_seq1.40.315.minus.R9_1	CE2	unclassified
342	comp124589_c0_seq1.1.407.minus.R1_1	CE10	unclassified
343	comp125154_c0_seq1.1.285.minus.R9_1	CE11	unclassified
344	comp129268_c0_seq1.1.376.minus.R7_1	CE2	unclassified
345	comp130319_c0_seq1.148.639.plus.R1_1	CE7	unclassified
346	comp130702_c0_seq1.1.525.minus.R8_1	CE11	unclassified
347	comp133614_c0_seq1.2.400.plus.R8_1	CE3	unclassified
348	comp134752_c0_seq1.1.325.minus.R1_1	CE3	unclassified
349	comp136772_c0_seq1.1.502.minus.R7_1	CE1	unclassified
350	comp13708_c0_seq1.1.651.minus.R1_1	CE1	unclassified
351	comp13754_c0_seq1.1.614.minus.R1_1	CE3	unclassified
352	comp137949_c0_seq1.1.438.minus.R8_1	CE4	unclassified
353	comp138629_c0_seq1.9.635.plus.R8_1	CE4	unclassified
354	comp138958_c0_seq1.1.661.minus.R1_1	CE9	unclassified
355	comp139636_c0_seq1.1.378.minus.R8_1	CE4	unclassified
356	comp140621_c0_seq1.1.300.minus.R1_1	CE1	unclassified
357	comp143115_c0_seq1.10.570.plus.R1_1	CE4	unclassified
358	comp14389_c0_seq1.56.676.minus.R8_1	CE1	unclassified
359	comp145664_c0_seq1.1.328.minus.R3_1	CE7	unclassified
360	comp145843_c0_seq1.29.553.plus.R8_1	CE10	unclassified
361	comp146510_c0_seq1.3.347.plus.R7_1	CE3	unclassified
362	comp14901_c0_seq1.1.597.minus.R3_1	CE12	unclassified
363	comp149083_c0_seq1.131.716.minus.R8_1	CE4	unclassified
364	comp149418_c0_seq1.1.304.minus.R1_1	CE3	unclassified
365	comp149935_c0_seq1.1.471.minus.R1_1	CE3	unclassified
366	comp150206_c0_seq1.1.457.minus.R7_1	CE1	unclassified
367	comp151192_c0_seq1.1.525.minus.R9_1	CE12	unclassified
368	comp154213_c0_seq1.1.363.minus.R1_1	CE4	unclassified
369	comp154477_c0_seq1.133.606.minus.R1_1	CE9	unclassified
370	comp155729_c0_seq1.1.364.minus.R7_1	CE9	unclassified
371	comp156812_c0_seq1.52.722.minus.R1_1	CE10	unclassified
372	comp15842_c0_seq1.1.398.minus.R7_1	CE9	unclassified
373	comp158607_c0_seq1.1.491.minus.R8_1	CE9	unclassified
374	comp159687_c0_seq1.1.375.minus.R8_1	CE4	unclassified
375	comp161618_c0_seq1.1.346.minus.R9_1	CE11	unclassified
376	comp163261_c0_seq1.1.398.minus.R1_1	CE4	unclassified
377	comp163736_c0_seq1.1.684.minus.R8_1	CE7	unclassified
378	comp164732_c0_seq1.1.416.minus.R1_1	CE11	unclassified
379	comp165597_c0_seq1.1.382.minus.R1_1	CE9	unclassified

380	comp165621_c0_seq1.29.352.minus.R9_1	CE12	unclassified
381	comp17080_c0_seq1.1.378.minus.R8_1	CE1	unclassified
382	comp17392_c0_seq1.1.429.minus.R3_1	CE4	unclassified
383	comp175004_c0_seq1.72.407.plus.R1_1	CE1	unclassified
384	comp17908_c0_seq1.1.657.minus.R1_1	CE9	unclassified
385	comp179432_c0_seq1.1.288.minus.R1_1	CE11	unclassified
386	comp181248_c0_seq1.1.436.minus.R8_1	CE4	unclassified
387	comp181368_c0_seq1.1.448.minus.R7_1	CE7	unclassified
388	comp18430_c0_seq1.1.383.minus.R8_1	CE1	unclassified
389	comp184962_c0_seq1.1.213.minus.R3_1	CE4	unclassified
390	comp189130_c0_seq1.1.423.minus.R8_1	CE3	unclassified
391	comp191141_c0_seq1.14.476.minus.R1_1	CE4	unclassified
392	comp191184_c0_seq1.1.439.minus.R1_1	CE6	unclassified
393	comp197947_c0_seq1.1.339.minus.R8_1	CE1	unclassified
394	comp198749_c0_seq1.1.382.minus.R1_1	CE11	unclassified
395	comp199009_c0_seq1.1.312.minus.R8_1	CE12	unclassified
396	comp202150_c0_seq1.1.373.minus.R8_1	CE1	unclassified
397	comp203353_c0_seq1.1.367.minus.R3_1	CE4	unclassified
398	comp205172_c0_seq1.1.459.minus.R9_1	CE10	unclassified
399	comp207789_c0_seq1.1.339.minus.R9_1	CE12	unclassified
400	comp20797_c0_seq1.10.531.plus.R7_1	CE3	unclassified
401	comp210010_c0_seq1.1.422.minus.R8_1	CE11	unclassified
402	comp211382_c0_seq1.1.388.minus.R1_1	CE3	unclassified
403	comp212028_c0_seq1.1.380.minus.R1_1	CE9	unclassified
404	comp213495_c0_seq1.1.595.minus.R1_1	CE8	unclassified
405	comp214432_c0_seq1.8.393.minus.R7_1	CE3	unclassified
406	comp215347_c0_seq1.1.502.minus.R3_1	CE12	unclassified
407	comp21674_c0_seq1.1.460.minus.R1_1	CE2	unclassified
408	comp217896_c0_seq1.1.333.minus.R8_1	CE1	unclassified
409	comp219253_c0_seq1.9.575.minus.R7_1	CE8	unclassified
410	comp219292_c0_seq1.5.411.minus.R1_1	CE9	unclassified
411	comp22167_c0_seq1.1.516.minus.R1_1	CE14	unclassified
412	comp22167_c0_seq2.1.649.minus.R1_1	CE14	unclassified
413	comp222207_c0_seq1.1.491.minus.R1_1	CE6	unclassified
414	comp226661_c0_seq1.1.324.minus.R7_1	CE11	unclassified
415	comp228445_c0_seq1.1.309.minus.R8_1	CE4	unclassified
416	comp228934_c0_seq1.1.425.minus.R8_1	CE12	unclassified
417	comp23010_c0_seq1.72.698.plus.R9_1	CE1	unclassified
418	comp2336_c0_seq1.1.419.minus.R3_1	CE1	unclassified
419	comp233986_c0_seq1.1.367.minus.R1_1	CE4	unclassified
420	comp236434_c0_seq1.1.593.minus.R1_1	CE3	unclassified
421	comp23798_c0_seq1.1.503.minus.R1_1	CE2	unclassified
422	comp238409_c0_seq1.1.425.minus.R1_1	CE1	unclassified

423	comp239070_c0_seq1.1.364.minus.R3_1	CE4	unclassified
424	comp23910_c0_seq1.1.343.minus.R8_1	CE3	unclassified
425	comp240095_c0_seq1.1.371.minus.R8_1	CE9	unclassified
426	comp240364_c0_seq1.1.331.minus.R1_1	CE3	unclassified
427	comp24254_c0_seq1.1.829.minus.R7_1	CE3	unclassified
428	comp247471_c0_seq1.1.556.minus.R1_1	CE6	unclassified
429	comp24989_c0_seq1.1.616.minus.R7_1	CE6	unclassified
430	comp24989_c0_seq2.1.620.minus.R7_1	CE6	unclassified
431	comp251329_c0_seq1.1.311.minus.R7_1	CE1	unclassified
432	comp255828_c0_seq1.1.325.minus.R1_1	CE1	unclassified
433	comp258130_c0_seq1.1.380.minus.R1_1	CE9	unclassified
434	comp262690_c0_seq1.25.384.minus.R8_1	CE12	unclassified
435	comp26273_c0_seq1.333.948.minus.R9_1	CE10	unclassified
436	comp26310_c0_seq1.4.423.minus.R3_1	CE3	unclassified
437	comp265616_c0_seq1.1.330.minus.R3_1	CE8	unclassified
438	comp266295_c0_seq1.1.469.minus.R8_1	CE9	unclassified
439	comp266884_c0_seq1.22.533.minus.R1_1	CE1	unclassified
440	comp278925_c0_seq1.1.357.minus.R1_1	CE14	unclassified
441	comp284063_c0_seq1.1.439.minus.R1_1	CE4	unclassified
442	comp287425_c0_seq1.1.336.minus.R3_1	CE1	unclassified
443	comp29763_c0_seq2.1.425.minus.R3_1	CE11	unclassified
444	comp30455_c0_seq1.108.478.minus.R3_1	CE3	unclassified
445	comp31146_c0_seq1.1.552.minus.R8_1	CE9	unclassified
446	comp315482_c0_seq1.6.303.minus.R8_1	CE12	unclassified
447	comp321584_c0_seq1.1.326.minus.R8_1	CE1	unclassified
448	comp322687_c0_seq1.13.328.minus.R8_1	CE2	unclassified
449	comp324233_c0_seq1.1.342.minus.R1_1	CE1	unclassified
450	comp32782_c0_seq3.1.583.minus.R1_1	CE1	unclassified
451	comp32908_c0_seq1.1.362.minus.R8_1	CE9	unclassified
452	comp33237_c0_seq8.14.469.plus.R3_1	CE1	unclassified
453	comp332893_c0_seq1.1.443.minus.R1_1	CE10	unclassified
454	comp33402_c0_seq1.35.617.minus.R7_1	CE10	unclassified
455	comp33581_c0_seq2.1.526.minus.R3_1	CE14	unclassified
456	comp340633_c0_seq1.1.312.minus.R1_1	CE2	unclassified
457	comp340880_c0_seq1.1.314.minus.R1_1	CE4	unclassified
458	comp359608_c0_seq1.1.347.minus.R7_1	CE10	unclassified
459	comp36092_c0_seq1.1.600.minus.R9_1	CE4	unclassified
460	comp36092_c0_seq3.1.395.minus.R9_1	CE4	unclassified
461	comp36128_c0_seq19.26.652.plus.R3_1	CE10	unclassified
462	comp36906_c0_seq1.152.761.minus.R8_1	CE10	unclassified
463	comp37443_c0_seq1.1.664.minus.R7_1	CE14	unclassified
464	comp37607_c0_seq1.133.570.plus.R9_1	CE1	unclassified
465	comp377228_c0_seq1.1.314.minus.R8_1	CE11	unclassified

466	comp38245_c0_seq1.1.406.minus.R8_1	CE11	unclassified
467	comp386152_c0_seq1.1.417.minus.R1_1	CE10	unclassified
468	comp39112_c0_seq2.30.443.plus.R9_1	CE4	unclassified
469	comp398290_c0_seq1.1.338.minus.R1_1	CE1	unclassified
470	comp42945_c0_seq1.185.1980.minus.R1_1	CE3	unclassified
471	comp44267_c0_seq1.35.607.plus.R3_1	CE4	unclassified
472	comp44267_c0_seq2.1.415.minus.R8_1	CE1	unclassified
473	comp44583_c0_seq1.1.308.minus.R1_1	CE8	unclassified
474	comp448200_c0_seq1.1.370.minus.R1_1	CE4	unclassified
475	comp448931_c0_seq1.1.489.minus.R1_1	CE4	unclassified
476	comp45084_c0_seq2.1.538.minus.R1_1	CE1	unclassified
477	comp45084_c0_seq3.1.388.minus.R1_1	CE1	unclassified
478	comp45724_c0_seq1.1.376.minus.R1_1	CE3	unclassified
479	comp46119_c0_seq1.4.462.plus.R8_1	CE3	unclassified
480	comp46237_c0_seq1.8.454.plus.R7_1	CE3	unclassified
481	comp47649_c0_seq1.1.543.minus.R9_1	CE1	unclassified
482	comp47924_c0_seq2.99.596.plus.R8_1	CE4	unclassified
483	comp48529_c0_seq1.145.727.minus.R3_1	CE8	unclassified
484	comp50845_c0_seq1.7.507.plus.R7_1	CE10	unclassified
485	comp52512_c0_seq1.548.1105.plus.R3_1	CE14	unclassified
486	comp53803_c0_seq1.1.426.minus.R3_1	CE14	unclassified
487	comp54907_c0_seq1.619.1176.plus.R7_1	CE3	unclassified
488	comp54934_c0_seq1.68.532.minus.R1_1	CE3	unclassified
489	comp55021_c0_seq7.30.655.minus.R7_1	CE9	unclassified
490	comp55776_c0_seq1.5.517.plus.R9_1	CE9	unclassified
491	comp58071_c0_seq1.29.652.plus.R1_1	CE1	unclassified
492	comp58071_c0_seq2.29.478.plus.R1_1	CE1	unclassified
493	comp58436_c0_seq1.66.685.minus.R1_1	CE10	unclassified
494	comp58436_c0_seq2.652.1247.minus.R1_1	CE10	unclassified
495	comp58920_c0_seq1.1.334.minus.R9_1	CE1	unclassified
496	comp5892_c0_seq1.1.693.minus.R9_1	CE10	unclassified
497	comp60556_c0_seq2.5.661.plus.R1_1	CE1	unclassified
498	comp62446_c0_seq1.28.723.plus.R1_1	CE3	unclassified
499	comp62565_c0_seq7.1475.2137.plus.R1_1	CE4	unclassified
500	comp62691_c0_seq14.1832.2440.plus.R1_1	CE4	unclassified
501	comp62691_c0_seq3.3448.4059.plus.R1_1	CE4	unclassified
502	comp64813_c0_seq1.80.770.minus.R9_1	CE9	unclassified
503	comp651028_c0_seq1.1.301.minus.R1_1	CE8	unclassified
504	comp70292_c0_seq1.1.517.minus.R1_1	CE9	unclassified
505	comp73999_c0_seq1.451.1097.minus.R3_1	CE8	unclassified
506	comp76275_c0_seq1.7.666.minus.R7_1	CE14	unclassified
507	comp76653_c0_seq1.1.626.minus.R3_1	CE1	unclassified
508	comp77108_c0_seq1.5.553.plus.R3_1	CE1	unclassified

509	comp7729_c0_seq1.1.578.minus.R8_1	CE1	unclassified
510	comp78000_c0_seq1.42.366.minus.R3_1	CE12	unclassified
511	comp78451_c0_seq1.1.692.minus.R3_1	CE1	unclassified
512	comp81561_c0_seq1.1.453.minus.R3_1	CE10	unclassified
513	comp81816_c0_seq1.7.405.plus.R7_1	CE3	unclassified
514	comp81924_c0_seq1.7.1011.plus.R8_1	CE3	unclassified
515	comp82078_c0_seq1.1.355.minus.R9_1	CE9	unclassified
516	comp82572_c0_seq1.1.351.minus.R9_1	CE8	unclassified
517	comp8346_c0_seq1.1.383.minus.R7_1	CE10	unclassified
518	comp84781_c0_seq1.25.555.plus.R3_1	CE2	unclassified
519	comp85002_c0_seq1.1.338.minus.R3_1	CE8	unclassified
520	comp8636_c0_seq1.1.446.minus.R8_1	CE1	unclassified
521	comp87174_c0_seq1.38.847.plus.R1_1	CE12	unclassified
522	comp87877_c0_seq1.1.427.minus.R9_1	CE6	unclassified
523	comp87_c0_seq1.1.427.minus.R9_1	CE4	unclassified
524	comp8867_c0_seq2.4.657.plus.R3_1	CE14	unclassified
525	comp89521_c0_seq1.1.588.minus.R8_1	CE1	unclassified
526	comp90053_c0_seq1.1.499.minus.R7_1	CE9	unclassified
527	comp90319_c0_seq1.1.684.minus.R1_1	CE2	unclassified
528	comp90462_c0_seq1.1.476.minus.R1_1	CE12	unclassified
529	comp91757_c0_seq1.6.308.plus.R3_1	CE3	unclassified
530	comp92195_c0_seq1.8.675.minus.R1_1	CE3	unclassified
531	comp93874_c0_seq1.1.406.minus.R9_1	CE4	unclassified
532	comp95804_c0_seq1.28.714.plus.R1_1	CE3	unclassified
533	comp96587_c0_seq1.74.619.plus.R3_1	CE3	unclassified
534	comp98729_c0_seq1.1.496.minus.R1_1	CE1	unclassified
535	comp99161_c0_seq1.1.353.minus.R3_1	CE4	unclassified
536	comp99268_c0_seq1.1.688.minus.R1_1	CE3	unclassified
