# Supplementary material for: Effects of dietary supplementation with lysozyme on the structure and function of the cecal microbiota in broiler chickens
Source: PLoS One. 2019 Jun 19;14(6):e0216748. doi: 10.1371/journal.pone.0216748 (PMC6583987; doi:10.1371/journal.pone.0216748)
Supplement: S6 Table — (PDF) [file pone.0216748.s006.pdf]

S6 Table. Taxonomy of carbohydrate-binding modules (CBM) genes identified in the cecal microbiota of broilers fed a corn-based diet supplemented with 0 (R1 in gene query name), 40 (R7 in gene query name), 100 (R8 in gene query name), or 200 ppm (R9 in gene query name) lysozyme or 400 ppm flavomycin (R3 in gene query name) [the gene names in query refer to those in the transcriptome dataset deposited as PRJNA523864 in NCBI Sequence Read Archive].

No	Gene query	Family	Taxonomy
1	comp27558_c0_seq1.26.2039.minus.R1_1	CBM32	*Acidobacteriaceae*_bacterium_KBS_96
2	comp27558_c0_seq1.26.2039.minus.R1_1	CBM32	*Acidobacteriaceae*_bacterium_KBS_96
3	comp49558_c0_seq2.684.4757.plus.R8_1	CBM56	*Bifidobacterium_bifidum*
4	comp67782_c0_seq1.35.1081.plus.R1_1	CBM50	*Arthrobacter*_sp._H20
5	comp67782_c0_seq1.35.1081.plus.R1_1	CBM50	*Arthrobacter*_sp._H20
6	comp67782_c0_seq1.35.1081.plus.R1_1	CBM50	*Arthrobacter*_sp._H20
7	comp78244_c0_seq1.1.2535.minus.R1_1	CBM32	*Collinsella_tanakaei*
8	comp78244_c0_seq1.1.2535.minus.R1_1	CBM32	*Collinsella_tanakaei*
9	comp131308_c0_seq1.53.1156.plus.R1_1	CBM32	*Bacteroides_barnesiae*
10	comp26298_c0_seq1.48.859.minus.R3_1	CBM16	*Bacteroides_barnesiae*
11	comp36462_c0_seq2.50.1408.plus.R3_1	CBM32	*Bacteroides_barnesiae*
12	comp38623_c0_seq1.50.814.plus.R9_1	CBM50	*Bacteroides_barnesiae*
13	comp39834_c0_seq1.28.1552.minus.R9_1	CBM32	*Bacteroides_barnesiae*
14	comp54358_c0_seq2.3921.5153.plus.R1_1	CBM48	*Bacteroides_barnesiae*
15	comp55836_c0_seq1.138.1367.plus.R8_1	CBM48	*Bacteroides_barnesiae*
16	comp9582_c0_seq1.2.937.plus.R3_1	CBM50	*Bacteroides_barnesiae*
17	comp23514_c0_seq1.75.2777.minus.R9_1	CBM20	*Bacteroides_barnesiae*
18	comp23514_c0_seq1.75.2777.minus.R9_1	CBM20	*Bacteroides_barnesiae*
19	comp48717_c0_seq2.277.2609.minus.R8_1	CBM32	*Bacteroides_barnesiae*
20	comp48717_c0_seq7.277.2622.minus.R8_1	CBM32	*Bacteroides_barnesiae*
21	comp100936_c0_seq1.31.909.minus.R1_1	CBM32	*Bacteroides_coprocola*
22	comp100936_c0_seq1.31.909.minus.R1_1	CBM32	*Bacteroides_coprocola*
23	comp31956_c0_seq2.6563.7726.plus.R3_1	CBM40	*Bacteroides_coprocola*
24	comp32455_c0_seq1.38.907.plus.R3_1	CBM50	*Bacteroides_coprocola*
25	comp33857_c0_seq1.6079.8076.plus.R3_1	CBM48	*Bacteroides_coprocola*
26	comp53999_c0_seq1.6046.7206.plus.R1_1	CBM40	*Bacteroides_coprocola*
27	comp10236_c0_seq1.25.2742.minus.R3_1	CBM20	*Bacteroides_coprocola*
28	comp57639_c0_seq3.905.3244.minus.R1_1	CBM32	*Bacteroides_coprocola*
29	comp61014_c0_seq4.448.2784.plus.R1_1	CBM32	*Bacteroides_coprocola*
30	comp61014_c0_seq8.79.2346.plus.R1_1	CBM32	*Bacteroides_coprocola*
31	comp27990_c0_seq1.7825.9753.plus.R1_1	CBM62	*Bacteroides_coprocola*_CAG
32	comp53999_c0_seq1.7275.8219.plus.R1_1	CBM32	*Bacteroides_coprocola*_CAG
33	comp53999_c0_seq3.2545.4602.plus.R1_1	CBM32	*Bacteroides_coprocola*_CAG
34	comp63824_c0_seq1.6408.7604.plus.R1_1	CBM48	*Bacteroides_coprocola*_CAG
35	comp32526_c0_seq1.1359.2420.plus.R1_1	CBM9	*Bacteroides_coprophilus*
36	comp35942_c0_seq1.491.1822.minus.R9_1	CBM50	*Bacteroides_coprophilus*

37	comp47591_c0_seq2.108.1034.plus.R1_1	CBM50	*Bacteroides_coprophilus*
38	comp51619_c0_seq2.1.819.minus.R7_1	CBM48	*Bacteroides_coprophilus*
39	comp51619_c0_seq6.710.2722.minus.R7_1	CBM48	*Bacteroides_coprophilus*
40	comp52203_c0_seq2.2637.3737.plus.R7_1	CBM50	*Bacteroides_coprophilus*
41	comp61052_c0_seq2.2545.3801.plus.R1_1	CBM50	*Bacteroides_coprophilus*
42	comp91163_c0_seq1.104.1112.minus.R7_1	CBM35	*Bacteroides_coprophilus*
43	comp9950_c0_seq1.65.1980.minus.R8_1	CBM32	*Bacteroides_coprophilus*
44	comp9950_c0_seq1.65.1980.minus.R8_1	CBM32	*Bacteroides_coprophilus*
45	comp54731_c0_seq8.1079.3415.plus.R7_1	CBM32	*Bacteroides_coprophilus*
46	comp60626_c0_seq1.846.3084.minus.R8_1	CBM32	*Bacteroides_coprophilus*
47	comp83850_c0_seq1.133.2292.plus.R1_1	CBM32	*Bacteroides_coprophilus*
48	comp83850_c0_seq1.133.2292.plus.R1_1	CBM32	*Bacteroides_coprophilus*
49	comp16435_c0_seq1.51.890.plus.R7_1	CBM50	*Bacteroides_coprophilus*_CAG
50	comp35965_c0_seq5.5889.6842.plus.R3_1	CBM50	*Bacteroides_coprophilus*_CAG
51	comp75953_c0_seq1.1828.3927.minus.R1_1	CBM67	*Bacteroides_coprophilus*_CAG
52	comp92250_c0_seq1.18.814.minus.R7_1	CBM32	*Bacteroides_coprophilus*_CAG
53	comp18468_c0_seq1.62.2776.plus.R1_1	CBM32	*Bacteroides_coprophilus*_CAG
54	comp33576_c0_seq1.2049.4330.minus.R1_1	CBM32	*Bacteroides_coprophilus*_CAG
55	comp33576_c0_seq1.2049.4330.minus.R1_1	CBM32	*Bacteroides_coprophilus*_CAG
56	comp49895_c0_seq9.575.4174.plus.R8_1	CBM13	*Bacteroides_coprophilus*_CAG
57	comp68993_c0_seq1.75.3911.minus.R7_1	CBM32	*Bacteroides_coprophilus*_CAG
58	comp74036_c0_seq1.428.2617.plus.R7_1	CBM20	*Bacteroides_coprophilus*_CAG
59	comp62789_c0_seq7.1.1375.minus.R1_1	CBM62	*Bacteroides_dorei*
60	comp10907_c0_seq1.139.1653.plus.R1_1	CBM35	*Bacteroides_faecichinchillae*
61	comp15989_c0_seq1.135.878.minus.R1_1	CBM32	*Bacteroides_faecichinchillae*
62	comp71320_c0_seq1.51.926.minus.R1_1	CBM50	*Bacteroides_faecichinchillae*
63	comp80281_c0_seq1.1.1350.minus.R1_1	CBM67	*Bacteroides_faecichinchillae*
64	comp54681_c0_seq1.1159.4758.minus.R1_1	CBM32	*Bacteroides_faecis*
65	comp55320_c0_seq1.5302.6645.plus.R7_1	CBM32	*Bacteroides_fluxus*
66	comp102946_c0_seq1.1.1413.minus.R1_1	CBM32	*Bacteroides_fragilis*
67	comp109059_c0_seq1.1.1425.minus.R3_1	CBM32	*Bacteroides_fragilis*
68	comp129355_c0_seq1.1.985.minus.R7_1	CBM32	*Bacteroides_fragilis*
69	comp62153_c0_seq2.4937.6169.plus.R1_1	CBM53	*Bacteroides_gallinarum*
70	comp79393_c0_seq1.35.739.plus.R1_1	CBM50	*Bacteroides_gallinarum*
71	comp112292_c0_seq1.29.736.plus.R8_1	CBM40	*Bacteroides_oleiciplenus*
72	comp66361_c0_seq1.1.795.minus.R3_1	CBM4	*Bacteroides_oleiciplenus*
73	comp44581_c0_seq1.1.1298.minus.R1_1	CBM66	*Bacteroides_ovatus*
74	comp164250_c0_seq1.1.1340.minus.R1_1	CBM66	*Bacteroides_plebeius*
75	comp44003_c0_seq1.1885.3692.minus.R3_1	CBM50	*Bacteroides_plebeius*
76	comp44003_c0_seq1.1885.3692.minus.R3_1	CBM50	*Bacteroides_plebeius*
77	comp44003_c0_seq1.1885.3692.minus.R3_1	CBM50	*Bacteroides_plebeius*
78	comp45907_c0_seq1.463.2364.plus.R1_1	CBM16	*Bacteroides_plebeius*
79	comp54015_c0_seq1.543.1323.minus.R7_1	CBM16	*Bacteroides_plebeius*
80	comp40367_c0_seq1.11089.13350.plus.R7_1	CBM4	*Bacteroides_plebeius*

81	comp40367_c0_seq1.11089.13350.plus.R7_1	CBM4	*Bacteroides_plebeius*
82	comp30122_c0_seq2.70.1752.plus.R3_1	CBM32	*Bacteroides_plebeius*_CAG
83	comp55088_c0_seq2.1318.3366.minus.R1_1	CBM9	*Bacteroides_plebeius*_CAG
84	comp61052_c0_seq1.394.1659.plus.R1_1	CBM50	*Bacteroides_plebeius*_CAG
85	comp61103_c0_seq2.1.1501.minus.R1_1	CBM66	*Bacteroides_plebeius*_CAG
86	comp44608_c0_seq1.94.2684.minus.R1_1	CBM32	*Bacteroides_plebeius*_CAG
87	comp62666_c0_seq7.2076.5657.plus.R1_1	CBM13	*Bacteroides_plebeius*_CAG
88	comp51511_c0_seq1.1.993.minus.R7_1	CBM48	*Bacteroides_salanitronis*
89	comp58105_c0_seq1.4253.5509.plus.R1_1	CBM23	*Bacteroides_salanitronis*
90	comp61174_c0_seq1.1.1400.minus.R8_1	CBM20	*Bacteroides_salanitronis*
91	comp68970_c0_seq1.21.1208.plus.R1_1	CBM48	*Bacteroides_salanitronis*
92	comp31729_c0_seq1.128.2818.minus.R1_1	CBM20	*Bacteroides_salanitronis*
93	comp56853_c0_seq2.41.5296.minus.R1_1	CBM56	*Bacteroides_salyersiae*
94	comp56853_c0_seq2.41.5296.minus.R1_1	CBM56	*Bacteroides_salyersiae*
95	comp83494_c0_seq1.4887.6215.plus.R1_1	CBM32	*Bacteroides*_sp._1_1_6
96	comp40365_c0_seq5.6.2027.plus.R9_1	CBM48	*Bacteroides*_sp._2_1_33B
97	comp89523_c0_seq1.1.1098.minus.R9_1	CBM67	*Bacteroides*_sp._2_1_33B
98	comp61019_c0_seq2.73.2247.plus.R1_1	CBM32	*Bacteroides*_sp._2_1_33B
99	comp52488_c0_seq1.37.1212.plus.R1_1	CBM66	*Bacteroides*_sp._3_1_13
100	comp52488_c0_seq2.902.2254.minus.R1_1	CBM66	*Bacteroides*_sp._3_1_13
101	comp103892_c0_seq1.1.962.minus.R8_1	CBM22	*Bacteroides*_sp._CAG
102	comp106369_c0_seq1.573.1362.minus.R8_1	CBM32	Bacteroides_sp._CAG
103	comp106369_c0_seq1.573.1362.minus.R8_1	CBM32	*Bacteroides*_sp._CAG
104	comp11669_c0_seq1.93.2090.minus.R1_1	CBM48	*Bacteroides*_sp._CAG
105	comp117716_c0_seq1.1.715.minus.R7_1	CBM32	*Bacteroides*_sp._CAG
106	comp11796_c0_seq1.199.981.plus.R9_1	CBM16	*Bacteroides*_sp._CAG
107	comp12131_c0_seq1.1.2002.minus.R7_1	CBM77	*Bacteroides*_sp._CAG
108	comp121491_c0_seq1.1.778.minus.R7_1	CBM66	*Bacteroides*_sp._CAG
109	comp20901_c0_seq1.68.1429.plus.R3_1	CBM6	*Bacteroides*_sp._CAG
110	comp22382_c0_seq1.14.2014.plus.R3_1	CBM48	*Bacteroides*_sp._CAG
111	comp26733_c0_seq1.11.1862.minus.R3_1	CBM16	*Bacteroides*_sp._CAG
112	comp26778_c0_seq1.1.867.minus.R3_1	CBM50	*Bacteroides*_sp._CAG
113	comp28550_c0_seq1.48.1244.minus.R3_1	CBM48	*Bacteroides*_sp._CAG
114	comp30468_c0_seq1.94.1410.plus.R9_1	CBM32	*Bacteroides*_sp._CAG
115	comp33676_c0_seq1.29.1135.minus.R9_1	CBM48	*Bacteroides*_sp._CAG
116	comp34826_c0_seq1.299.1081.plus.R3_1	CBM16	*Bacteroides*_sp._CAG
117	comp35042_c0_seq1.42.1238.minus.R9_1	CBM48	*Bacteroides*_sp._CAG
118	comp36098_c0_seq1.14.2164.plus.R3_1	CBM32	*Bacteroides*_sp._CAG
119	comp36254_c0_seq2.530.2182.plus.R3_1	CBM44	*Bacteroides*_sp._CAG
120	comp36462_c0_seq3.21.1364.plus.R3_1	CBM32	*Bacteroides*_sp._CAG
121	comp36462_c0_seq4.29.1615.plus.R3_1	CBM32	*Bacteroides*_sp._CAG
122	comp41005_c0_seq1.1178.3190.plus.R3_1	CBM48	*Bacteroides*_sp._CAG
123	comp44988_c0_seq1.15.1382.plus.R3_1	CBM6	*Bacteroides*_sp._CAG
124	comp45309_c0_seq1.33.902.minus.R7_1	CBM50	*Bacteroides*_sp._CAG

125	comp46016_c0_seq1.2.862.plus.R3_1	CBM32	*Bacteroides*_sp._CAG
126	comp49059_c0_seq6.1.1428.minus.R8_1	CBM32	*Bacteroides*_sp._CAG
127	comp50931_c0_seq1.815.2005.minus.R3_1	CBM48	*Bacteroides*_sp._CAG
128	comp54730_c0_seq3.10.1959.minus.R7_1	CBM32	*Bacteroides*_sp._CAG
129	comp54731_c0_seq9.20.1339.plus.R7_1	CBM32	*Bacteroides*_sp._CAG
130	comp55144_c0_seq6.1.1137.minus.R7_1	CBM13	*Bacteroides*_sp._CAG
131	comp55795_c0_seq1.70.939.plus.R3_1	CBM50	*Bacteroides*_sp._CAG
132	comp56426_c0_seq1.219.1134.minus.R3_1	CBM32	*Bacteroides*_sp._CAG
133	comp57624_c0_seq1.32.1182.minus.R8_1	CBM32	*Bacteroides*_sp._CAG
134	comp57976_c0_seq4.19.2013.plus.R1_1	CBM48	*Bacteroides*_sp._CAG
135	comp59633_c0_seq2.41.2024.minus.R1_1	CBM50	*Bacteroides*_sp._CAG
136	comp59633_c0_seq2.41.2024.minus.R1_1	CBM50	*Bacteroides*_sp._CAG
137	comp59633_c0_seq2.41.2024.minus.R1_1	CBM50	*Bacteroides*_sp._CAG
138	comp60916_c0_seq4.973.2925.plus.R1_1	CBM48	*Bacteroides*_sp._CAG
139	comp61007_c0_seq1.816.2617.minus.R1_1	CBM50	*Bacteroides*_sp._CAG
140	comp61007_c0_seq1.816.2617.minus.R1_1	CBM50	*Bacteroides*_sp._CAG
141	comp61007_c0_seq1.816.2617.minus.R1_1	CBM50	*Bacteroides*_sp._CAG
142	comp61176_c0_seq1.1178.3187.plus.R1_1	CBM48	*Bacteroides*_sp._CAG
143	comp61431_c0_seq1.60.2135.plus.R1_1	CBM6	*Bacteroides*_sp._CAG
144	comp61745_c0_seq2.94.1257.minus.R1_1	CBM50	*Bacteroides*_sp._CAG
145	comp61745_c0_seq4.94.1218.minus.R1_1	CBM50	*Bacteroides*_sp._CAG
146	comp61810_c0_seq5.2939.4948.minus.R1_1	CBM48	*Bacteroides*_sp._CAG
147	comp62328_c0_seq10.2460.4577.minus.R1_1	CBM48	*Bacteroides*_sp._CAG
148	comp62511_c0_seq1.926.2932.plus.R1_1	CBM48	*Bacteroides*_sp._CAG
149	comp62511_c0_seq3.926.2932.plus.R1_1	CBM48	*Bacteroides*_sp._CAG
150	comp62629_c0_seq1.1197.3101.plus.R1_1	CBM32	*Bacteroides*_sp._CAG
151	comp62629_c0_seq1.1197.3101.plus.R1_1	CBM32	*Bacteroides*_sp._CAG
152	comp62859_c0_seq1.6566.7423.plus.R1_1	CBM32	*Bacteroides*_sp._CAG
153	comp62915_c0_seq5.259.2138.minus.R1_1	CBM32	*Bacteroides*_sp._CAG
154	comp62915_c0_seq5.259.2138.minus.R1_1	CBM32	*Bacteroides*_sp._CAG
155	comp62930_c0_seq3.2719.3999.plus.R1_1	CBM50	*Bacteroides*_sp._CAG
156	comp62992_c0_seq2.15.2069.plus.R1_1	CBM32	*Bacteroides*_sp._CAG
157	comp63112_c0_seq1.5405.7219.plus.R1_1	CBM32	*Bacteroides*_sp._CAG
158	comp63453_c0_seq1.9283.11259.plus.R1_1	CBM16	*Bacteroides*_sp._CAG
159	comp64152_c0_seq1.627.1577.minus.R1_1	CBM50	*Bacteroides*_sp._CAG
160	comp68007_c0_seq1.1.1158.minus.R3_1	CBM32	*Bacteroides*_sp._CAG
161	comp72025_c0_seq1.500.1360.plus.R1_1	CBM50	*Bacteroides*_sp._CAG
162	comp77683_c0_seq1.1.1017.minus.R8_1	CBM48	*Bacteroides*_sp._CAG
163	comp82145_c0_seq1.153.2161.minus.R1_1	CBM32	*Bacteroides*_sp._CAG
164	comp82145_c0_seq1.153.2161.minus.R1_1	CBM32	*Bacteroides*_sp._CAG
165	comp82166_c0_seq1.39.896.plus.R8_1	CBM50	*Bacteroides*_sp._CAG
166	comp82288_c0_seq1.489.1751.plus.R1_1	CBM23	*Bacteroides*_sp._CAG
167	comp85398_c0_seq1.10.1329.plus.R1_1	CBM6	*Bacteroides*_sp._CAG
168	comp88898_c0_seq1.77.907.plus.R3_1	CBM49	*Bacteroides*_sp._CAG

169	comp9279_c0_seq1.1.1561.minus.R3_1	CBM16	*Bacteroides*_sp._CAG
170	comp96081_c0_seq1.1.1181.minus.R1_1	CBM48	*Bacteroides*_sp._CAG
171	comp18390_c0_seq1.1.2692.minus.R1_1	CBM67	*Bacteroides*_sp._CAG
172	comp25084_c0_seq1.3.2906.minus.R3_1	CBM56	*Bacteroides*_sp._CAG
173	comp25826_c0_seq1.3687.7871.plus.R3_1	CBM35	*Bacteroides*_sp._CAG
174	comp25826_c0_seq1.3687.7871.plus.R3_1	CBM57	*Bacteroides*_sp._CAG
175	comp35402_c0_seq2.17.2356.minus.R3_1	CBM32	*Bacteroides*_sp._CAG
176	comp38883_c0_seq1.1760.4101.minus.R9_1	CBM32	*Bacteroides*_sp._CAG
177	comp41112_c0_seq2.237.2916.minus.R1_1	CBM20	*Bacteroides*_sp._CAG
178	comp47462_c0_seq1.1661.4119.minus.R8_1	CBM35	*Bacteroides*_sp._CAG
179	comp48717_c0_seq1.46.2366.minus.R8_1	CBM32	*Bacteroides*_sp._CAG
180	comp48717_c0_seq6.46.2379.minus.R8_1	CBM32	Bacteroides_sp._CAG
181	comp49895_c0_seq1.575.4177.plus.R8_1	CBM13	*Bacteroides*_sp._CAG
182	comp49984_c0_seq2.430.3132.minus.R8_1	CBM20	*Bacteroides*_sp._CAG
183	comp49984_c0_seq2.430.3132.minus.R8_1	CBM20	*Bacteroides*_sp._CAG
184	comp52518_c0_seq1.170.2557.plus.R7_1	CBM32	*Bacteroides*_sp._CAG
185	comp54291_c0_seq3.6.3332.plus.R7_1	CBM32	*Bacteroides*_sp._CAG
186	comp54401_c0_seq3.8.2332.plus.R7_1	CBM32	*Bacteroides*_sp._CAG
187	comp54401_c0_seq4.46.2322.plus.R7_1	CBM32	*Bacteroides*_sp._CAG
188	comp57570_c0_seq1.30.2367.minus.R8_1	CBM32	*Bacteroides*_sp._CAG
189	comp60062_c0_seq12.1495.4005.plus.R1_1	CBM32	*Bacteroides*_sp._CAG
190	comp60062_c0_seq12.1495.4005.plus.R1_1	CBM32	*Bacteroides*_sp._CAG
191	comp60505_c0_seq1.25.2853.minus.R1_1	CBM32	*Bacteroides*_sp._CAG
192	comp61014_c0_seq2.71.2377.plus.R1_1	CBM32	*Bacteroides*_sp._CAG
193	comp61356_c0_seq1.31.2539.minus.R1_1	CBM32	*Bacteroides*_sp._CAG
194	comp61732_c0_seq2.19.2691.plus.R1_1	CBM20	*Bacteroides*_sp._CAG
195	comp61821_c0_seq11.2234.4588.minus.R1_1	CBM32	*Bacteroides*_sp._CAG
196	comp62344_c1_seq1.961.3726.minus.R1_1	CBM20	*Bacteroides*_sp._CAG
197	comp62344_c1_seq1.961.3726.minus.R1_1	CBM20	*Bacteroides*_sp._CAG
198	comp62915_c0_seq6.940.4542.minus.R1_1	CBM13	*Bacteroides*_sp._CAG
199	comp62992_c0_seq1.5978.9583.plus.R1_1	CBM32	*Bacteroides*_sp._CAG
200	comp62992_c0_seq1.5978.9583.plus.R1_1	CBM32	*Bacteroides*_sp._CAG
201	comp69879_c0_seq1.9.2910.minus.R7_1	CBM40	*Bacteroides*_sp._CAG
202	comp76766_c0_seq1.19.2400.plus.R1_1	CBM32	*Bacteroides*_sp._CAG
203	comp10030_c0_seq1.22.2401.minus.R7_1	CBM40	*Bacteroides*_sp._HPS0048
204	comp52869_c0_seq1.1.738.minus.R1_1	CBM26	*Bacteroides*_sp._HPS0048
205	comp72914_c0_seq1.5.1565.minus.R1_1	CBM40	*Bacteroides*_sp._HPS0048
206	comp55776_c0_seq2.1419.2948.plus.R7_1	CBM32	*Bacteroides*_sp._I48
207	comp31699_c0_seq1.1.2684.minus.R1_1	CBM67	*Bacteroides*_sp._I48
208	comp40060_c0_seq2.3137.5416.plus.R8_1	CBM16	*Bacteroides_stercorirosoris*
209	comp11177_c0_seq1.38.895.plus.R1_1	CBM50	*Bacteroides_stercoris*
210	comp289385_c0_seq1.1.762.minus.R1_1	CBM67	*Bacteroides_thetaiotaomicron*
211	comp48717_c0_seq8.25.2370.minus.R8_1	CBM32	*Bacteroides_thetaiotaomicron*
212	comp39828_c0_seq1.1878.2762.plus.R3_1	CBM32	*Bacteroides_thetaiotaomicron*_CAG

213	comp114364_c0_seq1.1.745.minus.R1_1	CBM48	*Bacteroides_uniformis*
214	comp94352_c0_seq1.413.1603.plus.R1_1	CBM48	*Bacteroides_uniformis*
215	comp94506_c0_seq1.50.1273.plus.R8_1	CBM69	*Bacteroides_uniformis*
216	comp62789_c0_seq8.2538.4472.minus.R1_1	CBM62	*Bacteroides_vulgatus*
217	comp62789_c0_seq8.2538.4472.minus.R1_1	CBM62	*Bacteroides_vulgatus*
218	comp39944_c0_seq1.982.3564.minus.R3_1	CBM57	*Bacteroides_vulgatus*
219	comp103713_c0_seq1.1.855.minus.R1_1	CBM69	*Bacteroides_xylanisolvens*
220	comp59402_c0_seq1.1451.2827.plus.R3_1	CBM66	*Bacteroides_xylanisolvens*
221	comp103955_c0_seq1.1.771.minus.R8_1	CBM32	Candidatus_Bacteroides_timonensis
222	comp31046_c0_seq1.68.844.plus.R1_1	CBM50	*Bacteroides* sp.
223	comp72606_c0_seq1.1552.3576.plus.R1_1	CBM62	*Bacteroides* sp.
224	comp72606_c0_seq1.1552.3576.plus.R1_1	CBM62	*Bacteroides* sp.
225	comp9091_c0_seq1.35.1627.plus.R3_1	CBM35	*Bacteroides* sp.
226	comp106422_c0_seq1.1.1050.minus.R8_1	CBM67	uncultured_*Bacteroides*_sp.
227	comp11519_c0_seq1.1.1383.minus.R9_1	CBM20	uncultured_*Bacteroides*_sp.
228	comp171614_c0_seq1.1.851.minus.R7_1	CBM67	uncultured_*Bacteroides*_sp.
229	comp35922_c0_seq1.1.1186.minus.R9_1	CBM13	uncultured_*Bacteroides*_sp.
230	comp38988_c0_seq1.1.1208.minus.R7_1	CBM32	uncultured_*Bacteroides*_sp.
231	comp48272_c0_seq1.6.1826.plus.R9_1	CBM32	uncultured_*Bacteroides*_sp.
232	comp48272_c0_seq1.6.1826.plus.R9_1	CBM32	uncultured_*Bacteroides*_sp.
233	comp78903_c0_seq1.42.800.plus.R9_1	CBM32	uncultured_*Bacteroides*_sp.
234	comp11741_c0_seq1.199.2850.plus.R8_1	CBM16	uncultured_*Bacteroides*_sp.
235	comp29876_c0_seq1.40.2373.plus.R9_1	CBM32	uncultured_*Bacteroides*_sp.
236	comp29876_c0_seq2.3.2354.plus.R9_1	CBM56	uncultured_*Bacteroides*_sp.
237	comp12224_c0_seq1.33.1838.plus.R1_1	CBM35	*Saccharicrinis_fermentans*
238	comp27842_c0_seq2.69.1262.plus.R8_1	CBM66	*Bacteroidales*_bacterium
239	comp33445_c0_seq1.65.1351.minus.R1_1	CBM66	*Bacteroidales*_bacterium
240	comp13546_c0_seq1.38.763.plus.R1_1	CBM34	*Barnesiella_intestinihominis*
241	comp21234_c0_seq1.23.1882.minus.R3_1	CBM40	*Barnesiella_intestinihominis*
242	comp36254_c0_seq4.895.2547.plus.R3_1	CBM44	*Barnesiella_intestinihominis*
243	comp101379_c0_seq1.1.861.minus.R8_1	CBM32	*Barnesiella_viscericola*
244	comp101379_c0_seq1.1.861.minus.R8_1	CBM32	*Barnesiella_viscericola*
245	comp128266_c0_seq1.52.877.minus.R1_1	CBM32	*Barnesiella_viscericola*
246	comp14571_c0_seq1.95.1039.plus.R1_1	CBM50	*Barnesiella_viscericola*
247	comp15134_c0_seq1.52.1494.plus.R1_1	CBM32	*Barnesiella_viscericola*
248	comp15386_c0_seq1.82.1485.minus.R1_1	CBM69	*Barnesiella_viscericola*
249	comp50107_c1_seq3.49.1422.plus.R1_1	CBM20	*Barnesiella_viscericola*
250	comp54957_c0_seq1.14.1518.minus.R1_1	CBM56	*Barnesiella_viscericola*
251	comp65871_c0_seq1.30.1523.plus.R8_1	CBM13	*Barnesiella_viscericola*
252	comp83575_c0_seq1.1.2092.minus.R1_1	CBM40	*Barnesiella_viscericola*
253	comp91371_c0_seq1.568.2043.minus.R1_1	CBM32	*Barnesiella_viscericola*
254	comp16582_c0_seq1.1.2929.minus.R1_1	CBM32	*Barnesiella_viscericola*
255	comp16582_c0_seq1.1.2929.minus.R1_1	CBM32	*Barnesiella_viscericola*
256	comp29409_c0_seq2.1.2324.minus.R9_1	CBM16	*Barnesiella_viscericola*

257	comp66375_c0_seq1.82.2625.plus.R1_1	CBM32	*Barnesiella_viscericola*
258	comp77634_c0_seq1.123.1019.plus.R1_1	CBM50	*Coprobacter_fastidiosus*
259	comp130415_c0_seq1.98.1429.minus.R1_1	CBM66	*Dysgonomonas_gadei*
260	comp60556_c0_seq3.79.1206.plus.R1_1	CBM48	*Dysgonomonas_mossii*
261	comp46845_c0_seq1.548.1613.minus.R7_1	CBM56	*Dysgonomonas*_sp._HGC4
262	comp46845_c0_seq1.548.1613.minus.R7_1	CBM32	*Dysgonomonas*_sp._HGC4
263	comp46845_c0_seq1.548.1613.minus.R7_1	CBM32	*Dysgonomonas*_sp._HGC4
264	comp20177_c0_seq1.5212.6486.plus.R3_1	CBM32	*Fermentimonas_caenicola*
265	comp11608_c0_seq1.1.1286.minus.R9_1	CBM20	*Parabacteroides*_bacterium
266	comp33304_c0_seq1.1.816.minus.R3_1	CBM58	*Parabacteroides*_bacterium
267	comp33828_c0_seq1.1.2705.minus.R9_1	CBM51	*Parabacteroides*_bacterium
268	comp33828_c0_seq1.1.2705.minus.R9_1	CBM51	*Parabacteroides*_bacterium
269	comp35569_c0_seq1.93.2579.plus.R3_1	CBM32	*Parabacteroides*_bacterium
270	comp39608_c0_seq1.1.2569.minus.R9_1	CBM32	*Parabacteroides*_bacterium
271	comp54096_c0_seq2.2390.4696.plus.R7_1	CBM32	*Parabacteroides*_bacterium
272	comp84603_c0_seq1.33.2219.minus.R1_1	CBM32	*Parabacteroides*_bacterium
273	comp118501_c0_seq1.23.1279.plus.R1_1	CBM66	*Parabacteroides_distasonis*
274	comp19061_c0_seq1.1.774.minus.R9_1	CBM50	*Parabacteroides_distasonis*
275	comp19061_c0_seq1.1.774.minus.R9_1	CBM50	*Parabacteroides_distasonis*
276	comp19061_c0_seq1.1.774.minus.R9_1	CBM50	*Parabacteroides_distasonis*
277	comp34829_c0_seq1.88.1281.plus.R3_1	CBM48	*Parabacteroides_distasonis*
278	comp49718_c0_seq1.1664.3736.minus.R7_1	CBM32	*Parabacteroides_distasonis*
279	comp60809_c0_seq1.79.1305.plus.R1_1	CBM50	*Parabacteroides_distasonis*
280	comp60809_c0_seq1.79.1305.plus.R1_1	CBM50	*Parabacteroides_distasonis*
281	comp61981_c0_seq1.820.2358.minus.R1_1	CBM50	*Parabacteroides_distasonis*
282	comp61981_c0_seq1.820.2358.minus.R1_1	CBM50	*Parabacteroides_distasonis*
283	comp61981_c0_seq2.1119.2648.minus.R1_1	CBM50	*Parabacteroides_distasonis*
284	comp61981_c0_seq2.1119.2648.minus.R1_1	CBM50	*Parabacteroides_distasonis*
285	comp78077_c0_seq1.38.1013.minus.R7_1	CBM66	*Parabacteroides_distasonis*
286	comp60066_c0_seq1.14.3631.plus.R1_1	CBM32	*Parabacteroides_distasonis*
287	comp60066_c0_seq1.14.3631.plus.R1_1	CBM32	*Parabacteroides_distasonis*
288	comp66053_c0_seq1.11.2698.plus.R8_1	CBM20	*Parabacteroides_distasonis*
289	comp66053_c0_seq1.11.2698.plus.R8_1	CBM20	*Parabacteroides_distasonis*
290	comp60882_c0_seq1.1.1000.minus.R9_1	CBM50	*Parabacteroides_johnsonii*
291	comp60882_c0_seq1.1.1000.minus.R9_1	CBM50	*Parabacteroides_johnsonii*
292	comp60882_c0_seq1.1.1000.minus.R9_1	CBM50	*Parabacteroides_johnsonii*
293	comp98941_c0_seq1.15.1621.minus.R1_1	CBM32	*Parabacteroides_johnsonii*
294	comp57639_c0_seq2.20.2344.minus.R1_1	CBM32	*Parabacteroides_merdae*
295	comp106893_c0_seq1.41.1823.minus.R1_1	CBM22	*Parabacteroides*_sp._CAG
296	comp15130_c0_seq1.17.1459.plus.R7_1	CBM32	*Parabacteroides*_sp._CAG
297	comp15505_c0_seq1.1.874.minus.R1_1	CBM48	*Parabacteroides*_sp._CAG
298	comp47591_c0_seq1.70.1293.plus.R1_1	CBM50	*Parabacteroides*_sp._CAG
299	comp47591_c0_seq1.70.1293.plus.R1_1	CBM50	*Parabacteroides*_sp._CAG
300	comp61050_c0_seq2.7.1857.plus.R1_1	CBM50	*Parabacteroides*_sp._CAG

301	comp61050_c0_seq2.7.1857.plus.R1_1	CBM50	*Parabacteroides*_sp._CAG
302	comp61050_c0_seq2.7.1857.plus.R1_1	CBM50	*Parabacteroides*_sp._CAG
303	comp62881_c0_seq1.37.1137.plus.R1_1	CBM56	*Parabacteroides*_sp._CAG
304	comp71005_c0_seq1.592.1716.minus.R1_1	CBM50	*Parabacteroides*_sp._CAG
305	comp80284_c0_seq1.2390.3493.plus.R1_1	CBM9	*Parabacteroides*_sp._CAG
306	comp84716_c0_seq1.10.1521.plus.R1_1	CBM32	*Parabacteroides*_sp._CAG
307	comp9918_c0_seq1.69.1295.plus.R9_1	CBM50	*Parabacteroides*_sp._CAG
308	comp9918_c0_seq1.69.1295.plus.R9_1	CBM50	*Parabacteroides*_sp._CAG
309	comp50546_c0_seq2.1595.5596.plus.R1_1	CBM51	*Parabacteroides*_sp._CAG
310	comp50546_c0_seq2.1595.5596.plus.R1_1	CBM51	*Parabacteroides*_sp._CAG
311	comp77664_c0_seq1.10.2685.minus.R1_1	CBM20	*Parabacteroides*_sp._CAG
312	comp77664_c0_seq1.10.2685.minus.R1_1	CBM20	*Parabacteroides*_sp._CAG
313	comp27961_c0_seq1.1.1278.minus.R7_1	CBM48	*Parabacteroides*_sp._YL27
314	comp15041_c0_seq1.1.746.minus.R7_1	CBM67	*Proteiniphilum_acetatigenes*
315	comp28676_c0_seq1.1.978.minus.R7_1	CBM48	*Tannerella*_sp._6_1_58FAA_CT1
316	comp75041_c0_seq1.81.842.plus.R1_1	CBM3	*Tannerella*_sp._CAG
317	comp44527_c0_seq1.1.916.minus.R1_1	CBM32	*Paraprevotella_clara*
318	comp54529_c0_seq1.1.1338.minus.R7_1	CBM16	*Paraprevotella_xylaniphila*
319	comp72392_c0_seq1.174.1434.minus.R3_1	CBM32	*Prevotella_brevis*
320	comp94143_c0_seq1.2071.3642.plus.R1_1	CBM37	*Prevotella_paludivivens*
321	comp47138_c0_seq1.157.2859.minus.R3_1	CBM20	*Prevotella*_sp._109
322	comp47138_c0_seq1.157.2859.minus.R3_1	CBM20	*Prevotella*_sp._109
323	comp18666_c0_seq1.53.1963.minus.R3_1	CBM4	*Prevotella*_sp._CAG
324	comp32491_c0_seq2.1001.3070.plus.R3_1	CBM48	*Prevotella*_sp._CAG
325	comp40471_c0_seq1.31.975.minus.R3_1	CBM50	*Prevotella*_sp._CAG
326	comp50876_c0_seq1.766.2120.minus.R3_1	CBM50	*Prevotella*_sp._CAG
327	comp52439_c0_seq1.10.813.plus.R3_1	CBM50	*Prevotella*_sp._CAG
328	comp75901_c0_seq1.30.914.plus.R3_1	CBM50	*Prevotella*_sp._CAG
329	comp75901_c0_seq1.30.914.plus.R3_1	CBM50	*Prevotella*_sp._CAG
330	comp26217_c0_seq1.114.2468.plus.R3_1	CBM22	*Prevotella*_sp._CAG
331	comp61037_c0_seq1.89.2242.plus.R8_1	CBM37	*Prevotella*_sp._CAG
332	comp61037_c0_seq1.89.2242.plus.R8_1	CBM37	*Prevotella*_sp._CAG
333	comp9575_c0_seq1.2676.6050.plus.R3_1	CBM16	*Prevotella*_sp._CAG
334	comp27974_c0_seq1.1.1383.minus.R3_1	CBM67	*Prevotella*_sp._KHD1
335	comp74954_c0_seq1.1480.4062.plus.R1_1	CBM67	*Prevotella*_sp._KHD1
336	comp11309_c0_seq2.8.2114.minus.R3_1	CBM32	*Prolixibacter_bellariivorans*
337	comp61837_c0_seq4.7.930.plus.R1_1	CBM32	*Prolixibacter_bellariivorans*
338	comp17941_c0_seq1.8.1366.plus.R3_1	CBM50	*Alistipes_finegoldii*
339	comp85137_c0_seq1.1.1051.minus.R7_1	CBM20	*Alistipes_finegoldii*
340	comp34380_c0_seq1.60.2348.plus.R3_1	CBM32	*Alistipes_finegoldii*
341	comp44079_c0_seq1.72.2280.minus.R3_1	CBM32	*Alistipes_finegoldii*
342	comp36087_c0_seq5.5563.7764.plus.R3_1	CBM32	*Alistipes_finegoldii*_CAG
343	comp54214_c0_seq1.2843.4708.plus.R1_1	CBM50	*Alistipes_finegoldii*_CAG
344	comp54214_c0_seq1.2843.4708.plus.R1_1	CBM50	*Alistipes_finegoldii*_CAG

345	comp54214_c0_seq1.2843.4708.plus.R1_1	CBM50	*Alistipes_finegoldii*_CAG
346	comp62094_c0_seq1.1.1049.minus.R9_1	CBM40	*Alistipes_inops*
347	comp118321_c0_seq1.113.982.plus.R1_1	CBM50	*Alistipes_putredinis*
348	comp118321_c0_seq1.113.982.plus.R1_1	CBM50	*Alistipes_putredinis*
349	comp103235_c0_seq1.116.1417.plus.R1_1	CBM50	*Alistipes_putredinis*_CAG
350	comp81303_c0_seq1.14.1201.plus.R3_1	CBM50	*Alistipes_shahii*
351	comp88532_c0_seq1.52.2112.plus.R1_1	CBM32	*Alistipes_shahii*
352	comp35963_c0_seq3.44.2638.plus.R3_1	CBM50	*Alistipes_shahii*
353	comp35963_c0_seq3.44.2638.plus.R3_1	CBM50	*Alistipes_shahii*
354	comp35963_c0_seq3.44.2638.plus.R3_1	CBM50	*Alistipes_shahii*
355	comp188667_c0_seq1.1.702.minus.R1_1	CBM16	*Alistipes*_sp._CAG
356	comp27438_c0_seq1.76.1020.plus.R3_1	CBM32	*Alistipes*_sp._CAG
357	comp27438_c0_seq2.76.885.plus.R3_1	CBM32	*Alistipes*_sp._CAG
358	comp29454_c0_seq1.693.2045.plus.R1_1	CBM66	*Alistipes*_sp._CAG
359	comp32981_c0_seq1.33.2039.plus.R3_1	CBM48	*Alistipes*_sp._CAG
360	comp52139_c0_seq1.7.1304.minus.R7_1	CBM66	*Alistipes*_sp._CAG
361	comp54730_c0_seq4.10.2011.minus.R7_1	CBM32	*Alistipes*_sp._CAG
362	comp54731_c0_seq1.17.1294.plus.R7_1	CBM32	*Alistipes*_sp._CAG
363	comp55346_c0_seq2.1.1247.minus.R7_1	CBM48	*Alistipes*_sp._CAG
364	comp55346_c0_seq6.1.1637.minus.R7_1	CBM48	*Alistipes*_sp._CAG
365	comp55346_c0_seq7.1.1250.minus.R7_1	CBM48	*Alistipes*_sp._CAG
366	comp57362_c0_seq1.189.1196.plus.R3_1	CBM37	*Alistipes*_sp._CAG
367	comp70277_c0_seq1.68.1581.minus.R1_1	CBM37	*Alistipes*_sp._CAG
368	comp104517_c0_seq1.1.3074.minus.R1_1	CBM32	*Alistipes*_sp._CAG
369	comp28902_c0_seq1.21.2840.minus.R1_1	CBM66	*Alistipes*_sp._CAG
370	comp32740_c0_seq1.1.2533.minus.R3_1	CBM20	*Alistipes*_sp._CAG
371	comp32740_c0_seq1.1.2533.minus.R3_1	CBM20	*Alistipes*_sp._CAG
372	comp48534_c0_seq1.17.2341.plus.R1_1	CBM37	*Alistipes*_sp._CAG
373	comp45854_c0_seq1.34.1660.minus.R3_1	CBM32	*Alistipes*_sp._HGB5
374	comp45854_c0_seq1.34.1660.minus.R3_1	CBM32	Alistipes_sp._HGB5
375	comp48398_c0_seq2.3870.5618.plus.R8_1	CBM32	*Alistipes*_sp._Marseille-P2431
376	comp82368_c0_seq1.1988.3658.plus.R1_1	CBM32	*Alistipes*_sp._Marseille-P2431
377	comp35456_c0_seq3.1547.4099.plus.R3_1	CBM32	*Rikenella_microfusus*
378	comp142671_c0_seq1.1.795.minus.R1_1	CBM32	*Runella_limosa*
379	comp61055_c1_seq7.9960.13031.plus.R1_1	CBM66	*Aquimarina_agarivorans*
380	comp85542_c0_seq1.100.1011.minus.R1_1	CBM50	*Bacillus_aquimaris*
381	comp85542_c0_seq1.100.1011.minus.R1_1	CBM50	*Bacillus_aquimaris*
382	comp85289_c0_seq1.1.703.minus.R8_1	CBM12	*Bacillus_licheniformis*
383	comp103319_c0_seq1.1.1412.minus.R1_1	CBM32	*Bacillus*_sp._FJAT-28004
384	comp54431_c0_seq1.1.2539.minus.R7_1	CBM50	*Paenibacillus*_sp._FSL_R5-0912
385	comp54431_c0_seq7.1.2748.minus.R7_1	CBM50	*Paenibacillus*_sp._FSL_R5-0912
386	comp46542_c0_seq2.3282.5681.plus.R8_1	CBM40	*Paenibacillus*_sp._Soil766
387	comp46542_c0_seq1.588.3602.plus.R8_1	CBM38	*Paenibacillus*_sp._TI45-13ar
388	comp46542_c0_seq1.588.3602.plus.R8_1	CBM38	*Paenibacillus*_sp._TI45-13ar

389	comp46542_c0_seq1.588.3602.plus.R8_1	CBM38	*Paenibacillus*_sp._TI45-13ar
390	comp46542_c0_seq1.588.3602.plus.R8_1	CBM38	*Paenibacillus*_sp._TI45-13ar
391	comp43501_c0_seq1.12.764.plus.R8_1	CBM56	*Paenibacillus*_sp._UNC451MF
392	comp31892_c0_seq2.125.3298.minus.R1_1	CBM32	*Paenibacillus_terrigena*
393	comp31892_c0_seq2.125.3298.minus.R1_1	CBM32	*Paenibacillus_terrigena*
394	comp74342_c0_seq1.1076.2036.minus.R1_1	CBM50	*Planococcus*_sp._PAMC_21323
395	comp60778_c0_seq1.3.785.plus.R3_1	CBM9	*Streptococcus_marimammalium*
396	comp49890_c0_seq11.1229.2890.minus.R8_1	CBM34	*Streptococcus_suis*
397	comp49890_c0_seq4.78.1594.minus.R8_1	CBM34	*Streptococcus_suis*
398	comp26391_c0_seq1.2593.4692.minus.R8_1	CBM48	*Butyricicoccus_pullicaecorum*
399	comp47725_c0_seq1.1.742.minus.R8_1	CBM48	uncultured_*Butyricicoccus*_sp.
400	comp59359_c0_seq1.2118.5240.plus.R1_1	CBM22	*Caloranaerobacter_azorensis*
401	comp49843_c0_seq1.22.3681.minus.R8_1	CBM32	*Clostridium_celatum*
402	comp49904_c0_seq1.83.5758.plus.R7_1	CBM32	*Clostridium_celatum*
403	comp49904_c0_seq1.83.5758.plus.R7_1	CBM32	*Clostridium_celatum*
404	comp39113_c0_seq3.8.1768.plus.R9_1	CBM32	*Clostridium_chauvoei*
405	comp11897_c0_seq2.10.3729.plus.R8_1	CBM32	*Clostridium_chauvoei*
406	comp11897_c0_seq2.10.3729.plus.R8_1	CBM46	*Clostridium_chauvoei*
407	comp26676_c0_seq1.3.2867.minus.R8_1	CBM32	*Clostridium_chauvoei*
408	comp31925_c0_seq1.59.3186.minus.R3_1	CBM32	*Clostridium_perfringens*
409	comp28541_c0_seq1.21.956.minus.R7_1	CBM12	*Clostridium*_sp._ASF356
410	comp28541_c0_seq1.21.956.minus.R7_1	CBM12	*Clostridium*_sp._ASF356
411	comp73556_c0_seq1.3.821.plus.R1_1	CBM50	*Clostridium*_sp._ASF356
412	comp90533_c0_seq1.1.1050.minus.R1_1	CBM54	*Clostridium*_sp._ASF356
413	comp60317_c0_seq1.61.3972.plus.R1_1	CBM54	*Clostridium*_sp._ASF356
414	comp108198_c0_seq1.1.768.minus.R1_1	CBM40	*Clostridium*_sp._C105KSO13
415	comp49644_c0_seq1.58.4206.minus.R8_1	CBM40	*Clostridium*_sp._C105KSO13
416	comp13781_c0_seq1.45.833.plus.R8_1	CBM50	*Clostridium*_sp._CAG
417	comp38785_c0_seq1.1.2008.minus.R8_1	CBM54	*Clostridium*_sp._CAG
418	comp38785_c0_seq1.1.2008.minus.R8_1	CBM54	*Clostridium*_sp._CAG
419	comp73688_c0_seq1.23.1240.minus.R1_1	CBM35	*Clostridium*_sp._CAG
420	comp97535_c0_seq1.42.1694.plus.R7_1	CBM32	*Clostridium*_sp._CAG
421	comp44604_c0_seq1.60.1501.minus.R8_1	CBM32	*Clostridium*_sp._D5
422	comp52270_c0_seq1.30.1820.plus.R1_1	CBM4	*Clostridium*_sp._Marseille-P299
423	comp113476_c0_seq1.1.735.minus.R1_1	CBM32	uncultured_*Clostridium*_sp.
424	comp45419_c0_seq1.1.852.minus.R1_1	CBM67	uncultured_*Clostridium*_sp.
425	comp54141_c0_seq1.87.1655.plus.R1_1	CBM22	uncultured_*Clostridium*_sp.
426	comp86761_c0_seq1.1.823.minus.R1_1	CBM32	uncultured_*Clostridium*_sp.
427	comp22154_c0_seq1.65.1588.plus.R3_1	CBM50	*Eubacterium_desmolans*
428	comp142540_c0_seq1.144.1274.plus.R7_1	CBM32	*Eubacterium*_sp._CAG
429	comp72085_c0_seq1.27.1556.plus.R8_1	CBM50	*Eubacterium*_sp._CAG
430	comp40910_c0_seq1.37.1596.plus.R7_1	CBM32	*Eubacterium*_sp._SB2
431	comp43847_c0_seq3.128.2087.minus.R8_1	CBM48	*Eubacteriaceae*_bacterium_CHKCI004
432	comp47949_c0_seq1.1.1131.minus.R1_1	CBM48	*Eubacteriaceae*_bacterium_CHKCI004

433	comp74051_c0_seq1.1.1501.minus.R8_1	CBM50	*Eubacteriaceae*_bacterium_CHKCI004
434	comp60921_c0_seq1.68.2485.minus.R1_1	CBM37	*Eubacteriaceae*_bacterium_CHKCI004
435	comp18379_c0_seq1.671.4975.minus.R8_1	CBM32	*Anaerobium_acetethylicum*
436	comp18379_c0_seq1.671.4975.minus.R8_1	CBM32	*Anaerobium_acetethylicum*
437	comp18379_c0_seq1.671.4975.minus.R8_1	CBM32	*Anaerobium_acetethylicum*
438	comp48117_c0_seq1.1116.3776.plus.R8_1	CBM32	*Anaerobium_acetethylicum*
439	comp52623_c0_seq1.36.2672.minus.R7_1	CBM32	*Anaerobium_acetethylicum*
440	comp141426_c0_seq1.9.904.minus.R1_1	CBM37	*Blautia_hydrogenotrophica*
441	comp141426_c0_seq1.9.904.minus.R1_1	CBM37	*Blautia_hydrogenotrophica*
442	comp100034_c0_seq1.1.1191.minus.R1_1	CBM48	*Blautia_schinkii*
443	comp17253_c0_seq1.217.1185.plus.R1_1	CBM37	*Blautia_schinkii*
444	comp45561_c0_seq1.1.1748.minus.R8_1	CBM32	*Blautia_schinkii*
445	comp99918_c0_seq1.30.2202.minus.R1_1	CBM48	*Blautia_schinkii*
446	comp99918_c0_seq1.30.2202.minus.R1_1	CBM48	*Blautia_schinkii*
447	comp30948_c0_seq2.1.710.minus.R9_1	CBM37	*Blautia*_sp._CAG
448	comp39347_c0_seq1.1.1010.minus.R9_1	CBM37	*Blautia*_sp._CAG
449	comp39347_c0_seq1.1.1010.minus.R9_1	CBM37	*Blautia*_sp._CAG
450	comp52921_c0_seq1.1.767.minus.R1_1	CBM37	uncultured_*Blautia*_sp.
451	comp54126_c0_seq2.1545.3380.plus.R1_1	CBM4	*Butyrivibrio_fibrisolvens*
452	comp54126_c0_seq2.1545.3380.plus.R1_1	CBM4	*Butyrivibrio_fibrisolvens*
453	comp54126_c0_seq2.1545.3380.plus.R1_1	CBM4	*Butyrivibrio_fibrisolvens*
454	comp55885_c0_seq4.30.2777.plus.R7_1	CBM41	*Butyrivibrio*_sp._CAG
455	comp49868_c1_seq1.110.5989.plus.R8_1	CBM32	*Coprococcus_comes*
456	comp49868_c1_seq1.110.5989.plus.R8_1	CBM32	*Coprococcus_comes*
457	comp46295_c0_seq2.52.2310.plus.R8_1	CBM4	*Herbinix_hemicellulosilytica*
458	comp46295_c0_seq2.52.2310.plus.R8_1	CBM4	*Herbinix_hemicellulosilytica*
459	comp49843_c0_seq3.51.2172.minus.R8_1	CBM32	*Lachnospiraceae*_bacterium_2_1_46FAA
460	comp49843_c0_seq4.22.2185.minus.R8_1	CBM32	*Lachnospiraceae*_bacterium_2_1_46FAA
461	comp62882_c0_seq2.165.7331.plus.R1_1	CBM32	*Lachnospiraceae*_bacterium_6_1_37FAA
462	comp15922_c0_seq1.1.1377.minus.R1_1	CBM50	*Lachnospiraceae*_bacterium_7_1_58FAA
463	comp26808_c0_seq1.38.4099.plus.R8_1	CBM32	*Lachnospiraceae*_bacterium_8_1_57FAA
464	comp26808_c0_seq1.38.4099.plus.R8_1	CBM40	*Lachnospiraceae*_bacterium_8_1_57FAA
465	comp26808_c0_seq1.38.4099.plus.R8_1	CBM40	*Lachnospiraceae*_bacterium_8_1_57FAA
466	comp10103_c0_seq1.1.892.minus.R8_1	CBM37	*Lachnospiraceae*_bacterium_A2
467	comp84507_c0_seq1.11.1953.minus.R7_1	CBM32	*Lachnospiraceae*_bacterium_AD3010
468	comp46612_c0_seq1.6.1383.minus.R1_1	CBM6	*Lachnospiraceae*_bacterium_MC2017
469	comp26841_c0_seq1.11.2941.plus.R1_1	CBM32	*Lachnospiraceae*_bacterium
470	comp36472_c0_seq3.49.2790.minus.R3_1	CBM32	*Lachnospiraceae*_bacterium
471	comp49843_c0_seq6.22.4950.minus.R8_1	CBM32	*Lachnospiraceae*_bacterium
472	comp49843_c0_seq6.22.4950.minus.R8_1	CBM32	*Lachnospiraceae*_bacterium
473	comp49843_c0_seq6.22.4950.minus.R8_1	CBM32	*Lachnospiraceae*_bacterium
474	comp58538_c0_seq1.39.2468.plus.R1_1	CBM32	Robinsoniella_peoriensis
475	comp38210_c0_seq2.25.2451.minus.R9_1	CBM32	*Robinsoniella*_sp._KNHs210
476	comp38210_c0_seq2.25.2451.minus.R9_1	CBM32	*Robinsoniella*_sp._KNHs210

477	comp38210_c0_seq2.25.2451.minus.R9_1	CBM32	*Robinsoniella*_sp._KNHs210
478	comp38210_c0_seq2.25.2451.minus.R9_1	CBM32	*Robinsoniella*_sp._KNHs210
479	comp8265_c0_seq1.59.2767.plus.R8_1	CBM32	*Robinsoniella*_sp._RHS
480	comp31351_c0_seq1.1.1106.minus.R3_1	CBM32	*Roseburia*_sp._CAG
481	comp32691_c0_seq1.1.1168.minus.R3_1	CBM40	*Tyzzerella_nexilis*
482	comp18379_c0_seq2.59.3942.minus.R8_1	CBM32	*Tyzzerella_nexilis*
483	comp18379_c0_seq2.59.3942.minus.R8_1	CBM32	*Tyzzerella_nexilis*
484	comp24341_c0_seq1.171.5702.plus.R7_1	CBM22	*Tyzzerella_nexilis*
485	comp24341_c0_seq1.171.5702.plus.R7_1	CBM6	*Tyzzerella_nexilis*
486	comp24341_c0_seq1.171.5702.plus.R7_1	CBM22	*Tyzzerella_nexilis*
487	comp35722_c0_seq1.65.2623.plus.R3_1	CBM32	*Tyzzerella_nexilis*
488	comp48071_c0_seq1.1.5130.minus.R8_1	CBM32	*Tyzzerella_nexilis*
489	comp48071_c0_seq1.1.5130.minus.R8_1	CBM32	*Tyzzerella_nexilis*
490	comp48071_c0_seq1.1.5130.minus.R8_1	CBM32	*Tyzzerella_nexilis*
491	comp48071_c0_seq1.1.5130.minus.R8_1	CBM32	*Tyzzerella_nexilis*
492	comp48071_c0_seq1.1.5130.minus.R8_1	CBM32	*Tyzzerella_nexilis*
493	comp60378_c0_seq1.35.772.minus.R8_1	CBM16	*Flavonifractor_plautii*
494	comp49046_c0_seq4.321.1262.minus.R8_1	CBM12	uncultured_*Flavonifractor*_sp.
495	comp59307_c0_seq1.45.1031.plus.R7_1	CBM12	uncultured_*Flavonifractor*_sp.
496	comp13346_c0_seq1.2.1651.plus.R8_1	CBM50	*Clostridiales_bacterium*_CHKCI001
497	comp33960_c0_seq1.102.2264.plus.R8_1	CBM32	*Clostridiales_bacterium*_CHKCI001
498	comp79724_c0_seq1.127.2070.minus.R1_1	CBM48	*Clostridiales_bacterium*_CHKCI006
499	comp56404_c0_seq1.26.2116.plus.R9_1	CBM54	*Desulfitibacter_alkalitolerans*
500	comp52198_c0_seq1.1.1243.minus.R7_1	CBM48	*Anaerotruncus*_sp._MT15
501	comp13185_c0_seq1.7.1537.minus.R8_1	CBM35	Candidatus_Soleaferrea_massiliensis
502	comp12407_c0_seq1.25.1989.minus.R1_1	CBM37	*Faecalibacterium_prausnitzii*
503	comp145572_c0_seq1.1.946.minus.R1_1	CBM32	*Faecalibacterium_prausnitzii*
504	comp35122_c0_seq2.1.1390.minus.R3_1	CBM48	*Faecalibacterium_prausnitzii*
505	comp35122_c0_seq4.1.1361.minus.R3_1	CBM48	*Faecalibacterium_prausnitzii*
506	comp61799_c0_seq4.471.1368.minus.R1_1	CBM50	*Faecalibacterium_prausnitzii*
507	comp62975_c0_seq1.3898.6237.minus.R1_1	CBM48	*Faecalibacterium_prausnitzii*
508	comp57970_c0_seq2.30.917.minus.R1_1	CBM13	*Faecalibacterium*_sp._CAG
509	comp58881_c0_seq1.1.1190.minus.R8_1	CBM48	*Faecalibacterium*_sp._CAG
510	comp82064_c0_seq1.12.851.minus.R3_1	CBM13	*Faecalibacterium*_sp._CAG
511	comp26380_c0_seq1.64.1983.plus.R8_1	CBM4	*Ruminococcaceae*_bacterium_AB4001
512	comp47518_c0_seq1.305.2218.minus.R8_1	CBM48	*Ruminococcaceae*_bacterium_AM2
513	comp34436_c0_seq3.1.1293.minus.R3_1	CBM35	*Ruminococcaceae*_bacterium_mt9
514	comp57456_c0_seq1.1.1623.minus.R1_1	CBM6	*Ruminococcaceae*_bacterium_mt9
515	comp57456_c0_seq1.1.1623.minus.R1_1	CBM61	*Ruminococcaceae*_bacterium_mt9
516	comp57456_c0_seq1.1.1623.minus.R1_1	CBM6	*Ruminococcaceae*_bacterium_mt9
517	comp57456_c0_seq1.1.1623.minus.R1_1	CBM35	*Ruminococcaceae*_bacterium_mt9
518	comp47987_c0_seq2.298.2043.plus.R8_1	CBM20	[*Clostridium*]_*cellulosi*
519	comp49725_c0_seq4.1.3517.minus.R8_1	CBM37	*Ruminococcus_faecis*
520	comp36477_c0_seq3.64.4677.plus.R3_1	CBM35	*Ruminococcus_lactaris*

521	comp61284_c0_seq1.1.3655.minus.R8_1	CBM32	*Ruminococcus_lactaris*
522	comp61284_c0_seq1.1.3655.minus.R8_1	CBM32	*Ruminococcus_lactaris*
523	comp61284_c0_seq1.1.3655.minus.R8_1	CBM32	*Ruminococcus_lactaris*
524	comp52229_c0_seq1.48.2051.plus.R8_1	CBM48	uncultured_*Ruminococcus*_sp.
525	comp49431_c0_seq1.89.2386.plus.R8_1	CBM35	uncultured_*Ruminococcus*_sp.
526	comp54247_c0_seq1.21.4337.plus.R8_1	CBM32	uncultured_*Ruminococcus*_sp.
527	comp54247_c0_seq1.21.4337.plus.R8_1	CBM32	uncultured_*Ruminococcus*_sp.
528	comp54247_c0_seq1.21.4337.plus.R8_1	CBM32	uncultured_*Ruminococcus*_sp.
529	comp85607_c0_seq1.7.3960.minus.R1_1	CBM35	uncultured_*Ruminococcus*_sp.
530	comp85607_c0_seq1.7.3960.minus.R1_1	CBM32	uncultured_*Ruminococcus*_sp.
531	comp85607_c0_seq1.7.3960.minus.R1_1	CBM35	uncultured_*Ruminococcus*_sp.
532	comp57570_c0_seq1.7.936.minus.R1_1	CBM13	*Subdoligranulum_variabile*
533	comp57570_c0_seq1.7.936.minus.R1_1	CBM13	*Subdoligranulum_variabile*
534	comp53953_c0_seq1.187.1311.minus.R1_1	CBM50	*Clostridia*_bacterium_UC5.1-1D10
535	comp60542_c0_seq2.13.5550.plus.R1_1	CBM54	*Clostridia*_bacterium_UC5.1-1D10
536	comp11632_c0_seq1.372.1217.plus.R1_1	CBM71	*Clostridia*_bacterium_UC5.1-1E11
537	comp58526_c0_seq1.1.1413.minus.R1_1	CBM6	*Clostridia*_bacterium_UC5.1-1E11
538	comp62878_c0_seq10.10.2193.plus.R1_1	CBM35	*Clostridia*_bacterium_UC5.1-1E11
539	comp60419_c0_seq1.175.1365.plus.R3_1	CBM50	*Clostridia*_bacterium_UC5.1-2F7
540	comp14305_c0_seq1.22.981.minus.R8_1	CBM37	*Caldicellulosiruptor_kronotskyensis*
541	comp14305_c0_seq1.22.981.minus.R8_1	CBM37	*Caldicellulosiruptor_kronotskyensis*
542	comp46542_c0_seq2.5711.7711.plus.R8_1	CBM38	*Thermoanaerobacter_italicus*
543	comp49868_c1_seq2.266.5041.plus.R8_1	CBM32	[*Clostridium*]_*saccharogumia*
544	comp49868_c1_seq2.266.5041.plus.R8_1	CBM35	[*Clostridium*]_*saccharogumia*
545	comp108331_c0_seq1.1.813.minus.R7_1	CBM32	[*Clostridium*]_*saccharogumia*
546	comp111934_c0_seq1.1.1084.minus.R7_1	CBM32	[*Clostridium*]_*saccharogumia*
547	comp127630_c0_seq1.1.701.minus.R1_1	CBM32	[*Clostridium*]_*saccharogumia*
548	comp127630_c0_seq1.1.701.minus.R1_1	CBM32	[*Clostridium*]_*saccharogumia*
549	comp154400_c0_seq1.1.925.minus.R1_1	CBM6	[*Clostridium*]_*saccharogumia*
550	comp154400_c0_seq1.1.925.minus.R1_1	CBM32	[*Clostridium*]_*saccharogumia*
551	comp38496_c0_seq1.38.1114.plus.R8_1	CBM32	[*Clostridium*]_*saccharogumia*
552	comp55060_c0_seq1.1.2382.minus.R1_1	CBM32	[*Clostridium*]_*saccharogumia*
553	comp55060_c0_seq1.1.2382.minus.R1_1	CBM32	[*Clostridium*]_*saccharogumia*
554	comp55060_c0_seq1.1.2382.minus.R1_1	CBM32	[*Clostridium*]_*saccharogumia*
555	comp36472_c0_seq1.1.2236.minus.R3_1	CBM32	*Erysipelotrichaceae*_bacterium_MTC7
556	comp44042_c0_seq1.1.1103.minus.R1_1	CBM34	*Acidaminococcus*_sp._CAG
557	comp62904_c0_seq1.2620.4632.plus.R1_1	CBM48	*Megamonas_funiformis*
558	comp114883_c0_seq1.1.735.minus.R7_1	CBM35	*Firmicutes*_bacterium_CAG
559	comp166787_c0_seq1.30.951.minus.R1_1	CBM56	*Firmicutes*_bacterium_CAG
560	comp44898_c0_seq2.228.1271.minus.R8_1	CBM50	*Firmicutes*_bacterium_CAG
561	comp60567_c0_seq1.1.1623.minus.R1_1	CBM48	*Firmicutes*_bacterium_CAG
562	comp60567_c0_seq1.1.1623.minus.R1_1	CBM48	*Firmicutes*_bacterium_CAG
563	comp61538_c0_seq1.11.1915.minus.R1_1	CBM50	*Firmicutes*_bacterium_CAG
564	comp78114_c0_seq1.12.1166.minus.R1_1	CBM26	*Firmicutes*_bacterium_CAG

565	comp78114_c0_seq1.12.1166.minus.R1_1	CBM26	*Firmicutes*_bacterium_CAG
566	comp78114_c0_seq1.12.1166.minus.R1_1	CBM26	*Firmicutes*_bacterium_CAG
567	comp83672_c0_seq1.131.1891.plus.R7_1	CBM35	*Firmicutes*_bacterium_CAG
568	comp85985_c0_seq1.13.1185.plus.R8_1	CBM50	*Firmicutes*_bacterium_CAG
569	comp95176_c0_seq1.22.1525.minus.R1_1	CBM61	*Firmicutes*_bacterium_CAG
570	comp42916_c0_seq1.4.2586.plus.R8_1	CBM48	*Firmicutes*_bacterium_CAG
571	comp42916_c0_seq1.4.2586.plus.R8_1	CBM48	*Firmicutes*_bacterium_CAG
572	comp61837_c0_seq3.97.3876.plus.R1_1	CBM40	uncultured_bacterium_F082
573	comp61837_c0_seq7.97.3876.plus.R1_1	CBM40	uncultured_bacterium_F082
574	comp45683_c0_seq1.1.855.minus.R8_1	CBM50	uncultured_bacterium
575	comp49741_c0_seq1.14.3739.minus.R1_1	CBM32	*Rhodopirellula_sallentina*
576	comp94637_c0_seq1.522.1337.minus.R1_1	CBM50	*Achromobacter_xylosoxidans*
577	comp37781_c0_seq1.1.837.minus.R9_1	CBM54	*Burkholderia_contaminans*
578	comp102373_c0_seq1.39.848.minus.R7_1	CBM50	*Pandoraea_norimbergensis*
579	comp52074_c0_seq1.26.3301.minus.R1_1	CBM32	*Massilia*_sp._NR_4-1
580	comp52074_c0_seq1.26.3301.minus.R1_1	CBM51	*Massilia*_sp._NR_4-1
581	comp42082_c0_seq1.1.1204.minus.R8_1	CBM35	*Corallococcus*_sp._CAG
582	comp60595_c0_seq1.1.1266.minus.R9_1	CBM50	*Corallococcus*_sp._CAG
583	comp46029_c0_seq1.2225.4318.plus.R1_1	CBM48	*Deferrisoma_camini*
584	comp84669_c0_seq1.48.1287.minus.R1_1	CBM51	*Rheinheimera*_sp._SA_1
585	comp84669_c0_seq1.48.1287.minus.R1_1	CBM32	*Rheinheimera*_sp._SA_1
586	comp26260_c0_seq1.54.4295.plus.R8_1	CBM32	*Treponema_lecithinolyticum*
587	comp26260_c0_seq1.54.4295.plus.R8_1	CBM32	*Treponema_lecithinolyticum*
588	comp39965_c0_seq1.18.1932.minus.R9_1	CBM48	*Cloacibacillus_porcorum*
589	comp28204_c0_seq1.160.2370.plus.R1_1	CBM48	*Opitutaceae*_bacterium_BACL24_MAG-120322-bin51
590	comp28204_c0_seq1.160.2370.plus.R1_1	CBM48	*Opitutaceae*_bacterium_BACL24_MAG-120322-bin51
591	comp61480_c0_seq2.2986.6651.plus.R1_1	CBM47	*Akkermansia_glycaniphila*
592	comp10336_c0_seq1.28.2457.plus.R1_1	CBM32	*Akkermansia_muciniphila*
593	comp82146_c0_seq1.28.3816.minus.R1_1	CBM32	*Akkermansia_muciniphila*
594	comp85262_c0_seq1.19.2454.plus.R1_1	CBM32	*Akkermansia*_sp._KLE1797
595	comp93327_c0_seq1.153.1397.minus.R1_1	CBM20	*Gregarina_niphandrodes*
596	comp15806_c0_seq1.1.1542.minus.R9_1	CBM32	*Blastocystis_hominis*
597	comp40447_c0_seq14.1.2157.minus.R9_1	CBM32	*Blastocystis_hominis*
598	comp40521_c0_seq7.1.924.minus.R9_1	CBM32	*Blastocystis_hominis*
599	comp45777_c0_seq1.1.1383.minus.R8_1	CBM32	*Blastocystis_hominis*
600	comp49595_c0_seq4.1.1959.minus.R8_1	CBM32	*Blastocystis_hominis*
601	comp52451_c0_seq2.1.1411.minus.R1_1	CBM32	*Blastocystis_hominis*
602	comp53530_c0_seq1.14.2168.minus.R1_1	CBM48	*Blastocystis_hominis*
603	comp54530_c0_seq1.1.1223.minus.R7_1	CBM67	*Blastocystis_hominis*
604	comp57795_c0_seq1.1.1337.minus.R1_1	CBM32	*Blastocystis_hominis*
605	comp36346_c0_seq5.1.2235.minus.R3_1	CBM32	*Blastocystis_hominis*
606	comp36602_c0_seq7.3.4958.plus.R3_1	CBM32	*Blastocystis_hominis*
607	comp40122_c0_seq1.1.4720.minus.R9_1	CBM32	*Blastocystis_hominis*
608	comp40122_c0_seq1.1.4720.minus.R9_1	CBM32	*Blastocystis_hominis*

609	comp40447_c0_seq15.1.2828.minus.R9_1	CBM32	*Blastocystis_hominis*
610	comp40447_c0_seq16.1.4937.minus.R9_1	CBM32	*Blastocystis_hominis*
611	comp40447_c0_seq18.1.2834.minus.R9_1	CBM32	*Blastocystis_hominis*
612	comp40447_c0_seq19.1.2730.minus.R9_1	CBM32	*Blastocystis_hominis*
613	comp40447_c0_seq4.1.4238.minus.R9_1	CBM32	*Blastocystis_hominis*
614	comp40447_c0_seq6.1.4232.minus.R9_1	CBM32	*Blastocystis_hominis*
615	comp40447_c0_seq8.1.2828.minus.R9_1	CBM32	*Blastocystis_hominis*
616	comp40494_c0_seq2.17.4948.minus.R9_1	CBM32	*Blastocystis_hominis*
617	comp40521_c0_seq8.1.3912.minus.R9_1	CBM32	*Blastocystis_hominis*
618	comp40521_c0_seq8.1.3912.minus.R9_1	CBM32	*Blastocystis_hominis*
619	comp40521_c0_seq8.1.3912.minus.R9_1	CBM32	*Blastocystis_hominis*
620	comp40521_c0_seq8.1.3912.minus.R9_1	CBM67	*Blastocystis_hominis*
621	comp46658_c0_seq1.1.4379.minus.R8_1	CBM32	*Blastocystis_hominis*
622	comp46658_c0_seq1.1.4379.minus.R8_1	CBM32	*Blastocystis_hominis*
623	comp49595_c0_seq1.1.3799.minus.R8_1	CBM32	*Blastocystis_hominis*
624	comp49595_c0_seq2.1.3510.minus.R8_1	CBM32	*Blastocystis_hominis*
625	comp49595_c0_seq3.1.4955.minus.R8_1	CBM32	*Blastocystis_hominis*
626	comp49595_c0_seq5.1.4951.minus.R8_1	CBM32	*Blastocystis_hominis*
627	comp54611_c0_seq2.1.2846.minus.R7_1	CBM32	*Blastocystis_hominis*
628	comp55679_c0_seq1.12.3398.minus.R7_1	CBM32	*Blastocystis_hominis*
629	comp55734_c0_seq2.100.4881.plus.R7_1	CBM32	*Blastocystis_hominis*
630	comp55734_c0_seq2.100.4881.plus.R7_1	CBM32	*Blastocystis_hominis*
631	comp55734_c0_seq2.100.4881.plus.R7_1	CBM67	*Blastocystis_hominis*
632	comp55734_c0_seq2.100.4881.plus.R7_1	CBM67	*Blastocystis_hominis*
633	comp55892_c0_seq4.1.4450.minus.R7_1	CBM67	*Blastocystis_hominis*
634	comp55892_c0_seq4.1.4450.minus.R7_1	CBM32	*Blastocystis_hominis*
635	comp55892_c0_seq4.1.4450.minus.R7_1	CBM32	*Blastocystis_hominis*
636	comp59330_c0_seq1.1.2902.minus.R1_1	CBM20	*Blastocystis_hominis*
637	comp61660_c0_seq3.3.4895.plus.R1_1	CBM32	*Blastocystis_hominis*
638	comp61660_c0_seq3.3.4895.plus.R1_1	CBM32	*Blastocystis_hominis*
639	comp61660_c0_seq3.3.4895.plus.R1_1	CBM67	*Blastocystis_hominis*
640	comp61863_c0_seq10.20.4978.plus.R1_1	CBM32	*Blastocystis_hominis*
641	comp61863_c0_seq1.25.4179.plus.R1_1	CBM32	*Blastocystis_hominis*
642	comp61863_c0_seq4.20.4975.plus.R1_1	CBM32	*Blastocystis_hominis*
643	comp61863_c0_seq5.20.4978.plus.R1_1	CBM32	*Blastocystis_hominis*
644	comp61863_c0_seq7.20.4978.plus.R1_1	CBM32	*Blastocystis_hominis*
645	comp61928_c0_seq2.7.4731.minus.R1_1	CBM32	*Blastocystis_hominis*
646	comp61928_c0_seq2.7.4731.minus.R1_1	CBM32	*Blastocystis_hominis*
647	comp62838_c0_seq7.7.4992.plus.R1_1	CBM32	*Blastocystis_hominis*
648	comp62943_c0_seq1.135.6848.plus.R1_1	CBM32	*Blastocystis_hominis*
649	comp62943_c0_seq1.135.6848.plus.R1_1	CBM32	*Blastocystis_hominis*
650	comp42998_c0_seq1.1.795.minus.R1_1	CBM48	*Blastocystis*_sp._subtype_4
651	comp42998_c0_seq2.1.795.minus.R1_1	CBM48	*Blastocystis*_sp._subtype_4
652	comp35346_c0_seq1.1.2443.minus.R3_1	CBM32	*Blastocystis*_sp._subtype_4

653	comp40275_c0_seq2.1.2267.minus.R9_1	CBM32	*Blastocystis*_sp._subtype_4
654	comp49045_c0_seq2.1.3482.minus.R8_1	CBM32	*Blastocystis*_sp._subtype_4
655	comp49045_c0_seq3.127.2529.plus.R8_1	CBM32	*Blastocystis*_sp._subtype_4
656	comp118232_c0_seq1.1.1018.minus.R1_1	CBM55	*Entamoeba_nuttalli*
657	comp14558_c0_seq1.1.714.minus.R1_1	CBM48	unclassified_bacterium
658	comp57556_c0_seq1.1.1554.minus.R8_1	CBM32	unclassified_bacterium
659	comp100065_c0_seq1.116.528.minus.R7_1	CBM56	unclassified
660	comp10028_c0_seq1.34.417.plus.R3_1	CBM50	unclassified
661	comp100693_c0_seq1.1.537.minus.R7_1	CBM15	unclassified
662	comp101444_c0_seq1.1.783.minus.R3_1	CBM46	unclassified
663	comp102303_c0_seq1.8.521.minus.R7_1	CBM50	unclassified
664	comp102467_c0_seq1.1.376.minus.R9_1	CBM37	unclassified
665	comp102648_c0_seq1.1.155.minus.R3_1	CBM50	unclassified
666	comp102741_c0_seq1.1.312.minus.R8_1	CBM20	unclassified
667	comp103050_c0_seq1.122.346.plus.R7_1	CBM50	unclassified
668	comp103073_c0_seq1.814.1455.plus.R1_1	CBM9	unclassified
669	comp10393_c0_seq1.1.687.minus.R1_1	CBM32	unclassified
670	comp104242_c0_seq1.56.394.plus.R1_1	CBM32	unclassified
671	comp105292_c0_seq1.1.697.minus.R3_1	CBM16	unclassified
672	comp105509_c0_seq1.1.469.minus.R8_1	CBM32	unclassified
673	comp10724_c0_seq1.1.514.minus.R1_1	CBM32	unclassified
674	comp107887_c0_seq1.1.507.minus.R3_1	CBM9	unclassified
675	comp108111_c0_seq1.1.424.minus.R9_1	CBM32	unclassified
676	comp10833_c0_seq1.27.458.plus.R8_1	CBM76	unclassified
677	comp108343_c0_seq1.1.499.minus.R3_1	CBM32	unclassified
678	comp108561_c0_seq1.50.535.plus.R7_1	CBM20	unclassified
679	comp108624_c0_seq1.1.396.minus.R7_1	CBM4	unclassified
680	comp108959_c0_seq1.1.614.minus.R9_1	CBM32	unclassified
681	comp109282_c0_seq1.1.515.minus.R9_1	CBM54	unclassified
682	comp109353_c0_seq1.1.561.minus.R1_1	CBM50	unclassified
683	comp109353_c0_seq1.1.561.minus.R1_1	CBM50	unclassified
684	comp109785_c0_seq1.1.349.minus.R8_1	CBM32	unclassified
685	comp110239_c0_seq1.1.527.minus.R9_1	CBM50	unclassified
686	comp11048_c0_seq1.35.484.plus.R1_1	CBM50	unclassified
687	comp113759_c0_seq1.1.361.minus.R9_1	CBM32	unclassified
688	comp11410_c0_seq1.1238.2296.plus.R8_1	CBM9	unclassified
689	comp114260_c0_seq1.48.611.plus.R1_1	CBM9	unclassified
690	comp114422_c0_seq1.1.636.minus.R8_1	CBM48	unclassified
691	comp116077_c0_seq1.1.312.minus.R1_1	CBM70	unclassified
692	comp11805_c0_seq1.1.3122.minus.R1_1	CBM32	unclassified
693	comp118118_c0_seq1.1.521.minus.R8_1	CBM51	unclassified
694	comp118246_c0_seq1.1.644.minus.R1_1	CBM51	unclassified
695	comp118246_c0_seq1.1.644.minus.R1_1	CBM51	unclassified
696	comp118384_c0_seq1.7.564.plus.R1_1	CBM32	unclassified

697	comp118729_c0_seq1.16.342.plus.R7_1	CBM24	unclassified
698	comp118927_c0_seq1.1.375.minus.R9_1	CBM50	unclassified
699	comp118927_c0_seq1.1.375.minus.R9_1	CBM50	unclassified
700	comp119005_c0_seq1.38.310.minus.R1_1	CBM50	unclassified
701	comp119233_c0_seq1.1.308.minus.R8_1	CBM32	unclassified
702	comp119275_c0_seq1.49.498.plus.R8_1	CBM32	unclassified
703	comp119757_c0_seq1.1.463.minus.R7_1	CBM8	unclassified
704	comp120985_c0_seq1.1.656.minus.R8_1	CBM48	unclassified
705	comp121723_c0_seq1.58.651.plus.R7_1	CBM50	unclassified
706	comp121723_c0_seq1.58.651.plus.R7_1	CBM50	unclassified
707	comp12187_c0_seq1.60.395.plus.R1_1	CBM50	unclassified
708	comp121977_c0_seq1.1.386.minus.R8_1	CBM48	unclassified
709	comp122360_c0_seq1.61.498.plus.R7_1	CBM6	unclassified
710	comp122833_c0_seq1.41.589.plus.R1_1	CBM32	unclassified
711	comp123202_c0_seq1.1.311.minus.R1_1	CBM32	unclassified
712	comp123458_c0_seq1.1.524.minus.R3_1	CBM50	unclassified
713	comp123458_c0_seq1.1.524.minus.R3_1	CBM50	unclassified
714	comp124047_c0_seq1.1.695.minus.R1_1	CBM67	unclassified
715	comp12410_c0_seq1.1.695.minus.R3_1	CBM48	unclassified
716	comp124485_c0_seq1.1.545.minus.R1_1	CBM20	unclassified
717	comp124485_c0_seq1.1.545.minus.R1_1	CBM20	unclassified
718	comp124748_c0_seq1.1.342.minus.R7_1	CBM48	unclassified
719	comp124868_c0_seq1.1.341.minus.R1_1	CBM32	unclassified
720	comp124955_c0_seq1.1.385.minus.R8_1	CBM32	unclassified
721	comp127081_c0_seq1.213.760.minus.R1_1	CBM32	unclassified
722	comp127683_c0_seq1.1.320.minus.R9_1	CBM48	unclassified
723	comp128143_c0_seq1.31.556.minus.R9_1	CBM38	unclassified
724	comp129024_c0_seq1.1.583.minus.R8_1	CBM32	unclassified
725	comp129734_c0_seq1.1.529.minus.R8_1	CBM32	unclassified
726	comp130954_c0_seq1.277.405.plus.R9_1	CBM50	unclassified
727	comp13257_c0_seq1.42.692.minus.R7_1	CBM9	unclassified
728	comp135956_c0_seq1.1.312.minus.R8_1	CBM70	unclassified
729	comp136616_c0_seq1.1.333.minus.R1_1	CBM32	unclassified
730	comp13670_c0_seq1.1.632.minus.R1_1	CBM32	unclassified
731	comp136902_c0_seq1.1.487.minus.R8_1	CBM50	unclassified
732	comp136902_c0_seq1.1.487.minus.R8_1	CBM50	unclassified
733	comp136902_c0_seq1.1.487.minus.R8_1	CBM50	unclassified
734	comp137247_c0_seq1.4.363.minus.R1_1	CBM50	unclassified
735	comp137247_c0_seq1.4.363.minus.R1_1	CBM50	unclassified
736	comp137792_c0_seq1.4.612.minus.R1_1	CBM13	unclassified
737	comp137792_c0_seq1.4.612.minus.R1_1	CBM13	unclassified
738	comp139035_c0_seq1.1.348.minus.R7_1	CBM35	unclassified
739	comp139274_c0_seq1.1.446.minus.R3_1	CBM40	unclassified
740	comp139397_c0_seq1.1.430.minus.R3_1	CBM70	unclassified

741	comp139948_c0_seq1.1.464.minus.R9_1	CBM16	unclassified
742	comp140042_c0_seq1.41.340.plus.R8_1	CBM51	unclassified
743	comp140555_c0_seq1.1.318.minus.R1_1	CBM8	unclassified
744	comp140640_c0_seq1.1.468.minus.R3_1	CBM56	unclassified
745	comp140909_c0_seq1.1.354.minus.R9_1	CBM50	unclassified
746	comp141131_c0_seq1.1.376.minus.R3_1	CBM67	unclassified
747	comp142929_c0_seq1.6.308.plus.R7_1	CBM32	unclassified
748	comp142986_c0_seq1.1.372.minus.R8_1	CBM32	unclassified
749	comp143008_c0_seq1.1.419.minus.R3_1	CBM66	unclassified
750	comp143064_c0_seq1.1.397.minus.R7_1	CBM37	unclassified
751	comp143076_c0_seq1.1.489.minus.R7_1	CBM32	unclassified
752	comp143484_c0_seq1.1.293.minus.R1_1	CBM17	unclassified
753	comp143933_c0_seq1.1.423.minus.R1_1	CBM48	unclassified
754	comp144455_c0_seq1.28.545.minus.R7_1	CBM32	unclassified
755	comp14445_c0_seq1.1.392.minus.R3_1	CBM67	unclassified
756	comp144991_c0_seq1.1.323.minus.R8_1	CBM32	unclassified
757	comp145208_c0_seq1.58.417.plus.R8_1	CBM32	unclassified
758	comp145260_c0_seq1.1.498.minus.R3_1	CBM67	unclassified
759	comp145544_c0_seq1.52.306.plus.R8_1	CBM32	unclassified
760	comp14606_c0_seq1.49.633.plus.R7_1	CBM9	unclassified
761	comp146322_c0_seq1.1.489.minus.R7_1	CBM48	unclassified
762	comp147047_c0_seq1.1.501.minus.R7_1	CBM32	unclassified
763	comp147356_c0_seq1.1.510.minus.R8_1	CBM46	unclassified
764	comp148348_c0_seq1.1.350.minus.R3_1	CBM51	unclassified
765	comp150327_c0_seq1.1.479.minus.R1_1	CBM51	unclassified
766	comp150799_c0_seq1.162.668.plus.R8_1	CBM51	unclassified
767	comp150892_c0_seq1.1.325.minus.R1_1	CBM37	unclassified
768	comp151744_c0_seq1.1.173.minus.R8_1	CBM50	unclassified
769	comp151995_c0_seq1.1.344.minus.R8_1	CBM32	unclassified
770	comp152028_c0_seq1.1.354.minus.R7_1	CBM32	unclassified
771	comp152210_c0_seq1.1.623.minus.R8_1	CBM32	unclassified
772	comp152390_c0_seq1.1.370.minus.R1_1	CBM50	unclassified
773	comp15338_c0_seq2.1.479.minus.R3_1	CBM37	unclassified
774	comp157684_c0_seq1.1.526.minus.R9_1	CBM38	unclassified
775	comp158267_c0_seq1.1.425.minus.R1_1	CBM32	unclassified
776	comp15907_c0_seq1.3.305.plus.R1_1	CBM50	unclassified
777	comp15907_c0_seq1.3.305.plus.R1_1	CBM50	unclassified
778	comp159422_c0_seq1.1.553.minus.R3_1	CBM66	unclassified
779	comp159501_c0_seq1.1.626.minus.R7_1	CBM32	unclassified
780	comp159828_c0_seq1.1.587.minus.R3_1	CBM34	unclassified
781	comp1600_c0_seq1.1.367.minus.R9_1	CBM32	unclassified
782	comp160430_c0_seq1.1.347.minus.R3_1	CBM44	unclassified
783	comp160793_c0_seq1.1.326.minus.R3_1	CBM50	unclassified
784	comp160793_c0_seq1.1.326.minus.R3_1	CBM50	unclassified

785	comp161114_c0_seq1.1.317.minus.R1_1	CBM50	unclassified
786	comp161636_c0_seq1.1.397.minus.R7_1	CBM61	unclassified
787	comp16186_c0_seq1.1.481.minus.R7_1	CBM80	unclassified
788	comp162613_c0_seq1.1.355.minus.R7_1	CBM34	unclassified
789	comp163374_c0_seq1.59.336.minus.R8_1	CBM50	unclassified
790	comp163755_c0_seq1.1.317.minus.R1_1	CBM48	unclassified
791	comp16456_c0_seq1.5.678.minus.R1_1	CBM32	unclassified
792	comp164633_c0_seq1.1.553.minus.R7_1	CBM2	unclassified
793	comp16508_c0_seq1.5.595.plus.R8_1	CBM50	unclassified
794	comp165095_c0_seq1.1.439.minus.R3_1	CBM37	unclassified
795	comp165205_c0_seq1.1.423.minus.R8_1	CBM13	unclassified
796	comp165513_c0_seq1.1.329.minus.R8_1	CBM48	unclassified
797	comp165659_c0_seq1.52.420.plus.R1_1	CBM50	unclassified
798	comp16625_c0_seq1.12.323.plus.R3_1	CBM50	unclassified
799	comp166720_c0_seq1.1.383.minus.R7_1	CBM32	unclassified
800	comp167099_c0_seq1.1.380.minus.R8_1	CBM22	unclassified
801	comp167960_c0_seq1.1.294.minus.R7_1	CBM12	unclassified
802	comp168565_c0_seq1.1.418.minus.R8_1	CBM35	unclassified
803	comp16994_c0_seq1.1.424.minus.R9_1	CBM48	unclassified
804	comp170361_c0_seq1.63.313.minus.R3_1	CBM37	unclassified
805	comp171526_c0_seq1.1.436.minus.R7_1	CBM48	unclassified
806	comp171551_c0_seq1.1.339.minus.R7_1	CBM50	unclassified
807	comp172236_c0_seq1.1.521.minus.R9_1	CBM32	unclassified
808	comp172249_c0_seq1.1.436.minus.R1_1	CBM9	unclassified
809	comp172813_c0_seq1.1.487.minus.R7_1	CBM56	unclassified
810	comp174321_c0_seq1.8.493.plus.R1_1	CBM50	unclassified
811	comp174321_c0_seq1.8.493.plus.R1_1	CBM50	unclassified
812	comp175647_c0_seq1.1.346.minus.R3_1	CBM44	unclassified
813	comp175707_c0_seq1.32.319.plus.R1_1	CBM32	unclassified
814	comp176074_c0_seq1.1.414.minus.R1_1	CBM4	unclassified
815	comp176243_c0_seq1.1.316.minus.R8_1	CBM4	unclassified
816	comp177318_c0_seq1.26.487.plus.R8_1	CBM66	unclassified
817	comp178061_c0_seq1.1.312.minus.R3_1	CBM6	unclassified
818	comp178098_c0_seq1.13.435.plus.R8_1	CBM50	unclassified
819	comp178098_c0_seq1.13.435.plus.R8_1	CBM50	unclassified
820	comp178227_c0_seq1.1.323.minus.R1_1	CBM48	unclassified
821	comp179880_c0_seq1.15.287.plus.R8_1	CBM50	unclassified
822	comp180407_c0_seq1.1.585.minus.R1_1	CBM37	unclassified
823	comp181550_c0_seq1.1.471.minus.R3_1	CBM37	unclassified
824	comp182732_c0_seq1.2.427.plus.R9_1	CBM50	unclassified
825	comp18364_c0_seq1.139.570.plus.R1_1	CBM9	unclassified
826	comp183913_c0_seq1.41.541.minus.R7_1	CBM50	unclassified
827	comp184115_c0_seq1.43.514.minus.R1_1	CBM35	unclassified
828	comp184538_c0_seq1.1.474.minus.R1_1	CBM6	unclassified

829	comp185709_c0_seq1.1.438.minus.R1_1	CBM73	unclassified
830	comp186587_c0_seq1.1.324.minus.R8_1	CBM22	unclassified
831	comp186821_c0_seq1.1.466.minus.R1_1	CBM32	unclassified
832	comp187991_c0_seq1.1.512.minus.R1_1	CBM50	unclassified
833	comp188497_c0_seq1.1.517.minus.R1_1	CBM67	unclassified
834	comp189359_c0_seq1.1.528.minus.R1_1	CBM67	unclassified
835	comp18_c0_seq1.1.343.minus.R8_1	CBM34	unclassified
836	comp190449_c0_seq1.1.421.minus.R1_1	CBM32	unclassified
837	comp190530_c0_seq1.1.336.minus.R8_1	CBM40	unclassified
838	comp190681_c0_seq1.1.377.minus.R9_1	CBM32	unclassified
839	comp192690_c0_seq1.1.413.minus.R3_1	CBM49	unclassified
840	comp19316_c0_seq1.1.442.minus.R9_1	CBM13	unclassified
841	comp19379_c0_seq1.185.526.plus.R9_1	CBM22	unclassified
842	comp194305_c0_seq1.1.348.minus.R8_1	CBM58	unclassified
843	comp194483_c0_seq1.1.425.minus.R7_1	CBM50	unclassified
844	comp194483_c0_seq1.1.425.minus.R7_1	CBM50	unclassified
845	comp194943_c0_seq1.1.314.minus.R8_1	CBM48	unclassified
846	comp194968_c0_seq1.1.633.minus.R7_1	CBM51	unclassified
847	comp194968_c0_seq1.1.633.minus.R7_1	CBM51	unclassified
848	comp195228_c0_seq1.1.311.minus.R8_1	CBM61	unclassified
849	comp195897_c0_seq1.1.328.minus.R3_1	CBM48	unclassified
850	comp196204_c0_seq1.1.429.minus.R3_1	CBM32	unclassified
851	comp196909_c0_seq1.1.342.minus.R7_1	CBM32	unclassified
852	comp19779_c0_seq1.1.546.minus.R1_1	CBM38	unclassified
853	comp198650_c0_seq1.1.332.minus.R1_1	CBM22	unclassified
854	comp199435_c0_seq1.17.304.minus.R8_1	CBM35	unclassified
855	comp199468_c0_seq1.107.377.minus.R1_1	CBM52	unclassified
856	comp199537_c0_seq1.1.349.minus.R1_1	CBM12	unclassified
857	comp201017_c0_seq1.73.474.plus.R7_1	CBM50	unclassified
858	comp201017_c0_seq1.73.474.plus.R7_1	CBM50	unclassified
859	comp201614_c0_seq1.1.387.minus.R1_1	CBM35	unclassified
860	comp204119_c0_seq1.1.303.minus.R8_1	CBM50	unclassified
861	comp205859_c0_seq1.1.309.minus.R3_1	CBM37	unclassified
862	comp206662_c0_seq1.1.312.minus.R9_1	CBM38	unclassified
863	comp207390_c0_seq1.1.504.minus.R1_1	CBM67	unclassified
864	comp20901_c0_seq3.78.275.plus.R3_1	CBM6	unclassified
865	comp210383_c0_seq1.1.381.minus.R8_1	CBM50	unclassified
866	comp21071_c0_seq1.1.432.minus.R8_1	CBM35	unclassified
867	comp210853_c0_seq1.1.349.minus.R8_1	CBM22	unclassified
868	comp214167_c0_seq1.1.309.minus.R3_1	CBM32	unclassified
869	comp21482_c0_seq1.1.506.minus.R1_1	CBM54	unclassified
870	comp216676_c0_seq1.1.300.minus.R9_1	CBM12	unclassified
871	comp219435_c0_seq1.1.313.minus.R1_1	CBM32	unclassified
872	comp222170_c0_seq1.13.336.minus.R7_1	CBM35	unclassified

873	comp223276_c0_seq1.28.426.plus.R8_1	CBM5	unclassified
874	comp223539_c0_seq1.23.256.plus.R8_1	CBM50	unclassified
875	comp224110_c0_seq1.1.648.minus.R7_1	CBM54	unclassified
876	comp224432_c0_seq1.1.373.minus.R8_1	CBM66	unclassified
877	comp225140_c0_seq1.1.540.minus.R1_1	CBM50	unclassified
878	comp22662_c0_seq1.15.689.minus.R7_1	CBM50	unclassified
879	comp22737_c0_seq1.1.425.minus.R9_1	CBM12	unclassified
880	comp22753_c0_seq1.71.747.minus.R1_1	CBM9	unclassified
881	comp227809_c0_seq1.1.488.minus.R1_1	CBM50	unclassified
882	comp227809_c0_seq1.1.488.minus.R1_1	CBM50	unclassified
883	comp228469_c0_seq1.1.583.minus.R9_1	CBM50	unclassified
884	comp229119_c0_seq1.1.376.minus.R9_1	CBM50	unclassified
885	comp229119_c0_seq1.1.376.minus.R9_1	CBM50	unclassified
886	comp233438_c0_seq1.1.345.minus.R8_1	CBM54	unclassified
887	comp23436_c0_seq1.1.633.minus.R3_1	CBM16	unclassified
888	comp234742_c0_seq1.1.392.minus.R1_1	CBM51	unclassified
889	comp23734_c0_seq1.1.306.minus.R8_1	CBM32	unclassified
890	comp238817_c0_seq1.80.397.plus.R8_1	CBM11	unclassified
891	comp24428_c0_seq1.1.533.minus.R3_1	CBM32	unclassified
892	comp245946_c0_seq1.1.331.minus.R1_1	CBM26	unclassified
893	comp246660_c0_seq1.1.407.minus.R1_1	CBM32	unclassified
894	comp24702_c0_seq1.1.445.minus.R3_1	CBM40	unclassified
895	comp248155_c0_seq1.1.405.minus.R8_1	CBM48	unclassified
896	comp250115_c0_seq1.1.315.minus.R7_1	CBM8	unclassified
897	comp251837_c0_seq1.1.407.minus.R1_1	CBM50	unclassified
898	comp252826_c0_seq1.1.318.minus.R1_1	CBM22	unclassified
899	comp254315_c0_seq1.1.381.minus.R1_1	CBM9	unclassified
900	comp25868_c0_seq1.58.501.plus.R1_1	CBM6	unclassified
901	comp258724_c0_seq1.1.422.minus.R7_1	CBM32	unclassified
902	comp258729_c0_seq1.1.312.minus.R1_1	CBM48	unclassified
903	comp258822_c0_seq1.124.465.minus.R1_1	CBM12	unclassified
904	comp26081_c0_seq1.1.624.minus.R1_1	CBM22	unclassified
905	comp261164_c0_seq1.1.430.minus.R8_1	CBM48	unclassified
906	comp26190_c0_seq1.1.557.minus.R7_1	CBM15	unclassified
907	comp264160_c0_seq1.1.490.minus.R1_1	CBM66	unclassified
908	comp26624_c0_seq1.1.288.minus.R8_1	CBM50	unclassified
909	comp26849_c0_seq1.14.409.plus.R7_1	CBM50	unclassified
910	comp26849_c0_seq1.14.409.plus.R7_1	CBM50	unclassified
911	comp268516_c0_seq1.1.366.minus.R8_1	CBM38	unclassified
912	comp26949_c0_seq1.1.675.minus.R9_1	CBM46	unclassified
913	comp271807_c0_seq1.1.379.minus.R8_1	CBM50	unclassified
914	comp271807_c0_seq1.1.379.minus.R8_1	CBM50	unclassified
915	comp27260_c0_seq1.22.522.minus.R3_1	CBM32	unclassified
916	comp27506_c0_seq1.1.387.minus.R9_1	CBM69	unclassified

917	comp27551_c0_seq1.4.384.plus.R8_1	CBM50	unclassified
918	comp27551_c0_seq1.4.384.plus.R8_1	CBM50	unclassified
919	comp27551_c0_seq2.4.384.plus.R8_1	CBM50	unclassified
920	comp27551_c0_seq2.4.384.plus.R8_1	CBM50	unclassified
921	comp27829_c0_seq1.32062.32646.plus.R3_1	CBM12	unclassified
922	comp280141_c0_seq1.1.307.minus.R7_1	CBM32	unclassified
923	comp28069_c0_seq1.14.592.minus.R8_1	CBM50	unclassified
924	comp28550_c0_seq2.1.675.minus.R3_1	CBM48	unclassified
925	comp28553_c0_seq1.28.513.plus.R8_1	CBM50	unclassified
926	comp285987_c0_seq1.65.328.plus.R1_1	CBM32	unclassified
927	comp28672_c0_seq2.1.595.minus.R7_1	CBM32	unclassified
928	comp28834_c0_seq1.1.330.minus.R1_1	CBM50	unclassified
929	comp29046_c0_seq1.84.578.plus.R7_1	CBM50	unclassified
930	comp29046_c0_seq1.84.578.plus.R7_1	CBM50	unclassified
931	comp29048_c0_seq1.1.747.minus.R3_1	CBM40	unclassified
932	comp293554_c0_seq1.1.356.minus.R9_1	CBM32	unclassified
933	comp29486_c0_seq1.1.415.minus.R3_1	CBM48	unclassified
934	comp295531_c0_seq1.1.357.minus.R1_1	CBM50	unclassified
935	comp295531_c0_seq1.1.357.minus.R1_1	CBM50	unclassified
936	comp295649_c0_seq1.1.341.minus.R7_1	CBM32	unclassified
937	comp29583_c0_seq1.1.308.minus.R7_1	CBM50	unclassified
938	comp30028_c0_seq1.1.462.minus.R8_1	CBM12	unclassified
939	comp308322_c0_seq1.1.513.minus.R7_1	CBM50	unclassified
940	comp30920_c0_seq1.1.488.minus.R1_1	CBM6	unclassified
941	comp30936_c0_seq1.1.353.minus.R3_1	CBM12	unclassified
942	comp312668_c0_seq1.1.338.minus.R1_1	CBM66	unclassified
943	comp313483_c0_seq1.1.624.minus.R1_1	CBM32	unclassified
944	comp31679_c0_seq1.1.333.minus.R8_1	CBM50	unclassified
945	comp31679_c0_seq1.1.333.minus.R8_1	CBM50	unclassified
946	comp31793_c0_seq1.17.715.minus.R1_1	CBM50	unclassified
947	comp31928_c0_seq1.1.387.minus.R9_1	CBM20	unclassified
948	comp320576_c0_seq1.1.303.minus.R1_1	CBM67	unclassified
949	comp323570_c0_seq1.1.381.minus.R7_1	CBM48	unclassified
950	comp32636_c0_seq1.1.212.minus.R1_1	CBM37	unclassified
951	comp32746_c0_seq1.1.296.minus.R8_1	CBM2	unclassified
952	comp328415_c0_seq1.1.349.minus.R1_1	CBM66	unclassified
953	comp32977_c0_seq1.11.524.minus.R9_1	CBM50	unclassified
954	comp33029_c0_seq1.1.644.minus.R8_1	CBM48	unclassified
955	comp331586_c0_seq1.1.443.minus.R8_1	CBM60	unclassified
956	comp332672_c0_seq1.1.305.minus.R1_1	CBM32	unclassified
957	comp33374_c0_seq1.1.674.minus.R8_1	CBM37	unclassified
958	comp33450_c0_seq1.456.1148.minus.R1_1	CBM50	unclassified
959	comp339558_c0_seq1.1.373.minus.R1_1	CBM50	unclassified
960	comp34242_c0_seq1.2030.2674.plus.R3_1	CBM50	unclassified

961	comp34567_c0_seq1.1.538.minus.R9_1	CBM35	unclassified
962	comp346673_c0_seq1.1.294.minus.R1_1	CBM50	unclassified
963	comp347503_c0_seq1.1.284.minus.R7_1	CBM50	unclassified
964	comp34827_c0_seq5.39.576.minus.R3_1	CBM76	unclassified
965	comp34836_c0_seq1.1.500.minus.R1_1	CBM34	unclassified
966	comp35092_c0_seq1.1.487.minus.R8_1	CBM70	unclassified
967	comp35199_c0_seq1.1.658.minus.R1_1	CBM20	unclassified
968	comp35199_c0_seq1.1.658.minus.R1_1	CBM20	unclassified
969	comp35408_c0_seq4.37.579.plus.R3_1	CBM32	unclassified
970	comp35967_c0_seq1.97.387.plus.R1_1	CBM50	unclassified
971	comp36111_c0_seq1.149.616.plus.R1_1	CBM50	unclassified
972	comp36202_c0_seq2.66.374.plus.R3_1	CBM36	unclassified
973	comp36510_c0_seq1.1.650.minus.R3_1	CBM32	unclassified
974	comp37296_c0_seq1.1.549.minus.R8_1	CBM50	unclassified
975	comp37731_c0_seq1.333.942.minus.R9_1	CBM50	unclassified
976	comp37881_c0_seq2.1.341.minus.R9_1	CBM32	unclassified
977	comp37881_c0_seq3.1.412.minus.R9_1	CBM32	unclassified
978	comp38151_c0_seq1.1.370.minus.R1_1	CBM67	unclassified
979	comp392602_c0_seq1.1.311.minus.R1_1	CBM20	unclassified
980	comp394326_c0_seq1.1.424.minus.R1_1	CBM50	unclassified
981	comp40507_c0_seq1.1.470.minus.R7_1	CBM9	unclassified
982	comp40518_c0_seq1.1.367.minus.R1_1	CBM4	unclassified
983	comp40636_c0_seq1.5.334.plus.R8_1	CBM6	unclassified
984	comp40679_c0_seq2.152.850.plus.R7_1	CBM50	unclassified
985	comp41179_c0_seq1.1.659.minus.R1_1	CBM32	unclassified
986	comp415501_c0_seq1.11.333.minus.R1_1	CBM6	unclassified
987	comp415959_c0_seq1.1.330.minus.R8_1	CBM13	unclassified
988	comp43194_c0_seq1.8.202.plus.R8_1	CBM50	unclassified
989	comp43371_c0_seq2.26.646.plus.R1_1	CBM40	unclassified
990	comp43445_c0_seq1.45.539.plus.R8_1	CBM50	unclassified
991	comp43445_c0_seq1.45.539.plus.R8_1	CBM50	unclassified
992	comp43447_c0_seq1.1.1077.minus.R8_1	CBM35	unclassified
993	comp43741_c0_seq1.1.402.minus.R1_1	CBM35	unclassified
994	comp43992_c0_seq1.1.320.minus.R8_1	CBM48	unclassified
995	comp44148_c0_seq1.33.683.minus.R1_1	CBM50	unclassified
996	comp44430_c0_seq1.45.784.minus.R7_1	CBM55	unclassified
997	comp44539_c0_seq1.55.771.plus.R7_1	CBM13	unclassified
998	comp44642_c0_seq2.56.577.plus.R8_1	CBM50	unclassified
999	comp45023_c0_seq1.132.644.plus.R8_1	CBM13	unclassified
1000	comp45194_c0_seq1.1.589.minus.R1_1	CBM48	unclassified
1001	comp45245_c0_seq1.1.483.minus.R7_1	CBM37	unclassified
1002	comp45606_c0_seq1.1.409.minus.R1_1	CBM35	unclassified
1003	comp46790_c0_seq1.32.436.plus.R8_1	CBM76	unclassified
1004	comp47168_c0_seq1.173.1276.minus.R7_1	CBM46	unclassified

1005	comp47609_c0_seq1.23.520.plus.R9_1	CBM40	unclassified
1006	comp47616_c0_seq1.378.692.plus.R8_1	CBM73	unclassified
1007	comp47725_c0_seq2.1.695.minus.R8_1	CBM48	unclassified
1008	comp47987_c0_seq3.111.519.minus.R8_1	CBM69	unclassified
1009	comp49212_c0_seq3.70.504.plus.R8_1	CBM4	unclassified
1010	comp49277_c0_seq3.7.345.plus.R7_1	CBM32	unclassified
1011	comp49367_c0_seq3.5.325.plus.R8_1	CBM30	unclassified
1012	comp49414_c0_seq1.155.463.plus.R8_1	CBM36	unclassified
1013	comp50044_c0_seq13.1.504.minus.R8_1	CBM6	unclassified
1014	comp50266_c0_seq1.1.643.minus.R7_1	CBM50	unclassified
1015	comp50266_c0_seq1.1.643.minus.R7_1	CBM50	unclassified
1016	comp50266_c0_seq1.1.643.minus.R7_1	CBM50	unclassified
1017	comp507822_c0_seq1.1.478.minus.R1_1	CBM54	unclassified
1018	comp50821_c0_seq3.5312.5929.plus.R7_1	CBM12	unclassified
1019	comp50821_c0_seq3.5312.5929.plus.R7_1	CBM12	unclassified
1020	comp50821_c0_seq3.5312.5929.plus.R7_1	CBM12	unclassified
1021	comp51093_c0_seq1.1.375.minus.R1_1	CBM4	unclassified
1022	comp51130_c0_seq1.59.379.plus.R7_1	CBM30	unclassified
1023	comp52260_c0_seq1.1.1457.minus.R7_1	CBM46	unclassified
1024	comp52907_c0_seq1.1.573.minus.R1_1	CBM48	unclassified
1025	comp52921_c0_seq2.1.575.minus.R1_1	CBM37	unclassified
1026	comp52951_c0_seq1.1.574.minus.R9_1	CBM32	unclassified
1027	comp53722_c0_seq1.80.403.plus.R8_1	CBM37	unclassified
1028	comp54073_c0_seq1.1.461.minus.R7_1	CBM55	unclassified
1029	comp54608_c0_seq1.111.465.minus.R1_1	CBM50	unclassified
1030	comp54981_c0_seq3.1.369.minus.R7_1	CBM12	unclassified
1031	comp54981_c0_seq8.1.432.minus.R7_1	CBM12	unclassified
1032	comp55055_c0_seq1.1.337.minus.R1_1	CBM2	unclassified
1033	comp55211_c0_seq9.147.402.minus.R7_1	CBM67	unclassified
1034	comp55232_c0_seq5.1142.1789.plus.R7_1	CBM9	unclassified
1035	comp55308_c0_seq2.54.641.plus.R1_1	CBM9	unclassified
1036	comp55609_c0_seq2.1.590.minus.R7_1	CBM20	unclassified
1037	comp55809_c0_seq1.10.2223.minus.R8_1	CBM32	unclassified
1038	comp55825_c0_seq4.32.569.minus.R7_1	CBM76	unclassified
1039	comp56857_c0_seq1.1.1097.minus.R9_1	CBM37	unclassified
1040	comp57185_c0_seq1.8.193.plus.R1_1	CBM50	unclassified
1041	comp579841_c0_seq1.1.337.minus.R1_1	CBM9	unclassified
1042	comp58116_c0_seq6.1.592.minus.R1_1	CBM48	unclassified
1043	comp58192_c0_seq2.1.619.minus.R1_1	CBM48	unclassified
1044	comp60181_c0_seq1.488.1084.minus.R1_1	CBM13	unclassified
1045	comp60181_c0_seq1.488.1084.minus.R1_1	CBM13	unclassified
1046	comp61033_c0_seq1.46.564.plus.R9_1	CBM50	unclassified
1047	comp61152_c0_seq5.7185.8081.plus.R1_1	CBM32	unclassified
1048	comp61660_c0_seq1.24.656.plus.R1_1	CBM19	unclassified

1049	comp61837_c0_seq1.104.2806.plus.R1_1	CBM32	unclassified
1050	comp62078_c0_seq1.20.468.minus.R7_1	CBM48	unclassified
1051	comp62666_c0_seq11.2050.2733.plus.R1_1	CBM9	unclassified
1052	comp63848_c0_seq1.1.392.minus.R3_1	CBM9	unclassified
1053	comp63897_c0_seq1.1.432.minus.R9_1	CBM9	unclassified
1054	comp64485_c0_seq1.34.234.plus.R3_1	CBM5	unclassified
1055	comp65724_c0_seq1.47.1447.minus.R1_1	CBM37	unclassified
1056	comp6603_c0_seq1.1.443.minus.R7_1	CBM48	unclassified
1057	comp66137_c0_seq1.35.736.plus.R9_1	CBM50	unclassified
1058	comp66316_c0_seq1.7041.7661.plus.R1_1	CBM32	unclassified
1059	comp66426_c0_seq1.404.598.plus.R9_1	CBM5	unclassified
1060	comp67461_c0_seq1.18.524.minus.R1_1	CBM48	unclassified
1061	comp68083_c0_seq1.200.607.plus.R1_1	CBM50	unclassified
1062	comp68083_c0_seq1.200.607.plus.R1_1	CBM50	unclassified
1063	comp68211_c0_seq1.1.667.minus.R9_1	CBM13	unclassified
1064	comp70485_c0_seq1.1.577.minus.R9_1	CBM32	unclassified
1065	comp7051_c0_seq1.86.394.plus.R9_1	CBM34	unclassified
1066	comp70521_c0_seq1.1.1657.minus.R9_1	CBM67	unclassified
1067	comp70710_c0_seq1.64.576.plus.R8_1	CBM9	unclassified
1068	comp71465_c0_seq1.711.1310.minus.R7_1	CBM12	unclassified
1069	comp71784_c0_seq1.22.486.plus.R8_1	CBM32	unclassified
1070	comp72221_c0_seq1.22.651.plus.R1_1	CBM66	unclassified
1071	comp73369_c0_seq1.56.581.minus.R9_1	CBM50	unclassified
1072	comp73369_c0_seq1.56.581.minus.R9_1	CBM50	unclassified
1073	comp73369_c0_seq1.56.581.minus.R9_1	CBM50	unclassified
1074	comp73419_c0_seq1.347.580.plus.R1_1	CBM21	unclassified
1075	comp73454_c0_seq1.209.763.minus.R1_1	CBM50	unclassified
1076	comp75542_c0_seq1.1.334.minus.R3_1	CBM48	unclassified
1077	comp7594_c0_seq1.1.363.minus.R9_1	CBM48	unclassified
1078	comp76023_c0_seq1.1215.1747.minus.R1_1	CBM50	unclassified
1079	comp76023_c0_seq1.1215.1747.minus.R1_1	CBM50	unclassified
1080	comp76275_c0_seq1.1.326.minus.R8_1	CBM69	unclassified
1081	comp7627_c0_seq1.1.373.minus.R9_1	CBM6	unclassified
1082	comp77149_c0_seq1.40.396.plus.R9_1	CBM50	unclassified
1083	comp77997_c0_seq1.3.754.minus.R7_1	CBM50	unclassified
1084	comp78356_c0_seq1.1.500.minus.R3_1	CBM32	unclassified
1085	comp78627_c0_seq1.1.522.minus.R8_1	CBM13	unclassified
1086	comp79546_c0_seq1.1.474.minus.R8_1	CBM32	unclassified
1087	comp80555_c0_seq1.180.830.plus.R1_1	CBM66	unclassified
1088	comp82169_c0_seq1.37.564.plus.R7_1	CBM66	unclassified
1089	comp82800_c0_seq1.115.375.minus.R8_1	CBM50	unclassified
1090	comp83453_c0_seq1.13.603.plus.R1_1	CBM50	unclassified
1091	comp83542_c0_seq1.3.384.minus.R9_1	CBM50	unclassified
1092	comp83576_c0_seq1.8.679.minus.R1_1	CBM9	unclassified

1093	comp84697_c0_seq1.88.402.plus.R3_1	CBM16	unclassified
1094	comp85027_c0_seq1.23.571.plus.R3_1	CBM32	unclassified
1095	comp85265_c0_seq1.1.386.minus.R8_1	CBM12	unclassified
1096	comp86687_c0_seq1.50.421.plus.R1_1	CBM13	unclassified
1097	comp87417_c0_seq1.78.497.plus.R8_1	CBM32	unclassified
1098	comp87883_c0_seq1.1.776.minus.R7_1	CBM55	unclassified
1099	comp87913_c0_seq1.1.350.minus.R3_1	CBM11	unclassified
1100	comp8831_c0_seq1.18.609.minus.R9_1	CBM32	unclassified
1101	comp88630_c0_seq1.39.422.plus.R7_1	CBM14	unclassified
1102	comp89463_c0_seq1.1.552.minus.R9_1	CBM9	unclassified
1103	comp90259_c0_seq1.21.665.plus.R7_1	CBM66	unclassified
1104	comp91005_c0_seq1.54.692.minus.R8_1	CBM50	unclassified
1105	comp91005_c0_seq1.54.692.minus.R8_1	CBM50	unclassified
1106	comp91297_c0_seq1.9.608.minus.R1_1	CBM32	unclassified
1107	comp92445_c0_seq1.1.427.minus.R9_1	CBM4	unclassified
1108	comp92994_c0_seq1.8.697.minus.R1_1	CBM13	unclassified
1109	comp93625_c0_seq1.1.348.minus.R7_1	CBM34	unclassified
1110	comp9374_c0_seq1.1.633.minus.R3_1	CBM48	unclassified
1111	comp94274_c0_seq1.14.457.plus.R8_1	CBM50	unclassified
1112	comp95861_c0_seq1.100.384.plus.R8_1	CBM37	unclassified
1113	comp95989_c0_seq1.16.660.plus.R7_1	CBM9	unclassified
1114	comp96801_c0_seq1.326.985.minus.R1_1	CBM9	unclassified
1115	comp97590_c0_seq1.1.463.minus.R1_1	CBM32	unclassified
1116	comp98673_c0_seq1.1.373.minus.R1_1	CBM48	unclassified
1117	comp98777_c0_seq1.1.579.minus.R1_1	CBM48	unclassified
1118	comp98816_c0_seq1.10.300.plus.R8_1	CBM50	unclassified
