# Supplementary material for: Effects of dietary supplementation with lysozyme on the structure and function of the cecal microbiota in broiler chickens
Source: PLoS One. 2019 Jun 19;14(6):e0216748. doi: 10.1371/journal.pone.0216748 (PMC6583987; doi:10.1371/journal.pone.0216748)
Supplement: S7 Table — (PDF) [file pone.0216748.s007.pdf]

S7 Table. Taxonomy of auxiliary activities (AA) genes identified in the cecal microbiota of broilers fed a corn-based diet supplemented with 0 (R1 in gene query), 40 (R7 in gene query name), 100 (R8 in gene query name), or 200 ppm (R9 in gene query name) lysozyme or 400 ppm flavomycin (R3 in gene query name) [the gene names in query refer to those in the transcriptome dataset deposited as PRJNA523864 in NCBI Sequence Read Archive].

No	**Gene query**	**Family**	**Taxonomy**
1	comp115090_c0_seq1.1.2121.minus.R1_1	AA7	methanogenic_archaeon_mixed_culture_ISO4-G1
2	comp71672_c0_seq1.23.1210.plus.R1_1	AA6	*Coriobacteriaceae*_bacterium_CHKCI002
3	comp63711_c0_seq1.26.878.minus.R3_1	AA6	*Bacteroides_barnesiae*
4	comp55345_c0_seq1.13.1200.plus.R1_1	AA6	*Bacteroides_coprocola*
5	comp55345_c0_seq4.13.1203.plus.R1_1	AA6	*Bacteroides_coprocola*
6	comp46891_c0_seq1.31.857.minus.R8_1	AA6	*Bacteroides_coprophilus*
7	comp46891_c0_seq3.31.1225.minus.R8_1	AA6	*Bacteroides_coprophilus*
8	comp48628_c0_seq1.1.1182.minus.R3_1	AA6	*Bacteroides_plebeius*
9	comp29998_c0_seq5.50.1242.minus.R3_1	AA6	*Bacteroides*_sp._CAG
10	comp46891_c0_seq2.24.841.minus.R8_1	AA6	*Bacteroides*_sp._CAG
11	comp54838_c0_seq1.39.1238.plus.R7_1	AA6	*Bacteroides*_sp._CAG
12	comp54838_c0_seq6.16.1221.plus.R7_1	AA6	*Bacteroides*_sp._CAG
13	comp62200_c0_seq11.1041.2240.plus.R1_1	AA6	*Bacteroides*_sp._CAG
14	comp9533_c0_seq1.343.1542.minus.R3_1	AA6	*Bacteroides*_sp._CAG
15	comp60044_c0_seq7.4140.5324.plus.R1_1	AA6	*Parabacteroides*_sp._CAG
16	comp278064_c0_seq1.20.766.plus.R1_1	AA6	*Parabacteroides*_sp._D13
17	comp60044_c0_seq2.4168.5361.plus.R1_1	AA6	*Parabacteroides*_sp._D13
18	comp137868_c0_seq1.34.801.plus.R1_1	AA6	*Tannerella*_sp._6_1_58FAA_CT1
19	comp74713_c0_seq1.404.1412.minus.R7_1	AA6	*Alistipes_inops*
20	comp29185_c0_seq2.496.1378.minus.R3_1	AA6	*Alistipes*_sp._HGB5
21	comp9796_c0_seq1.78.1268.minus.R3_1	AA6	*Alistipes*_sp. norank
22	comp61078_c0_seq1.581.3106.plus.R1_1	AA6	*Clostridium*_sp._CAG
23	comp12976_c0_seq1.12.728.minus.R8_1	AA6	*Eubacteriaceae*_bacterium_CHKCI004
24	comp42650_c0_seq1.26.1438.minus.R8_1	AA7	*Eubacteriaceae*_bacterium_CHKCI004
25	comp55118_c0_seq1.1570.3039.plus.R1_1	AA4	*Eubacteriaceae*_bacterium_CHKCI004
26	comp29269_c0_seq1.1.853.minus.R3_1	AA6	[*Ruminococcus*]_*torques*
27	comp48888_c0_seq1.73.1479.plus.R8_1	AA7	*Coprococcus_catus*
28	comp60052_c0_seq1.391.2375.minus.R1_1	AA6	*Robinsoniella*_sp._KNHs210
29	comp61214_c0_seq18.54.835.minus.R1_1	AA6	*Flavonifractor_plautii*
30	comp61620_c0_seq2.1.1068.minus.R1_1	AA7	*Intestinimonas_butyriciproducens*
31	comp61505_c0_seq1.405.2282.minus.R1_1	AA3	*Faecalibacterium_prausnitzii*
32	comp56603_c0_seq1.15.1110.minus.R1_1	AA6	*Ruminococcaceae*_bacterium_AM2
33	comp36474_c0_seq1.212.1408.plus.R9_1	AA6	[*Clostridium*]_*cellulosi*
34	comp53500_c0_seq1.171.1592.plus.R3_1	AA4	uncultured_*Megasphaera*_sp.
35	comp33127_c0_seq2.45.1928.minus.R1_1	AA3	*Sedimentibacter*_sp._B4
36	comp50929_c0_seq1.117.1943.minus.R1_1	AA3	*Parasutterella_excrementihominis*

37	comp49845_c0_seq1.55.1473.plus.R7_1	AA3	*Sutterella_parvirubra*
38	comp48607_c0_seq1.22.1419.plus.R7_1	AA3	*Sutterella_wadsworthensis*
39	comp8670_c0_seq1.1.764.minus.R9_1	AA4	*Desulfovibrio_alaskensis*
40	comp35136_c0_seq1.1.782.minus.R9_1	AA6	*Desulfovibrio_piger*
41	comp58431_c0_seq1.21.1226.minus.R1_1	AA6	*Desulfovibrio_piger*
42	comp58431_c0_seq4.21.945.minus.R1_1	AA6	*Desulfovibrio_piger*
43	comp49532_c0_seq3.326.1837.minus.R8_1	AA4	Deltaproteobacterium_NaphS2
44	comp12448_c0_seq1.754.1946.minus.R1_1	AA6	Deltaproteobacterium_PSCGC_5296
45	comp36055_c0_seq1.16.1218.plus.R3_1	AA6	*Cloacibacillus_evryensis*
46	comp39860_c0_seq1.1953.3311.plus.R9_1	AA4	*Cloacibacillus_porcorum*
47	comp55311_c0_seq1.43.1309.minus.R1_1	AA6	*Mastigamoeba_balamuthi*
48	comp35646_c0_seq2.58.2064.plus.R3_1	AA6	*Mastigamoeba_balamuthi*
49	comp101703_c0_seq1.1.461.minus.R7_1	AA6	unclassified
50	comp104483_c0_seq1.31.567.plus.R1_1	AA6	unclassified
51	comp104926_c0_seq1.1.604.minus.R1_1	AA6	unclassified
52	comp107967_c0_seq1.93.524.minus.R1_1	AA6	unclassified
53	comp11543_c0_seq1.41.658.minus.R1_1	AA6	unclassified
54	comp11970_c0_seq1.38.661.minus.R1_1	AA6	unclassified
55	comp126339_c0_seq1.174.693.minus.R7_1	AA6	unclassified
56	comp129221_c0_seq1.1.394.minus.R3_1	AA6	unclassified
57	comp130834_c0_seq1.6.509.minus.R1_1	AA6	unclassified
58	comp135109_c0_seq1.104.574.plus.R8_1	AA6	unclassified
59	comp142260_c0_seq1.1.692.minus.R7_1	AA4	unclassified
60	comp14346_c0_seq1.132.725.plus.R1_1	AA6	unclassified
61	comp158693_c0_seq1.1.636.minus.R1_1	AA6	unclassified
62	comp169504_c0_seq1.1.496.minus.R3_1	AA6	unclassified
63	comp219495_c0_seq1.1.504.minus.R1_1	AA6	unclassified
64	comp29269_c0_seq2.1.355.minus.R3_1	AA6	unclassified
65	comp29577_c0_seq1.1.400.minus.R1_1	AA6	unclassified
66	comp29932_c0_seq1.306.881.minus.R7_1	AA6	unclassified
67	comp304412_c0_seq1.1.329.minus.R1_1	AA6	unclassified
68	comp32197_c0_seq1.1.594.minus.R3_1	AA4	unclassified
69	comp345719_c0_seq1.54.458.minus.R1_1	AA6	unclassified
70	comp35696_c0_seq1.3.575.plus.R3_1	AA6	unclassified
71	comp38151_c0_seq1.46.510.plus.R8_1	AA6	unclassified
72	comp39929_c0_seq1.601.1269.plus.R9_1	AA6	unclassified
73	comp41913_c0_seq1.149.685.plus.R7_1	AA6	unclassified
74	comp431391_c0_seq1.1.307.minus.R9_1	AA6	unclassified
75	comp45045_c0_seq1.6.635.plus.R8_1	AA6	unclassified
76	comp45048_c0_seq1.17.580.plus.R1_1	AA6	unclassified
77	comp45670_c0_seq1.1.529.minus.R1_1	AA6	unclassified
78	comp50106_c0_seq1.324.902.plus.R1_1	AA6	unclassified
79	comp53383_c0_seq1.955.1377.plus.R1_1	AA6	unclassified

80	comp53383_c0_seq2.955.1377.plus.R1_1	AA6	unclassified
81	comp58005_c0_seq1.592.1206.plus.R1_1	AA6	unclassified
82	comp58424_c0_seq1.1.384.minus.R1_1	AA6	unclassified
83	comp58534_c0_seq1.114.536.plus.R1_1	AA6	unclassified
84	comp61009_c0_seq3.193.615.plus.R1_1	AA6	unclassified
85	comp64207_c0_seq1.48.633.minus.R3_1	AA6	unclassified
86	comp68335_c0_seq1.32.561.minus.R9_1	AA6	unclassified
87	comp71132_c0_seq1.21.515.plus.R1_1	AA6	unclassified
88	comp73038_c0_seq1.11.550.plus.R7_1	AA6	unclassified
89	comp7396_c0_seq1.94.516.plus.R3_1	AA6	unclassified
90	comp77967_c0_seq1.147.538.minus.R1_1	AA6	unclassified
91	comp79891_c0_seq1.667.1098.plus.R1_1	AA6	unclassified
92	comp81118_c0_seq1.152.556.plus.R7_1	AA6	unclassified
93	comp82956_c0_seq1.51.473.minus.R1_1	AA6	unclassified
94	comp84088_c0_seq1.446.1002.minus.R1_1	AA6	unclassified
95	comp88866_c0_seq1.1.686.minus.R7_1	AA4	unclassified
96	comp91105_c0_seq1.328.753.minus.R8_1	AA6	unclassified
97	comp94653_c0_seq1.1.359.minus.R9_1	AA6	unclassified
98	comp97466_c0_seq1.62.652.minus.R1_1	AA6	unclassified
99	comp99199_c0_seq1.612.1129.minus.R1_1	AA6	unclassified
100	comp99889_c0_seq1.6.473.minus.R1_1	AA6	unclassified
