# Supplementary material for: Effects of dietary supplementation with lysozyme on the structure and function of the cecal microbiota in broiler chickens
Source: PLoS One. 2019 Jun 19;14(6):e0216748. doi: 10.1371/journal.pone.0216748 (PMC6583987; doi:10.1371/journal.pone.0216748)
Supplement: S8 Table — (PDF) [file pone.0216748.s008.pdf]

S8 Table. Composition of dominant bacterial species (with average percentage abundance >0.5% in all caecal samples) and their distribution in the caeca of broiler chickens fed a corn-based diet supplemented with 40, 100 and 200 ppm lysozyme or 400 ppm flavomycin.

Species	Operational taxonomic unit (OUT)	Control	400 ppm flavomycin	40 ppm lysozyme	100 ppm lysozyme	200 ppm lysozyme
**Uncultured *Clostridiales* bacterium**	OTU319	5.0(±3.6)%	6.1(±5.0)%	5.7(±1.7)%	5.1(±3.2)%	5.0(±2.8)%
**Unclassified *Lactobacillus* bacterium**	OTU326	3.2(±3.4)%	1.7(±1.8)%	4.0(±4.9)%	4.5(±6.4)%	5.6(±5.6)%
**Unclassified *Lactobacillus* bacterium**	OTU305	3.4(±3.7)%	1.5(±1.6)%	4.6(±5.3)%	4.3(±6.1)%	4.9(±4.7)%
**Unclassified *Phascolarctobacterium* bacterium**	OTU460	2.6(±3.2)%	5.1(±5.9)%	2.2(±2.7)%	3.0(±3.4)%	6.6(±6.1)%
**Unclassified *Ruminococcus* bacterium**	OTU188	2.4(±1.9)%	3.2(±3.7)%	3.2(±1.3)%	4.0(±3.4)%	3.7(±2.5)%
Bacteroides caecicola	OTU499	4.9(±5.6)%	2.6(±3.5)%	2.9(±5.3)%	4.2(±5.8)%	3.2(±3.7)%
**Uncultured *Bacteroides* bacterium**	OTU476	2.7(±4.0)%	4.4(±6.3)%	1.3(±2.1)%	5.2(±5.9)%	2.3(±3.2)%
**Uncultured *Faecalibacterium* bacterium**	OTU345	8.2(±14.2)%	0.4(±0.4)%	0.7(±1.3)%	1.0(±1.3)%	1.3(±1.4)%
Lactobacillus aviarius	OTU73	6.7(±6.6)%	0.9(±0.9)%	1.5(±1.5)%	1.1(±1.0)%	1.3(±1.3)%
***Alistipes* sp. CHKCI003**	OTU328	0.7(±0.5)%	0.6(±0.6)%	2.7(±4.6)%	6.1(±9.6)%	1.2(±1.9)%
Bifidobacterium saeculare	OTU25	0.5(±0.8)%	7.6(±8.3)%	0.9(±0.9)%	0.3(±0.3)%	1.3(±2.4)%
***Bacteroides coprocola* DSM_17136**	OTU59	2.9(±4.6)%	2.8(±3.6)%	1.7(±2.6)%	3.3(±4.3)%	1.2(±2.0)%
**Unclassified *Megamonas* bacterium**	OTU462	1.7(±2.3)%	4.4(±4.9)%	2.1(±2.3)%	1.0(±1.2)%	2.7(±2.9)%
**Uncultured *Faecalibacterium* bacterium**	OTU262	2.5(±2.1)%	3.0(±1.3)%	0.8(±0.6)%	3.3(±2.5)%	1.2(±0.9)%
**Unclassified *Lachnospiraceae* bacterium**	OTU207	0.8(±1.1)%	1.5(±1.5)%	2.2(±2.4)%	1.9(±1.7)%	2.7(±3.4)%
**Uncultured *Synergistes* bacterium**	OTU278	0.5(±0.5)%	5.6(±10.2)%	1.4(±2.5)%	1.1(±2.1)%	2.0(±3.1)%
Desulfovibrio piger	OTU486	2.3(±3.8)%	1.1(±1.5)%	2.0(±3.0)%	2.1(±3.4)%	1.1(±1.7)%
***Barnesiella viscericola* DSM_18177**	OTU52	0.6(±0.7)%	1.0(±1.4)%	4.3(±7.9)%	0.8(±0.9)%	1.2(±2.1)%
**Unclassified *Bacteroides* bacterium**	OTU377	0.5(±0.8)%	1.3(±1.9)%	0.5(±0.5)%	2.4(±4.0)%	2.6(±4.2)%
**Unclassified *Lactobacillus* bacterium**	OTU69	1.3(±1.3)%	0.4(±0.6)%	1.0(±1.3)%	0.9(±0.6)%	2.3(±2.1)%
***Butyricicoccus pullicaecorum* 1.2**	OTU23	1.3(±1.4)%	0.8(±1.2)%	1.1(±1.5)%	1.2(±1.9)%	1.3(±2.0)%
**Unclassified *Lachnospiraceae* NK4A136_group bacterium**	OTU312	0.6(±0.8)%	1.2(±1.5)%	1.2(±1.5)%	1.0(±1.4)%	1.2(±1.6)%

**Uncultured *Anaerotruncus* bacterium**	OTU76	0.7(±0.5)%	0.9(±1.1)%	1.2(±1.4)%	1.2(±1.5)%	1.3(±1.8)%
Lactobacillus salivarius	OTU11	0.7(±0.5)%	1.0(±1.0)%	0.8(±0.9)%	1.2(±1.8)%	1.6(±2.3)%
Anaerostipes butyraticus	OTU260	0.8(±0.1)%	1.4(±1.7)%	1.5(±0.7)%	0.9(±0.8)%	0.6(±0.5)%
Bacteroides gallinaceum	OTU57	2.2(±2.7)%	0.3(±0.3)%	1.1(±1.6)%	0.8(±0.9)%	1.7(±2.1)%
**Unclassified *Lachnospiraceae* bacterium**	OTU194	0.9(±1.0)%	0.8(±0.8)%	1.0(±0.5)%	1.1(±1.0)%	0.9(±0.9)%
**Unclassified *Blautia* bacterium**	OTU270	1.1(±0.8)%	0.9(±0.9)%	1.6(±1.5)%	0.4(±0.2)%	0.6(±0.4)%
**Unclassified *Alistipes* bacterium**	OTU497	1.0(±1.1)%	1.3(±2.0)%	1.1(±1.2)%	0.9(±1.0)%	0.9(±1.0)%
Bacteroides coprophilus	OTU156	0.3(±0.4)%	3.2(±3.7)%	0.0(±0.1)%	0.4(±0.5)%	0.7(±1.0)%
**Uncultured *Parabacteroides* bacterium**	OTU54	0.9(±1.5)%	0.7(±1.3)%	1.7(±2.0)%	0.9(±1.6)%	0.5(±0.9)%
**Unclassified *Lachnoclostridium* bacterium**	OTU309	0.6(±0.6)%	0.9(±0.8)%	0.7(±0.3)%	0.7(±0.5)%	0.9(±0.7)%
**Unclassified *Lachnospiraceae* bacterium**	OTU386	0.9(±0.8)%	0.9(±1.0)%	0.9(±0.6)%	0.6(±0.4)%	0.6(±0.4)%
**Unclassified *Subdoligranulum* bacterium**	OTU117	0.8(±0.7)%	0.5(±0.6)%	0.5(±0.6)%	0.5(±0.4)%	1.4(±0.8)%
**Uncultured *Subdoligranulum* bacterium**	OTU314	0.8(±1.0)%	0.5(±0.6)%	0.5(±0.4)%	1.1(±1.8)%	0.4(±0.4)%
**Unclassified *Sellimonas* bacterium**	OTU310	0.4(±0.5)%	0.5(±0.5)%	0.9(±0.7)%	0.8(±1.1)%	0.6(±0.7)%
**Unclassified *Escherichia* bacterium**	OTU322	1.0(±1.6)%	0.4(±0.6)%	0.6(±0.6)%	0.8(±0.8)%	0.6(±0.6)%
Blautia hydrogenotrophica	OTU187	0.2(±0.1)%	0.6(±0.6)%	0.7(±0.8)%	0.6(±0.7)%	1.0(±1.1)%
Bacteroides plebeius	OTU496	0.4(±0.6)%	0.5(±0.7)%	1.0(±1.3)%	1.0(±1.8)%	1.2(±2.1)%
**Uncultured *Eisenbergiella* bacterium**	OTU189	0.3(±0.2)%	0.9(±0.9)%	0.7(±0.6)%	0.8(±0.7)%	0.5(±0.4)%
**Unclassified bacterium_ic1391**	OTU203	0.7(±0.8)%	0.6(±0.5)%	0.5(±0.4)%	0.6(±0.6)%	0.4(±0.4)%
***Clostridiales* bacterium CHKCI001**	OTU103	0.5(±0.7)%	0.6(±0.3)%	1.5(±1.4)%	0.4(±0.6)%	0.5(±0.5)%
**Unclassified *Bacteroides* bacterium**	OTU475	0.2(±0.3)%	0.7(±0.8)%	0.3(±0.5)%	1.0(±1.5)%	1.2(±1.9)%
**Unclassified *Lachnospiraceae* bacterium**	OTU132	0.2(±0.3)%	0.9(±1.3)%	0.8(±1.3)%	0.4(±0.2)%	0.1(±0.1)%
