# Supplementary figures and images for: Effects of dietary supplementation with lysozyme on the structure and function of the cecal microbiota in broiler chickens
Source: PLoS One. 2019 Jun 19;14(6):e0216748. doi: 10.1371/journal.pone.0216748 (PMC6583987; doi:10.1371/journal.pone.0216748)

▼ Down-fdr-0.01   ▼ Down-fdr-0.05   ◆ No significance   ▲ Up-fdr-0.05   ▲ Up-fdr-0.01

(A) Flavomycin

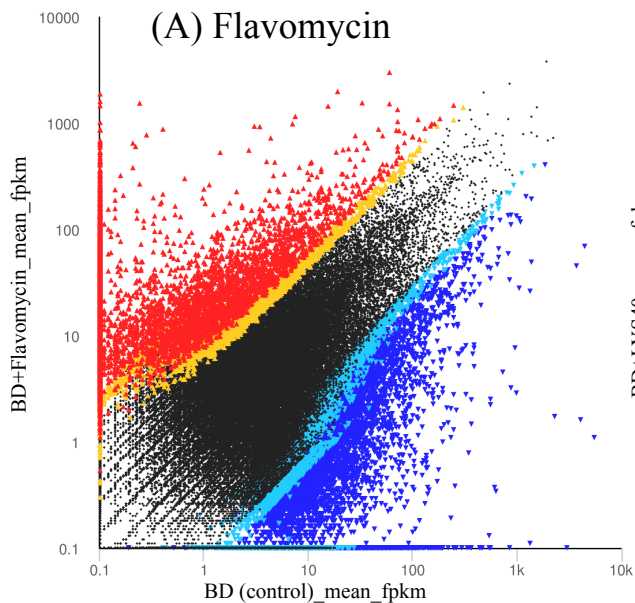

(B) LYS40

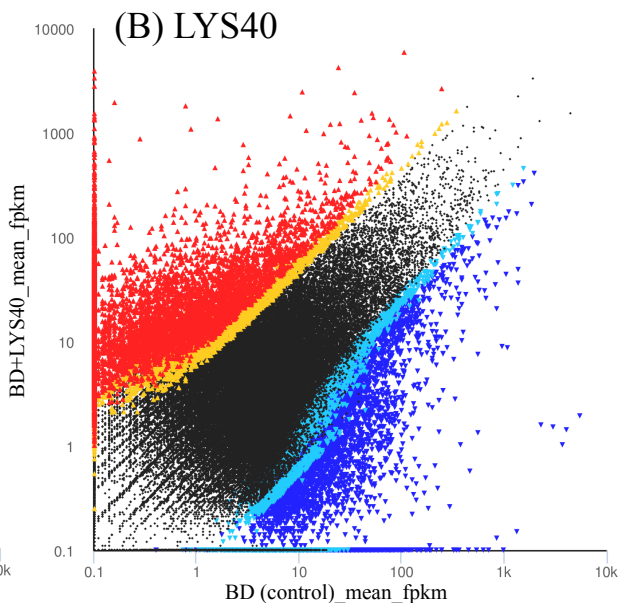

(C) LYS100

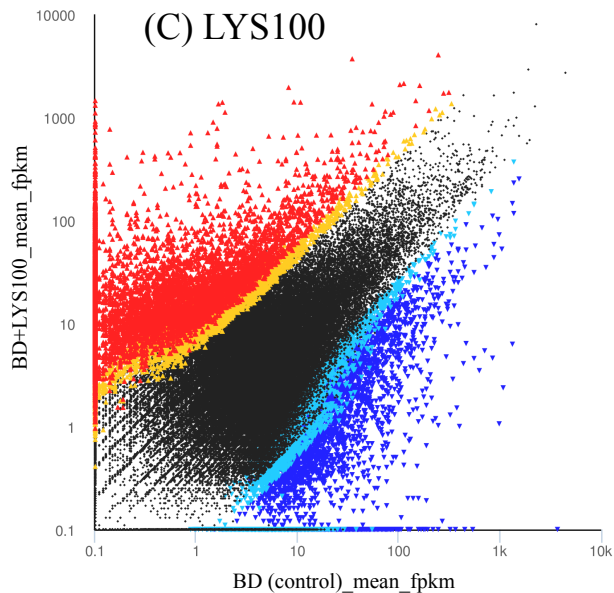

(D) LYS200

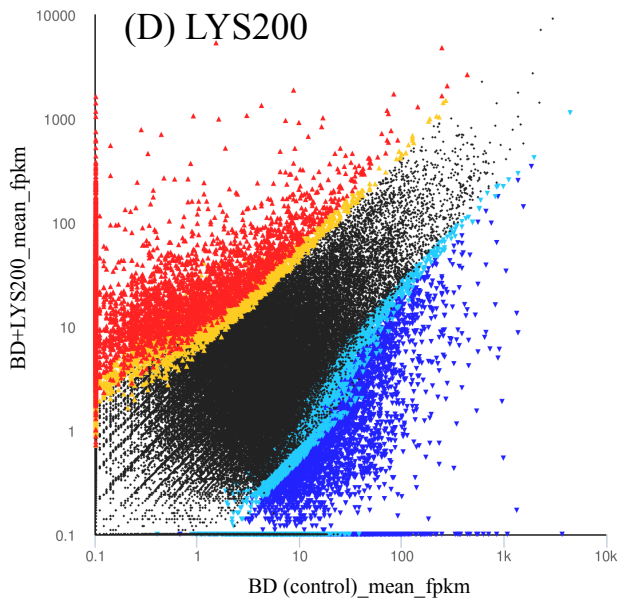

Supplement: S1 Fig — (PDF) [file pone.0216748.s009.pdf]
